# Three new species in the harvestmen genus *Acuclavella* (Opiliones, Dyspnoi, Ischyropsalidoidea), including description of male *Acuclavella quattuor* Shear, 1986

**DOI:** 10.3897/zookeys.311.2920

**Published:** 2013-06-20

**Authors:** Casey H. Richart, Marshal Hedin

**Affiliations:** 1 Department of Biology, San Diego State University, 5500 Campanile Drive, San Diego, California, 92182, USA

**Keywords:** Molecular phylogenetics, morphometrics, Pacific Northwest, northern Idaho, Salmon River, Olympic Peninsula, species delimitation, cybertaxonomy

## Abstract

In Shear’s (1986) cladistic analysis of the Ischyropsalidoidea, he described the new genus *Acuclavella* including four new species from the Pacific Northwest states of Washington and Idaho. Several of these species descriptions were based on very limited sample sizes. Our recent field work has increased by more than an order of magnitude both the number of specimens and known localities for *Acuclavella*. We use this new material to interpret species limits in *Acuclavella* using morphometric analyses and DNA sequence data from four gene regions. We sequence for the first time the protein-coding homolog of the Wnt2 gene for phylogenetic reconstruction in Opiliones. Our multi-locus phylogeny corroborates a sister relationship between *Acuclavella* and *Ceratolasma*, as hypothesized using morphology by Shear (1986). Within *Acuclavella*, morphometric clusters and reciprocal allelic monophyly allows recognition of three additional species: *Acuclavella leonardi* **sp. n.**, *Acuclavella sheari* **sp. n.**, and *Acuclavella makah* **sp. n.** This work also describes the previously unknown male of *Acuclavella quattuor*, from specimens collected at the type locality. Our research identifies a number of novel morphologies for *Acuclavella*, including females with four pairs of spines, individuals with three pairs of spines on scute areas I-III, and a population with two pairs of spines disjunct from *Acuclavella quattuor*, which was diagnosed with this spination character. We were unable to assign these populations to existing species, and conservatively do not yet recognize them as new. Intrageneric morphometrics and phylogenetic inference in *Acuclavella* were often concordant. However, we demonstrate that species delimitation signal would not be detected if only a single line of evidence were utilized.

## Introduction

The genus *Acuclavella* was described in 1986 by Shear in his revision of the superfamily Ischyropsalidoidea (Opiliones, Dyspnoi). In this work, four new species in the genus were described (Shear 1986). These descriptions were based in large part on very small sample sizes: *Acuclavella cosmetoides* Shear, 1986, the generic type species, was described from a single male and female; *Acuclavella shoshone* Shear, 1986 was described from two males and one female; and *Acuclavella quattuor* Shear, 1986 was described from a single female. Each of these three species was known only from their respective type localities in northern Idaho, north of the Salmon River. The fourth species, *Acuclavella merickeli* Shear, 1986, was represented by thirteen individuals. Twelve of these (four males and eight females) were from the type locality also in Idaho north of the Salmon River. The remaining individual was collected from the Cascade Mountains of western Washington, and due to morphological similarity, was considered by Shear to be conspecific with *Acuclavella merickeli* despite a geographic separation of over 500 kilometers.

The biogeographic situation in *Acuclavella*, with several short-range endemic species from a small geographic area, coupled with an apparently widespread (but disjunct) species, clearly invites further investigation. The biogeographic barriers separating the Cascade Mountains from the Rocky Mountains of northern Idaho have promoted speciation in a variety of taxa (e.g., amphibians: Nielson et al. 2001, Steele et al. 2005; arachnids: Derkarabetian et al. 2010; insects: Barr 2011). In addition, recent surveys have uncovered populations of *Acuclavella* from the Olympic Peninsula of northwestern Washington State, and from south of the Salmon River in Idaho. Cascade Mountain and Olympic Peninsula ecoregions of western Washington have been shown to house distinct species of non-vagile organisms (e.g., Good and Wake 1992). In Idaho, a habitat corridor of *Abies grandis*, north-facing and high above the south side of the Salmon River, may house unique populations of organisms that survived in an unrecognized compartment of a structured Pleistocene glacial refugium (Brunsfeld and Sullivan 2006).

The goal of this paper is to use morphometrics and molecular phylogenetics to investigate the validity of the four species hypothesized by Shear (1986), to delimit potentially new species within *Acuclavella*, and to revise the genus as necessary. We hypothesize that molecular phylogenies will reveal consistent reciprocal monophyly conforming to Idaho versus Washington taxa. Furthermore, we expect that *Acuclavella* populations from the two ecoregions in western Washington will show evidence of molecular divergence. In Idaho, expectations are that a morphologically distinct new population discovered south of the Salmon River will show molecular phylogenetic divergence from the four northern species described by Shear (1986).

## Methods

### Taxon sampling

Most fieldwork was conducted in the summer of 2008, with additional adult specimens collected from May to September in 2006, 2007, and 2009 (Appendix I - Collection Locality Information). *Acuclavella* are crenophilic denizens of small, perennial water features such as headwater streams and seeps in the *Tsuga heterophylla* Zone and the coastal *Picea sitchensis* Zone of the Pacific Northwest (Franklin and Dyrness 1988). Appropriate habitat and microhabitat was targeted for specimen collection throughout northern Idaho and western Washington, and collections include all type localities. When encountered, at least one specimen from each locality was preserved for molecular analyses by placing specimens into 100% EtOH, and subsequently transferring specimens to a -80° C freezer (following Vink et al. 2005).

### Molecular data collection

Genomic DNA was extracted from two legs per specimen using the Qiagen DNeasy kit, per manufacturer’s protocol. Currently, few genes are available for resolving shallow phylogenetic relationships in Opiliones (reviewed in Hedin et al. 2010). In this study, PCR amplification targeted four gene regions, including mitochondrial protein-coding cytochrome oxidase I (COI), the 28S large subunit ribosomal RNA (28S), nuclear protein-coding Elongation Factor-1 alpha (EF-1α), and the nuclear protein-coding *wingless* (Wnt2). COI is a standard gene used for species-level delimitation in animals, and has been used extensively in opilionid phylogenetic studies (e.g. Boyer et al. 2005, 2007; Thomas and Hedin 2008; Hedin and Thomas 2010; Derkarabetian et al. 2011). 28S rRNA data has been used successfully to elucidate deeper relationships in opilionid phylogenies (e.g. Boyer et al. 2007, Derkarabetian et al. 2010, Giribet et al. 2010, Schönhofer and Martens 2010, Sharma and Giribet 2011), but generally evolves too slowly to resolve shallow relationships (e.g. Hedin and Thomas 2010). EF-1α has been shown to have phylogenetic utility at two levels (Hedin et al. 2010); in the region amplified for *Acuclavella*, more slowly evolving exons bracket a relatively rapidly evolving intron. The Wnt2 gene region has been used to reconstruct spider phylogenies (e.g., Blackledge et al. 2009, Satler et al. 2011), but is used here for the first time in Opiliones. PCR primer and amplification conditions are available as a supplementary file (Appendix II - PCR Primer Information). Amplicons were visualized on agarose gels, purified via polyethylene glycol (PEG) precipitation or on Millipore plates, and Sanger sequenced at the San Diego State University Microchemical Core Facility (http://www.sci.sdsu.edu/dnacore/sdsu_dnacore.html) or at Macrogen USA.

### Phylogenetic analyses

Bi-directional Sanger reads were assembled into contiguous sequences using Sequencher v4.5 (Gene Codes Corporation, MI). EF-1α haplotypes were reconstructed in PHASE (Stephens and Donnelly 2003) using an ingroup-only matrix with the following parameter settings: 100 iterations, thinning interval of 1, burn-in of 100, probability cut-off set to 0.70 (Harrigan et al. 2008). Haplotypes with nucleotides inferred with probability values < 0.70 were left ambiguous. The PHASE input file was converted from a FASTA alignment using SeqPHASE (Flot 2010). COI, EF-1α exon, and Wnt2 gene regions were unambiguously aligned using amino acid translations in MacClade v4.06 (Maddison and Maddison 2003). EF-1α intron and 28S rRNA data were aligned using MAFFT (Katoh et al. 2005), utilizing the Q-INS-i alignment algorithm strategy with parameters: BLOSUM62 alignment scoring matrix, 200PAM / K=2 scoring matrix value, an opening gap penalty of 1.53, and an offset value of 0.1. Regions of alignment uncertainty were removed from the 28S matrix with Gblocks (Castresana 2000) with the following parameter settings: minimum length of a block = 3, allowed gap position with half, minimum number of sequences used to identify a conserved position and a flanking position 11 and 17 respectively, and 8 as the maximum number of contiguous non-conserved positions.

Individual gene trees were reconstructed using maximum likelihood and Bayesian inference. Bayesian analyses were implemented using MrBayes v3.1.2 (Ronquist and Huelsenbeck 2003) run on an XSEDE utilizing the CIPRES portal (Miller et al. 2010). Models of DNA sequence evolution used in Bayesian analyses were determined using jModelTest v0.1.1 (Posada 2008, Guindon and Gascuel 2003). Likelihood scores were computed with three substitution schemes (24 models), unequal base frequencies, proportion of invariable sites, rate variation among sites, and used the BIONJ algorithm of Gascuel (1997). The goodness of fit of alternative models was determined using AIC (Akaike 1974) with CI set to 100%. Bayesian gene tree analyses were run for 5 X 10^6^ generations sampling every 1000 trees, using best-fit models of molecular evolution. At this many generations, the average standard deviation of split frequencies between runs was < 0.01. The first 40% of the sampled trees were discarded as burn-in, and stationarity was confirmed visually using Tracer v2.4 (Rambaut and Drummond 2007). Maximum likelihood gene tree analyses were conducted using RAxML v7.2.8 (Stamatakis 2006), also on the CIPRES portal using the GTR + GAMMA model for tree inference and bootstrapping (Stamatakis et al. 2008). The EF-1α data set was partitioned by intron and exon, and the COI data was partitioned by codon position. The Wnt2 and 28S matrices were not partitioned. Outgroup sequences were used to root all gene trees.

All available DNA sequences, with the exception of apparently nuclearized copies of COI (see Results), were concatenated for phylogeny reconstruction. The concatenated matrix was analyzed using both maximum likelihood (RAxML) and Bayesian approaches (MrBayes v3.1.2) using a seven-partition strategy (EF-1α intron + exon, Wnt2, 28S, individual COI codon positions). The Bayesian analysis was run for 1 X 10^7^ generations; tree sampling and burn-in were as above. Outgroup sequences for five of six ischyropsalidoid genera (*Ceratolasma*, *Taracus*, *Hesperonemastoma*, *Sabacon*, and *Ischyropsalis*) and two genera from Troguloidea (*Dendrolasma* and *Ortholasma*) were used in concatenated phylogenetic analyses. These sequences were generated in-house or downloaded from GenBank (see Table 1).

### Morphometric analyses

Specimen measurements were taken using an Olympus SZX12 dissecting microscope with an ocular micrometer. Individuals with missing data (i.e., no Leg II) were excluded from analyses. Standard measurements (Acosta et al. 2007) of 16 characters were determined for 261 individuals (131 males, 133 females); the measurement data, including a figure showing our character measurements scheme, are available as a supplementary file (Appendix III). Total body length was not measured due to the variation in length allowed by free tergites and sternites in individuals that are gravid or have recently eaten; this measurement is known to vary with nutritional condition in Opiliones (Acosta and Machado 2007).

Principle components analyses (PCA) and discriminant function analyses (DFA) were carried out using SYSTAT 12 (Systat Software, Inc.). Though both PCA and DFA are multivariate analyses, DFA is a validation approach that deals specifically with the problem of separating predetermined groups. In taxonomy, DFAs are almost exclusively used to differentiate between morphologically similar species that are difficult to identify from single characteristics (see Klimov et al. 2004, Seifert 2003, Fisher 1936, Lubishew 1962). Multispecies delimitation via DFA, as done here, is becoming more common (see Schlick-Steiner et al. 2006, Schönhofer and Martens 2008, Gebiola et al. 2012).

In taxonomy, PCAs are regularly employed for species delimitation. This utilization has ranged from comparing the vector angles in a scatter plot of predefined species groups (Hamm 2010), to mathematical inference of groups without *a priori* assignment of samples to species (Ezard et al. 2010). Because a PCA has no intrinsic measure of group exclusivity, the parameters of group recovery in morphospace need to be defined. For purposes of this research we defined three PCA categories: a “recovered” group clustered together and did not overlap with samples from other groups; a “nearly recovered” group clustered together with minimal overlap with other groups (one or two individuals at the periphery of their respective group that are not enclosed within another); a “not recovered” group failed to cluster exclusively. For these analyses, three methods were employed to explore this morphospace: (1) hypothesized species were compared pairwise; (2) all combinations of principle components explaining a significant part of the variation in the data were explored; and (3) a nested approach where if clusters of hypothesized species were recovered by initial analyses, further analyses were conducted on those clusters. An in-depth discussion of morphometric methods is available as a supplementary file (Appendix IV).

Due to sexual dimorphism, male and female specimens were analyzed separately for all morphometric analyses. Since the data used to conduct PCAs were measured in the same units (mm), analyses were conducted on both correlation and covariance matrices. PCAs using covariance matrices tend to be dominated by characters showing the most variability (Jolliffe 2002). In order to reduce possible excessive influence on the principle components, variables were coded to have a mean of zero and variance of one; analyses on this data are based on a correlation matrix. Analyses run on covariance matrices assume a multivariate normal distribution. This is often violated in taxonomic studies where data consists of multiple species of varying morphological distinctiveness; outliers could represent members of an under-sampled group. All unusual measurements were confirmed by repeated measurement, then included in analyses under the assumption they do not excessively affect interpretation of the data.

### Species delimitation

We initiated analyses with seven *a priori* hypothesized species, with species-level distinctions based on geographic criteria or on preliminary specimen sorting (i.e., not morphometrics). Geographic criteria were based on the results of work on amphibians endemic to the disjunct mesic forests of the Pacific Northwest (Nielson et al. 2001), or the physiogeographic regions of western Washington (Good and Wake 1992). Areas of expected endemism included western Washington versus northern Idaho, as well as between Cascade Mountain and Olympic Peninsula regions in Washington, areas separated by the low valley of the Puget Trough. In Idaho, a morphologically diminutive population was discovered south of the Salmon River; this group was also tested as a species.

**Figure 1.**
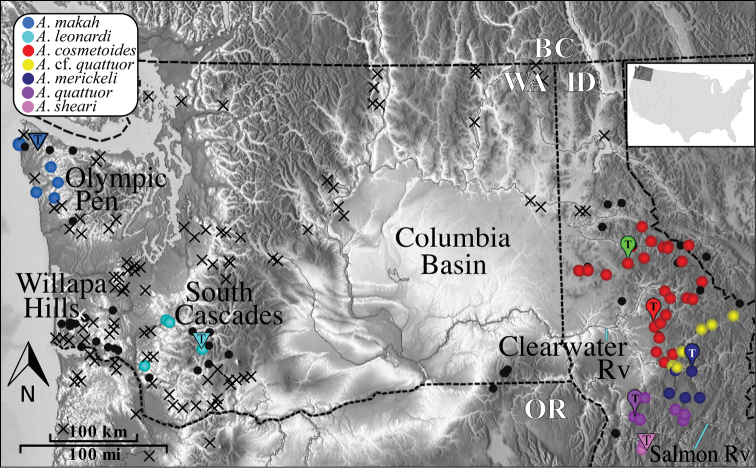
Distribution of *Acuclavella*. Black circles are localities where *Acuclavella* were specifically targeted, but not encountered. Black crosses indicate localities where general surveys of litter invertebrates (e.g., Opiliones, Diplopoda, terrestrial Gastropoda) were conducted and *Acuclavella* were not encountered. Blue lines point to labeled rivers. Type localities (T) from Shear (1986) are indicated with paddle icons; green paddle = *Acuclavella shoshone*. Type localities of species described in this work are indicated with inverted triangles. Image saved from Google Earth.

**Figure 2.**
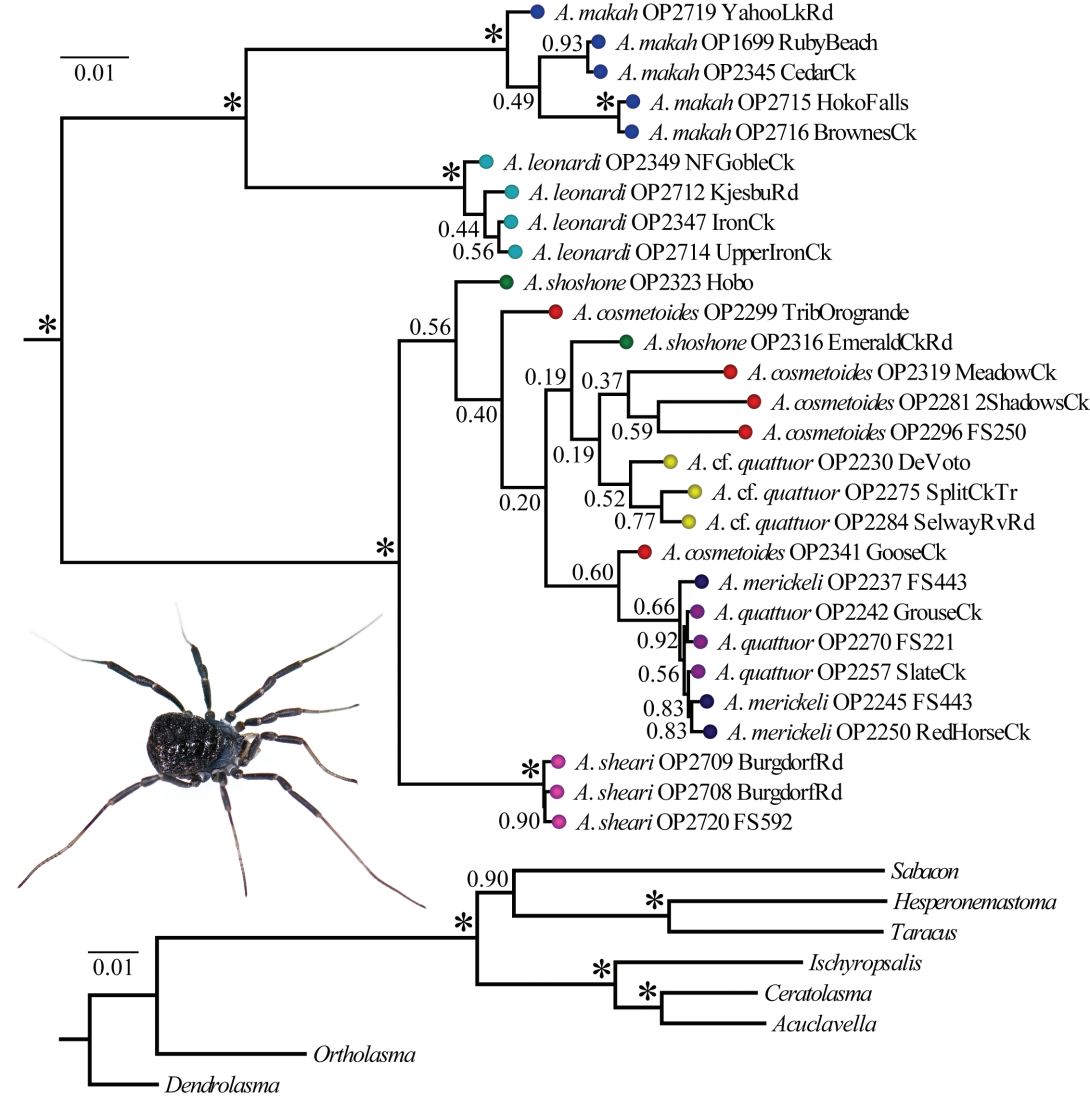
Bayesian phylogram resulting from analysis of concatenated dataset. Numbers at nodes correspond to Bayesian posterior probabilities. The outgroup topology is shown at bottom. The inset picture is a female *Acuclavella shoshone* (*sensu stricto*) collected from the type locality (Shear 1986).

**Figure 3.**
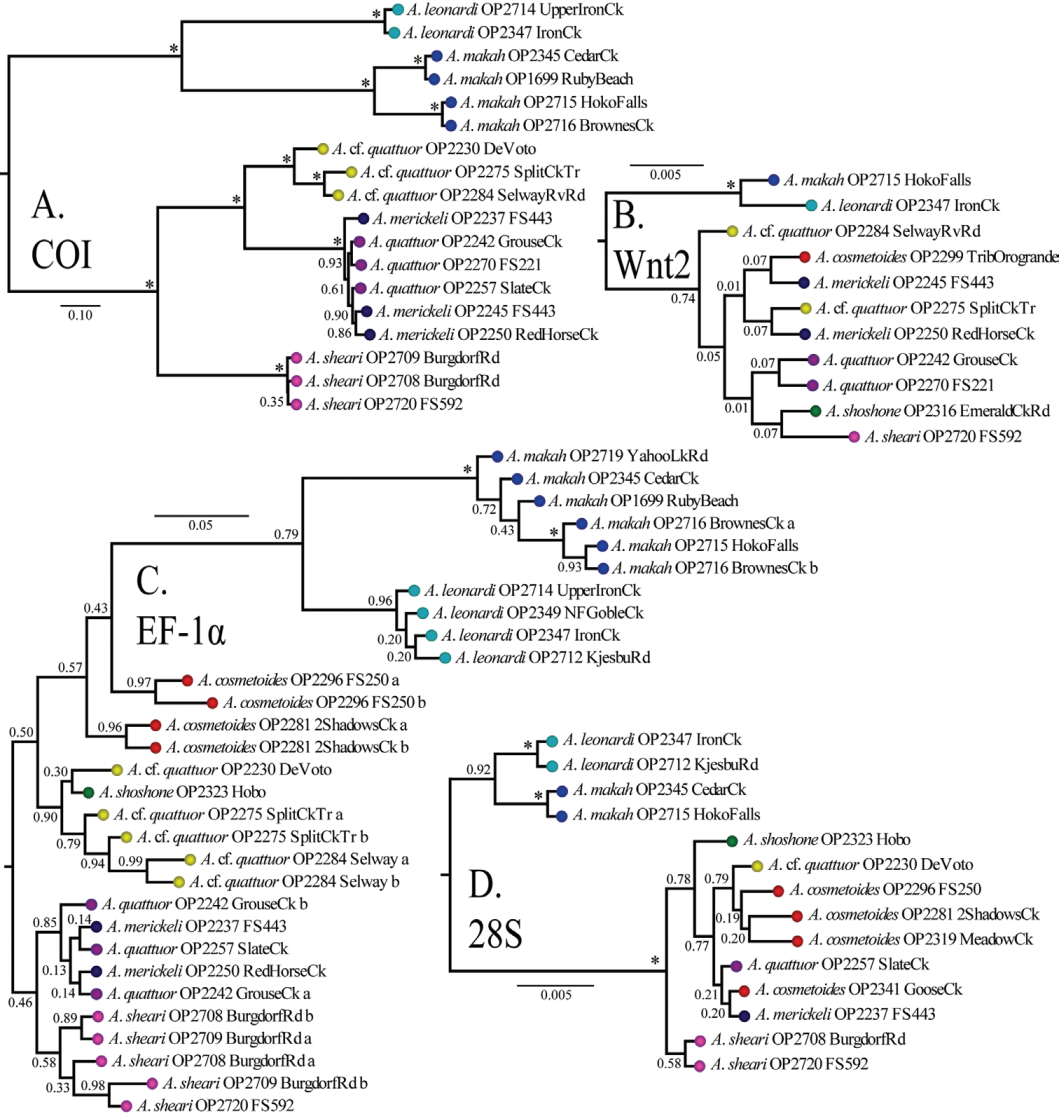
Bayesian gene trees with posterior probabilities: **A** COI **B** Wnt2 **C** EF-1α **D** 28S. All trees outgroup rooted (Appendix VII, Figure 1). RAxML concatenated phylogeny (Appendix VII, Figure 2) and gene trees (Appendix VII, Figures 3-5) are also available.

**Figure 4.**
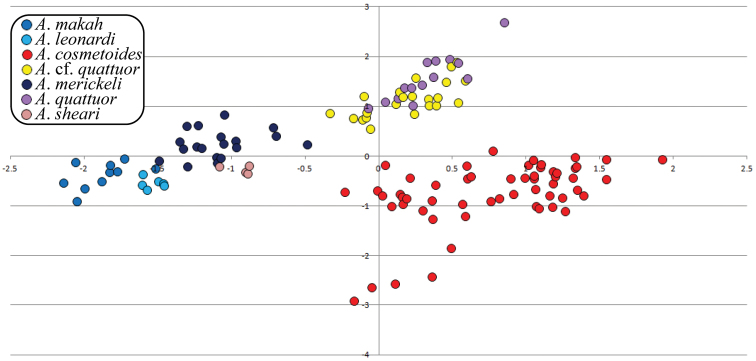
PCA bi-dimensional plot. PCA results of male data using a covariance matrix; plot of principle components 2 and 3.

North of the Salmon River in Idaho, *Acuclavella merickeli* and *Acuclavella quattuor* were distinguished using the diagnostic features outlined by Shear (1986). Due to a gradation of intermediate morphologies spanning the diagnostic characters (Shear 1986) for *Acuclavella cosmetoides* and *Acuclavella shoshone* (Appendix V: males Figure 1, females Figure 2), coupled with the observation that the described morphologies of these species more or less occur only at the type localities (Appendix V: males, Figure 3; females, Figure 4), an initial attempt to distinguish these two species was abandoned. Since *Acuclavella cosmetoides* is the type species for the genus, all morphotypes north of the Middle Fork Clearwater were preliminarily identified as *Acuclavella cosmetoides*. A newly discovered population in northern Idaho matched the diagnostic morphology of *Acuclavella quattuor* of having pairs of spines on scute areas I and II. However, the new population is distributed between the Selway and Lochsa rivers, whereas *Acuclavella quattuor* occurs between the South Fork Clearwater and Salmon Rivers. This population was tested as a new species with the working name *Acuclavella* cf. *quattuor*.

### Cybertaxonomy

A cybertaxonomic approach was undertaken for enhanced dissemination of this work (e.g., Miller et al. 2010). This work is available as an open access PDF (doi: 10.3897/zookeys.311.2920). Molecular sequence data has been uploaded to GenBank (http://www.ncbi.nlm.nih.gov/Entrez; see Table 1 for accession numbers). Some species of *Acuclavella* are difficult to diagnose using morphological characters alone. An effort has been made to facilitate DNA barcoding by uploading COI sequences to the Barcode of Life Data Systems (BOLD) v. 2.5 (http://boldsystems.org). Aligned matrices and all phylogenetic trees have been deposited in the Dryad Digital Depository (doi: 10.5061/dryad.16737). Anatomical images included in this publication and many additional images have been deposited in MorphBank (http://www.morphbank.net/?id=822371). A Keyhole Markup Language file (KML) for interactive viewing of species distributions and collection information in Google Earth (http://earth.google.com) is available as a supplementary file (Appendix VI). All nomenclatural acts have been registered with ZooBank (http://www.zoobank.org). New species described in this study are provided to Encyclopedia of Life (http://www.eol.org) where interconnection of information is linked back to this open access, peer-reviewed work.

## Data resources

The data underpinning the analyses reported in this paper are deposited in the Dryad Data Repository at doi: 10.5061/dryad.16737.

## Results and discussion

### Taxon sampling

Fieldwork resulted in the collection of 272 *Acuclavella* specimens from 61 localities. Populations were sampled from throughout northern Idaho, as well as in the Cascade Mountain and Olympic Peninsula physiogeographic regions of western Washington (Figure 1). The first state records for Montana were secured from the Bitterroot Mountains. Specimens were collected from the four type localities (Shear 1986), including male specimens from the type locality of *Acuclavella quattuor*, which Shear originally described from a single female. The vast majority of specimens were collected from beneath moist woody debris near small perennial water features.

Although the overall geographic distribution of *Acuclavella* is now reasonably well-known, additional collecting efforts in the following areas may prove fruitful: south of known localities in Idaho, east into more inland areas of Montana, the Coast Range of Oregon, and the Willapa Hills of Washington. For example, most animal taxa that include populations in the Olympic Peninsula and Cascade Mountains are also found in the Willapa Hills (e.g. *Rhyacotriton*, Good and Wake 1992; *Octoglena anura*, Shelley et al. 2010).

### DNA sequence characteristics

Mitochondrial COI DNA sequences for a geographical subset of *Acuclavella* specimens were inconsistent with expectations of protein-coding gene evolution. TheseCOI sequence reads showed ambiguous nucleotides at individual sites, a large number of mutations resulting in replacement amino acid substitutions, as well as insertions and deletions. Some deletions resulted in frame-shift mutations resulting in stop codons. These patterns of variation suggest that nuclearized copies of the mitochondrial COI gene (NUMTs; White et al. 2008, Baldo et al. 2011) exist in *Acuclavella*.

Sequences showing evidence for nuclearization were discarded, resulting in the removal of all sequences for individuals collected north of the Middle Fork Clearwater River, including all *Acuclavella cosmetoides* and *Acuclavella shoshone* (*sensu stricto*). In addition to apparent authentic COI sequences, we have multiple nuclear markers providing a similar history for three new species, so our taxonomic conclusions should not be compromised. The final COI matrix included 18 ingroup sequences (L = 1224 basepairs; Table 1). The first, second, and third codon positions included 34, 9, and 282 parsimony-informative characters in the ingroup. GenBank numbers for all mitochondrial gene sequences can be found in Table 1.

**Table 1.** Phylogenetic taxon sample and GenBank accession numbers.<br/>

**Species / Voucher No.**	**COI**	**28S**	**EF-1** α	**Wnt2**
Acfquattuor_OP2230_DeVoto	KF181730	KF181744	KF181759	
Acfquattuor_OP2275_SplitCkTr	KF181731		KF181760	KF181779
Acfquattuor_OP2284_SelwayRvRd	KF181732		KF181761	KF181780
Acosmetoides_OP2281_2ShadowsCk		KF181745	KF181762	
Acosmetoides_OP2296_FS250		KF181746	KF181763	
Acosmetoides_OP2299_TribOrogrande				KF181781
Acosmetoides_OP2319_MeadowCk		KF181747		
Acosmetoides_OP2341_GooseCk		KF181748		
Aleonardi_OP2347_IronCk	GQ870648	KF181749	GQ872169	KF181782
Aleonardi_OP2349_NFGobleCk			KF181764	
Aleonardi_OP2712_KjesbuRd		KF181750	KF181765	
Aleonardi_OP2714_UpperIronCk	KF181728		KF181766	
Americkeli_OP2237_FS443	KF181733	KF181751	KF181767	
Americkeli_OP2245_FS443	KF181734			KF181783
Americkeli_OP2250_RedHorseCk	KF181735		KF181768	KF181784
Amakah_OP1699_RubyBeach	KF181736		KF181769	
Amakah_OP2345_CedarCk	GQ870647	KF181752	GQ872168	
Amakah_OP2715_HokoFalls	KF181737	KF181753	KF181770	KF181785
Amakah_OP2716_BrownesCk	KF181738		KF181771	
Amakah_OP2719_YahooLkRd			KF181772	
Aquattuor_OP2242_GrouseCk	KF181739		KF181773	KF181786
Aquattuor_OP2257_SlateCk	KF181740	KF181754	KF181774	
Aquattuor_OP2270_FS221	KF181729			KF181787
Asheari_OP2708_BurgdorfRd	KF181741	KF181755	KF181775	
Asheari_OP2709_BurgdorfRd	KF181742		KF181776	
Asheari_OP2720_FS592	KF181743	KF181756	KF181777	KF181788
Ashoshone_OP2316_EmeraldCkRd				KF181789
Ashoshone_OP2323_Hobo		KF181757	KF181778	
Ceratolasma	GQ912865	JX573543	AF240864	KF181790
Dendrolasma	KF181727	GQ912771	AF240865	
Hesperonemastoma	JX573642	JX573548	AF240869	KF181791
Ischyropsalis	JX573639	AF240870	JX573603	KF181792
Ortholasma	GQ912870	KF181758	GQ872161	
Sabacon	JX573670	JX573551	AF240877	KF181793
Taracus	JX573680	JX573592	AF240881	KF181794

Gblocks removal of ambiguous sites in the 28S matrix resulted in a reduction of alignment length from 1211 to 1173 positions. The aligned matrix included 26 parsimony informative characters for 14 ingroup sequences. For the EF-1α gene, eight of 22 *Acuclavella* sequences showed signs of heterozygosity. Three of these individuals were ambiguous at a single base pair, two were ambiguous at two sites, and remaining individuals were ambiguous at 3, 4, or 5 sites respectively. All haplotypes were reconstructed using PHASE. The resulting aligned matrix consisted of 690 bp of exon data, and an 80 bp intron with gaps. The EF-1α exon contained 30 parsimony informative characters in the ingroup. The intron sequences did not have outgroup representatives; there were 18 parsimony informative characters in the intron. The Wnt2 gene matrix (L = 370) included 7 ingroup sequences, with only 4 parsimony informative characters. GenBank numbers for all nuclear gene sequences can be found in Table 1.

### Phylogenetic analyses

Models of evolution inferred from jModelTest are summarized in Table 2. These partition-specific models were used in a Bayesian analysis of the concatenated matrix. Figure 2 shows results from the Bayesian analysis and associated posterior probabilities; the phylogeny resulting from the RAxML analysis is available in a supplementary file (Appendix VII). The concatenated Bayesian and ML phylograms are congruent in their strong support for a monophyletic *Acuclavella*. *Ceratolasma* is recovered as sister to *Acuclavella* with strong support, confirming the hypothesis of Shear (1986). Deeper phylogenetic relationships within Ischyropsalidoidea are beyond the scope of this research, and no emendations to the current taxonomy are proposed, pending collection of additional data.

**Table 2.** Models of DNA sequence evolution as determined by jModelTest v0.1.1 <br/>

**Gene**	**Model**
COI 1st partition	GTR+I+G
COI 2nd partition	GTR+I+G
COI 3rd partition	GTR+G
28S	GTR+I+G
EF-1α intron	GTR+G
EF-1α exon	SYM+G
Wnt2	SYM+I

Within *Acuclavella*, samples from Washington and Idaho are reciprocally monophyletic in both ML and Bayesian analyses, with regional clades strongly-supported and subtended by relatively long branches (Figure 2, Appendix VIII). Within Washington, both analyses recover samples from the Olympic Peninsula and southern Cascade Mountains as monophyletic lineages separated by long branches with high support. Both ML and Bayesian analyses also strongly support *Acuclavella sheari* as monophyletic and sister to all other Idaho samples. *Acuclavella* cf. *quattuor* was recovered by both analyses, but is not supported by either (PP = 0.52, BS = 13). No other molecular clades are recovered conforming to other described species of northern Idaho *Acuclavella*. A KML file has been created to more easily visualize where sequences used to construct the Bayesian phylogeny occur in geographic space (Appendix VI).

All Bayesian individual gene trees had their longest branch separating Washington and Idaho genetic groups (Figure 3). The COI, EF-1α, and 28S gene trees (Figure 3A, 3C, and 3D respectively) also show a deep split separating monophyletic *Acuclavella leonardi* and *Acuclavella makah* samples. *Acuclavella sheari* was also recovered in each of the individual trees, but with varied support and topological placement within a clade of Idaho samples. The COI gene tree shows significant support for a monophyletic *Acuclavella sheari*, which is sister to all remaining Idaho sequences. This species is recovered as monophyletic but without support by EF-1α and 28S gene trees. The Wnt2 gene tree (Figure 3B) supports reciprocal monophyly of samples from Washington and Idaho, but lacks phylogenetic signal within Idaho. The lack of structure for shallow evolutionary events within *Acuclavella*, coupled with high support for most nodes within Ischyropsalidoidea above the generic level, points to a deeper phylogenetic utility for Wnt2 in Opiliones.

### Morphometric analyses

DFA and PCA morphometric analyses of male and female data sets most frequently recovered *Acuclavella sheari*, followed by *Acuclavella leonardi* and *Acuclavella makah*. Undescribed morphologies from northern Idaho clustered with samples of *Acuclavella cosmetoides* and *Acuclavella shoshone* (*sensu stricto*) of Shear, 1986. *Acuclavella merickeli* was also frequently recovered in these analyses. The two populations with two pairs of scutal spines on scute areas I and II (*Acuclavella quattuor* and *Acuclavella* cf. *quattuor*) were frequently recovered as distinct from other hypothesized species, but were not recovered as morphometrically distinct from each other.

Since the group frequencies of hypothesized species varied greatly (Table 3), jackknifed classification of some data sets reduced the number of individuals used to define a group to just two or three individuals. An assumption is made here that variation seen within hypothesized species is normal in terms of representing the cluster. Male *Acuclavella* are robustly discriminated by DFA analysis, with nearly all species correctly classified with 100% accuracy. An exception is *Acuclavella* cf. *quattuor*, with two specimens classified as an *Acuclavella quattuor* (classification error rate of 0.09). Results of DFAs are available Results of DFAs are available in Appendix IV. The analysis was repeated with a jackknife classification resulting in successful discrimination of 94% of male individuals. Considering the high variation in group frequencies of hypothesized species (Table 3), analyses were rerun allowing prior probabilities of group membership. These analyses resulted in very similar groupings (not shown).

**Table 3.** Sample sizes for PCA and DFA analyses.<br/>

**Species**	**Males**	**Females**	**Total**
*Acuclavella makah*	10	14	24
*Acuclavella leonardi*	6	4	10
*Acuclavella sheari*	4	3	7
*Acuclavella quattuor*	14	17	28
*Acuclavella merickeli*	19	19	38
*Acuclavella* cf. *quattuor*	22	18	40
*Acuclavella cosmetoides*	56	58	114
**Total**	131	133	261

The number of individuals from each hypothesized species used in morphometric analyses.

Groupings based on female specimens were not recovered as frequently as male-based groups. Females of *Acuclavella leonardi*, *Acuclavella merickeli*, and *Acuclavella sheari* were correctly discriminated in the classification matrix. There was support for female samples of *Acuclavella makah* and *Acuclavella cosmetoides*, with correct classification 93% and 97% of the time respectively. About 25% of *Acuclavella quattuor* and *Acuclavella* cf. *quattuor* females were misclassified as the other species. Results from the jackknifed classification matrix show similar, if slightly lower percentages of correctly classified individuals. In this matrix, one *Acuclavella sheari* is misclassified as *Acuclavella cosmetoides*, and three *Acuclavella cosmetoides* are misclassified as *Acuclavella sheari*. This is likely the result of convergent similarities – females of *Acuclavella sheari* and some *Acuclavella cosmetoides* (*Acuclavella shoshone sensu stricto*) lack scutal spines.

Principle components analyses regularly recovered all hypothesized species of *Acuclavella* with the exception of *Acuclavella* cf. *quattuor* from *Acuclavella quattuor*. Figure 4 shows a graph recovering or nearly recovering these species. *Acuclavella sheari* was most frequently recovered in morphospace, with strong evidence for *Acuclavella leonardi* and *Acuclavella makah* as morphometrically distinct. Also well-supported, *Acuclavella merickeli* and *Acuclavella cosmetoides* were recovered in PCA analyses. *Acuclavella* cf. *quattuor* and *Acuclavella quattuor* were regularly recovered as distinct from other hypothesized species, but were not distinguishable from each other. Generally, analyses run on covariance matrices had more success of clustering hypothesized species than analyses run on a correlation matrix. Similar to DFAs, males tended to be recovered more frequently than females. Graphs plotting principle components from all PCA analyses conducted (n=143), as well as a description of strategies employed to explore morphospace, are available as a supplementary file (Appendix IV).

### Difficulties with previously described species

Both phylogenetic and morphometric analyses strongly support three new species: *Acuclavella leonardi*, *Acuclavella makah*, and *Acuclavella sheari*, but do not clearly support already recognized taxa within Idaho samples north of the Salmon River. Molecular and morphological data sets agree that phylogeographic structure associated with the Salmon, Selway, and Lochsa Rivers exists, and morphological data suggests differentiation across the South Fork Clearwater River. Although the number of individuals and known localities was greatly improved by this research, further specimen and genetic sampling is needed to adequately test species limits in northern Idaho. In particular, more rapidly-evolving genetic markers are needed for resolution of what is likely a relatively recent evolutionary radiation in this region. A lack of phylogenetic structure within most of northern Idaho, coupled with the finding that some of the undescribed morphologies cluster together with *Acuclavella shoshone* and *Acuclavella cosmetoides*, make it possible that *Acuclavella cosmetoides* is a widespread and morphologically highly variable species that encompasses *Acuclavella shoshone*. The same hypothesis may apply to the *Acuclavella merickeli* plus *Acuclavella quattuor* lineage.

Though they lack molecular support, *Acuclavella merickeli* and *Acuclavella quattuor* are readily discriminated by morphometric analyses (Figure 4). Since male *Acuclavella quattuor* were collected from the type locality and agree with the diagnostics for females (Shear 1986), the male for this species is here described and figured for the first time.

### Biogeography

The Western Hemlock Zone (WHZ) (Franklin and Dyrness 1988) of northern Idaho is disjunct from its coastal counterpart. This disjunction is explained by the orogeny of the Cascade Mountains 8-5 MYA (Mitchell and Montgomery 2006), and subsequent xerification of the Columbia Basin due to the rain shadow formed from this uplift (Leopold and Denton 1987). Since this isolation, the WHZ in Idaho has been subject to the numerous glacial oscillations including alpine glaciers and continental ice sheets (Gates 1983). During peak glaciation it is believed that organisms of the WHZ were forced into compartmented refugia, either in valleys or south of glaciated regions (Brunsfeld and Sullivan 2006, Nielson et al. 2006).

Reconstructed molecular phylogenies show that *Acuclavella* biogeography is largely congruent with that seen in other dispersal-limited taxa of the Pacific Northwest. The deep phylogenetic split between Washington and Idaho (Figures 2, 3) conforms to the ancient vicariance hypothesis (Brunsfeld et al. 2001) seen in amphibians including *Ascaphus* (Nielson et al. 2001), *Dicamptodon* (Steele et al. 2005), and *Plethodon* (Carstens et al. 2004). Relatively long branches separate *Acuclavella* species from the Cascade Mountain and Olympic Peninsula ecoregions of western Washington, which is congruent with patterns seen in *Rhyacotriton* salamanders (Good and Wake 1992).

### Natural history

*Acuclavella* are riparian obligate forest-dwellers. The vast majority of specimens were collected within the channel wall of perennial headwater streams or in seep-like features. Most specimens were found underneath large woody debris, though excavation of woody sediment wedges (May and Gresswell 2003) or mats of moss adjacent to these streams also resulted in securing individuals. Coniferous canopy cover is typically consistent across these water features at northern Idaho localities. The dominant vegetation at Idaho sites included a canopy of *Tsuga heterophylla* and *Thuja plicata*, or *Abies grandis* in southern areas. Washington localities typically had a riparian vegetation corridor along small streams dominated by *Alnus rubra* and *Rubus spectabilis* (Appendix I).

The primary defense of *Acuclavella* appears to be one of crypsis. Their dark bodies are cryptic against moist woody debris, onto which specimens adhere with outstretched legs. When detected, specimens are easily collected by grabbing onto an outstretched leg; a hurried scramble is soon followed by limb rigidity. Autospasy was not encountered. A strategy to overcome the crypsis of *Acuclavella* is to expose appropriate areas and wait a few minutes for animals to come out of thanatosis to scurry for cover and appropriate microclimatic conditions. *Acuclavella* likely employ mechanical defense via spination. When turning a cover object the first author placed the pad of his finger directly on an individual; it felt like being pricked by a rose. It is likely that heavy sclerotization of the integument and the ubiquitous hemispherical warts add to the structural integrity of these spines. Though circumstantial, this proposal of heavy sclerotization for mechanical defense is consistent with recent findings in other harvestmen (Souza and Willemart 2011).

*Acuclavella* appear to have an annual life cycle, with seasonal adults. Penultimate stage subadult and adult animals have been collected in April and May; additionally, a young instar was collected in mid October (CHR3435; same locality as CHR 1409; Appendix I). Adults have been found from May to September. Mating was not encountered, nor was feeding. A single individual (CHR2403) (Appendix I) was found dead in the web of a *Pimoa* spider (CHR2404).

### Poor-person’s rainforest

Recent surveys of litter dwelling animals in mesic forests of the Pacific Northwest have led to the discovery of many new species. Taxa include terrestrial gastropods (Leonard et al. 2011, Leonard et al. 2003), millipeds (Shear and Leonard 2003, Shelley and Shear 2006, Shear and Leonard 2007, Shelley et al. 2010), pseudoscorpions (Mark Harvey personal communication), and Opiliones (this work, additional undescribed species). An appreciation of these creatures benefits society in numerous ways. Taxonomic knowledge provides a better understanding of forest ecosystems, by identifying potential nutrient recyclers and vertebrate prey items. Taxonomic knowledge also provides future researchers with a baseline from which to compare trends – in the words of Joseph Grinnell (1910) “this value will not, however, be realized until the lapse of many years, possibly a century…”. There is promise of novel chemical discovery in the repugnant secretions of harvestmen and millipeds. To some they embellish the aesthetic beauty of our forests. Though many of these newly described organisms are widespread, and without conservation concern, this is likely not true for all taxa (e.g. Shear and Leonard 2004). It is important that surveys continue in this geographic region and for litter faunas in particular. Litter dwellers are ideal organisms for establishing or adding to a metric of biodiversity, and it is important that true microendemics are identified; this information greatly informs conservation efforts (Harvey et al. 2011).

## Systematics

The genus *Acuclavella*, and species *Acuclavella merickeli*, *Acuclavella cosmetoides*, *Acuclavella shoshone*, and female *Acuclavella quattuor* are described by Shear (1986); where descriptive information follows those entries, it should be considered an addendum to his descriptions. Depository abbreviations: American Museum of Natural History (AMNH), California Academy of Sciences (CAS), University of Washington Burke Museum (UWBM), personal collection of primary author (CHR).

### Genus Acuclavella

Shear, 1986

http://species-id.net/wiki/Acuclavella

Acuclavella Shear, 1986: 13. Type species *Acuclavella cosmetoides* Shear, 1986.

#### Description.

The simple, distally tapering penis (Appendix VIII, Figure I) and short ovipositor (Appendix III, Figure 2) morphologies are conserved across species, with intraspecific variation seemingly as great as interspecific variation. Penis sheath with two sclerotized bands. Metatarsus of leg II with or without false leg articulations (Appendix IX). Distitarsi with three segments on legs I and II; distal end of legs III and IV with two constrictions, each comprised of two segments. All males with raised, glandular (Shear 1986), setose mound dorsally on basal article of chelicerae, setae often capped with secretions (MorphBank image 822802); mounds lacking in females. Chela teeth diaphanous, with two heavily sclerotized teeth distally on fingers (MorphBank image 822817). All leg coxae with prolateral and retrolateral tubercles in the form of distended clusters of warts (MorphBank image 828524). Epistome of stomotheca horn-like, projecting outward (MorphBank image 828520) to strongly decurved (MorphBank image 82215), without apparent interspecific trends. Pseudotrachea of pedipalpal coxaphysis sclerotized ctenoid (MorphBank image 822815).

#### Key to the species of *Acuclavella*.

This dichotomous key should allow users to identify the new species described herein. However, discovery of new morphologies in northern Idaho not described by Shear (1986), and not ascribed to species herein, are currently of uncertain placement at the species level. These morphologies include females with pairs of spines on scute areas I-IV, individuals with three pairs of spines on scute areas I-III, and individuals with pairs of spines on scute areas I-II which are geographically and genetically distinct, but morphologically similar to *Acuclavella quattuor*. For the purposes of this work, the four species named by Shear (1986) are treated *sensu stricto*, and follow his diagnoses.

**Table:** 

1a	Setose mounds dorsally on surface of cheliceral article I; males (Fig. 6)	2
1b	Without raised mounds on chelicerae; females	8
2a (1a)	Paramedian tubercles acute spines on scute area II only (Fig. 9)	3
2b	Paramedian tubercles acute spines on multiple scute areas (Fig. 11)	6
3a (2a)	Ocularium height (ventral edge of eye to tip of ocularium) ≤ 0.60 mm; area II spine height ≤ 0.50 mm from surface of tergite	*Acuclavella sheari* sp. n.
3b	Ocularium and area II spine heights ≥ 0.80 mm	4
4a (3b)	Distal ends of leg femora, patellae, and tibiae distinctly light, contrasting as light joints; palpi white or nearly so; with or without false leg articulations on leg II metatarsi (Appendix IX); with or without distal, dark, prolateral tubercle on palpal patellae; western Washington	5
4b	Distal ends of leg segments not light; palpi light brown or dark; without false leg articulations and palpal tubercles; Idaho	*Acuclavella merickeli* Shear, 1986
5a (4a)	Leg II femur ≤ 3.76 mm; Cascade Mountains	*Acuclavella leonardi* sp. n.
5b	Leg II femur ≥ 3.76 mm; Olympic Peninsula	*Acuclavella makah* sp. n.
6a (2b)	Paramedian tubercles raised into acute spines on scute areas I and II only (Fig. 11); known distribution bracketed by the Salmon River and South Fork Clearwater River	*Acuclavella quattuor* Shear, 1986
6b	Paramedian tubercles with acute spines on three or more scute areas; scute area III always with such tubercles (Appendix V, Figure 1)	7
7a (6b)	Paramedian tubercles not raised into spines on scute area I, paired spines on areas II, III, and IV	*Acuclavella cosmetoides* Shear, 1986
7b	Paramedian tubercles raised into acute spines on scute areas I – IV (four pairs of abdominal spines	*Acuclavella shoshone* (Shear, 1986)
8a (1b)	Paramedian tubercles acute spines on scute area II only (Fig. 9)	9
8b	Tubercles not as previous; if spines on area II only, greatly reduced	11
9a (8a)	As couplet 4a	10
9b	As couplet 4b	*Acuclavella merickeli*
10a (9a)	Known only from the Cascade Mountains of Washington State; no perceived discriminating features	*Acuclavella leonardi* sp. n.
10b	Known only from the Olympic Peninsula of Washington State; no perceived discriminating features	*Acuclavella makah* sp. n.
11a (8b)	As couplet 6a	*Acuclavella quattuor* Shear, 1986
11b	Paramedian tubercles not as previous	12
12a (11a)	As couplet 7a	*Acuclavella cosmetoides* Shear, 1986
12b	Paramedian tubercles not enlarged into spines, but paired, low, rounded tubercles on all scutal areas (Fig. 9)	13
13a (11b)	Palpal femur ≤ 0.88 mm; leg II tarsus ≤ 3.92 mm; leg II femur ≤ 2.75 mm; currently known south of Salmon River	*Acuclavella sheari* sp. n.
13b	Palpal femur ≥ 0.90 mm; leg II tarsus ≥ 3.95 mm; leg II femur ≥ 2.72 mm, north of Middle Fork Clearwater River	*Acuclavella shoshone* (Shear, 1986)

### Acuclavella leonardi sp. n.

urn:lsid:zoobank.org:act:3F79C21E-E37A-4FC5-92E3-170CA8D9481C

http://species-id.net/wiki/Acuclavella_leonardi

MorphBank images of specimens considered this species include:

Paratype AMNH, MorphBank Specimen Id: 822643, 1 image

Paratype CASENT9039218, MorphBank Specimen Id: 822508, 4 images

Paratype CASENT9039224, MorphBank Specimen Id: 822516, 2 images

SDSU OP2347, MorphBank Specimen Id: 822509, 1 image

SDSU OP2349, MorphBank Specimen Id: 822511, 3 images

SDSU OP2712, MorphBank Specimen Id: 822515, 1 image

SDSU OP2714, MorphBank Specimen Id: 822517, 3 images

**Figure 5.**
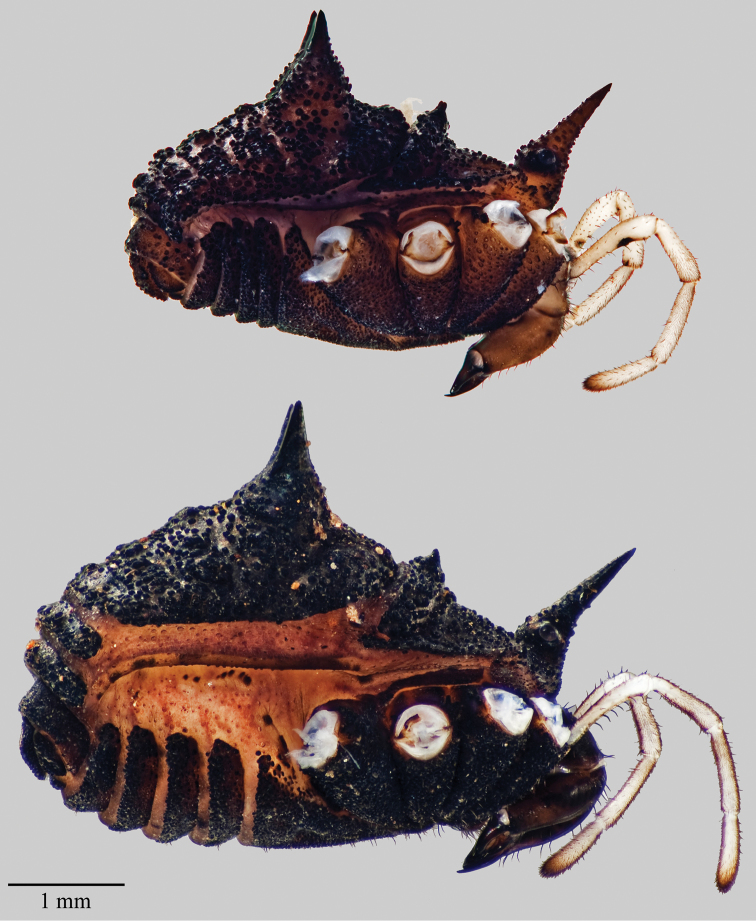
*Acuclavella leonardi*. Top: male, CHR3325; bottom: female, CHR 2492.

**Figure 6.**
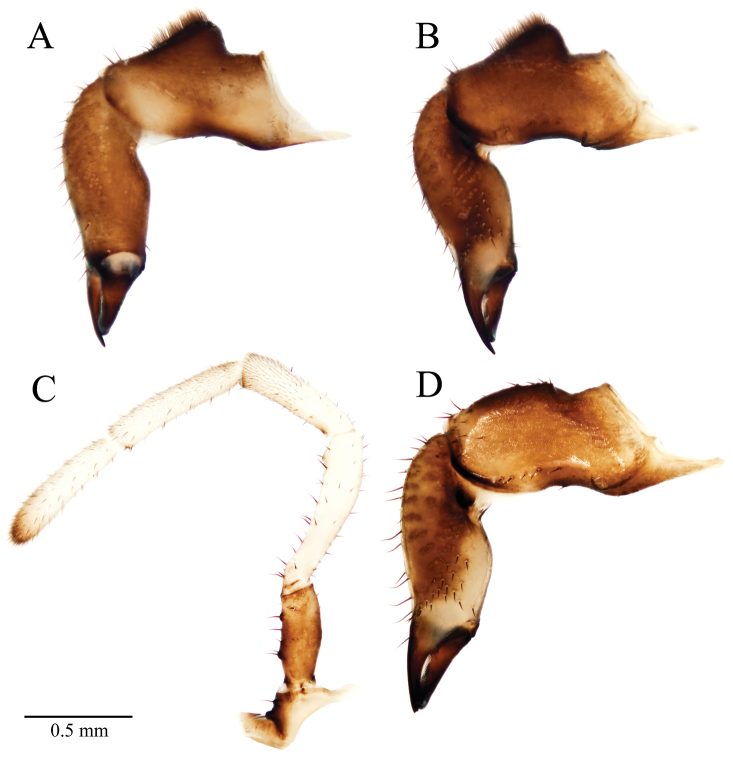
*Acuclavella leonardi* chelicerae and pedipalp. **A** right chelicera, retrolateral view **B** left chelicera, prolateral view **C** right pedipalp, retrolateral view (A–C, male CASENT9039218) **D** left chelicera, retrolateral view (female CASENT9039224).

Figure 5 and 6 , Appendix VIII: Figure 1, Figure 2

Acuclavella merickeli Shear, 1986 (in part: specimen from Pg 21-22)

#### Type material.

Male **holotype** (AMNH), and male (CAS, CASENT9039218) and female (AMNH) **paratypes** from a tributary of Iron Creek, Forest Service Rd 25 4.6 miles south of FS Rd 300, Gifford Pinchot National Forest, Lewis County, Washington; male **paratype** (UWBM, WA2392/6319) and female **paratype** (CAS, CASENT9039224) from upper Iron Creek, FS Rd 28 0.1 miles E of FS 25, Gifford Pinchot National Forest, Skamania County, Washington; female **paratype** (UWBM, WA2391/6027) from a tributary of Goble Creek, Cowlitz County, Washington. Further information on type localities can be found in Appendix I with the exception of female paratype from Goble Creek. This specimen was collected by the first author 3 August 2005 and deposited in a research collection; this specimen was not used in morphometric analyses, but is used to characterize the species in the description below. This specimen was collected at a location accessed via S Goble Creek Rd; 2.0 miles east of Rose Valley Rd turn right, 1.6 miles turn right, 0.6 miles park; 46.0963°N, 122.7607°W, elevation 227 meters.

#### Etymology.

The specific epithet is a patronym in honor of the naturalist and careful observer William P. Leonard for his work on litter-dwelling organisms in the poor-person’s rainforest of the Pacific Northwest.

#### Diagnosis.

Distinguished from all *Acuclavella* except *Acuclavella makah* by the combination of having paramedian tubercles as enlarged spines on area II only, and having light, strongly contrasting ends to sclerotized leg segments, giving the appearance of banding. Also distinguished from these taxa in that false leg articulations on the metatarsi of legs II are present, or single dark prolateral tubercles on the palpal patellae are present, but these features are not consistently found in *Acuclavella leonardi*. Scutes posterior to spines containing many raised mounds bearing warty tubercles, more distinct than in *Acuclavella makah*. Though the height of scutal spines is similar, the base of the spines in *Acuclavella leonardi* appears broader than in *Acuclavella makah*. Diagnostic COI sequences have been uploaded to the Barcode of Life Data Systems (BOLD: ACUOP005-13).

#### Description.

**Description of male.** Body arched and convex dorsally (Figure 5); sides parallel or nearly so when broader posteriorly. Nearly all of body heavily sclerotized black or brown, with densely scattered hemispherical warts which irregularly house short setae apically or posteriorly. Total length 4.18 mm (*n*=3, 3.88–4.35 mm), carapace length 1.22 mm (*n*=7, 1.10–1.35 mm), carapace width 2.53 mm (*n*=3, 2.44–2.65 mm), length of fused tergites I-V 2.24 mm (*n*=7, 2.10–2.41 mm), scutum length 2.73 mm (*n*=3, 2.65–2.85 mm), scutum width 2.63 mm (*n*=3, 2.55–2.75 mm).

Eye tubercle at anterior edge of carapace prolonged into an acute spine lacking hemispherical warts on distal half; standing 1.25 mm above the surface of the carapace (*n*=3, 1.13–1.35 mm), 1.05 mm (*n*=7, 0.88–1.19 mm) from the ventral edge of the eye to the tip of the tubercle. Eye color brown, brown-gray, or gray, located basally on tubercle. Metapeltidial paramedian sensory cones raised into a sharp, acute spine standing 0.26 mm (*n*=7, 0.23–0.28 mm) above surface of the carapace, curving slightly towards the midline; lateral to these spines clusters of warts form tubercles.

All scutal tergites with pairs of median tubercles, these prolonged into large spines on area II only; lateral tubercles distinct. Tergite I with paired median tubercles as raised mounds adorned with warts standing 0.09 mm (*n*=7, 0.08–0.13 mm) above the scutal surface; two additional pairs of warty mounds reduce in size laterally. Median tubercles on area II tergite greatly enlarged into erect spines standing 1.04 mm (*n*=7, 0.85–1.16 mm) above the surface of the scutum; lateral to these tubercles are raised mounds adorned with warts. Area III tergite with three pairs of relatively large tubercles in the form of raised mounds adorned with warts; these not decreasing in size laterally; apical setae on each mound; median pair 0.06 mm above scutum (*n*=7, 0.03–0.08 mm). Tergite areas IV and V with three or four (UWBM) pairs of tubercles in the form of raised mounds adorned with warts, these not decreasing in size laterally or posteriorly; setae as previous; area IV median tubercle height 0.06 mm (*n*=7, 0.05–0.08 mm). First free tergite (VI) with relatively large and numerous tubercles in the form of raised warty mounds. Second free tergite (VII) as previous in the holotype and paratype (CAS); paratype (UWBM) without tubercles. Free tergite VIII without distinguishable tubercles, or with a median pair only (CAS paratype).

Abdominal sternite warty sculpturing strongest laterally and on posterior margins; sternites brown. Sclerotized areas of genital operculum relatively setose. Prosomal sternum (*n*=1) length 0.19 mm, width 0.37 mm; brown; without setae. Labium weakly to moderately sclerotized, wider than long or longer than wide (CAS); lengths: 0.14, 0.20, 0.08 mm, widths: 0.28, 0.16, 0.18 mm; light brown to brown; without setae. Palpal endites setose, light brown or brown. Leg II endites bearing 1 or 2 (UWBM) setae, leg IV bearing 1 setae in paratype only, all other endites without setae. Horn-shaped process of epistome decurved, projecting 0.40 mm from sulcus (*n*=1).

Chelicerae brown or light brown (Figure 6); darker dorsally; article II with prolateral and retrolateral striations of darker, more sclerotized cuticle; weakly so in paratypes. Cheliceral measurements (*n*=3): article I length 1.14 mm (1.03–1.34 mm), width 0.41 mm (0.40–0.44 mm), article II length 1.32 mm (1.20–1.38 mm), width 0.38 mm (0.35–0.38 mm), article III length 0.56 mm (0.53–0.60 mm). Dorsal surface of article I with raised, glandular area dense with setae, these capped with a white secretion in the CAS paratype; proximal end of article I with boss-like tubercles on retrolateral and proventrolateral surfaces. Palpal coxae brown or light brown (UWBM paratype), with 2 seta-bearing tubercles. Palpal measurements given in Table 4. Trochanter (Figure 6) brown, light brown, or pale brown, with 3 or 4 (UWBM paratype) seta-bearing tubercles. Palpal femora brown or white; patella white without a dark diffuse band, with dark, prolateral tubercles distally, bearing small trichia and setae distally; tibia white, with scattered setae and dense microtrichia; tarsus white, darkening distally, with vestiture of microtrichia and setae. Claw rudiment very small.

**Table 4.** *Acuclavella leonardi* palpus and leg measurements.<br/>

**Appendage**	**Segment**	**Male**			**Female**		
		**mean**	**Range**	***n***	**Mean**	**Range**	***n***
Palpus	trochanter	0.55	0.52–0.58	3	0.61	0.58–0.65	3
	femur	0.94	0.84–1.00	7	0.94	0.90–0.98	5
	patella	0.67	0.66–0.68	3	0.71	0.70–0.73	3
	tibia	0.74	0.73–0.74	3	0.73	0.67–0.80	3
	tarsus	0.69	0.67–0.72	3	0.66	0.64–0.68	3
Leg I	trochanter	0.49	0.48–0.52	3	0.50	0.45–0.56	3
	femur	2.37	2.28–2.44	3	2.25	1.96–2.60	3
	patella	0.88	0.88	3	0.87	0.84–0.92	3
	tibia	1.60	1.48–1.72	3	1.46	1.44–1.50	3
	metatarsus	2.17	1.84–2.36	3	2.19	2.04–2.28	3
	tarsus	2.89	2.72–3.08	3	2.80	2.68–2.88	3
Leg II	trochanter	0.57	0.52–0.60	7	0.55	0.52–0.56	5
	femur	3.53	3.32–3.76	7	3.46	2.88–3.92	5
	patella	1.02	0.96–1.08	7	1.00	0.92–1.08	5
	tibia	2.38	2.16–2.60	7	2.36	2.12–2.60	5
	metatarsus	3.70	3.44–4.05	7	3.69	3.28–3.96	5
	tarsus	4.93	4.74–5.19	7	4.58	4.40–4.70	5
Leg III	trochanter	0.48	0.48	3	0.54	0.52–0.60	3
	femur	2.23	2.12–2.28	3	2.10	1.88–2.25	3
	patella	0.85	0.80–0.88	3	0.84	0.80–0.88	3
	tibia	1.57	1.56–1.60	3	1.50	1.48–1.52	3
	metatarsus	2.49	2.40–2.56	3	2.38	2.24–2.45	3
	tarsus	3.13	3.08–3.20	3	2.95	2.80–3.05	3
Leg IV	trochanter	0.57	0.52–0.64	3	0.58	0.55–0.64	3
	femur	3.23	3.08–3.32	3	3.09	2.76–3.35	3
	patella	1.03	0.96–1.08	3	0.99	0.96–1.00	3
	tibia	2.01	1.96–2.08	3	1.99	1.96–2.05	3
	metatarsus	4.03	3.88–4.12	3	3.85	3.64–4.00	3
	tarsus	4.09	4.08–4.12	3	3.87	3.76–4.99	3

All measurements in millimeters; *n*= sample size.

Leg measurements given in Table 4. Microsculpture of femora, patellae, and tibiae scattered, distally elevated scales, bilobed scales not observed; scales subtend setae, occasionally housing seta apically. Leg trochanters, femora, patellae, tibiae light brown, dark brown, or black, lighter at joints; metatarsi of leg III with proximal one-third to one-half light brown, brown, or black; leg IV with proximal three-quarters light brown, brown, or black; proximal end of metatarsi of legs I and II pale brown, brown, or black; remaining metatarsal areas pale brown; tarsi pale brown, darkening distally. Scaled microsculpture subequal to darkened areas, remainder with setae and microtrichia. Metatarsi of leg II with false leg articulations (*n*=7).

Penis length 2.48 mm (*n*=1), glans plate 0.37 mm, stylus 0.09 mm, stylus slightly twisted, not decurved.

**Description of female.** Similar to male for nearly all characters. Total length 4.71 mm (*n*=3, 4.45–5.06 mm); carapace length 1.35 mm (*n*=5, 1.25–1.44 mm); carapace width 3.01 mm (*n*=3, 2.81–3.13 mm); scutum length 3.63 mm (*n*=3, 3.19–4.30 mm); scutum width 3.70 mm (*n*=3, 3.12–3.50 mm); length of fused sternites I-V 2.96 mm (*n*=5, 2.81–3.06 mm).

Eye tubercle height above surface of carapace 1.18 mm (*n*=3, 1.00–1.28 mm); distance from ventral edge of eye to tip of spine 1.06 mm (*n*=5, 0.90–1.13 mm). Eye color dark brown, pink-gray, or light brown. Metapeltidial spine 0.22 mm (*n*=5, 0.18–0.25 mm).

Paramedian tubercles or tergite I height 0.08 mm (*n*=5, 0.05–0.13 mm) above surface of tergite; median tubercles raised mounds adorned with warts, two additional pairs of warty mounds reducing in size laterally. Tergite II paramedian tubercles greatly enlarged into erect spines standing 0.94 mm (*n*=5, 0.78–1.03 mm) above tergite surface; lateral to these are raised mounds adorned with warts. Paramedian tubercles of tergite III 0.07 mm (*n*=5, 0.03–0.10 mm); IV 0.06 mm (*n*=5, 0.03–0.10 mm). Tergite areas III, IV, and V with three pairs of relatively large tubercles in the form of raised mounds adorned with warts; these not decreasing in size laterally, decreasing slightly posteriorly across tergites. First free tergite (VI) adorned with raised warty mounds; tergites VII and VIII without discernable tubercles in paratypes (AMNH, CAS), tergite VII of paratype (UWBM) with tubercles as in VI.

Sternites brown. Transverse furrow and membranous lateral sutures of genital operculum less distinct than in other species. Prosomal sternum (*n*=1) length 0.18 mm, width 0.39 mm; pale-brown; without setae. Labium wider than long or subequal; lengths: 0.10, 0.19, 0.13 mm; widths: 0.16, 0.18, 0.19 mm; moderately or weakly sclerotized; brown; without setae. Palpal endites light brown. Leg II endites adorned with 2 setae.

Horn-shaped process of epistome decurved, projecting 0.42 mm from sulcus. Chelicerae brown or light brown; article I length 1.18 mm (*n*=3, 1.16–1.21 mm), width 0.43 mm (0.42–0.44 mm); article II length 1.39 mm (1.34–1.42 mm), width 0.41 mm (0.38–0.43 mm); article III length 0.59 mm (0.58–0.62 mm). Article I without raised glandular mound (Figure 6). Article II with 6 setae on prolateral dark area at cleavage of corpus and fixed finger of chela; 15 setae on ventral surface of article II; these patches discrete. Palpus dimensions in Table 4; coxae light brown or brown with two seta-bearing tubercles; trochanters brown or pale brown with 4 or 5 seta-bearing tubercles; only paratype (CAS) with tubercle on patella, only paratype (UWBM) with partial diffuse band on patella.

Leg measurements given in Table 4. Leg trochanters, femora, patellae, and tibiae brown to dark brown, lighter at joints; metatarsi of legs III with proximal one-half brown, of leg IV with proximal three-quarters light brown to brown, proximal ends of legs I and II light brown to brown, remaining metatarsal areas pale brown; tarsi pale brown, darkening distally.

Ovipositor length 0.80 mm, width 0.44 mm; corona of setae at furcal base surrounding lobes, apical setae on lobes; furca without dorsoventral differentiation.

#### Distribution and habitat.

*Acuclavella leonardi* is found in the southern Cascade Mountains of Washington State in the Cowlitz River (includes Iron Creek) and Coweeman River (includes Goble Creek) watersheds in Lewis, Cowlitz, and Skamania Counties (Appendix I). Found in coniferous forests with small perennial water-features such as side-slope seeps, springs, and headwater streams; underneath stream-side woody debris.

### Acuclavella makah sp. n.

urn:lsid:zoobank.org:act:8C34D2F8-D211-42B7-B58A-A90285771589

http://species-id.net/wiki/Acuclavella_makah

MorphBank images of specimens considered this species include:

Holotype AMNH, MorphBank Specimen Id: 822644, 2 images

Paratype CASENT9039219, MorphBank Specimen Id: 822645, 3 images

CHR1536, MorphBank Specimen Id: 828519, 2 images

SDSU OP1699, MorphBank Specimen Id: 828516, 1 image

CHR2457.0, MorphBank Specimen Id: 822505, 4 images

CHR2457.1, MorphBank Specimen Id: 822506, 3 images

CHR2457.2, MorphBank Specimen Id: 822507, 3 images

CHR3387.0., MorphBank Specimen Id: 822647, 1 image

CHR3387.1, MorphBank Specimen Id: 822646, 1 image

**Figure 7.**
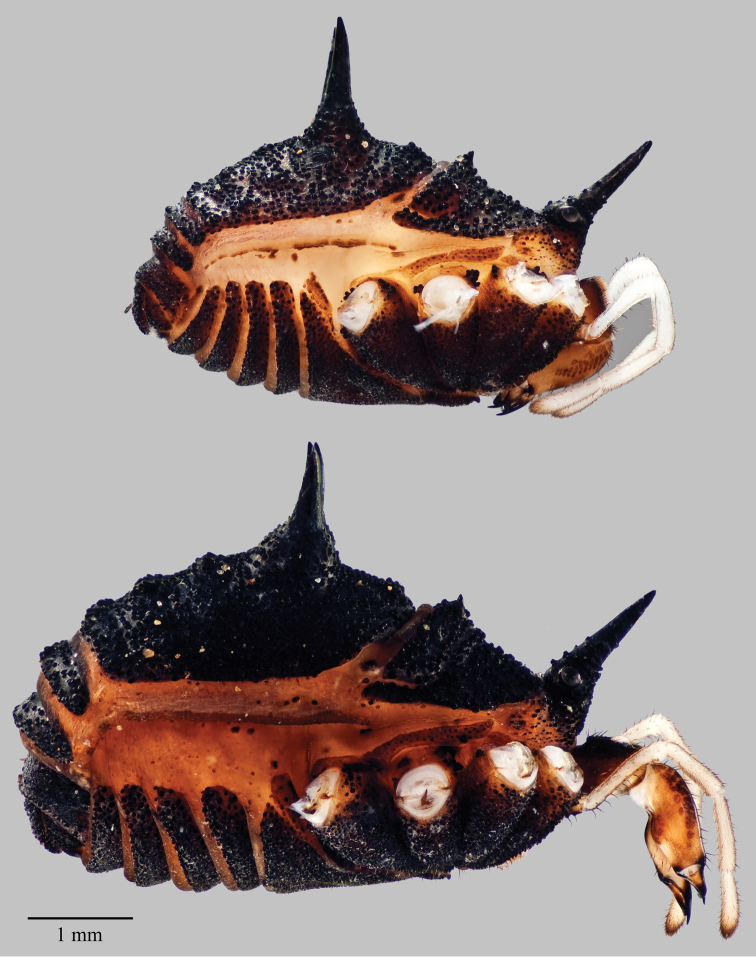
*Acuclavella makah*. Top: male, CHR2457.1; bottom: female, CASENT9039219.

**Figure 8.**
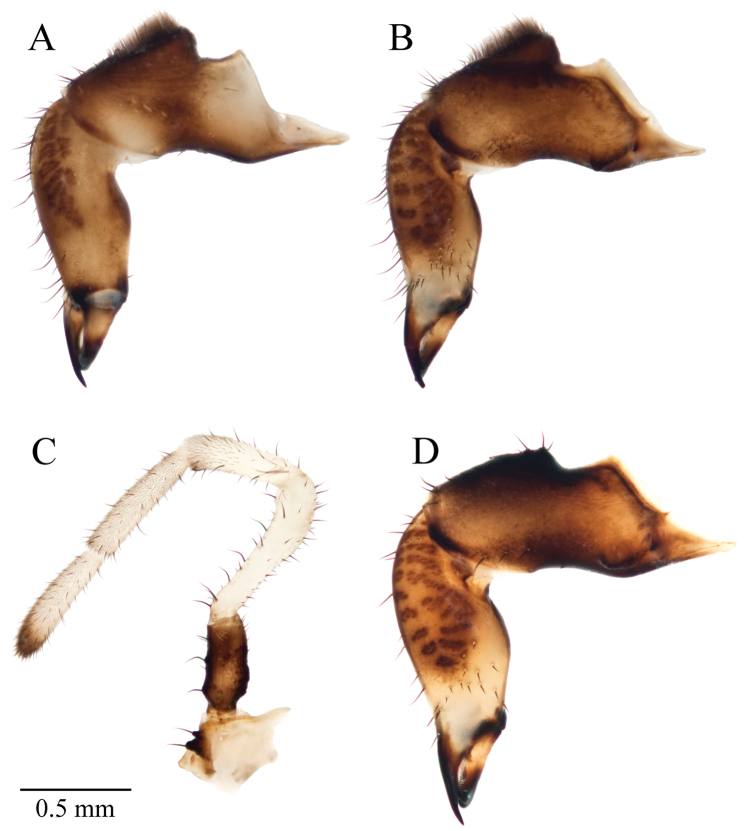
*Acuclavella makah* chelicerae and pedipalp. **A** right chelicera, retrolateral view **B** left chelicera, prolateral view **C** right pedipalp, retrolateral view (A–C, male CHR2457-0) **D** left chelicera, retrolateral view (female CHR2457-2).

Figure 7 and 8 , Appendix VIII: Figure 1, Figure 2 

#### Type material.

Male **holotype** and female **paratype** (AMNH), and male and female **paratypes** (CAS, CASENT9039219) from Brownes Creek, Clallam County, Washington; male and female **paratypes** (UWBM, WA2393/7641) from Yahoo Lake Road 1.4 miles east of Hoh-Clearwater Road, Jefferson County, Washington (Appendix I). An additional specimen is housed at UWBM (WA0973/8147): Ahlstroms Prairie, Olympic National Park, Clallam County, Washington, (NAD 1927) 48.157°N, 124.704°W, elevation 40 meters; Rod Crawford (collected under permit), 16–17 July 1984.

#### Etymology.

The specific epithet refers to the Makah Nation, which historically occupied much of the known distribution of the species. The name Makah was given to these people by their neighbors; it means “generous with food”. These people have shared with many people access to their beautiful land, next to the rocks and gulls. For more information on the Makah Nation see: http://www.makah.com.

#### Diagnosis.

Distinguished from all *Acuclavella* except *Acuclavella leonardi* by the combination of having paramedian tubercles on area II only, having light, and strongly contrasting ends to sclerotized leg segments, giving the appearance of banding at joints. Though not always present, false leg articulations on the metatarsi of legs II, and single dark prolateral tubercles on the palpal patellae also diagnose it from these species. Scutal tubercles lateral to paramedian tubercles tend to be on more distinctive raised mounds in *Acuclavella leonardi*. Area II spines more narrow at base than in *Acuclavella leonardi*. Best diagnosed from *Acuclavella leonardi* using molecular data. Diagnostic COI sequences have been uploaded to the Barcode of Life Data Systems (BOLD: ACUOP007-13).

#### Description.

**Description of male.** Body arched and convex dorsally (Figure 7), sides parallel with equal scutum and carapace widths; nearly all of body heavily sclerotized, black, with densely scattered hemispherical warts which irregularly house short setae apically or posteriorly. Total length 4.11 mm (*n*=3; 4.05–4.20 mm), carapace length in midline 1.26 mm (*n*=10; 1.05–1.44 mm), greatest carapace width 2.57 mm (*n*=3; 2.50–2.72 mm); length of fused scutes I-V in midline 2.33 (*n*=10; 2.20–2.48 mm), scutum length in midline 2.74 (*n*=3; 2.60–2.90 mm) greatest scutum width 2.57 mm (*n*=3; 2.50–2.72 mm).

Eye tubercle at anterior edge of carapace, prolonged anteriad into a sharp conical spine 1.27 mm (*n*=10; 1.05–1.45 mm) from ventral edge of eye to tip of spine; 1.58 mm above the carapace (*n*=3; 1.53–1.64 mm). Eyes light-brown to brown, located basally on tubercle. Surface of carapace evenly curved, posterior margin arcuate. Metapeltidial paramedian sensory cones short, acute spines standing 0.19 mm (*n*=10; 0.13–0.25 mm) above the surface of the metapeltidium, shiny, lacking warts.

All scutal areas with pairs of paramedian tubercles. Area I paramedian tubercles cluster of cuticular warts standing 0.04 mm above the surrounding scute (*n*=10; 0.025–0.075 mm), two additional pairs of warty tubercles reduce in size laterally. Paramedian tubercles of area II rise to form large acute spines standing 1.37 mm above the scutum surface (*n*=10; 1.13–1.75 mm); lateral to spines a pair of tubercles typically small cluster of warts, though one individual (CHR1536) with lateral pair of short spines. Scute areas III, IV, and V with three pairs of wart-clustered tubercles each; paramedian pair largest, diminishing in size laterally along scute, and posteriorly across scute areas. Paramedian tubercles of area III stand 0.03 mm (*n*=10, 0.03–0.05 mm) above surface of the tergite; area IV tubercle height 0.04 mm (*n*=10, 0.03–0.08 mm). Holotype and paratype (CAS) with first free tergite (area VI) with barely distinguishable tubercle as median pair of enlarged warts, tergites VII and VIII without tubercles; paratype (UWBM) tergite VI with median and lateral pair of enlarged warty tubercles, tergite VII with barely distinguishable tubercles, these lacking on tergite VIII. Tergite IX divided, triangular, bracketing tergite X, which forms the anal operculum.

Abdominal sternites with infrequent setae; warty sculpturing strongest on posterior and lateral margins; brown to dark brown. Prosomal sternum length 0.14 mm (*n*=2, 0.13–0.16 mm), width 0.28 mm (0.26–0.30 mm); brown; without setae. Labium weakly sclerotized, wider than long, length 0.08 mm (*n*=3; 0.05–0.11 mm), width 0.15 mm (0.13–0.17 mm); light brown to yellow-brown, without setae. Palpal endites brown to yellow-brown, large, free, bearing many setae; leg II endite bearing 3 setae in holotype and paratype (CAS), 2 in paratype (UWBM); legs I, III, IV endites without setae. Epistome with horn-like anteriad projection, decurved or slightly decurved, projecting 0.34 mm (*n*=2, 0.33–0.36 mm) from sulcus. Chelicerae basal article (I) dark brown dorsally, middle article (II) with prolateral and retrolateral striations of darker, more sclerotized striations. Article I length 1.18 mm (*n*=3, 1.06–1.30 mm), width (I) at widest point 0.41 mm (0.38–0.43 mm), article II length 1.40 mm (1.34–1.46 mm), width (II) at widest point 0.40 mm (0.38–0.43 mm), article III length 0.53 mm (0.50–0.57 mm). Article I with raised, setose glandular area on dorsal surface (Figure 8). Article I with boss-like tubercles proximally on proventrolateral and retrolateral surfaces, one individual with proventrolateral tubercles bilobed; proventrolateral tubercle may function to macerate food or manipulate food items in conjunction with the epistome process. Cleavage of corpus and fixed finger housing 5 or 6 setae on prolateral side of article II; 12 or 15 setae on ventral surface of article II; these patches discrete. Palpal coxae yellow-brown to brown, with two seta-bearing tubercles ventrally (Figure 8). Palpal measurements given in Table 5. Trochanter brown to dark-brown with 3 (holotype) or 4 seta-bearing tubercles; femur white; patella white without dark band medially, with prolateral, distal darkened tubercles; bearing microtrichia and small setae distally; tibia white with scattered setae and dense microtrichia; tarsus white, usually darkening distally. Claw rudiment very small.

**Table 5.** Acuclavella makah palpus and leg measurements.<br/>

**Appendage**	**Segment**	**Male**			**Female**		
		**Mean**	**Range**	***n***	**Mean**	**Range**	***n***
Palpus	trochanter	0.58	0.54-0.62	3	0.57	0.54-0.60	3
	femur	0.95	0.90-0.98	10	0.98	0.88-1.05	14
	patella	0.67	0.62-0.72	3	0.70	0.67-0.74	3
	tibia	0.79	0.73-0.84	3	0.72	0.68-0.76	3
	tarsus	0.73	0.71-0.76	3	0.68	0.67-0.68	3
Leg I	trochanter	0.57	0.56-0.60	3	0.53	0.50-0.56	3
	femur	2.67	2.60-2.72	3	2.46	2.36-2.56	3
	patella	0.97	0.92-1.00	3	0.90	0.88-0.92	3
	tibia	1.75	1.72-1.76	3	1.57	1.55-1.60	3
	metatarsus	2.36	2.24-2.44	3	2.13	2.08-2.20	3
	tarsus	2.93	2.76-3.04	3	2.72	2.56-2.80	3
Leg II	trochanter	0.60	0.56-0.64	10	0.61	0.56-0.64	14
	femur	4.01	3.76-4.35	10	3.85	3.60-4.20	14
	patella	1.12	1.00-1.20	10	1.14	1.04-1.28	14
	tibia	2.65	2.40-2.88	10	2.64	2.40-2.92	14
	metatarsus	3.91	3.60-4.25	10	3.80	3.52-4.75	14
	tarsus	5.11	4.55-5.50	10	4.62	4.25-4.94	14
Leg III	trochanter	0.55	0.52-0.56	3	0.57	0.55-0.60	3
	femur	2.47	2.32-2.56	3	2.21	2.12-2.32	3
	patella	0.93	0.92-0.96	3	0.96	0.88-1.05	3
	tibia	1.64	1.60-1.68	3	1.60	1.52-1.65	3
	metatarsus	2.64	2.56-2.76	3	2.48	2.40-2.64	3
	tarsus	3.16	3.00-3.32	3	2.95	2.85-3.12	3
Leg IV	trochanter	0.67	0.60-0.72	3	0.65	0.60-0.72	3
	femur	3.64	3.52-3.80	3	3.34	3.16-3.60	3
	patella	1.05	0.96-1.12	3	1.11	1.04-1.20	3
	tibia	2.32	2.32	3	2.30	2.16-2.44	3
	metatarsus	4.16	4.05-4.30	3	3.99	3.76-4.25	3
	tarsus	4.17	3.85-4.35	3	3.86	3.68-3.95	3

All measurements in millimeters; *n*= sample size

Leg measurements given in Table 5. Trochanters, femora, patellae, tibiae black, lighter at joints, with scattered, distally elevated scales which subtend short setae, scales occasionally house setae apically or posteriorly. Metatarsi of leg III with proximal half black or darkened; leg IV proximal three-quarters black or darkened; legs I and II with proximal end darkened; remaining metatarsal areas pale-brown to yellow-brown. Scaled-microsculpture subequal to darkened areas. Metatarsi and tarsi without tubercles. Six of ten males with false leg articulations on metatarsi of leg II, including holotype. Leg claws single, black, not toothed, evenly curved.

Penis 2.39 mm in length (*n*=2, 2.38–2.40 mm); glans plate length 0.34 mm (*n*=2, both 0.34 mm); stylus length 0.16 mm (0.14–0.17 mm). Shaft evenly tapered, broadening slightly at glans; glans with scattered small setae; stylus spirally twisted, very slightly decurved.

**Description of female.** Similar to male for nearly all characters. Total length 5.14 mm (*n*=3; 5.00–5.31 mm); carapace length 1.37 mm (*n*=14; 1.25–1.81 mm); carapace width 3.16 mm (*n*=3; 3.00–3.31 mm); scutum length 3.82 mm (*n*=3; 3.63–3.94 mm); scutum width 3.33 mm (*n*=3; 3.19–3.50 mm); length of fused sternites I-V 2.96 mm (*n*=14; 2.75–3.19 mm).

Eye tubercle height above surface of carapace 1.37 mm (*n*=3; 1.31–1.46 mm); distance from ventral surface of eye to tip of spine 1.21 mm (*n*=14; 1.08–1.33 mm). Eye color brown. Metapeltidial spine 0.16 mm (*n*=14; 0.10–0.23 mm).

Paramedian tubercles of tergite I height 0.06 mm (*n*=14; 0.03–0.10 mm) above surface of tergite. Tergite II paramedian tubercles greatly enlarged spines standing 1.10 mm (*n*=14; 0.93–1.28 mm) above tergite surface; tubercles lateral to spines warty mounds. Paramedian tubercle height of tergite III 0.07 mm (*n*=14; 0.05–0.10 mm); IV 0.06 mm (*n*=14; 0.03–0.08 mm). Tergites III, IV, V with three pair of tubercles with median pair largest, reducing in size laterally along tergite and posteriorly across tergites; the right median tubercle of tergite III of paratype (CAS) enlarged into mound with diminished frequency of warts standing 0.22 mm above carapace. First free tergite (IV) with median and lateral pair of enlarged warty tubercles, or barely distinguishable median pair of enlarged warts; tergite VII without tubercles, or tubercles barely distinguishable, or more noticeable; tergite VIII without distinguishable tubercles.

Sternites brown. Prosomal sternum length 0.17 mm (*n*=2, 0.17–0.18 mm), width 0.32 mm (0.30–0.33 mm); dark brown. Labium wider (0.17 mm, 0.16 mm, 0.14 mm) than long (0.09 mm, 0.09 mm, 0.14 mm), or nearly equal; yellow-brown to light brown. Palpal endites brown, yellow-brown, or light brown. Paratypes (AMNH, CAS) with 3 setae on leg II endite; paratype (UWBM) with 2 setae. Horn-shaped process of epistome decurved or projecting downward; length 0.48 mm (*n*=2, 0.47–0.50 mm).

Chelicerae article I length 1.35 mm (*n*=3; 1.20–1.47 mm), width 0.45 mm (*n*=3, 0.44–0.46 mm); article II length 1.49 mm (*n*=3; 1.48–1.50 mm), width 0.42 mm (0.42–0.43); article III length 0.59 mm (*n*=3; 0.56–0.60 mm). Article I without raised glandular mound (Figure 8); prolateral side of cheliceral article II with 2 or 4 setae at cleavage of corpus and fixed finger; 12 setae on ventral surface of article II; these patches discrete. Palpus dimensions in Table 5; coxae brown with two seta-bearing tubercles; trochanter brown to dark brown with 4 seta-bearing tubercles; only paratype with tubercle on patella.

Leg measurements given in Table 5. Leg trochanters, femora, patellae, and tibiae black, lighter at joints; metatarsi of leg III with proximal half black or darkened, leg IV with proximal three-quarters black or darkened, proximal end of legs I and II black or darkened, remaining metatarsal areas yellow-brown or pale brown; tarsi yellow-brown or pale brown, darkening distally.

Ovipositor length 0.74 mm (*n*=2, 0.72–0.77 mm), width 0.46 mm (0.44–0.47 mm); furca without dorsoventral differentiation; corona of setae at furcal base surround lobes; apical setae on lobes.

#### Distribution and habitat.

Known from the northwest areas of the Olympic Peninsula in Clallam and Jefferson Counties, Washington State (Appendix I). Found in coniferous or riparian forests along small, perennial water-features such as headwater streams, springs, and seeps; underneath woody debris and moss.

### Acuclavella sheari sp. n.

urn:lsid:zoobank.org:act:FC134B12-DA1C-4184-B9AB-6AC36E990BB5

http://species-id.net/wiki/Acuclavella_sheari

MorphBank images of specimens considered this species include:

Holotype AMNH, MorphBank Specimen Id: 822513, 4 images

Paratype AMNH, MorphBank Specimen Id: 822512, 6 images

SDSU OP2708, MorphBank Specimen Id: 822514, 3 images

SDSU OP2720, MorphBank Specimen Id: 828518, 3 images

**Figure 9.**
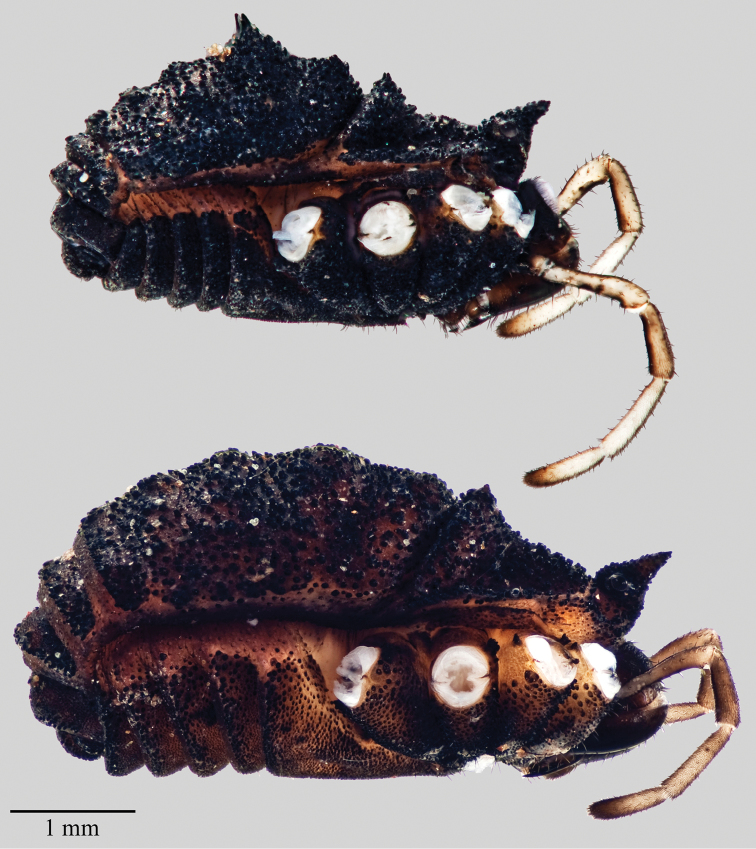
*Acuclavella sheari*. Top: male, CHR3254; bottom: female, CHR3404.

**Figure 10.**
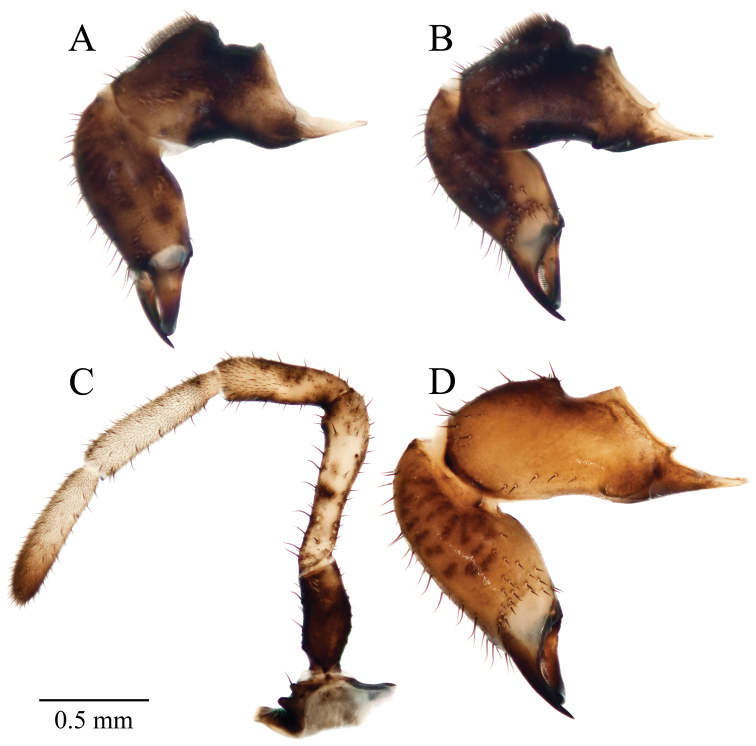
*Acuclavella sheari* chelicerae and pedipalp. **A** right chelicera, retrolateral view **B** left chelicera, prolateral view **C** right pedipalp, retrolateral view (A–C, male CASENT9039217) **D** left chelicera, retrolateral view (female AMNH).

Figure 9 and 10 , Appendix VIII: Figure 1, Figure 2 

#### Type material.

Male **holotype** (AMNH) and female **paratypes** (AMNH; CAS, CASENT9039225; UWBM) from Burgdorf Road 12.4 miles northwest of Warren Wagon Rd, tributary of Fall Creek, Payette National Forest, Idaho County, Idaho; two male **paratypes** (CAS, CASENT9039217; UWBM, ID0016/5360) from Burgdorf Road 16.6 miles northwest of Warren Wagon Road; otherwise as previous (Appendix I).

#### Etymology.

The specific epithet honors Dr. William A. Shear, eminent milliped and opilionid taxonomist. His influence has been important to the authors’ aspirations to be systematic biologists, and we thank him sincerely, and with pleasure.

#### Diagnosis.

Generally reduced dimensions overall. Males with one pair of scutal spines on area II only; scutal spines ≤ 0.50 mm, distance from ventral edge of eye to tip of ocularium ≤ 0.60 mm distinguishes it from other males with single pair of spines. Females lack scutal spines. Diagnosed from spine-less *Acuclavella shoshone* (Shear, 1986 *sensu stricto*) females by having palpal femora ≤ 0.88 mm, leg II tarsi ≤ 3.92 mm, leg II femora ≤ 2.75 mm.

#### Description.

**Description of male.** Body arched and convex dorsally (Figure 9); sides broader posteriorly. Nearly all of body heavily sclerotized black or dark brown, with densely scattered hemispherical warts which irregularly house short setae apically or posteriorly; bilobed warts sporadic in holotype. Total length 3.90 mm (*n*=3, 3.68–4.06 mm), carapace length 1.24 mm (*n*=4, 1.15–1.32 mm), carapace width 2.34 mm (*n*=3, 2.31–2.36 mm); length of fused tergites I-V 2.26 mm (*n*=4, 2.16–2.31 mm), scutum length 2.61 mm (*n*=3, 2.32–2.81 mm), scutum width 2.60 mm (*n*=3, 2.55–2.63 mm).

Eye tubercle at anterior edge of carapace, hemispherical warts cover entirety of ocular spine, prolonged into an acute spine standing 0.67 mm above the surface of the carapace (*n*=3, 0.64–0.70 mm), 0.53 mm (*n*=4, 0.48–0.60 mm) from the ventral edge of the eye to the tip of the tubercle, eye spine less acute than in other species of *Acuclavella*. Eyes dark brown to brown, located basally on tubercle. Surface of carapace evenly curved, posterior margin arcuate. Metapeltidial paramedian sensory cones raised into sharp, acute spines standing 0.21 mm (*n*=4, 0.17–0.24 mm) above surface of the carapace, curving slightly towards the midline, lacking warty microsculpture, shiny; lateral to spines, clusters of warts form tubercles, missing in paratype (UWBM).

All scutal tergites with pairs of paramedian tubercles; relatively short, pointed spines on area II only. Fused tergite I with paramedian tubercles as raised mounds, relatively tall but not spike-like, standing 0.125 mm above the surrounding scute (*n*=4, all 0.125), these adorned with warts; lateral to these, two additional raised mounds become smaller away from the midline, these lacking in holotype. Paramedian tubercles of area II form acute spine 0.39 mm above the scutum surface (*n*=4, 0.34–0.43 mm), curved slightly posteriad; lateral to these are raised tubercles adorned with warts. Fused tergites III, IV, and V with paramedian tubercles in the form of raised mound adorned with warts; lateral to these, area III with 2 or 3 additional pairs of tubercles, area IV with 1 or 2 additional tubercles, area V with 0 or 2 additional tubercles; these tend to diminish in size away from the midline and posteriorly across tergites. Area III tubercle height above tergite 0.081 mm (*n*=4, 0.075–0.10 mm); area IV tubercle height 0.075 mm (all 0.075 mm). Free tergites without discernable tubercles, or tubercles occur in single pairs as small warty mounds.

Abdominal sternites with infrequent setae; warty sculpturing strongest laterally and on posterior margin; sternites brown, dark brown, or black. Sclerotized areas of genital operculum relatively setose. Prosomal sternum (*n*=1) wider than long: length 0.19 mm, width 0.29 mm; dark brown; without setae. Labium weakly to well-sclerotized, wider than long (*n*=3):, length 0.09 mm (0.08-0.10 mm), width 0.16 mm (0.13-0.19 mm), light brown to dark brown, darkness commensurate to sclerotization, without setae. All coxae with endites. Palpal endites setose, light brown, dark brown, or black. Leg II endite bearing 2 setae, other leg endites unadorned. Horn-shaped process of epistome decurved, projecting 0.34 mm from sulcus.

Chelicerae light brown, dark brown, or black; lighter individuals with article I darker dorsally, article II with darker, more sclerotized striations on prolateral and retrolateral surfaces (Figure 10). Article I length 1.11 mm (*n*=3, 1.00-1.18 mm), width 0.39 mm (0.36-0.42 mm), article II length 1.21 mm (1.12-1.27 mm), width 0.40 mm (all 0.40 mm), article III length 0.54 mm (0.52-0.56 mm). Dorsal surface of article I with raised, glandular area dense with setae. Palpal coxae pale-brown, light brown, or black, with 2 or 1 (UWBM) seta-bearing tubercles. Palpal measurements given in Table 6. Trochanter (Figure 10) pale-brown or dark brown, with 2 (CAS), 3 (holotype), or 4 (UWBM) seta-bearing tubercles; these reduced on later individual; femur pale-brown, brown, or dark brown; patella pale-brown or brown, with (CAS) or without a diffuse dark band on middle third, without darkened tubercle distally, bearing small setae and microtrichia distally; tibiae pale brown or white (holotype), with scattered setae and dense microtrichia; tarsus pale-brown or white (holotype), darkening to brown distally. Claw rudiment very small to absent.

**Table 6.** *Acuclavella sheari* palpus and leg measurements.<br/>

**Appendage**	**Segment**	**Male**			**Female**		
		**Mean**	**Range**	***n***	**Mean**	**Range**	***n***
Palpus	trochanter	0.51	0.49–0.54	3	0.50	0.43–0.54	3
	Femur	0.85	0.78–0.88	4	0.85	0.83–0.88	4
	Patella	0.63	0.62–0.65	3	0.66	0.60–0.70	3
	Tibia	0.74	0.72–0.75	3	0.73	0.65–0.79	3
	Tarsus	0.70	0.67–0.71	3	0.65	0.59–0.68	3
Leg I	trochanter	0.53	0.48–0.56	3	0.45	0.40–0.50	3
	Femur	1.80	1.76–1.84	3	1.73	1.48–1.94	3
	Patella	0.87	0.84–0.92	3	0.81	0.68–0.91	3
	Tibia	1.33	1.28–1.44	3	1.30	1.16–1.40	3
	metatarsus	1.97	1.96–2.00	3	1.88	1.68–2.09	3
	Tarsus	2.29	2.24–2.32	3	2.22	1.88–2.41	3
Leg II	trochanter	0.57	0.56–0.60	4	0.52	0.44–0.63	4
	Femur	2.53	2.48–2.64	4	2.52	2.16–2.75	3
	Patella	0.98	0.88–1.12	4	0.93	0.76–1.03	3
	Tibia	1.96	1.88–2.08	4	1.90	1.64–2.09	3
	metatarsus	2.99	2.88–3.12	4	3.00	2.56–3.24	3
	Tarsus	3.67	3.64–3.72	4	3.65	3.20–3.92	3
Leg III	trochanter	0.53	0.52–0.56	3	0.52	0.44–0.59	3
	Femur	1.72	1.64–1.80	3	1.71	1.56–1.84	3
	Patella	0.85	0.84–0.88	3	0.79	0.48–0.97	3
	Tibia	1.33	1.20–1.48	3	1.43	1.32–1.56	3
	metatarsus	2.21	2.16–2.28	3	2.18	1.84–2.38	3
	Tarsus	2.56	2.52–2.64	3	2.53	2.20–2.84	3
Leg IV	trochanter	0.59	0.52–0.68	3	0.59	0.52–0.69	3
	Femur	2.41	2.32–2.56	3	2.38	2.12–2.63	3
	Patella	0.96	0.88–1.04	3	0.89	0.80–0.94	3
	Tibia	1.84	1.76–1.88	3	1.77	1.56–2.03	3
	metatarsus	3.31	3.28–3.32	3	3.24	2.80–3.48	3
	Tarsus	3.23	3.08–3.32	3	3.29	2.80–3.68	3

All measurements in millimeters; *n*=sample size

Leg measurements given in Table 6. Microsculpture of femora, patellae, and tibiae scattered, distally elevated scales, bilobed scales not observed; scales subtend setae, occasionally housing seta apically. Trochanters, femora, patellae, tibiae black, or in holotype, trochanters light brown, femora, patellae, and tibiae brown; metatarsi of leg III proximal one-third to one-quarter brown or black; leg IV with proximal half black or brown; proximal end of metatarsi on legs I and II black or brown, remaining metatarsal areas pale brown or brown; all tarsi pale brown or brown, darkening distally. Scaled microsculpture subequal to darkened areas, remainder with setae and microtrichia. False leg articulations not observed. Leg claws single, black, not toothed, evenly curved.

Penis length 2.94 mm (*n*=1); glans plate 0.31 mm, scattered small setae; stylus 0.13 mm, slightly spirally twisted and decurved.

**Description of female.** Similar to male, differing in dimensions, secondary sexual characteristics, and dorsal armature. Total length 4.25 mm (*n*=3; 4.05-4.65 mm); carapace length 1.325 mm (*n*=4, 1.05-1.45 mm), width 2.62 mm (*n*=3, 2.35-3.05 mm); scutum length 3.03 (*n*=3, 2.90-3.25 mm), width 3.23 mm (*n*=3, 2.85-3.55 mm); fused tergites I-V length in midline 2.81 mm (*n*=4, 2.20-3.10 mm).

Eye tubercle height above surface of carapace 0.67 mm (*n*=3, 0.56-0.73 mm), distance from ventral surface of eye to tip of spine 0.54 mm (*n*=4, 0.46-0.60 mm). Eye color brown or gray. Metapeltidial spine 0.11 mm (*n*=4, 0.08-0.15 mm), curving slightly posteriad and towards the midline, lateral to these spines clusters of warts form tubercles.

Dorsal armature lacking. Dorsal adornment generally with median tubercles as raised mounds adorned with warts, decreasing in size away from midline and posteriorly across tergites. Two lateral pairs of warts on areas III and IV, area V with median pair of tubercles barely discernable, two pairs of lateral tubercles, or shallowly raised mounds adorned with warts. Free tergites with tubercles in the form of clusters of warts, or without discernable tubercles. Paramedian tubercles heights above the surface of the tergite (*n*=4): of area I 0.07 mm (0.05-0.08 mm); area II 0.10 mm (0.08-0.15 mm); area III 0.06 mm (0.05-0.08 mm); area IV 0.05 mm (0.03-0.08 mm).

Sternites brown, black, or light brown. Prosomal sternum (*n*=1) length: 0.26 mm, width 0.31 mm; light brown, without setae. Labium wider (0.13, 0.17, 0.16 mm) than long (0.08, 0.09, 0.08 mm); light brown or pale brown. Palpal endites brown or pale brown. Endites of leg II adorned with two or one (CAS) setae.

Chelicerae article I length 0.98 mm (*n*=3, 0.92-1.06 mm), width 0.41 mm (0.37-0.44 mm); article II length 1.31 mm (1.14-1.40 mm), width 0.43 mm (0.40-0.46 mm); article III length 0.57 mm (0.52-0.62 mm). Article I without raised glandular mound (Figure 10). Article I darker dorsally, chelicerae light brown or black with areas of dark brown. Palpus dimensions in Table 6; coxae light brown, dark brown, or white, with two seta-bearing tubercles; trochanters light brown, dark brown, or white, with three (AMNH) or four (CAS, UWBM) seta-bearing tubercles; femora and patella pale brown, brown, or white, patella without both distal tubercles and diffuse bands; tibiae white or pale-brown; tarsus white or pale-brown, fading to brown distally. Claw rudiment very small (AMNH, UWBM), or absent.

Leg measurements given in Table 6. Leg trochanters brown, black, or pale-brown; femora, patellae, and tibiae dark brown, back, or pale brown; metatarsi of legs III with proximal third brown, black, or pale-brown; leg IV metatarsi with proximal half brown, black, or pale-brown; proximal end of legs I and II metatarsi brown, black, or pale-brown; remaining metatarsal areas pale-brown, brown, or white; tarsi pale brown or white, darkening distally.

Ovipositor length 0.82 mm, width 0.46 mm (*n*=1); otherwise as *Acuclavella makah*.

#### Distribution and habitat.

Known from north-facing, horizontal band of *Abies grandis* forest above south shore of Salmon River. This habitat is mainly roadless, though intersected by FS 592 and Burgdorf Road in Payette National Forest, Idaho County, Idaho. Adults collected in June and September. *Abies grandis*, *Pseudotsuga menziesii*, and *Picea engelmannii* associated canopy cover; collected under woody debris adjacent to headwater streams.

### Acuclavella quattuor

Shear, 1986

http://species-id.net/wiki/Acuclavella_quattuor

MorphBank images of specimens considered this species include:

AMNH, MorphBank Specimen Id: 822462, 3 images

CHR2146.2, MorphBank Specimen Id: 822463, 3 images

CHR2146.5, MorphBank Specimen Id: 822464, 3 images

CHR2147.7, MorphBank Specimen Id: 822604, 1 image

SDSU OP2256, MorphBank Specimen Id: 822496, 3 images

SDSU OP2266, MorphBank Specimen Id: 822605, 1 image

CHR2180.0, MorphBank Specimen Id: 822606, 2 images

CHR2180.3, MorphBank Specimen Id: 822607, 1 image

**Figure 11.**
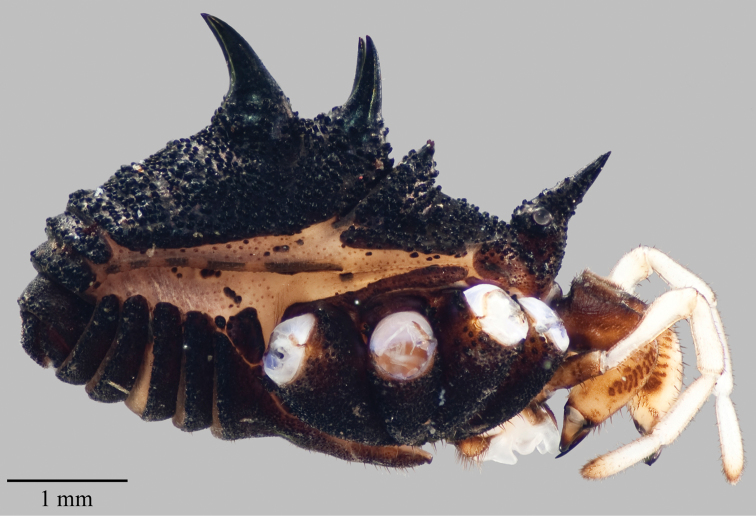
*Acuclavella quattuor*. Male AMNH.

**Figure 12.**
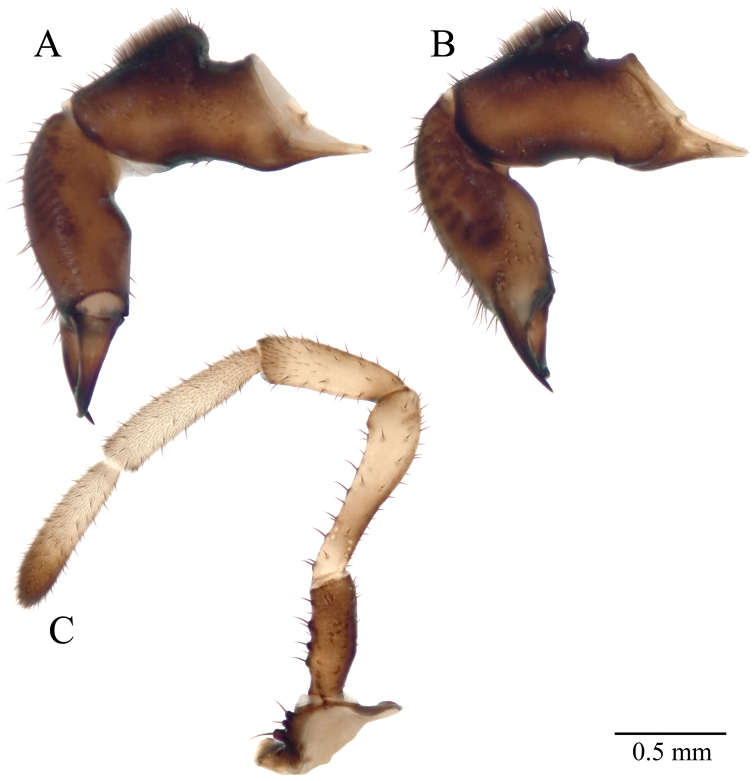
*Acuclavella quattuor* chelicerae and pedipalp. Male CHR2146.5, **A** right chelicera, retrolateral view **B** left chelicera, prolateral view **C** right pedipalp, retrolateral view.

Figure 11 and 12 , Appendix VIII: Figure 1, Figure 2 

#### Material examined.

Males (AMNH; CAS, CASENT9039220; UWBM, ID0013/5661) from the type locality: Slate Creek Road 10.2 miles east of US 95, Nez Perce National Forest, Idaho County, Idaho.

#### Diagnosis.

Diagnosed from all *Acuclavella* species except for *Acuclavella* cf. *quattuor* by having two pairs of erect spines on tergites I and II; similar morphologically to *Acuclavella* cf. *quattuor*, and is best diagnosed using molecular data: basepair 23 of the EF-1α intron cytosine in *Acuclavella* cf. *quattuor*, adenine in *Acuclavella quattuor*; basepair 44 guanine in *Acuclavella quattuor*, thymine or adenine in *Acuclavella* cf. *quattuor* (thymine if sequences aligned due to a 3 basepair deletion in 40% of *Acuclavella* cf. *quattuor* at basepair 39–41).

#### Description.

**Description of male.** Body arched and convex dorsally (Figure 11); sides parallel or slightly broader posteriorly. Nearly all of body heavily sclerotized, black, with densely scattered hemispherical warts which irregularly house short setae apically or posteriorly. Total length 4.22 mm (*n*=3, 4.06–4.45 mm); carapace length 1.35 mm (*n*=14, 1.25–1.50 mm), width 2.60 mm (*n*=3, 2.56–2.64 mm); scutum length 2.84 mm (*n*=3, 2.56–3.25 mm), width 2.80 mm (*n*=3, 2.69–2.88 mm); length fused tergites I-V 2.69 mm (*n*=14, 2.50–3.10 mm).

Eye tubercle at anterior edge of carapace, erect, spine-like, standing 0.98 mm above the surface of the carapace (*n*=3, 0.94–1.05 mm), distance from ventral edge of eye to tip of spine 0.84 mm (*n*=14, 0.65–0.93 mm). Eye color brown, on basal part of ocularium. Surface of carapace evenly curved, posterior margin arcuate. Metapeltidial paramedian sensory cones acute or blunt spines standing 0.28 mm (*n*=14, 0.18-0.38 mm) above surface of carapace.

Scutum of opisthosoma rounded anteriorly, squared of posteriorly. All fused tergites with paramedian pair of tubercles, these in the form of erect spines on areas I and II. Spines of tergites I and II curve posteriad, stand 0.82 mm (*n*=14, 0.65-1.05 mm) and 0.91 (*n*=14, 0.70-1.12 mm) above the surface of the tergite respectively; tubercles lateral to spines as small raised wart mounds; one of 14 males with lateral spines on area II. Tergites III, IV, and V with four pairs of tubercles as raised areas adorned with warts; paramedian tubercles of area III height 0.06 mm (*n*=14, 0.03-0.20 mm); area IV paramedian tubercle height 0.05 mm (*n*=14, 0.03-0.08 mm); two individuals with one of paramedian tubercles of area III spine-like, left side on one, right side on other. First free tergite (VI) with small median tubercles in the form of enlarged shiny wart; remaining free tergites without discernable tubercles.

Abdominal sternite warty sculpturing strongest laterally and on posterior margin; sternites brown or dark brown. Genital operculum tongue-shaped, clearly delineated by transverse furrow, lateral ends of furrow membranous suture, distal margin rebordered, glossy. Prosomal sternum wider than long; length 0.18 mm (*n*=3, 0.17-0.21 mm), width 0.29 mm (*n*=3, 0.26-0.31 mm); pale brown or brown; without setae. Labium moderately sclerotized, wider than long or dimensions subequal; length 0.11 mm (*n*=3, 0.09-0.13 mm), width 0.14 mm (0.13-0.16 mm); brown or dark brown; without setae. Palpal endites setose; brown. Leg II endites bearing 2 (AMNH, CAS) or 3 setae (UWBM), other leg endites glabrous. Horn-shaped process of epistome strongly decurved; projecting 0.43 mm (*n*=4, 0.41-0.44 mm) from sulcus.

Chelicerae light brown or brown; darker dorsally; article II with darker, more sclerotized striations on prolateral and retrolateral surfaces (Figure 12). Article I length 1.21 mm (*n*=3, 1.18-1.25 mm), width 0.44 mm (*n*=3, 0.42-0.45 mm); article II length 1.38 mm (*n*=3, 1.35-1.40 mm), width 0.41 mm (*n*=3, 0.38-0.45 mm); article III length 0.59 mm (*n*=3, 0.58-0.60 mm). Article I dorsal surface with raised, glandular area densely setose (Figure 12). Prolateral side of article II with dark sclerotization at cleavage of corpus and fixed finger housing 6, 6, 5, 5 setae; ventral surface of article with 19, 20, 16, 23 setae; these patches discrete in three of four individuals examined. Palpal measurements given in Table 7. Palpal coxae light brown or brown (Figure 12), with 2 seta-bearing tubercles. Palpal trochanters light brown, brown, or dark brown; with 4 seta-bearing tubercles. Femora and patella white or yellow-brown; patella without distal prolateral tubercle, without diffuse dark band, bearing small setae and microtrichia distally. Tibia white or pale yellow, with scattered setae and dense microtrichia. Tarsi white or pale yellow, darkening distally; with setae and dense microtrichia. Claw rudiment very small.

**Table 7.** *Acuclavella quattuor* male palpus and leg measurements.<br/>

		**Palpus**	**Leg I**	**Leg II**	**Leg III**	**Leg IV**
Trochanter	Mean	0.53	0.61	0.61	0.51	0.64
	Range	0.52–0.54	0.60–0.62	0.56–0.68	0.48–0.56	0.56–0.75
	*n*	3	3	14	3	3
Femur	Mean	1.01	2.31	3.14	2.00	2.88
	Range	0.95–1.10	2.20–2.44	2.72–3.44	1.96–2.04	2.76–3.00
	*n*	14	3	14	3	3
Patella	Mean	0.67	0.97	1.08	0.95	1.10
	Range	0.64–0.73	0.92–1.03	1.00–1.20	0.92–1.00	0.96–1.20
	*n*	3	3	14	3	3
Tibia	Mean	0.83	1.54	2.21	1.54	2.11
	Range	0.80–0.88	1.48–1.58	2.00–2.41	1.48–1.64	2.00–2.20
	*n*	3	3	14	3	3
Metatarsus	Mean	-	2.36	3.55	2.61	3.98
	Range	-	2.16–2.63	2.96–4.08	2.48–2.80	3.68–4.25
	*n*	-	3	14	3	3
Tarsus	Mean	0.77	2.74	4.71	3.10	4.04
	Range	0.73–0.85	2.48–3.03	3.90–5.25	2.76–3.28	3.56–4.31
	*n*	3	3	14	3	3

All measurements in millimeters, *n*=sample size.

Leg measurements given in Table 7. Microsculpture of femora, patellae, and tibiae scattered, distally elevated scales, bilobed scales not observed; scales infrequently house seta apically or distally. Trochanters, femora, patellae, and tibiae dark brown to black; metatarsi of leg III with proximal one-quarter brown or black, leg IV with proximal one-half brown or black, proximal end of metatarsi of I and II brown or black, remaining metatarsal areas brown; tarsi brown, darkening distally. Leg claws single, black, not toothed, evenly curved.

Penis length 2.49 mm (*n*=4, 2.35–2.55 mm); shaft evenly tapered, broadening slightly at glans. Glans bearing scattered small setae; glans length 0.32 mm (*n*=3, 0.29–0.36 mm). Stylus length 0.18 mm (*n*=3, 0.16–0.19 mm); stylus spirally twisted, decurved.

For female description see Shear (1986).

#### Distribution and habitat.

*Acuclavella quattuor* populations are bracketed by the Salmon River to the south and the South Fork Clearwater River to the north, whereas *Acuclavella* cf. *quattuor* is found north of the Selway River and south of the Lolo Trail Ridge. *Acuclavella quattuor* habitats are dominated by *Abies grandis* and *Picea englemannii*; microhabitat in litter, moss, and moist woody debris adjacent to headwater streams.

### Acuclavella merickeli

Shear, 1986

http://species-id.net/wiki/Acuclavella_merickeli

MorphBank images of specimens considered this species include:

CHR2100.1, MorphBank Specimen Id: 822457, 2 images; type locality

CHR2100.2, MorphBank Specimen Id: 822597, 1 image

CHR2121.2, MorphBank Specimen Id: 822601, 3 images

CHR2121.4, MorphBank Specimen Id: 822602, 2 images

CHR2121.5, MorphBank Specimen Id: 822459, 3 images

CHR2140.0, MorphBank Specimen Id: 822460, 3 images

CHR2140.2, MorphBank Specimen Id: 822603, 1 image

Figures: Appendix IX 

#### Diagnosis.

For species descriptions see Shear 1986. Discriminated from other *Acuclavella* having a single pair of paramedian spines on tergite II by having relatively homogenously colored leg segments, not giving the appearance of banding at leg joints (from *Acuclavella makah* and *Acuclavella leonardi*) or by having scutal spines > 0.50 mm and ocularium height from ventral edge of eye to tip of spine > 0.60 mm (from *Acuclavella sheari*).

#### Distribution and habitat.

*Acuclavella merickeli* populations are bracketed to the south by the South Fork Clearwater River and to the north by the Selway River; all localities in Nez Perce National Forest, Idaho County, Idaho. Coniferous habitat dominated by *Picea englemannii* or *Thuja plicata* and *Pseudotsuga menziesii*; microhabitats include moist woody debris and moss adjacent to small perennial water features such as side-slope seeps and headwater streams.

## Supplementary Material

XML Treatment for Acuclavella

XML Treatment for Acuclavella leonardi

XML Treatment for Acuclavella makah

XML Treatment for Acuclavella sheari

XML Treatment for Acuclavella quattuor

XML Treatment for Acuclavella merickeli

## Appendix I

Collection Locality Information (doi: 10.3897/zookeys.311.2920.app1) File format: Adobe PDF file (pdf).

## Appendix II

PCR Primer Information. (doi: 10.3897/zookeys.311.2920.app2) File format: Adobe PDF file (pdf).

## Appendix III

Male and Female Morphometric Data and Measurement Scheme. (doi: 10.3897/zookeys.311.2920.app3) File format: Microsoft Office Excel (xlsx).

## Appendix IV

PCA Methods and Results. (doi: 10.3897/zookeys.311.2920.app4) File format: Adobe PDF file (pdf).

## Appendix V

Morphologies of Acuclavella cf. cosmetoides and Geography of Morphotypes. Figure 1: male morphologies. Figure 2: female morphologies. Figure 3: geographic distribution of male morphologies. Figure 4:geographic distribution of female morphologies. (doi: 10.3897/zookeys.311.2920.app5) File format: Adobe PDF file (pdf).

## Appendix VI

Acuclavella distribution. KMZ file of Acuclavella sampling effort, species distributions, and distribution of individuals used in concatenated phylogenetic reconstruction. (doi: 10.3897/zookeys.311.2920.app6) File format: Google Earth Keyhole Markup (kmz).

## Appendix VII

Phylogenetic Trees. Figure 1: Outgroup relationships from Bayesian gene trees. Figure 2: RAxML concatenated phylogeny. Figure 3: RAxML COI gene tree. Figure 4: RAxML EF-1α gene tree. Figure 5: 28S and Wnt2 RAxML gene trees. (doi: 10.3897/zookeys.311.2920.app7) File format: Adobe PDF file (pdf).

## Appendix VIII

Acuclavella genitalia. Figure 1: penises. Figure 2: ovipositors. (doi: 10.3897/zookeys.311.2920.app8) File format: Adobe PDF file (pdf).

## Appendix IX

K. Leg II Morphologies. With and without femoral false leg articulations. (doi: 10.3897/zookeys.311.2920.app9) File format: Adobe PDF file (pdf).
