# Supplementary material for: Three new species in the harvestmen genus Acuclavella (Opiliones, Dyspnoi, Ischyropsalidoidea), including description of male Acuclavella quattuor Shear, 1986
Source: Zookeys. 2013 Jun 20;(311):19–68. doi: 10.3897/zookeys.311.2920 (PMC3698555; doi:10.3897/zookeys.311.2920)
Supplement: Supplementary file 7 — Collection Locality Information (doi: 10.3897/zookeys.311.2920.app1) File format: Adobe PDF file (pdf). [file ZooKeys-311-019-s001.pdf]

Table A.1 Collecting Locality Information.

Taxon	Voucher Number	Sex	Date	Latitude, Longitude°	Elev. (m)	Habitat; Microhabitat	Locality
A. makah	CASENT9039219<br>AMNH<br>AMNH<br>CASENT9039219<br>OP2716	M<br>M<br>F<br>F<br>M	4-Aug-09	48.2043, -124.4402	90	*Tsuga heterophylla*, *Thuja plicata*, *Alnus rubra*, *Rubus spectabilis*; stream-side woody debris	WA, Clallam Co, 2.1 km (1.3 mi) N of Hoko-Ozette Rd 11.4 km (7.1 mi) W of SR 112, tributary of Brownes Creek
A. makah	OP2715	F	4-Aug-09	48.2002, -124.4318	35	*Alnus rubra*, *Tsuga heterophylla*, *Rubus spectabilis*; stream-side woody debris	WA, Clallam Co, Hoko-Ozette Rd 11.3 km (7.0 mi) SW of SR 112, tributary of Hoko River
A. makah	CHR3387.0<br>CHR3387.1<br>CHR3387.2<br>CHR3387.3<br>OP2717<br>OP2718	M<br>F<br>F<br>F<br>F<br>F	5-Aug-09	47.9571, -124.2215	170	*Alnus rubra*, *Picea sitchensis*; stream-side woody debris	WA, Clallam Co, Olympic Nat For, FS 29 15.8 km (9.8 mi) E of US 101, tributary of Sitkum River
A. makah	CHR2457.0<br>CHR2457.1<br>CHR2457.2<br>OP2343<br>OP2344	M<br>M<br>F<br>M<br>F	4-Aug-08	47.8133, -124.1051	133	*Alnus rubra*, *Rubus spectabilis* riparian bracketed by *Tsuga heterophylla*, *Picea sitchensis*; stream-side woody debris	WA, Jefferson Co, Upper Hoh Rd 13.4 km (8.3 mi) E of US 101, Pole Creek
A. makah	OP1699<br>OP1818	M<br>M	2-Jul-07	47.7105, -124.4095	10	*Alnus rubra*, *Rubus spectabilis*, *Picea sitchensis*; stream-side woody debris	WA, Jefferson Co, Cedar Creek east of US 101
	OP2345<br>OP2346	M<br>F	4-Aug-08				
A. makah	UWBM WA2393/7641<br>UWBM WA2393/7641<br>OP2719	M<br>F<br>F	5-Aug-09	47.6661, -124.1433	155	*Tsuga heterophylla*, *Alnus rubra*, *Polystichum munitum*; moss, stream-side woody debris	WA, Jefferson Co, Olympic Nat For, Yahoo Lake Rd 2.3 km (1.4 mi) E of Hoh-Clearwater Road, tributary of Clearwater River
A. leonardi	UWBM WA2392/6319<br>CASENT9039224<br>OP2714	M<br>F<br>M	29-Jul-09	46.3019, -121.9719	1065	*Pseudotsuga menziesii*, *Tsuga heterophylla*, *Abies grandis*; stream-side woody debris	WA, Skamania Co, Gifford Pinchot Nat For, FS 28 0.2 km (0.1 mi) E of FS 25, Iron Creek

(table continues)

Table A.1 Collecting Locality Information (continued).

Taxon	Voucher Number	Sex	Date	Latitude, Longitude°	Elev. (m)	Habitat; Microhabitat	Locality
A. leonardi	OP2712<br>OP2713	F<br>M	28-Jul-09	46.5625, -122.4832	290	*Tsuga heterophylla* tree farm, *Alnus rubra* riparian; stream-side woody debris	WA, Lewis Co, Kjesbu Road 0.6 km (0.4 mi) N of Flynn Rd, Harmony Creek
A. leonardi	AMNH<br>CASENT9039218<br>AMNH<br>OP2347<br>OP2348	M<br>M<br>F<br>M<br>M	6-Aug-08	46.4033, -121.9902	676	*Alnus rubra*, *Tsuga heterophylla*; stream-side woody debris	WA, Lewis Co, Gifford Pinchot Nat For, FS 250 7.4 km (4.6 mi) S of FS 300, tributary of Iron Ck
A. leonardi	OP2349	F	7-Aug-08	46.1212, -122.7598	130	*Alnus rubra*, *Acer macrophyllum*, *Thuja plicata*, *Pseudotsuga menziesii*; stream-side woody debris	WA, Cowlitz Co, Mahaffey Rd 0.8 km (0.5 mi) east of Goble Creek Loop, North Fork Goble Creek
A. quattuor	AMNH<br>CHR2146.2<br>CASENT9039220<br>UWBM ID0013/5661<br>CHR2146.5<br>CHR2146.6<br>CHR2146.7<br>OP2256<br>OP2257<br>OP2258<br>OP2259<br>OP2260<br>OP2261	M<br>F<br>M<br>M<br>M<br>M<br>M<br>F<br>F<br>F<br>F<br>F<br>F	8-Jul-08	45.6385, -116.1096	920	*Picea engelmannii*; stream-side woody debris	ID, Idaho Co, Nez Perce Nat For, Slate Ck Rd 16.4 km (10.2 mi) E US 95, tributary Slate Creek
A. quattuor	OP2242<br>OP2243<br>OP2244	M<br>F<br>M	6-Jul-08	45.8120, -115.9530	1005	*Abies grandis*; litter, stream-side woody debris	ID, Idaho Co, Nez Perce Nat For, FS 279 3.1 km (1.9 mi) SW of Hungry Ridge Rd, Grouse Creek
A. quattuor	OP2255	F	7-Jul-08	45.5791, -115.4431	1870	*Abies grandis*; moss, stream-side woody debris	ID, Idaho Co, Nez Perce Nat For, FS 222 22.7 km (14.1 mi) S of Red Rv Rd, tributary of Crooked Creek

(table continues)

Table A.1 Collecting Locality Information (continued).

Taxon	Voucher Number	Sex	Date	Latitude, Longitude°	Elev. (m)	Habitat; Microhabitat	Locality
A. quattuor	OP2266<br>OP2267	M<br>F	9-Jul-08	45.6853, -115.5427	1460	*Picea engelmannii*; stream-side woody debris	ID, Idaho Co, Nez Perce Nat For, FS 311 20.9 km (13.0 mi) S of SR 14 via Crooked River Rd, EF Crooked River
A. quattuor	CHR2176.0<br>CHR2176.1<br>OP2268<br>OP2269	M<br>M<br>F<br>F	9-Jul-08	45.6338, -115.4177	1700	*Picea engelmannii*; moss, stream-side woody debris	ID, Idaho Co, Nez Perce Nat For, FS 222 35.6 km (22.1 mi) S of SR 14 via Red River Rd, SF Red River
A. quattuor	CHR2180.0<br>CHR2180.1<br>CHR2180.2<br>CHR2180.3<br>CHR2180.4<br>OP2270<br>OP2271<br>OP2272<br>OP2273<br>OP2274	M<br>M<br>M<br>F<br>F<br>F<br>F<br>F<br>F<br>F	9-Jul-08	45.6099, -116.0300	1675	*Abies grandis*; stream-side woody debris	ID, Idaho Co, Nez Perce Nat For, FS Rd 221 4.5 km (2.8 mi) S FS Rd 354, tributary of Little Slate Creek
A. sheari	AMNH<br>AMNH<br>CASENT9039225<br>UWBM ID0015/5359	M<br>F<br>F<br>F	19-Jun-09	45.3902, -115.9877	1788	*Abies grandis*, *Pseudotsuga menziesii*; stream-side woody debris	ID, Idaho Co, Payette Nat For, Burgdorf Rd 20.0 km (12.4 mi) NW of Warren Wagon Rd, tributary of Fall Creek
A. sheari	CASENT9039217<br>UWBM ID0016/5360<br>OP2708	M<br>M<br>M	19-Jun-09	45.3723, -116.0220	1504	*Abies grandis*, *Picea engelmannii*; stream-side woody debris	ID, Idaho Co, Payette Nat For, Burgdorf Rd 26.7 km (16.6 mi) NW of Warren Wagon Rd, tributary of Fall Creek
A. sheari	OP2720	F	5-Sep-09	45.3822, -115.9779	1730	*Abies grandis*, *Pseudotsuga menziesii*, *Rubus parviflorus*; stream-side woody debris	ID, Idaho Co, Payette Nat For, FS 592 1.1 km (0.7 mi) E of FS 246, tributary of Fall Creek
A. merickeli	OP2236	F	6-Jul-08	46.0433, -115.2954	560	*Thuja plicata*, *Pseudotsuga menziesii*; stream-side woody debris	ID, Idaho Co, Nez Perce Nat For, FS 443 1.0 km (0.6 mi) S of Selway River Rd, tributary of Meadow Creek

(table continues)

Table A.1 Collecting Locality Information (continued).

Taxon	Voucher Number	Sex	Date	Latitude, Longitude°	Elev. (m)	Habitat; Microhabitat	Locality
A. merickeli	CHR2100.0	M	6-Jul-08	46.0385, -115.2943	535	spring, stream-side woody debris	ID, Idaho Co, Nez Perce Nat For, FS 443 1.3 km (0.8 mi) south of Selway River Road, tributary of Meadow Creek
	CHR2100.1	M					
	CHR2100.2	F					
	OP2237	F					
	OP2238	F					
	OP2239	M					
	OP2240	M					
	OP2241	F					
A. merickeli	CHR2121.0	M	7-Jul-08	46.0559, -115.5195	590	*Thuja plicata*, *Pseudotsuga menziesii*; moss, stream-side woody debris	ID, Idaho Co, Nez Perce Nat For, FS 443 4.2 km (2.6 mi) S of FS 223, tributary of O'Hara Creek
	CHR2121.1	M					
	CHR2121.2	M					
	CHR2121.3	M					
	CHR2121.4	F					
	CHR2121.5	F					
	CHR2121.6	F					
	OP2245	M					
	OP2246	M					
	OP2247	M					
	OP2248	F					
	OP2249	F					
A. merickeli	CHR2161.0	M	8-Jul-08	45.8437, -115.6178	1156	*Picea engelmannii*; stream-side woody debris	ID, Idaho Co, Nez Perce Nat For, FS 1858 2.7 km (1.7 mi) N of SR 14, Newsome Creek
	CHR2161.1	M					
	CHR2161.2	F					
	CHR2161.3	F					
	CHR2161.4	F					
	CHR2161.5	F					
	OP2262	F					
	OP2263	F					
	OP2264	F					
	OP2265	F					

(table continues)

Table A.1 Collecting Locality Information (continued).

Taxon	Voucher Number	Sex	Date	Latitude, Longitude°	Elev. (m)	Habitat; Microhabitat	Locality
A. merickeli	CHR2140.0	M	7-Jul-08	45.7853, -115.2026	1510	*Picea engelmannii*; moss, stream-side woody debris	ID, Idaho Co, Nez Perce Nat For, Red River Rd 16.4 km (10.2 mi) NE of FS 22, headwaters of Red River
	CHR2140.1	M					
	CHR2140.2	F					
	OP2251	M					
	OP2252	M					
	OP2253	M					
	OP2254	M					
A. merickeli	OP2250	M	7-Jul-08	45.7939, -115.4006	1270	*Picea engelmannii*; side-slope seep, woody debris	ID, Idaho Co, Nez Perce Nat For, FS 22 0.2 km (0.1 mi) SE of FS 1818, Red Horse Creek
A. cf. *quattuor*	CHR2070.1	M	5-Jul-08	46.5389, -114.6762	1100	Old growth *Thuja plicata*; seep-like riparian, woody debris	ID, Idaho Co, Clearwater Nat For, DeVoto Memorial Cedar Grove, US 12 15.3 km (9.5 mi) west of Lolo Pass
	CHR2070.2	F					
	CHR2070.3	M					
	CHR2070.4	M					
	CHR2070.5	F					
	CHR2070.6	M					
	CHR2070.7	M					
	CHR2070.8	M					
	CHR2070.9	M					
	OP2230	M					
	OP2231	M					
	OP2232	M					
	CHR2445.0	F	30-Jul-08				
	CHR2445.1	F					
	CHR2445.2	F					
A. cf. *quattuor*	OP2287	F	17-Jul-08	46.1025, -115.5555	470	*Thuja plicata*, *Abies grandis*; stream-side woody debris	ID, Idaho Co, Nez Perce Nat For, Selway River Road 6.6 km (4.1 mi) southeast of US 12, tributary of Selway River
A. cf. *quattuor*	OP2342	F	31-Jul-08	46.4678, -114.9854	870	*Thuja plicata*, *Abies grandis*; stream-side woody debris	ID, Idaho Co, Clearwater Nat For, US 12 48.1 km (29.9 mi) west of Lolo Pass, tributary of Post Office Creek

(table continues)

Table A.1 Collecting Locality Information (continued).

Taxon	Voucher Number	Sex	Date	Latitude, Longitude°	Elev. (m)	Habitat; Microhabitat	Locality
A. cf. *quattuor*	OP2283	F	17-Jul-08	46.0848, -115.4955	480	*Thuja plicata*, *Abies grandis*; stream-side woody debris	ID, Idaho Co, Nez Perce Nat For, Selway River Rd 13.0 km (8.1 mi) southeast of US 12, tributary of Selway River
A. cf. *quattuor*	CHR2192.0<br>CHR2192.1<br>CHR2192.2<br>CHR2192.3<br>CHR2192.4<br>CHR2192.5<br>OP2275<br>OP2276<br>OP2277	M<br>M<br>M<br>M<br>M<br>F<br>M<br>M<br>M	16-Jul-08	46.2311, -115.4161	536	*Abies grandis*, *Thuja plicata*; stream-side woody debris	ID, Idaho Co, Nez Perce Nat For, Split Ck Trail, US 12 23.5 km (14.6 mi) E of Selway River Rd, tributary of Lochsa River
A. cf. *quattuor*	CHR2227.0<br>CHR2227.1<br>CHR2227.2<br>OP2284<br>OP2285<br>OP2286	M<br>M<br>F<br>F<br>M<br>M	17-Jul-08	46.0498, -115.3013	545	*Thuja plicata*, *Abies grandis*; stream-side woody debris	ID, Idaho Co, Nez Perce Nat For, Selway River Rd 30.0 km (18.8 mi) SE of US 12, tributary of Selway River
A. cf. *quattuor*	CHR2076.0<br>CHR2076.1<br>CHR2076.2<br>OP2233<br>OP2234<br>OP2235	F<br>F<br>F<br>F<br>F<br>F	5-Jul-08	46.4292, -115.1335	813	*Thuja plicata*, *Pseudotsuga menziesii*; seep-like riparian adjacent Lochsa River, woody debris	ID, Idaho Co, Clearwater Nat For, Eagle Mt Trail, US 12 46.9 km (40.3 mi) W of Lolo Pass, Lochsa River
"*A.cosmetoides*"	CHR2207.0<br>CHR2207.1<br>OP2281<br>OP2282	M<br>F<br>F<br>F	16-Jul-08	46.1396, -115.6660	465	*Thuja plicata*, *Abies grandis*; stream-side woody debris	ID, Idaho Co, Clearwater Nat For, US 12 5.8 km (3.6 mi) west of Selway River Road, Two Shadows Creek
"*A.cosmetoides*"	OP2298	F	19-Jul-08	46.5105, -115.6941	1342	*Picea engelmannii*; stream-side woody debris	ID, Clearwater Co, Clearwater Nat For, FS 250 17.1 km (10.6 mi) N of SR 11, China Gulch

(table continues)

Table A.1 Collecting Locality Information (continued).

Taxon	Voucher Number	Sex	Date	Latitude, Longitude°	Elev. (m)	Habitat; Microhabitat	Locality
"*A.cosmetoides*"	CHR2200.0	M	16-Jul-08	46.2101, -115.5442	490	*Thuja plicata*, *Abies grandis*; stream-side woody debris	ID, Idaho Co, Clearwater Nat For, Canyon Ck Trail, US 12 11.3 km (7.0 mi) E of Selway River Rd, tributary of Lochsa River
	CHR2200.1	M					
	CHR2200.2	M					
	CHR2200.3	M					
	CHR2200.4	M					
	CHR2200.5	F					
	OP2278	M					
	OP2279	M					
	OP2280	F					
"*A.cosmetoides*"	CHR2248.0	M	17-Jul-08	46.2395, -115.7691	965	*Thuja plicata*, *Abies grandis*; stream-side woody debris	ID, Idaho Co, Clearwater Nat For, FS 519 1.0 km (0.6 mi) SE of FS 100, tributary of Yakus Creek
	CHR2248.1	M					
	CHR2248.2	M					
	CHR2248.3	F					
	CHR2248.4	F					
	CHR2248.5	F					
	OP2288	M					
	OP2289	M					
	OP2290	M					
	OP2291	F					
	OP2292	F					
"*A.cosmetoides*" 2.0 km SE of Pierce; TypeLocality (Shear 1986)	CHR2264.0	M	19-Jul-08	46.4767, -115.7809	960	*Abies grandis*; stream-side woody debris	ID, Clearwater Co, Clearwater Nat For, FS 250 3.2 km (2.0 mi) SE of SR 11, tributary of Rhodes Creek
	CHR2264.1	M					
	CHR2264.2	M					
	CHR2264.3	F					
	OP2296	M					
	OP2297	F					
	OP2360	F					
"*A.cosmetoides*"	OP2302	M	20-Jul-08	46.8776, -115.0889	1140	*Picea engelmannii*; stream-side woody debris	ID, Clearwater Co, Clearwater National Forest, Forest Service Road 720 1.3 km (0.8 mi) north of Forest Service Road 250, tributary of North Fork Clearwater River
	OP2303	F					

(table continues)

Table A.1 Collecting Locality Information (continued).

Taxon	Voucher Number	Sex	Date	Latitude, Longitude°	Elev. (m)	Habitat; Microhabitat	Locality
"*A.cosmetoides*"	CHR2309.0	M	20-Jul-08	46.7192, -115.2317	865	*Thuja plicata*, *Abies grandis*; stream-side woody debris	ID, Clearwater Co, Clearwater Nat For, FS 255 2.7 km (1.7 mi) E FS 250, tributary of Kelly Creek
	CHR2309.1	M					
	CHR2309.2	F					
	CHR2309.3	F					
	OP2304	F					
	OP2305	M					
	OP2306	F					
"*A.cosmetoides*"	CHR2312.0	M	21-Jul-08	46.7331, -115.3111	944	*Thuja plicata*, *Abies grandis*; stream-side woody debris	ID, Clearwater Co, Clearwater Nat For, FS 711 1.9 km (1.2 mi) N of FS 250, tributary of Cold Springs Creek
	CHR2312.1	M					
	CHR2312.2	M					
	CHR2312.3	F					
	CHR2312.4	F					
	OP2307	M					
	OP2308	M					
	OP2309	M					
	OP2310	F					
"*A.cosmetoides*"	CHR2348.0	M	24-Jul-08	47.0352, -116.7896	995	*Abies grandis*, *Thuja plicata*; stream-side woody debris	ID, Benewah Co, Idaho Panhandle Nat For, Meadow Creek Road 13.8 km (8.6 mi) northwest of SR 6, Meadow Creek
	CHR2348.1	M					
	CHR2348.2	F					
	OP2319	M					
	OP2320	M					
	OP2321	M					
	OP2322	F					
"*A.cosmetoides*"	CHR2403	F	27-Jul-08	47.2122, -115.5484	897	*Thuja plicata*; stream-side woody debris	ID, Shoshone Co, Idaho Panhandle National Forest, FS Rd 1214 0.2 km (0.1 mi) N of FS Rd 50, Eagle Creek
"*A.cosmetoides*" *A. shoshone* type (Shear 1986)	OP1648	F	7-Jul-07	47.0861, -116.1129	1295	Old-growth *Thuja plicata*, *Taxus brevifolia*; seep-springs, stream-side woody debris	ID, Shoshone Co, Idaho Panhandle Nat For, Hobo Cedar Grove Botanical Area
	CHR2359.0	M	25-Jul-08				
	CHR2359.1	F					
	OP2323	F					
	OP2324	F					

(table continues)

Table A.1 Collecting Locality Information (continued).

Taxon	Voucher Number	Sex	Date	Latitude, Longitude°	Elev. (m)	Habitat; Microhabitat	Locality
"*A.cosmetoides*"	CHR2368.0<br>CHR2368.1<br>CHR2368.2<br>CHR2368.3<br>OP2325<br>OP2326<br>OP2327	M<br>M<br>F<br>F<br>M<br>M<br>F	26-Jul-08	47.2436, -116.0500	675	*Thuja plicata*, *Abies grandis*, *Tsuga heterophylla*; stream-side woody debris	ID, Shoshone Co, Idaho Panhandle National Forest, FS Rd 50 at Pocono Creek
"*A.cosmetoides*"	CHR2379.0<br>CHR2379.1<br>CHR2379.2<br>OP2328<br>OP2329	M<br>F<br>F<br>F<br>F	26-Jul-08	47.2236, -115.6072	860	*Thuja plicata*; stream-side woody debris	ID, Shoshone Co, Idaho Panhandle Nat For, FS Rd 752 0.2 km (0.1 mi) south of FS 50, Prospector Creek
"*A.cosmetoides*"	OP2334	F	27-Jul-08	47.2296, -115.5406	977	*Thuja plicata*, *Abies grandis*; stream-side woody debris	ID, Shoshone Co, Idaho Panhandle Nat For, FS 1214 2.3 km (1.4 mi) N of FS 50, tributary of Eagle Creek
"*A.cosmetoides*"	CHR2414.0<br>CHR2414.1<br>CHR2414.2<br>CHR2414.3<br>CHR2414.4<br>OP2335<br>OP2336<br>OP2337<br>OP2338<br>OP2339	M<br>M<br>M<br>M<br>F<br>M<br>M<br>M<br>M<br>F	28-Jul-08	47.1296, -115.8819	1269	*Thuja plicata*, *Abies grandis*; stream-side woody debris	ID, Shoshone Co, Idaho Panhandle Nat For, FS 201 0.2 km (0.1 mi) E of FS 301, tributary of Fishhook Creek
"*A.cosmetoides*"	OP2340	F	29-Jul-08	47.4304, -115.8913	1110	*Thuja plicata*, *Abies grandis*, *Tsuga heterophylla*; stream-side woody debris	ID, Shoshone Co, Placer Creek Road 7.4 km (4.6 mi) southeast of High Street via King Sttree in Wallace, tributary of Placer Creek
"*A.cosmetoides*"	OP2315	F	21-Jul-08	46.8093, -115.6156	940	*Thuja plicata*, *Abies grandis*; stream-side woody debris	ID, Clearwater Co, Clearwater National Forest, FS Rd 246 3.2 km (2.0 mi) east of FS 247, Sourdough Creek

(table continues)

Table A.1 Collecting Locality Information (continued).

Taxon	Voucher Number	Sex	Date	Latitude, Longitude°	Elev. (m)	Habitat; Microhabitat	Locality
"*A.cosmetoides*"	OP2311<br>OP2312<br>OP2313<br>OP2314	F<br>F<br>F<br>F	21-Jul-08	46.7395, -115.5444	645	*Thuja plicata*, *Abies grandis*; stream-side woody debris	ID, Clearwater Co, Clearwater National Forest, FS Rd 247 15.8 km (9.8 mi) NW of FS Rd 250, tributary of North Fork Clearwater River
"*A.cosmetoides*"	OP2316	F	23-Jul-08	46.9873, -116.4199	985	*Thuja plicata*, *Abies grandis*; stream-side woody debris	ID, Latah Co, Idaho Panhandle Nat For, Emerald Creek Rd 15.6 km (9.7 mi) west of SR 3 via FS Rd 504, East Fork Emerald Creek
"*A.cosmetoides*"	OP2330<br>OP2331	F<br>M	26-Jul-08	47.2834, -115.7738	834	*Thuja plicata*; stream-side woody debris	ID, Shoshone Co, Idaho Panhandle Nat For, North Fork St Joe River Rd 5.5 km (3.4 mi) N of FS 50,tributary of North Fork St Joe River
"*A.cosmetoides*"	OP2332<br>OP2333	M<br>F	27-Jul-08	47.0802, -115.3548	1096	*Picea engelmannii*; stream-side woody debris	ID, Shoshone Co, Idaho Panhandle Nat For, FS Rd 218 12.4 km (7.7 mi) southeast of FS Rd 50, tributary of St Joe River
"*A.cosmetoides*"	CHR2341.0<br>CHR2341.1<br>CHR2341.2<br>OP2317<br>OP2318	M<br>M<br>F<br>M<br>M	24-Jul-08	47.0351, -116.6725	926	*Thuja plicata*, *Abies grandis*, *Tsuga heterophylla*, *Alnus sitchensis*; stream-side woody debris	ID, Latah Co, Idaho Panhandle Nat For, SE of intersect of FS 1443 and SR 6, tributary of Mannering Creek
	CHR1409<br>CHR1410<br>CHR1411<br>OP1645	M<br>F<br>F<br>F	7-Jul-07				
"*A.cosmetoides*"	CHR2280.0<br>CHR2280.1<br>CHR2280.2<br>OP2299<br>OP2300<br>OP2301	F<br>F<br>F<br>F<br>F<br>F	19-Jul-08	46.5836, -115.6164	968	*Thuja plicata*; stream-side woody debris	ID, Clearwater Co, Clearwater Nat For, FS 250 19.0 mi N of SR 11, tributary of Orogrande Ck

(table continues)

Table A.1 Collecting Locality Information (continued).

Taxon	Voucher Number	Sex	Date	Latitude, Longitude°	Elev. (m)	Habitat; Microhabitat	Locality
"*A.cosmetoides*"	OP2293<br>OP2294<br>OP2295	M<br>F<br>F	18-Jul-08	46.3721, -115.7235	1000	*Thuja plicata*, *Abies grandis*; stream-side woody debris	ID, Clearwater Co, Clearwater Nat For, FS 103 3.5 km (2.2 mi) NE of FS 100, tributary of Lolo Creek
"*A.cosmetoides*"	OP1634	F	13-May-06	47.0377, -116.6735	900	*Thuja plicata*, *Abies grandis*; stream-side woody debris	ID, Latah Co, Idaho Panhandle Nat For, SR 6 at the Benewah County Line, Mannering Creek
"*A.cosmetoides*"	OP2341	M	30-Jul-08	47.2279, -115.2464	1305	*Thuja plicata*, *Abies grandis*, *Picea engelmannii*; stream-side woody debris	MT, Mineral Co, Lolo National Forest, FS Rd 282 15.6 km (9.7 mi) south of Mullan Gulch Road, Goose Creek

Appendix A. *A.*: *Acuclavella*. Collections: OP: SDSU Opiliones molecular collection, CHR: personal collection of author, AMNH: American Museum of Natural History, CAS: California Academy of Sciences, UWBM: University of Washington Burke Museum, NCSM: North Carolina Museum of Natural Sciences. For each entry the taxon name, voucher number, sex, date, coordinates in decimal degrees, elevation in meters, habitat and microhabitat, and locality information is provided.
