# Supplementary material for: Three new species in the harvestmen genus Acuclavella (Opiliones, Dyspnoi, Ischyropsalidoidea), including description of male Acuclavella quattuor Shear, 1986
Source: Zookeys. 2013 Jun 20;(311):19–68. doi: 10.3897/zookeys.311.2920 (PMC3698555; doi:10.3897/zookeys.311.2920)
Supplement: Supplementary file 8 — PCR Primer Information. (doi: 10.3897/zookeys.311.2920.app2) File format: Adobe PDF file (pdf). [file ZooKeys-311-019-s002.pdf]

Supplemental File B. Primer information.					
Gene	Direction	Name	Authors	Length	Sequence
28S	Forward	ZX1	van der Auwera et al. 1994	21	5'- ACCCGCTGAATTTAAGCATAT -3'
	Reverse	ZR2	Waeschenbach et al. 2007	22	5'- GCTATCCTGAGGGAAACTTCGG -3'
EF-1α	Forward	EF1-OP2ASAB	Hedin et al. 2010	23	5'- GCTGTGCTTATTGTTGCTGCYGG -3'
	Forward	EF1-OP2BSAB	Hedin et al. 2010	23	5'- GGTACTGGTGAGTTTGAAGCTGG -3'
	Reverse	EF1-OPRC4	Hedin et al. 2010	21	5'- GAACTTGCANGCAATGTGAGC -3'
COI	Forward	PMT1	Folmer et al. 1994	25	5'- GGTCAACAAATCATAAAGATATTGG -3'
	Forward	C1-J-1517Spid	Thomas & Hedin 2008	20	5'- AATCATARGGATATTGGAAC -3'
	Forward	1718SPID	Vink et al. 2005	24	5'- GGNGGATTTGGAAATTGRTTRGTT -3'
	Forward	1652_Ac	Newly designed	20	5'- GTCTTAGTGACWGCCCATGC -3'
	Reverse	2776S	Vink et al. 2005	23	5'- GGATAATCAGAATANCGNCGAGG -3'
	Reverse	2500_Ac	Newly designed	20	5'- GAGGACATAGTGAAAGTGAG -3'
WNT2	Forward	MYWNT6FOR	Satler et al. 2011	20	5'- TGCAARTGCCACGGNATGTC -3'
	Reverse	MYWNT2REV	Satler et al. 2011	22	5'- ACYTGGCARCACCARTGAAAYG -3'
Ambiguity codes are standard. Vink et al. 2005 primers modified from Simons et al. 1994					

Literature Cited

Folmer O, Black M, Hoeh W, Lutz R, Vrijenhoek R (1994) DNA Primersfor Amplification of Mitochondrial Cytochrome C Oxidase Subunit 1 from Diverse Metazoan Invertebrates. Molecular Marine Biology and Biotechnology 3(5): 294-299.

Hedin M, Derkarabetian S, McCormack M, Richart C, Shultz JW (2010) The Phylogenetic Utility of the Nuclear Protein-Coding Gene EF-1α for Resolving Recent Divergences in Opiliones, Emphasizing Intron Evolution. Journal of Arachnology 38: 9-20.

Satler JD, Starrett J, Hayashi CY, Hedin M (2011) Inferring Species Trees from Gene Trees in a Radiation of California Trapdoor Spiders (Araneae, Antrodiaetidae, Aliatypus). PLoS ONE 6(9): e25355.

Simons C, Frati F, Beckenback A, Crespi B, Liu H, Floors P (1994) Evolution, Weighting, and Phylogenetic Utility of Mitochondrial Gene Sequences and a Compilation of Conserved Polymerase Chain Reaction Primers. Annals of the Entomological Society of America 87(6): 651-701.

Thomas SM, Hedin M (2008) Multigenic Phylogeographic Divergence in the Paleoendemic Southern Appalachian Opilionid *Fumontana deprehendor* Shear (Opiliones, Laniatores, Triaenonychidae). Molecular Phylogenetics and Evoution 46(2): 645-658.

Van der Auwera G, Chapelle S, De Wächter R (1994) Structure of the Large Ribosomal Subunit RNA of Phytophthora megasperma, and Phylogeny of the omycetes. FEBS Letters 338(2): 133-136.

Vink CJ, Thomas SM, Paquin P, Hayashi CY, Hedin M (2005) The Effects of Preservatives and Temperatures on Arachnid DNA. Invertebrate Systematics 19(2): 99-104.

Waeschenback A, Webster BL, Bray RA, Littlwood DTJ (2007) Added Resolution Among Ordinal Level Relationchips of Tapeworms (Platyhelminthes: Cestoda) with Complete Small and Large Subunit Nuclear Ribosomal RNA Genes. Molecular Phylogenetics and Evolution 45(1): 311-325.
