# Supplementary material for: Three new species in the harvestmen genus Acuclavella (Opiliones, Dyspnoi, Ischyropsalidoidea), including description of male Acuclavella quattuor Shear, 1986
Source: Zookeys. 2013 Jun 20;(311):19–68. doi: 10.3897/zookeys.311.2920 (PMC3698555; doi:10.3897/zookeys.311.2920)
Supplement: Supplementary file 10 — PCA Methods and Results. (doi: 10.3897/zookeys.311.2920.app4) File format: Adobe PDF file (pdf). [file ZooKeys-311-019-s004.pdf]

Richart and Hedin 2013 Supplemental File D – Morphometrics

Methods

Measurements for morphometric analyses were taken using an Olympus SZX12 dissecting microscope with an ocular micrometer. Individuals with missing data (i.e., no Leg II) were not included in analyses. Customary measurements (Acosta et al. 2007) from 16 characters were determined for 261 individuals (131 males, 133 females); the measurements data are available as a supplementary file (<SupplementC_Male&FemaleMorphometricData.xls>). Carapace and scute lengths were taken dorsally at the midline. Carapace measurement was taken from the anterior edge and did not include the forward-projecting ocular spine. Ocularium height was taken from the base of the eye to the tip of the spine. Metapeltidial sensory cones and scutal spines or median tubercles were measured as the vertical distance from the sagittal cleft to the tip of the tubercle in lateral orientation. The distance from the eye spine tip to the area II spine or tubercle tip was taken in an effort to capture the variability of the anteriad projection of eye spines and posteriad curve of scutal spines. The length of the palpal femur and leg II trochanter, femur, patella, tibia, metatarsus, and tarsus were measured linearly between the distalmost points on either side of the segment. Total body length was not taken due to the variation in length allowed by free tergites and sternites in individuals that are gravid or have recently eaten; this measurement is known to vary with nutritional condition in Opiliones (Acosta and Machado 2007).

Traditionally, morphological delimitations of species assume that morphological gaps exist between species (Cracraft 1987, de Queiroz 2007). Morphometric analyses were conducted to infer if two or more morphological groups are a better way of describing the sampled data than a single group. Two types of morphometric analyses were utilized: principle components analysis (PCA) and discriminant function analysis (DFA); these were carried out using Systat 12 (Systat Software, Inc.). Though both PCA and DFA are multivariate analyses, DFA deals specifically with the problem of separating predetermined groups. The discriminant functions maximize the ratio of inter- and intra-group variance. Canonically, DFA is able to measure to which group a specimen has the highest classification scores. In taxonomy, DFA analyses are nearly exclusively used to differentiate between two species

that are morphologically similar and difficult to identify from a single characteristic (see Klimov et al. 2004, Seifert 2003, Fisher 1936, Lubishew 1962). Multispecies delimitation via DFA, as done here, is uncommon (see Schlick-Steiner et al. 2006).

In taxonomy, PCAs are regularly employed for species delimitation. Plotting two principle components from a PCA illustrates the variability in the data set; it is naïve to group assignment. An implicit assumption of this analysis is that distinguishing traits are present in the data set. Individuals within species are expected to cluster in morphospace with sparsely inhabited or empty space between them (Ezard et al. 2010). This utilization has ranged from comparing the vector angles in a scatter plot of predefined species groups (Hamm 2010), to mathematical inference of groups without *a priori* assignment of samples to species (Ezard et al. 2010). Because a PCA has no intrinsic measure of group exclusivity, the parameters of recovering a group in morphospace via PCA need to be defined. For purposes of this research we defined three PCA categories: a "recovered" group clustered together and did not overlap with samples from other groups; a "nearly recovered" group clustered together with minimal overlap (one or two individuals at the periphery of their respective groups, which are not deeply subsumed within another) with other groups; a "not recovered" group failed to cluster exclusively. For these analyses, three methods were employed to explore this morphospace: (1) hypothesized species were compared pairwise; (2) all combinations of principle components explaining a significant part of the variation in the data were explored; and (3) a Bayesian approach where the posterior becomes the next prior, if clusters of hypothesized species are recovered in initial analyses, further analyses can occur on those clusters.

In this study, an attempt is made to distinguish seven *a priori* defined species; some appear similar morphologically and others show obvious morphological differences. Three apparently new strategies are employed to reveal variation in the substructure of the data: 1) pairwise PCA, 2) nested PCA, and 3) exhaustive components PCA. Pairwise PCA directly compares each hypothesized species group on a one-to-one basis. Nested PCA assesses the substructure of groups recovered during complete data set analyses. If PCAs cluster *a priori* hypothesized species or clusters of such species, as distinct from other *a priori* hypothesized

species, or clusters of such species, then an additional "nested" PCA was conducted on recovered clusters. Nested PCAs were continued until further clusters of *a priori* defined species or clusters of species were no longer recovered. Lastly, the exhaustive components PCA compares all principle components with an eigenvalue greater than one (Norman and Streiner 1994) for analyses conducted on correlation matrices, and the first four principle components returned on analyses based on covariance matrices. In these analyses, bi-dimensional graphs illustrate the structure seen using all component combinations. For example, component 1 and 2, 1 and 3, 1 and 4, 2 and 3, 2 and 4, and 3 and 4 are all used to visualize the morphological structure of the data. DFAs were employed in order to test the hypothesis of no difference between hypothesized species.

Due to sexual dimorphism, male and female specimens were analyzed separately for all morphometric analyses. Since the data used to conduct PCAs were measured in the same units (mm), analyses were conducted on both correlation and covariance matrices. PCAs using covariance matrices tend to be dominated by characters showing the most variability (Jolliffe 2002). In order to reduce possible excessive influence on the principle components, variables were coded to have a mean of zero and variance of one; analyses on this data are based on a correlation matrix. Analyses run on covariance matrices assume a multivariate normal distribution. This is often violated in taxonomic studies where data consists of multiple species of varying morphological distinctiveness; outliers could represent members of a relatively under-sampled group. All unusual measurements were confirmed by repeated measurement, then included in analyses under the assumption they do not excessively affect interpretation of the data.

Species Delimitation

In order for a group to be defined as morphologically distinct from other groups, the following criteria must be met by any of the four morphometric data sets (male/female; correlation/covariance): 100% of the individuals recovered in a classic DFA, 95% of individuals recovered in jackknifed DFA, and recovered as distinct by nested and pairwise PCAs. Being recovered morphologically, with gaps in morphospace, implies a lack of

genetic exchange. Therefore, predetermined groups separated by gaps are considered different species.

Results

Pairwise PCAs (Figures Supplement D.1-D.21) were conducted for each hypothesized species pair on a covariance and correlation matrix for both male and female data. Group sample sizes, partitioned by hypothesized species and sex, are shown in Supplement Table D.1. Of the three methods employed to elucidate the variation of a large data set with multiple groups, pairwise PCAs were the most successful at recovering *a priori* hypothesized species (Supplement Table D2). All species pairs were discriminated in at least one of the four analyses with the exception of *A. quattuor* from *A.* cf. *quattuor*. Results were similar for correlation and covariance matrices, and males were discriminated more frequently than females. The most successful analyses were found using the male covariance matrix data. In these analyses, all hypothesized species were recovered as discrete except for *A. quattuor* from *A.* cf. *quattuor*. The male correlation data set was very similar to that based on covariance, though the morphospace occupied by *A. sheari* with respect to *A. cosmetoides*, *A. merickeli* with respect to *A. quattuor*, and *A.* cf. *quattuor* with respect to *A. cosmetoides* are not recovered as discretely.

Female pairwise PCA analyses were less successful at resolving *a priori* species groupings. *Acuclavella quattuor* was not separated from *A.* cf. *quattuor* in analyses based on either correlation or covariance matrices. Also, *A. cosmetoides* was not discretely recovered from *A. leonardi*, *A. quattuor*, *A. merickeli*, or *A.* cf. *quattuor*; this result is likely due to high variation within the larger *A. cosmetoides* sample (Supplement Table D.3). Additionally, female data analyzed on a correlation matrix did not distinguish *A. merickeli* from *A.* cf. *quattuor*; and only weakly differentiated *A. leonardi* from *A.* cf. *quattuor*, and *A. merickeli* from *A. quattuor*. Based on a covariance matrix, PCA on the female data set failed to recover *A. leonardi* as morphologically distinct from *A. makah* and *A. merickeli*, and nearly recovered *A. makah* from *A. merickeli*. These results reflect the morphological species hypothesis of Shear (1986) that *A. merickeli* and individuals from Washington are conspecific.

Table Supplement D1 Group Frequencies for PCA and DFA Analyses.

Species	Male	Females	**Total**
A. makah	10	14	24
A. leonardi	6	4	10
A. sheari	4	3	7
A. quattuor	14	17	28
A. merickeli	19	19	38
A. cf. *quattuor*	22	18	40
A. cosmetoides	56	58	114
**Total**	131	133	261

Nested PCAs (Supplement Figures D.22-D.36) on *a priori* hypothesized species groups resolve five of seven hypothesized species of *Acuclavella* as morphologically discrete groups (Supplement Table D2). Analyses of male correlation (Supplement Figures D.22-D.26) and covariance (Supplement Figures D.30-D.34) matrices recovered all hypothesized species as morphologically discrete except for *A. quattuor* from *A.* cf. *quattuor*. However, the levels of nesting by which species were recovered differed. The male correlation matrix (Supplement Figures D.22 recovered *A. sheari*, and two groups of *a priori* defined species: Washington individuals and Clearwater individuals (*A. quattuor*, *A. merickeli*, *A.* cf. *quattuor*, and *A. cosmetoides*). Secondary nesting of Washington individuals (Supplement Figure D.23) recovered *A. makah* discrete from *A. leonardi*, and secondary nesting of Clearwater individuals (Supplement Figure D.24) nearly recovered *A. cosmetoides* from the grouping of *A. quattuor*, *A. merickeli*, and *A.* cf. *quattuor*. Further nesting on these hypothesized species (Supplement Figure D.25) recovered *A. merickeli* as discrete from *A. quattuor* and *A.* cf. *quattuor*. Lastly, analysis of these two species did not recover groups. In the covariance analyses of all males (Supplement Figure D.30), *A. sheari* is recovered, as are two groups of *a priori* hypothesized species: "*Acuclavella merickeli*" of Shear, 1986 (*A. merickeli* of Idaho with Washington samples), and *A. cosmetoides* with *A. quattuor* and *A.* cf. *quattuor*. Analysis of "*A. merickeli*" Shear, 1986 (Supplement Figure D.31) recovered samples from Idaho, and tertiary nesting of Washington males (Supplement Figure D.32) successfully recovered them. A nested PCA on the *A. cosmetoides* with *A. quattuor* and *A.* cf. *quattuor* group (Supplement Figure D.33) recovered *A. cosmetoides*. Once again, *A. quattuor* and *A.* cf. *quattuor* are not recovered when analyzed individually (Supplement Figure D.34).

Table Supplement D2. Comparison of PCA Techniques and Species Recovery.

	A. makah	*A. leonardi*	*A. sheari*	*A. quattuor*	*A. merickeli*	*A.* cf. *quattuor*	*A. cosmetoides*	% Recovered
PairwiseMaleCorrelation	6	6	5.5	4.5	5.5	4.5	5	0.88
PairwiseFemaleCorrelation	6	4.5	6	3.5	3.5	2.5	2	0.67
PairwiseMaleCovariance	6	6	6	5	6	5	6	0.95
PairwiseFemaleCovariance	4.5	3	6	4	3.5	4	2	0.64
NestedMaleCorrelation	X	X	X	-	X	-	X	0.71
NestedFemaleCorrelation	X	X	X	-	-	-	-	0.43
NestedMaleCovariance	X	X	X	-	X	-	X	0.71
NestedFemaleCovariance	-	-	X	-	-	-	-	0.14
ExhaustiveMaleCorrelation	-	-	X	-	~	-	~	0.29
ExhaustiveFemaleCorrealtion	-	-	X	-	-	-	~	0.21
ExhaustiveMaleCovariance	X	X	X	-	X	-	X	0.71
ExhaustiveFemaleCovariance	~	-	X	-	-	-	X	0.36
% Recovered	0.67	0.58	0.96	0.17	0.50	0.17	0.63	

Table D.2 compares PCA methods employed to explore structure in male and female data sets conducted on different association matrices. For pairwise entries, the number corresponds to the number of pairwise comparisons recovered for that hypothesized species; nearly recovered individuals are represented by half-points. X, species recovered; ~, species nearly recovered; -, species not recovered. The % Recovered column represents the percentage of species recovered by that combination of method, data set, and association matrix. The % Recovered row is a measure of how frequently a particular hypothesized species was recovered by analyses. For both of these percentages, "~" were given half points. For pairwise entries, the % Recovered column was calculated by summing the row, then dividing by twice the number of PCAs conducted on that parameter combination; the % Recovered row was calculated by counting "6" entries as "recovered" and non-"6" entries as "nearly recovered". Recovered species were given a full point, nearly recovered a half point, and not recovered zero points. The percent at the bottom is out of twelve possible points; one for each combination of method, data set, and association matrix. Definitions of recovered, nearly recovered, and not recovered can be found in the methods.

As with pairwise analyses, nested PCA performed less well on female data. Female analyses on correlation (Supplement Figure D.27) and covariance (Supplement Figure D.35) matrices recovered *A. sheari* using only the first PCA. The correlation matrix also recovered Washington and Clearwater groups. Nesting on these groups recovered the two Washington species (Supplement Figure D.28), but did not recover the Clearwater species (Supplement Figure D.29). Further analysis of the covariance matrix excluding *A. sheari* (Supplement Figure D.36) did not recover other *a priori* defined species or groups of such species.

Exhaustive components PCA (Supplement Figures D.37-D.64) was the most time-consuming method employed, with each analysis having the full complement of data points. Further, it was the least successful method in recovering hypothesized species of *Acuclavella* of the three methods employed. The eigenvalue equals one rule for correlation matrix analyses (Norman and Streiner 1994) resulted in four principle components for male analyses and five for female analyses (Supplement Figures D.37-D.42 and D.43-D.52 respectively). Though tests of eigenvalue equality exist for deciding which principle components to retain in covariance analyses (Jackson 1991), these were not employed; the variance explained by the largest four principle components was explored.

Bi-dimensional graphs for covariance exhaustive component PCAs are shown in Supplement Figures D.53-D.58 (males), and D.59-D.64 (females). *Acuclavella sheari* was recovered in all analyses; indeed, the single principle component accounting for the greatest variability recovered both sexes on both matrices. Looking at the structure of different components had varying success recovering hypothesized species not already recovered by the first two components. Male correlation analyses: nearly recovered *A. merickeli* and *A. cosmetoides* (Supplement Figures D.40-D.42), Washington samples were recovered from Idaho samples, Clearwater samples were nearly recovered (Supplement Figures D.40, D.41), and the second and third components nearly recovered *A. quattuor* from *A.* cf. *quattuor*. The female correlation matrix had poor results. Only *A. sheari* was recovered. The variation represented by the third and fourth components nearly recovered *A. cosmetoides* (Supplement Figure D.50). Some groups of hypothesized species are seen in these secondary components, but all were recovered by the first two components. The male covariance analyses were most

successful. In this case, looking at the structure seen in secondary components recovered many species not recovered by the first two components. The two components accounting for the most variation in the data set (67.7% combined) recovered *A. sheari* (Supplement Figure D.53); and nearly recovered *A. merickeli*, and samples from Washington, Idaho, and Clearwater groups. However, bi-dimensional representation of second and third components (33.4% of the variation) recovered or nearly recovered every hypothesized species except for the *A. quattuor*-*A.* cf. *quattuor* morphogroup (Supplement Figure D.56), as well as recovering samples from Washington and Idaho. Of the PCAs conducted on a full data set, this is the only one that discriminates all hypothesized species recovered by any PCA analysis. The second and fourth components (25.5%) show largely similar results, although *A. quattuor* and *A.* cf. *quattuor* are found within the morphospace of *A. cosmetoides*. The female covariance matrix performed poorly, though better than the correlation matrix. The first two components recovered *A. sheari* (Supplement Figure D. 59). *Acuclavella cosmetoides* was recovered by plotting the first and third components (Supplement Figure D.60), the second and third components (Supplement Figure D.62), and nearly recovered by components three and four (Supplement Figure D.64). The first and third components also nearly recovered *A. makah*. Washington and Clearwater groups are nearly recovered when represented by the first and fourth, and second and third components (Supplement Figure D.61).

These methods were used to elucidate variation in a data set containing numerous individuals and groups. Generally, pairwise PCA recovered hypothesized species with most regularity (Supplement Table D.2), followed by nested PCAs, then exhaustive components PCAs. Exhaustive component analyses outperformed nested analysis with the female covariance matrix, and performed equally well on the male covariance matrix. A problem can arise in taxonomic application of morphometric when the intraspecific range of morphological variation exceeds the range of interspecies variation (Ezard et al. 2010). Results from pairwise analysis may have been higher if not for situations were one hypothesized species is swamped by the range of variation within another. This problem is likely exacerbated when species occupying a smaller range of morphological variation is compared to a morphologically diverse species with a large sample size. I believe this happened frequently

within *A. cosmetoides*. Thus, it is important to look at complete data set analyses and an attempt should be made to analyze approximately equivalent sample sizes. Pairwise analyses on covariance matrices outperformed their correlation counterpart for both sexes. Though uncommon in practice, these results show that taxonomists should consider exploring their multivariate data sets with covariance matrices when measurements are in the same units. This may result in characters with large variation dominating principle components, but this variation may have biological relevance. Interestingly, males were recovered more frequently than females for all three methods and both association matrices.

The results of PCA show that *A. sheari* is morphologically the most distinct species; both sexes were recovered using both matrices in all three types of analyses with the exception of the male correlation pairwise PCA with *A. cosmetoides*, in which it was nearly recovered. *Acuclavella makah* was recovered in the majority of analyses, especially well in nested and pairwise PCAs. Male *A. leonardi* were recovered by all covariance methods, and by all correlation methods excluding exhaustive components PCA. Female *A. leonardi* were successfully recovered in most analyses, but not recovered by exhaustive components PCAs. Male *A. cosmetoides* were recovered or nearly so in all analyses on both matrices. This highly-variable group was the only hypothesized species where exhaustive component analyses outperformed the other two methods; specifically, for female data sets, though male data sets performed nearly as well. Both male matrices recovered or nearly recovered *A. merickeli* in all methods. Females of this group were recovered in about half of pairwise analyses, but not recovered by the other two methods. Nested PCAs and exhaustive components analyses failed to recover *A. quattuor* from *A.* cf. *quattuor*. In all analyses they were clustered together, though often recovered from other hypothesized species. Pairwise analyses were able to differentiate the morphospace jointly occupied by this species pair in nearly all male analyses on both association matrices; females were recovered less frequently.

All characters and individuals used for PCA were also used for DFA. In cases where within group and between group variance does not have the same dominant direction, discarding low variance characters can result in discarding most of the information concerning

intergroup variation (Jolliffe 2002). Additionally, DFA are almost exclusively used to discriminate between two taxa (though see Schlick-Steiner et al. 2006); since these analyses attempted to discriminate seven hypothesized species, all recorded variables were used. Male *Acuclavella* are robustly discriminated; a classification matrix is shown in Supplement Table D.3. Discriminant function analysis discriminated all species with 100% accuracy with the exception of *A.* cf. *quattuor*, which on two occasions was classified as *A. quattuor* for a classification error rate of 0.09. The same analysis was run with a jackknife classification (Supplement Table D.4) where each individual is allocated to a group without using that individual to determine the center of its assigned group. This analysis discriminates 94% of all individuals. Since group frequencies varied greatly (Supplement Table D.1), analyses were rerun allowing for prior probabilities of group membership. This analysis resulted in very similar results (not shown): males in the jackknife classification had an additional *A.* cf. *quattuor* misclassified as an *A. quattuor*, and an additional *A. quattuor* correctly classified. Similar to PCA, females were not recovered as frequently as males (Supplement Table D.5; jackknife classification Supplement Table D.8). *Acuclavella leonardi*, *A. merickeli*, and *A. sheari* were correctly discriminated in the classification matrix. *A. makah* and *A. cosmetoides* were well-supported, with correct classification 93% and 97% of the time respectively. About 25% of *A. quattuor* and *A.* cf. *quattuor* were misclassified as the other species. Results from the jackknifed classification matrix show similar, if slightly lower percent of correctly classified individuals. In this matrix, an *A. sheari* is misclassified as an *A. cosmetoides*, and three *A. cosmetoides* are misclassified as *A. sheari*. This is likely the result of convergent similarities – females of *A. sheari* and some *A. cosmetoides* lack scutal spines. Again, to account for the large discrepancy in group frequency in the data set, the analysis was rerun to allow for prior probabilities of group membership. The results (not shown) were largely similar with two exceptions: discrimination of female *A. cosmetoides* was enhanced with fewer individuals classified as *A. sheari*, whereas female *A. leonardi* were misclassified 50% of the time, with one classified as *A. makah*, and one classified as *A. merickeli*.

Table Supplement D.3 Discriminant Function Analysis – Male Classification Matrix.

	A. makah	*A. leonardi*	*A.* cf. *quattuor*	*A. cosmetoides*	*A. merickeli*	*A. quattuor*	*A. sheari*	%
A. makah	10	0	0	0	0	0	0	100
A. leonardi	0	6	0	0	0	0	0	100
A. cf. *quattuor*	0	0	20	0	0	2	0	91
A. cosmetoides	0	0	0	56	0	0	0	100
A. merickeli	0	0	0	0	19	0	0	100
A. quattuor	0	0	0	0	0	14	0	100
A. sheari	0	0	0	0	0	0	4	100
Total	10	6	20	56	19	16	4	98

Table Supplement D.4 Discriminant Function Analysis – Male Jackknife Classification Matrix.

	A. makah	*A. leonardi*	*A.* cf. *quattuor*	*A. cosmetoides*	*A. merickeli*	*A. quattuor*	*A. sheari*	%
A. makah	10	0	0	0	0	0	0	100
A. leonardi	1	5	0	0	0	0	0	83
A. cf. *quattuor*	0	0	18	0	0	4	0	82
A. cosmetoides	0	0	0	56	0	0	0	100
A. merickeli	0	0	0	0	19	0	0	100
A. quattuor	0	0	3	0	0	11	0	79
A. sheari	0	0	0	0	0	0	4	100
Total	11	5	21	56	19	15	4	94

Table Supplement D.5 Discriminant Function Analysis – Female Classification Matrix.

	A. makah	*A. leonardi*	*A.* cf. *quattuor*	*A. cosmetoides*	*A. merickeli*	*A. quattuor*	*A. sheari*	%
A. makah	13	1	0	0	0	0	0	93
A. leonardi	0	4	0	0	0	0	0	100
A. cf. *quattuor*	0	0	14	0	0	4	0	78
A. cosmetoides	0	0	1	56	0	0	1	97
A. merickeli	0	0	0	0	19	0	0	100
A. quattuor	0	0	4	0	0	13	0	76
A. sheari	0	0	0	0	0	0	3	100
Total	13	5	19	56	19	17	4	92

Table Supplement D.6 Discriminant Function Analysis – Female Jackknife Classification Matrix.

	A. makah	*A. leonardi*	*A.* cf. *quattuor*	*A. cosmetoides*	*A. merickeli*	*A. quattuor*	*A. sheari*	%
A. makah	13	1	0	0	0	0	0	93
A. leonardi	1	2	0	0	1	0	0	50
A. cf. *quattuor*	0	0	13	0	0	5	0	72
A. cosmetoides	0	0	1	54	0	0	3	93
A. merickeli	0	1	0	0	18	0	0	95
A. quattuor	0	0	7	0	0	10	0	59
A. sheari	0	0	0	1	0	0	2	67
Total	14	4	21	55	19	15	5	84

Parameters defined in order to recover entities as discrete (recovered in 100% of DFA, 95% of jackknifed DFA, pairwise PCA, and nested PCA for any sex-matrix combination) recovers *A. makah*, *A. sheari*, *A. merickeli*, and *A. cosmetoides*. *Acuclavella leonardi* meets all criteria except for being misclassified as an *A. makah* in 17% of jackknifed DFA (Supplement Table D.6; n=1). Samples representing *A. quattuor* and *A.* cf. *quattuor* were not recovered as discrete by these parameters.

PRINCIPLE COMPONENTS ANALYSES

14

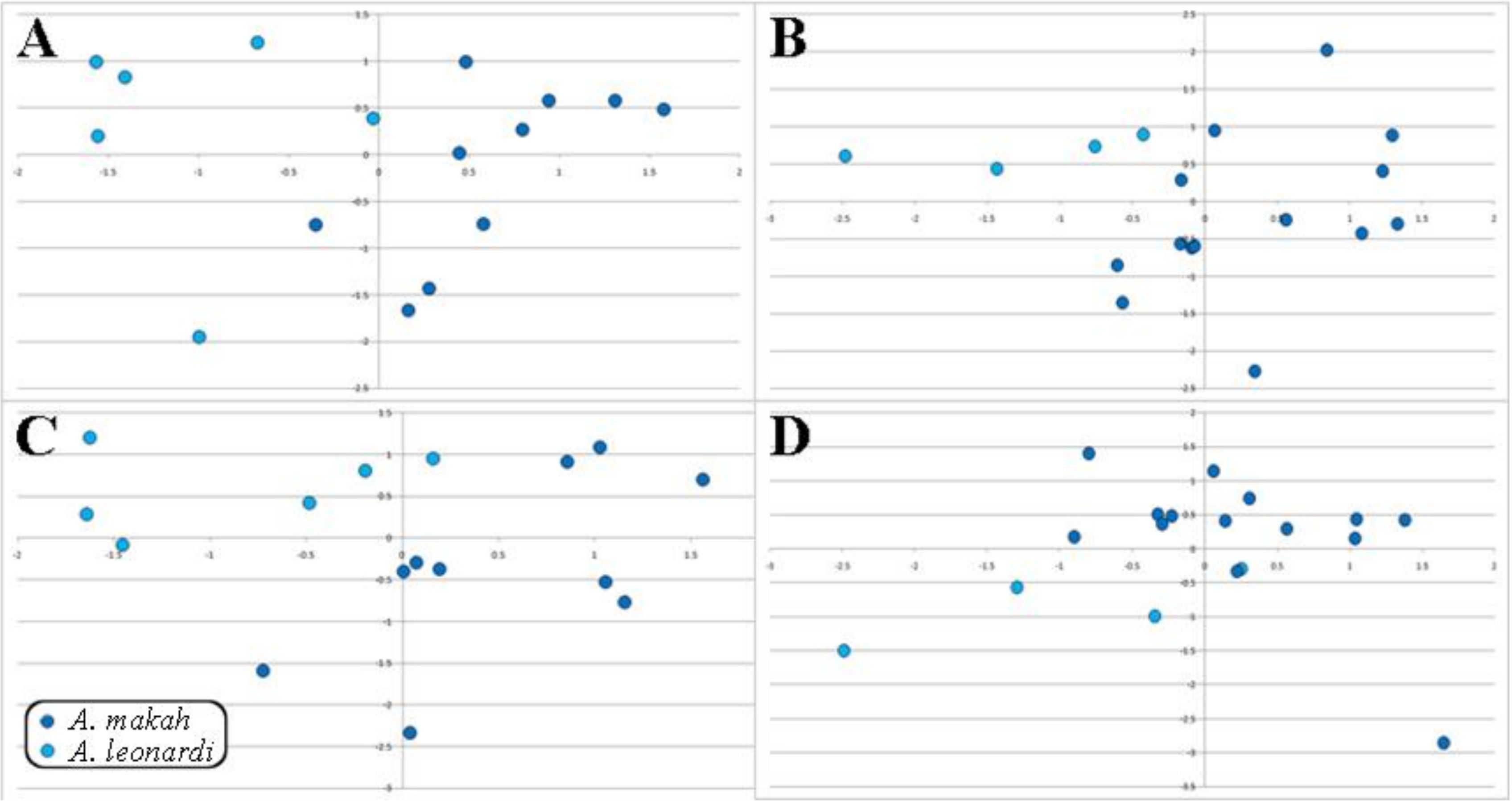

Figure Supplement D.1. Pairwise PCA – *A. makah* and *A. leonardi*. Plotting the two principle components that account for most of the variation in the data recovers *A. makah* and *A. leonardi* as discrete in three of the four analyses. A. male, correlation matrix; B. female, correlation matrix; C. male, covariance matrix; D. female, covariance matrix.

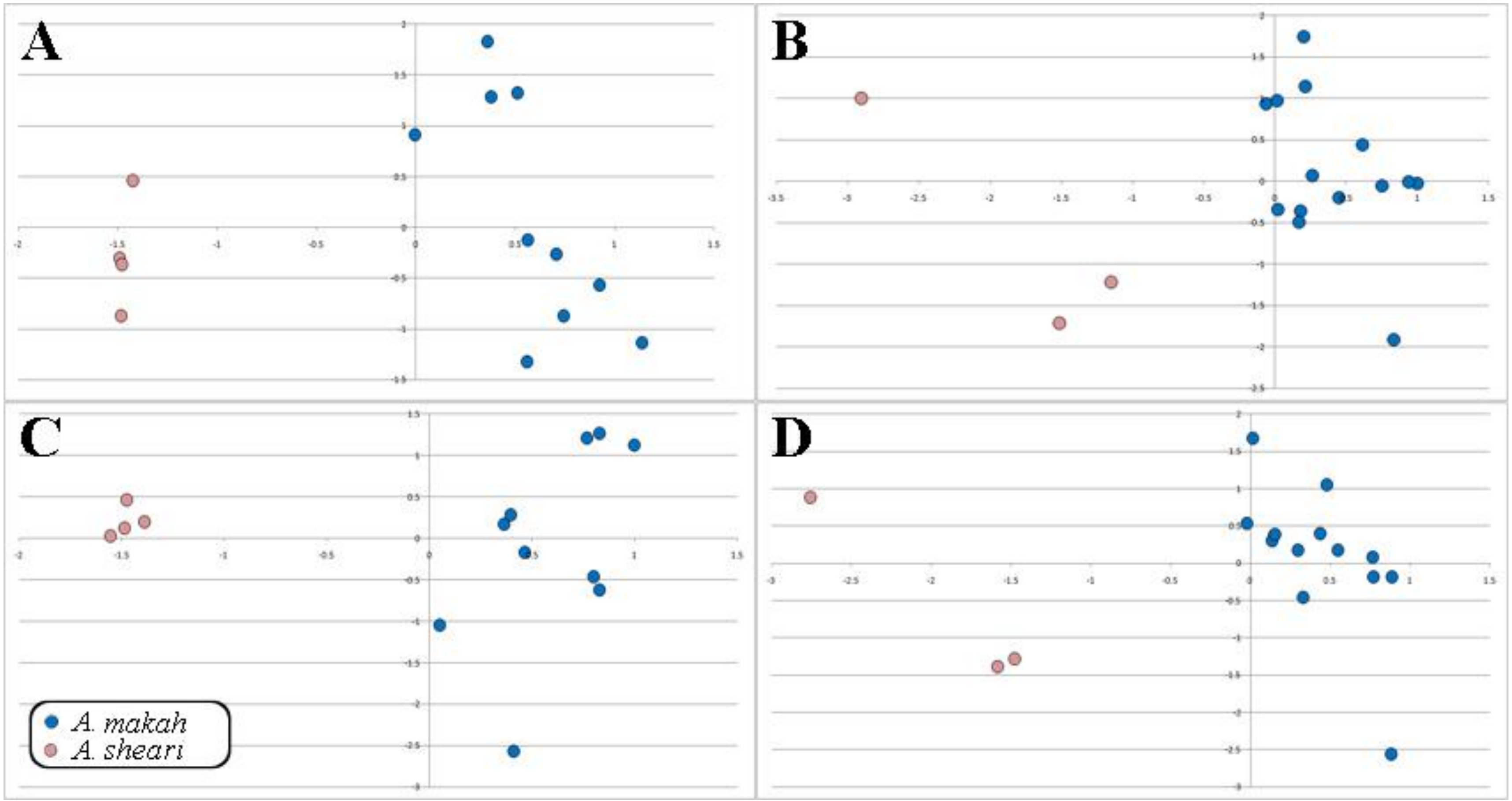

Figure Supplement D.2. Pairwise PCA – *A. makah* and *A. sheari*. Plotting the two principle components that account for most of the variation in the data recovers *A. makah* and *A. sheari* as discrete in all analyses. A. male, correlation matrix; B. female, correlation matrix; C. male, covariance matrix; D. female, covariance matrix.

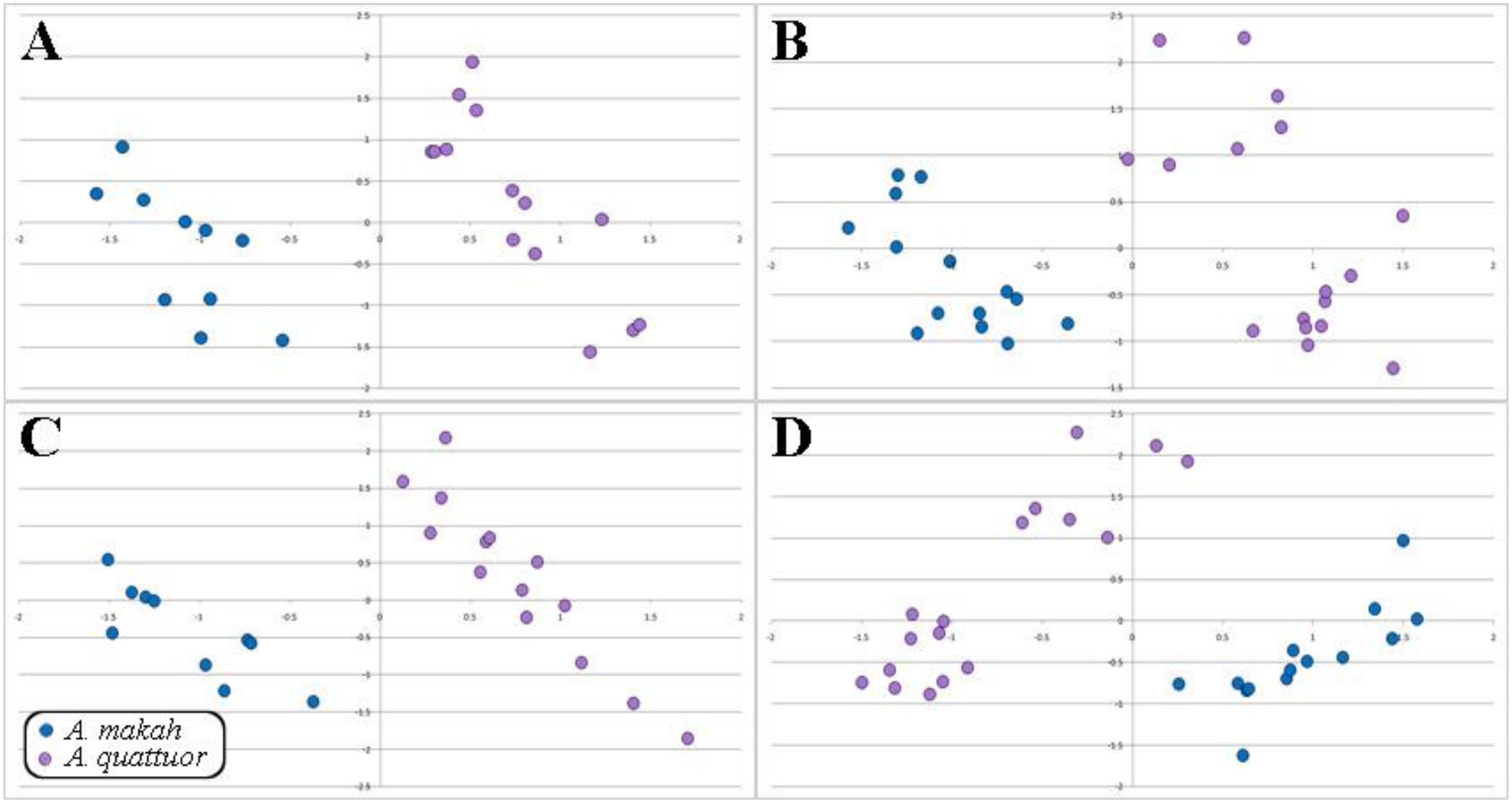

Figure Supplement D.3. Pairwise PCA – *A. makah* and *A. quattuor*. Plotting the two principle components that account for most of the variation in the data recovers *A. makah* and *A. quattuor* as clearly discrete in all analyses. A. male, correlation matrix; B. female, correlation matrix; C. male, covariance matrix; D. female, covariance matrix.

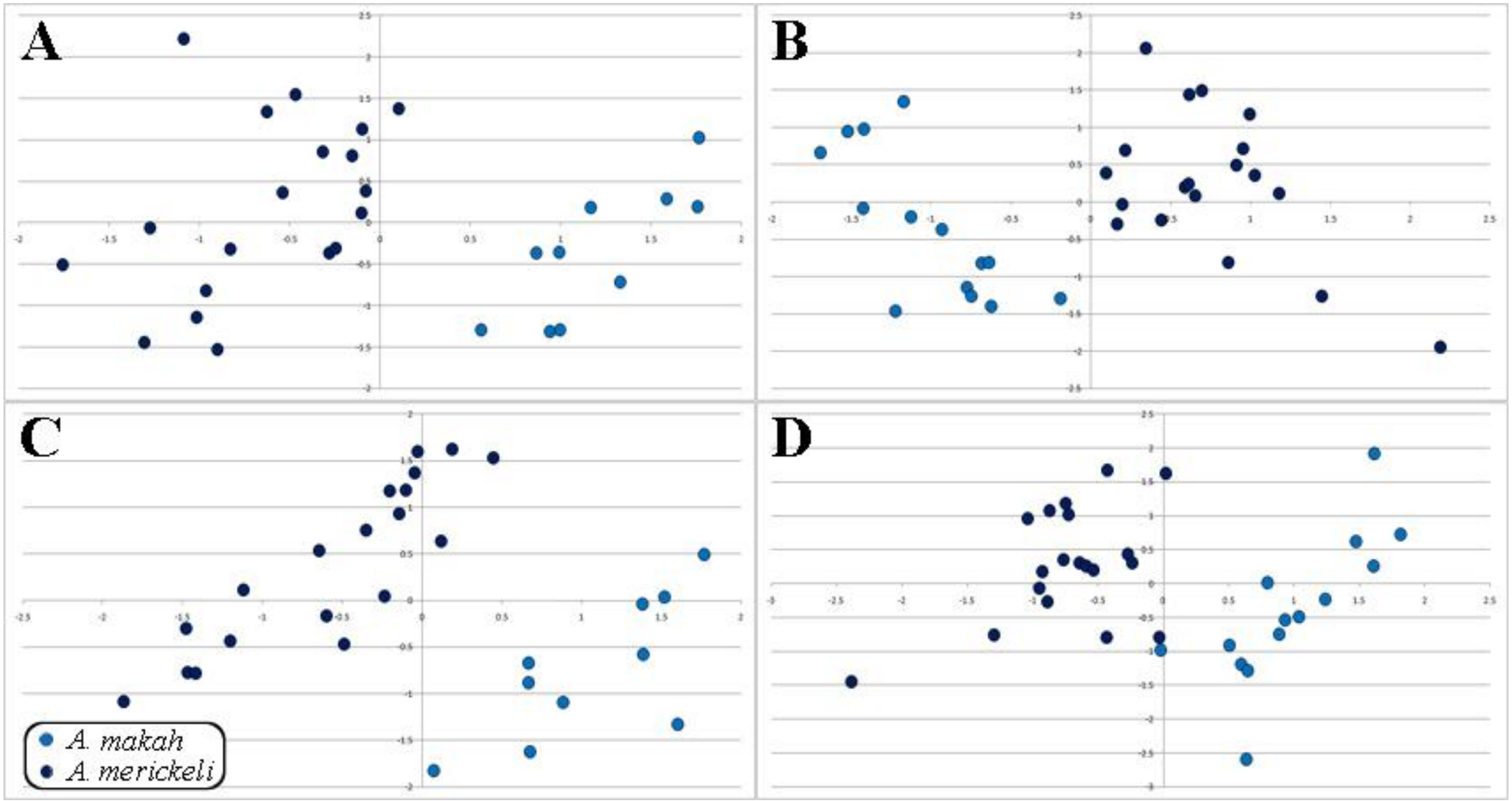

Figure Supplement D.4. Pairwise PCA – *A. makah* and *A. merickeli*. Plotting the two principle components that account for most of the variation in the data recovers *A. makah* and *A. quattuor* as discrete in all pairwise analyses, though weakly so for females based on a covariance matrix. A. male, correlation matrix; B. female, correlation matrix; C. male, covariance matrix; D. female, covariance matrix.

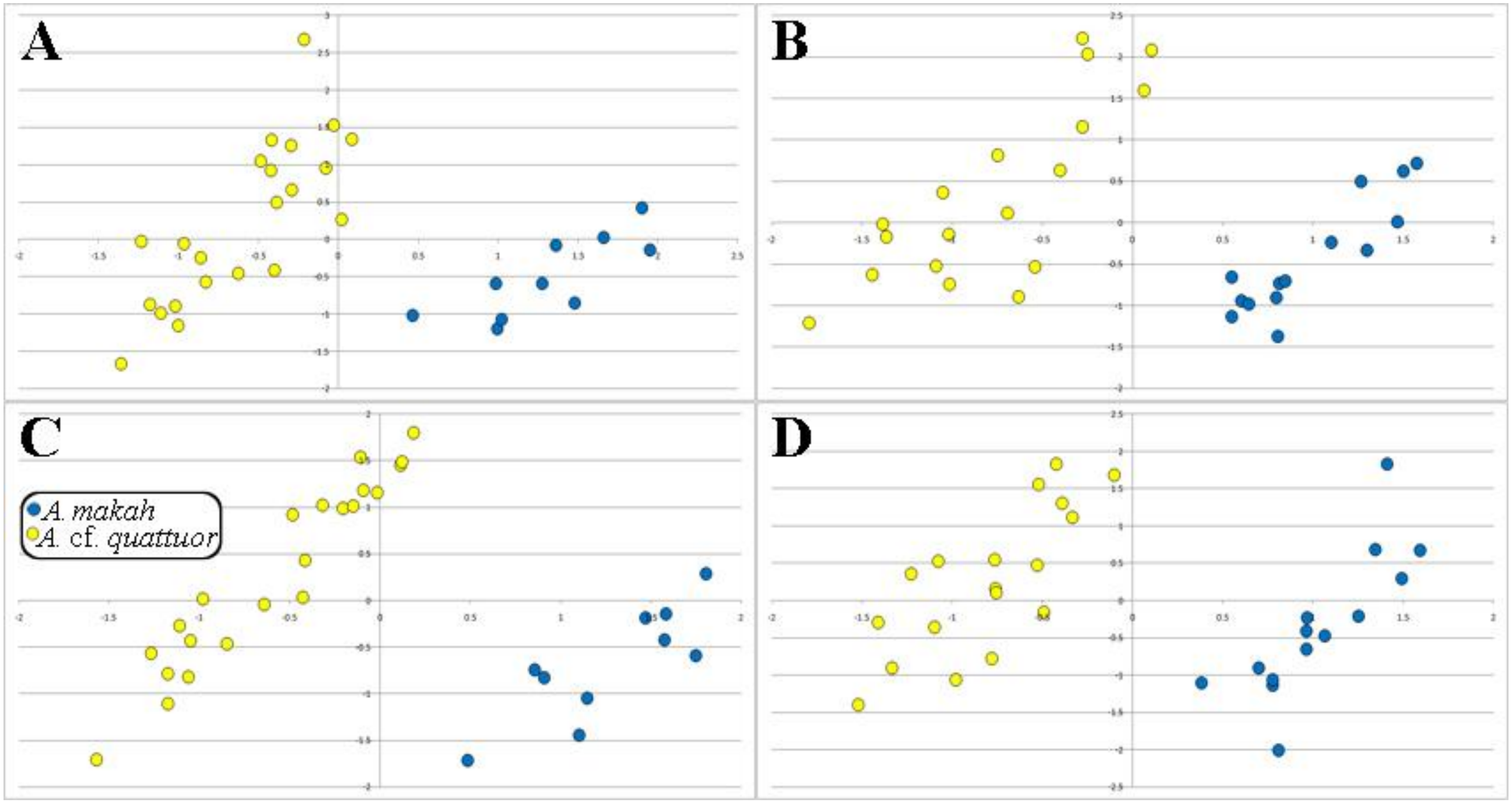

Figure Supplement D.5. Pairwise PCA – *A. makah* and *A.* cf. *quattuor*. Plotting the two principle components that account for most of the variation in the data strongly recovers *A. makah* and *A.* cf. *quattuor* as discrete in all analyses. A. male, correlation matrix; B. female, correlation matrix; C. male, covariance matrix; D. female, covariance matrix.

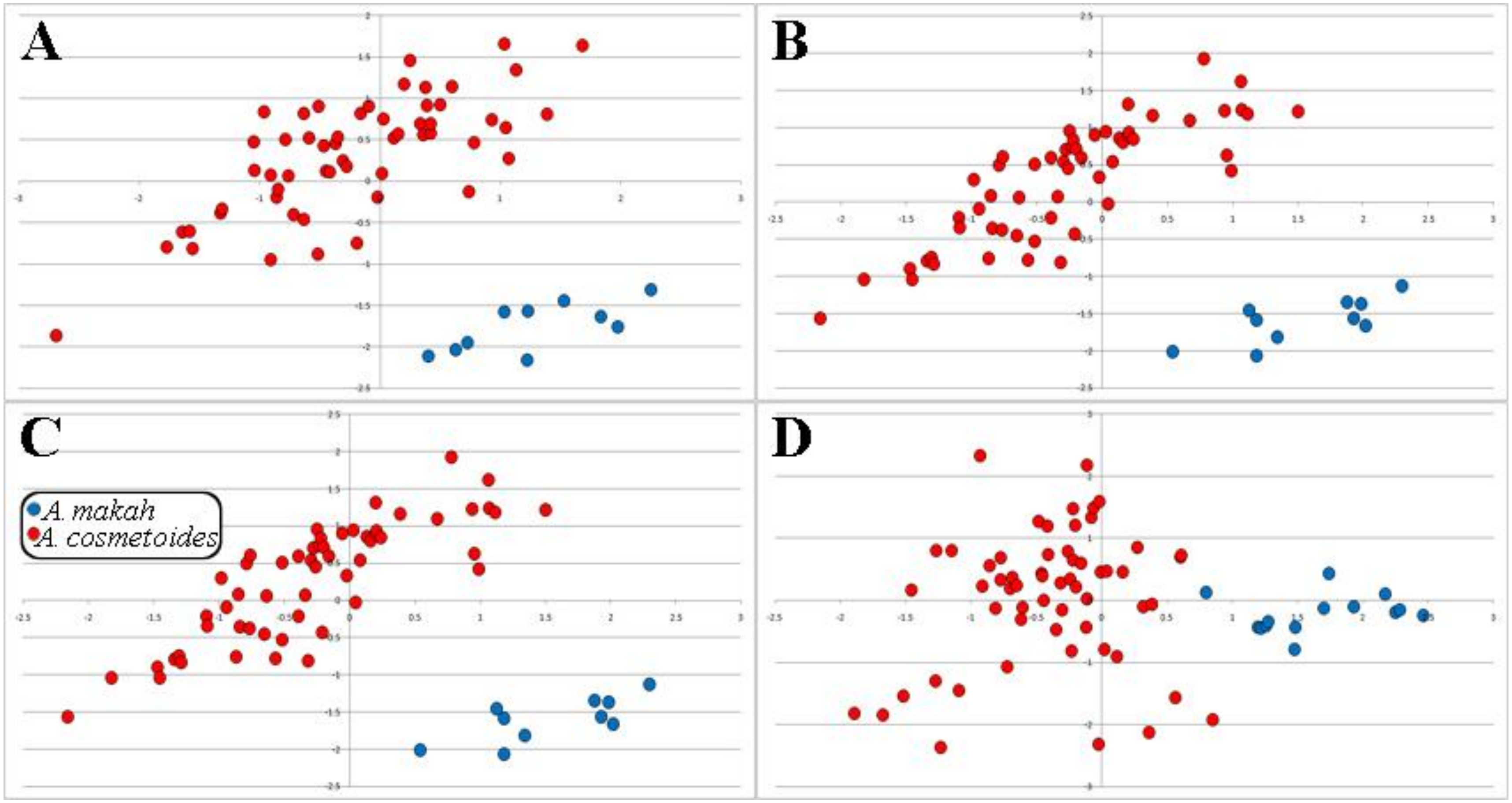

Figure Supplement D.6. Pairwise PCA – *A. makah* and *A. cosmetoides*. Plotting the two principle components that account for most of the variation in the data recovers *A. makah* and *A. cosmetoides* as discrete in all analyses. A. male, correlation matrix; B. female, correlation matrix; C. male, covariance matrix; D. female, covariance matrix.

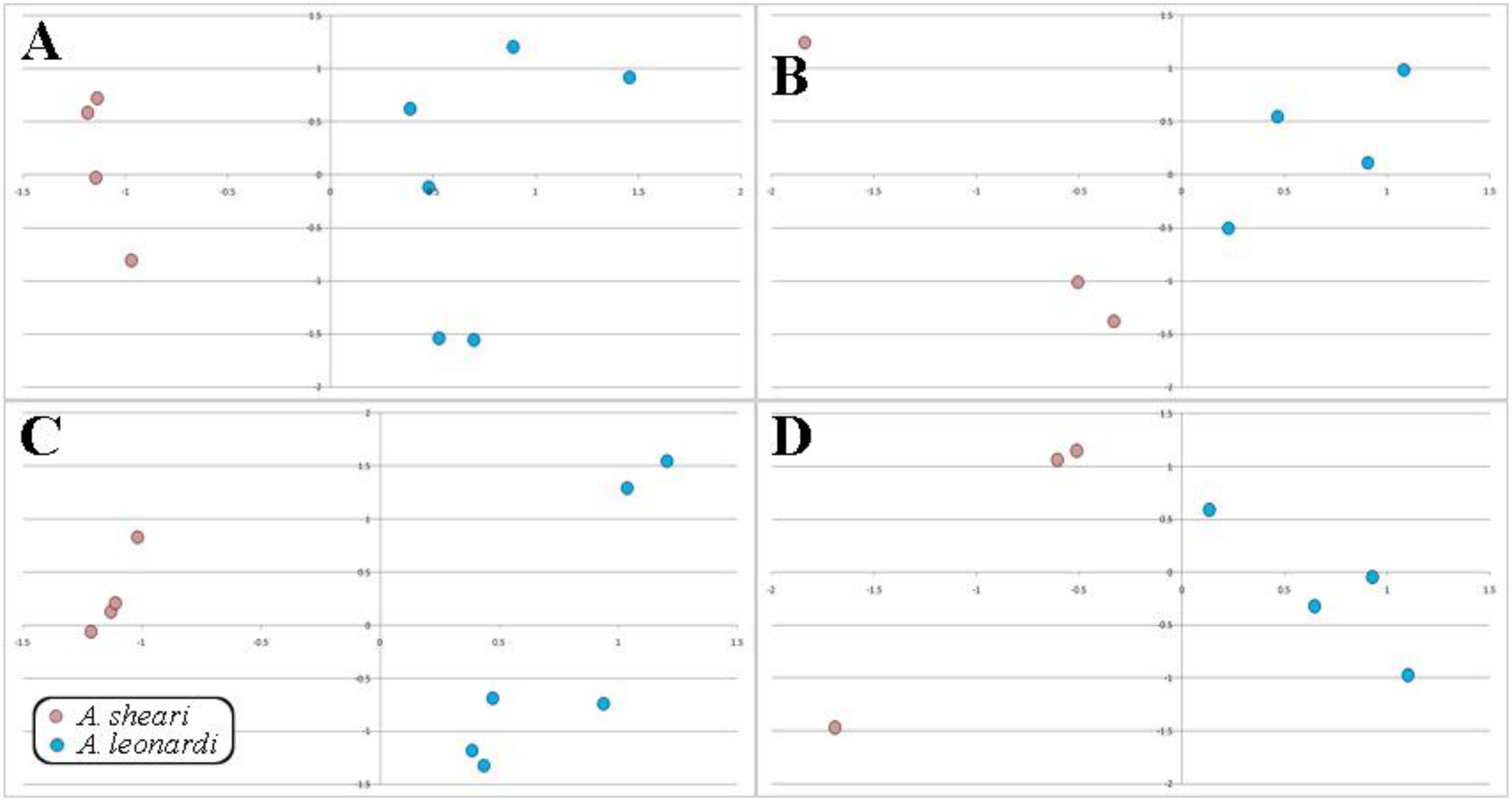

Figure Supplement D.7. Pairwise PCA – *A. leonardi* and *A. sheari*. Plotting the two principle components that account for most of the variation in the data recovers *A. leonardi* and *A. sheari* as discrete in all analyses. A. male, correlation matrix; B. female, correlation matrix; C. male, covariance matrix; D. female, covariance matrix.

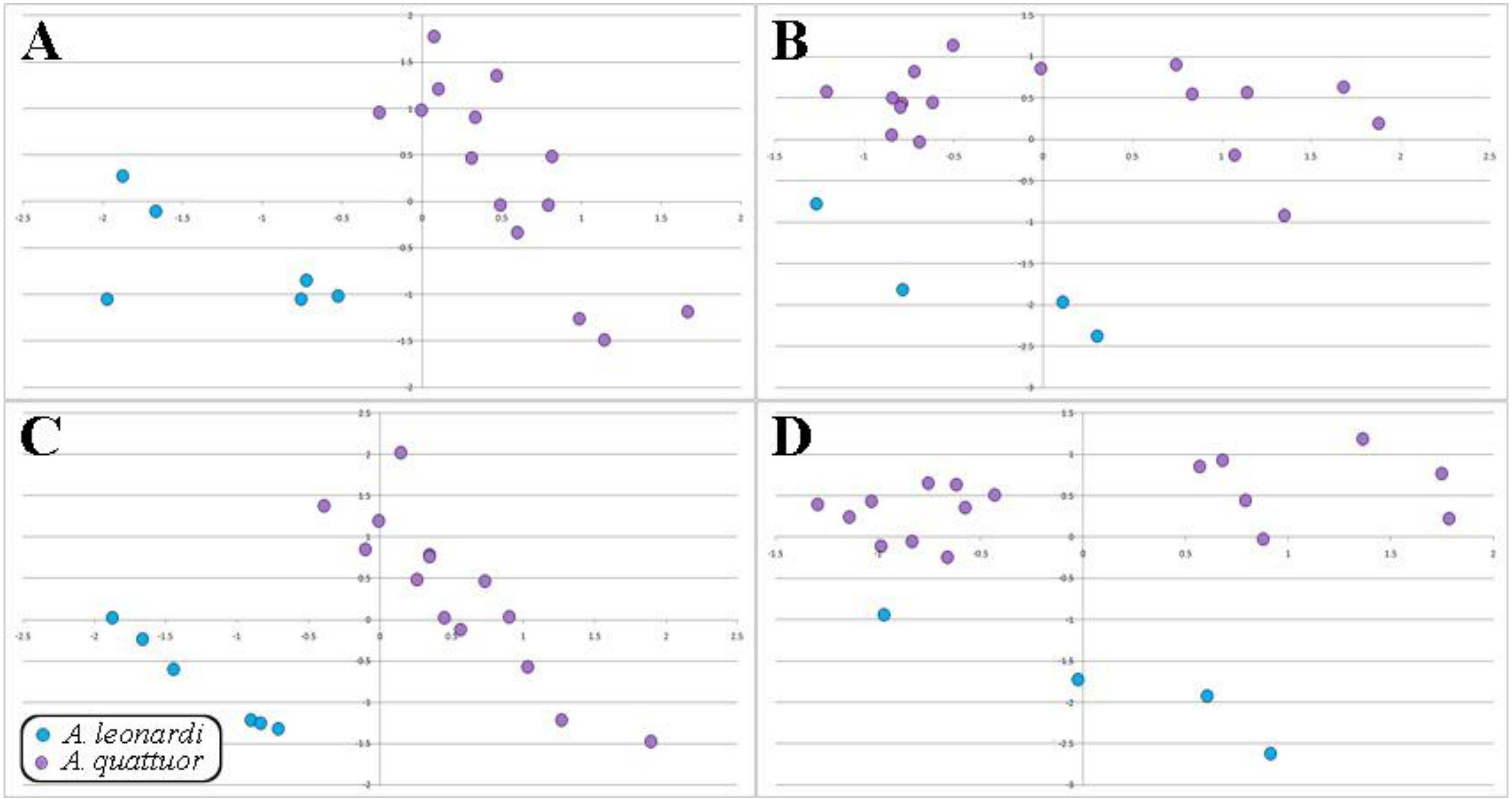

Figure Supplement D.8. Pairwise PCA – *A. leonardi* and *A. quattuor*. Plotting the two principle components that account for most of the variation in the data recovers *A. leonardi* and *A. quattuor* as discrete in all analyses. A. male, correlation matrix; B. female, correlation matrix; C. male, covariance matrix; D. female, covariance matrix.

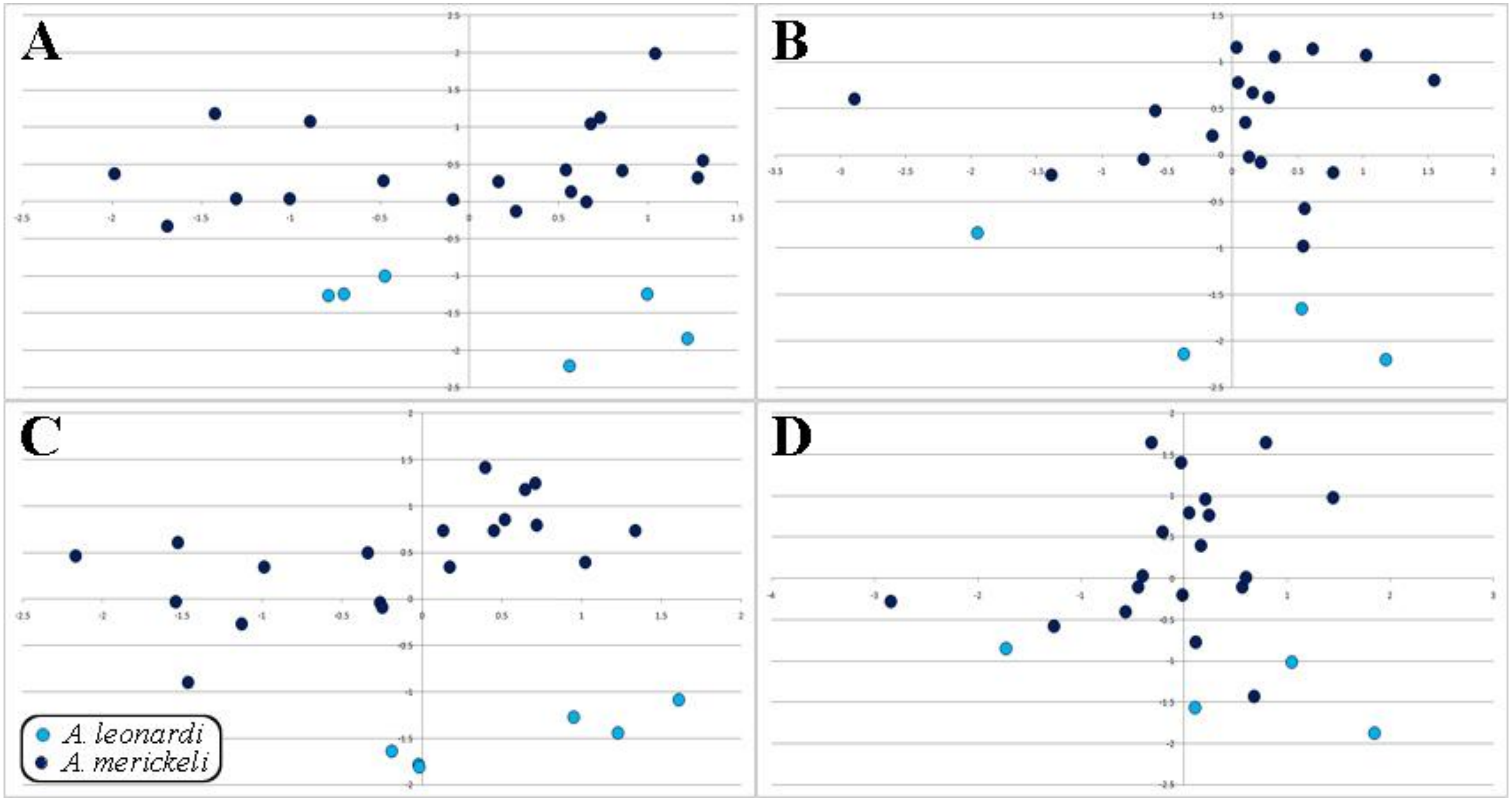

Figure Supplement D.9. Pairwise PCA – *A. leonardi* and *A. merickeli*. Plotting the two principle components that account for most of the variation in the data recovers *A. leonardi* and *A. merickeli* as discrete in three of four analyses; there is no morphospace gap in the covariance-based female analysis. A. male, correlation matrix; B. female, correlation matrix; C. male, covariance matrix; D. female, covariance matrix.

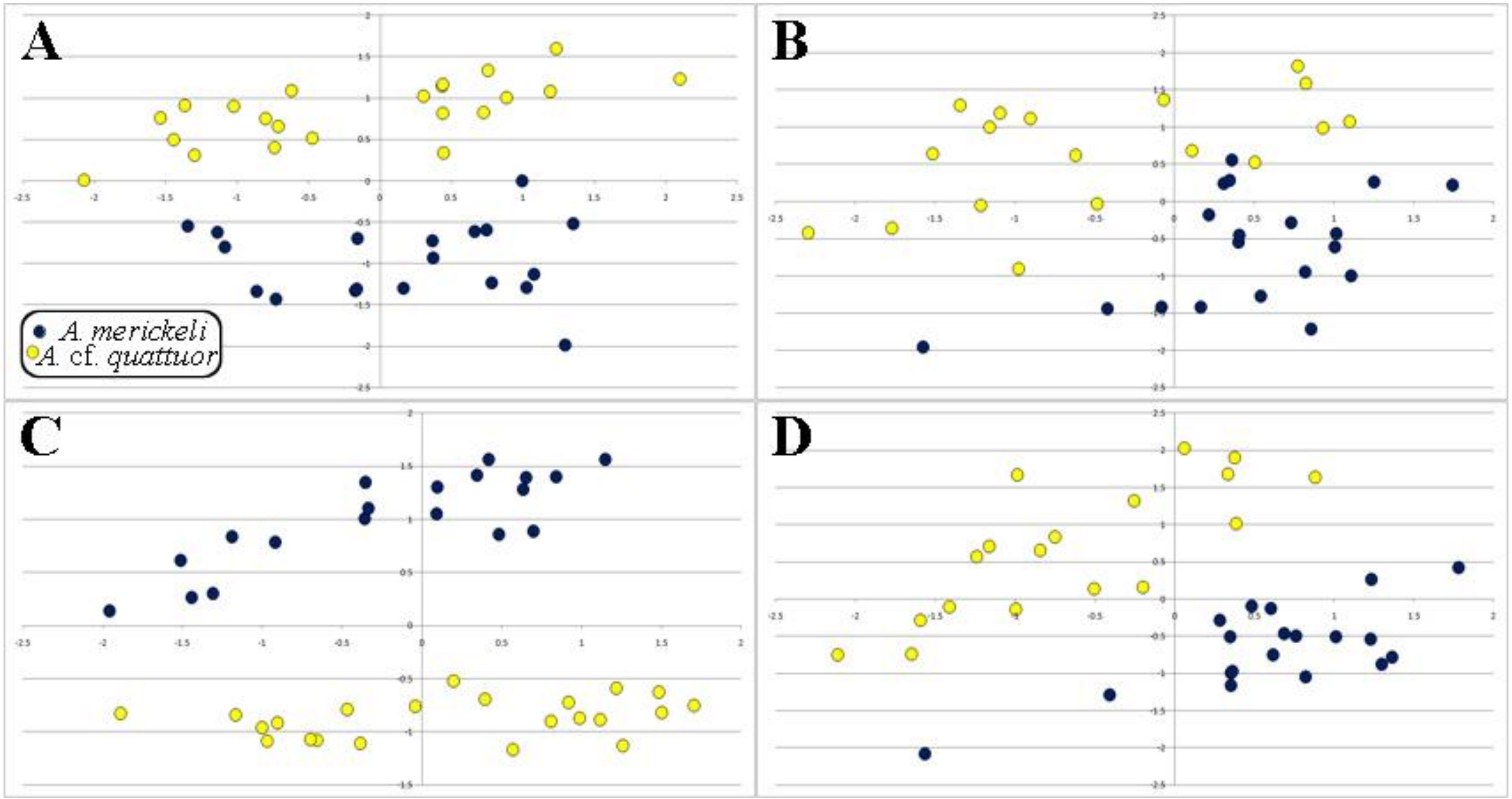

Figure Supplement D.10. Pairwise PCA – *A. leonardi* and *A.* cf. *quattuor*. Plotting the two principle components that account for most of the variation in the data recovers *A. leonardi* and *A.* cf. *quattuor* as discrete in three of four analyses; there is no morphospace gap in the correlation-based female analysis. A. male, correlation matrix; B. female, correlation matrix; C. male, covariance matrix; D. female, covariance matrix.

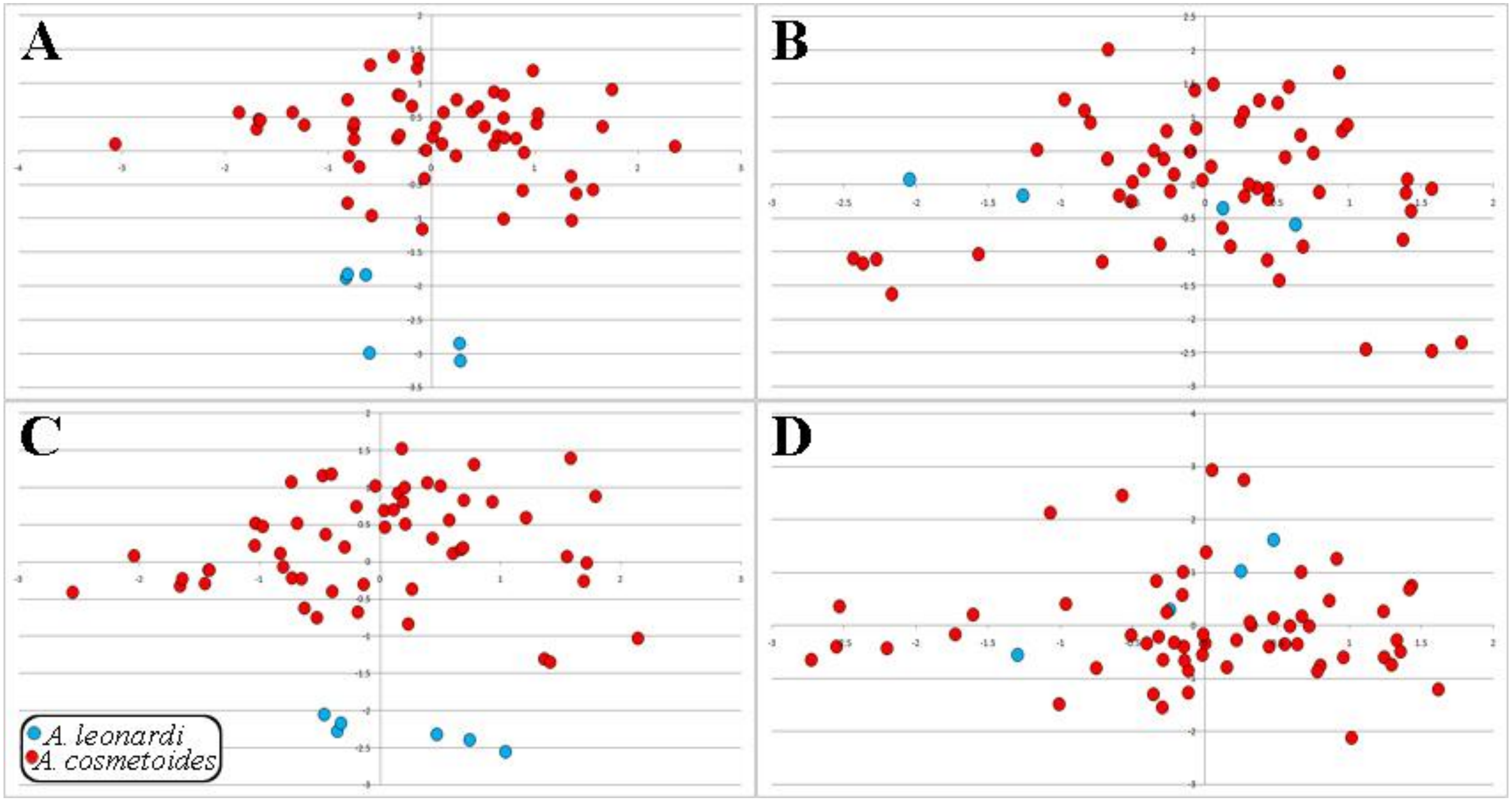

Figure Supplement D.11. Pairwise PCA – *A. leonardi* and *A. cosmetoides*. Plotting the two principle components that account for most of the variation in the data recovers *A. leonardi* and *A. cosmetoides* as discrete in male analyses only; female *A. leonardi* are apparently obscured by the variable *A. cosmetoides* females. A. male, correlation matrix; B. female, correlation matrix; C. male, covariance matrix; D. female, covariance matrix.

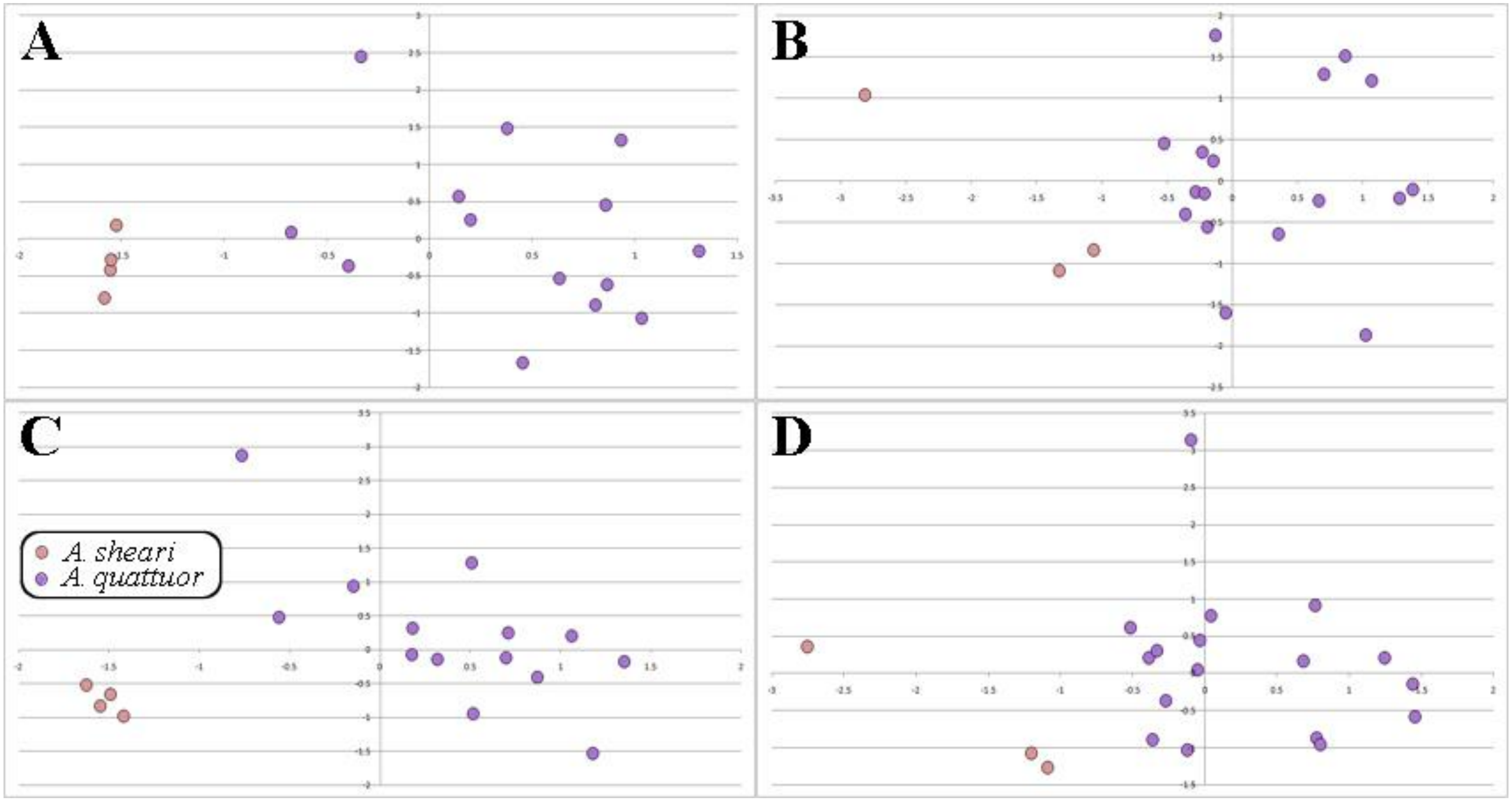

Figure Supplement D.12. Pairwise PCA – *A. sheari* and *A. quattuor*. Plotting the two principle components that account for most of the variation in the data recovers *A. sheari* and *A. quattuor* as clearly discrete in all analyses. A. male, correlation matrix; B. female, correlation matrix; C. male, covariance matrix; D. female, covariance matrix.

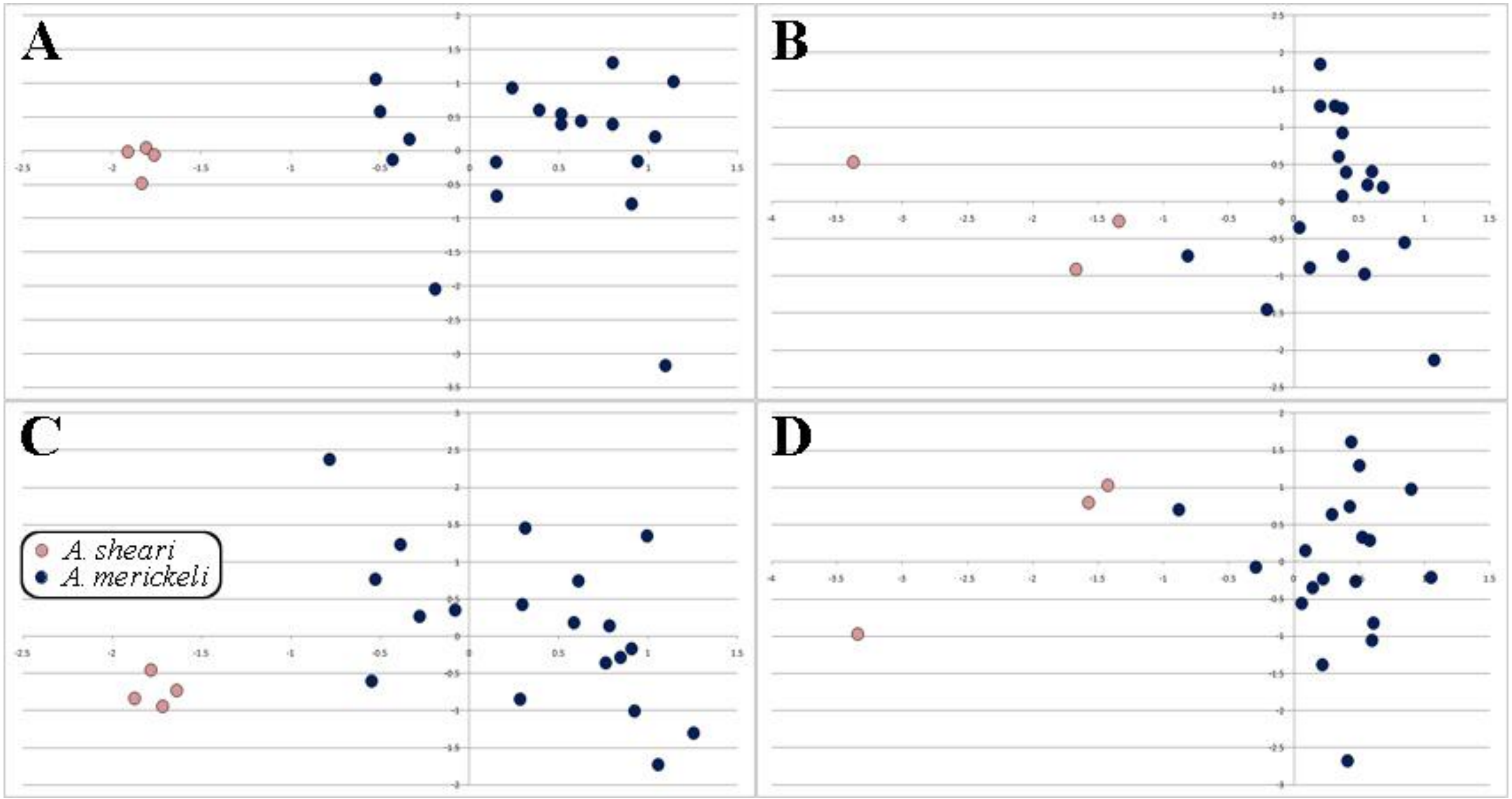

Figure Supplement D.13. Pairwise PCA – *A. sheari* and *A. merickeli*. Plotting the two principle components that account for most of the variation in the data recovers *A. sheari* and *A. merickeli* as clearly discrete in all analyses. A. male, correlation matrix; B. female, correlation matrix; C. male, covariance matrix; D. female, covariance matrix.

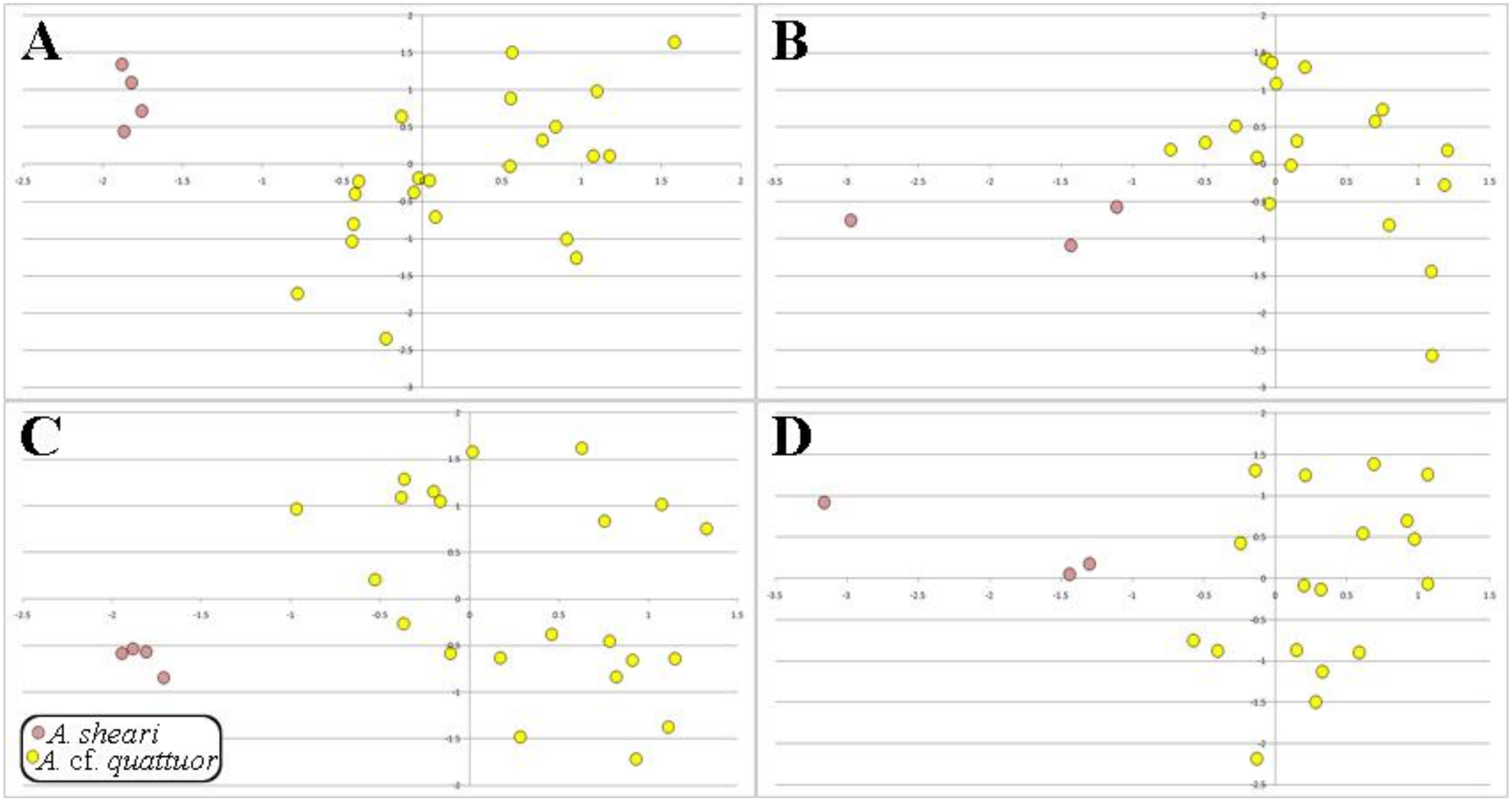

Figure Supplement D.14. Pairwise PCA – *A. sheari* and *A.* cf. *quattuor*. Plotting the two principle components that account for most of the variation in the data recovers *A. sheari* and *A.* cf. *quattuor* as clearly discrete in all analyses. A. male, correlation matrix; B. female, correlation matrix; C. male, covariance matrix; D. female, covariance matrix.

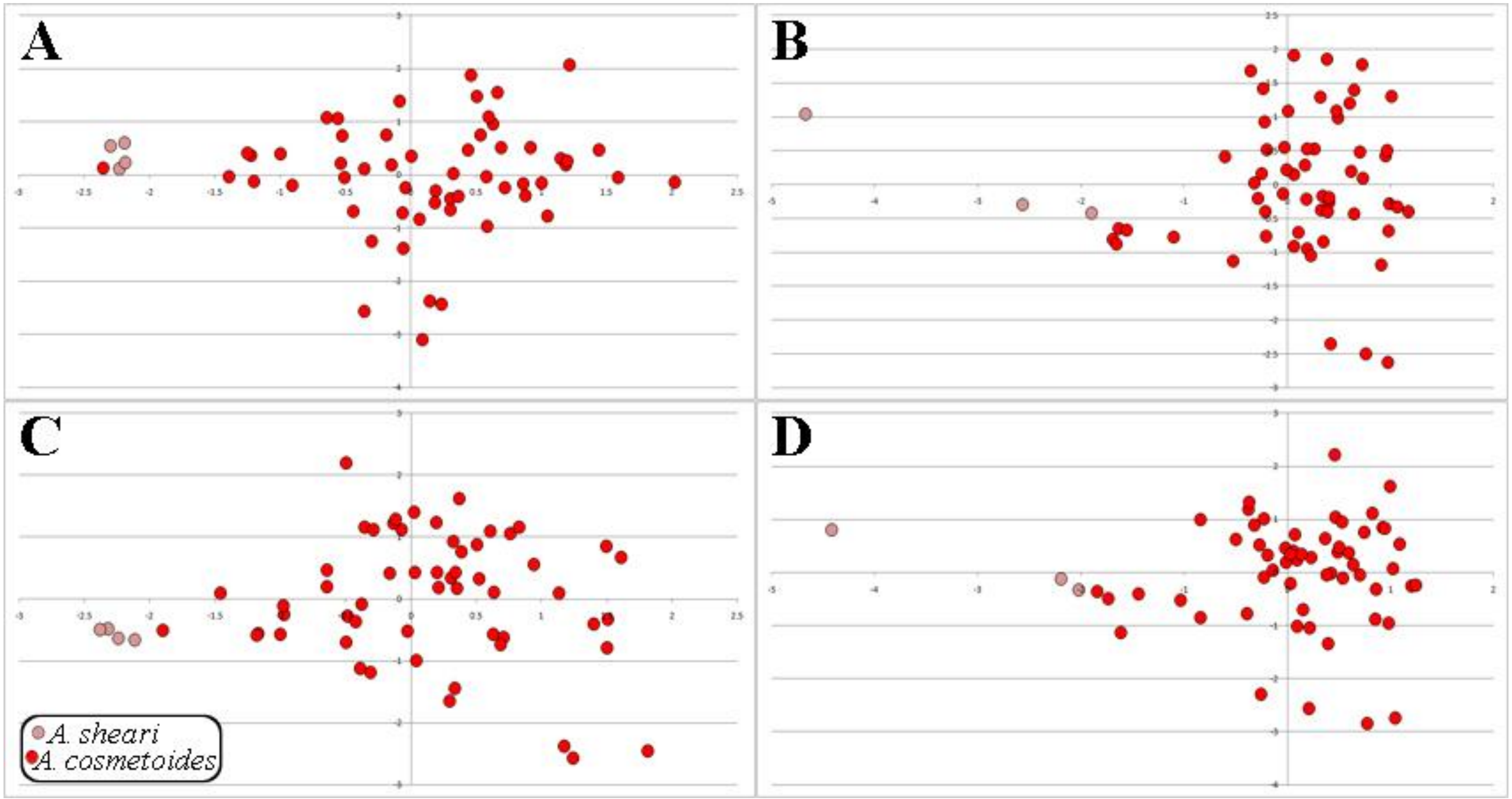

Figure Supplement D.15. Pairwise PCA – *A. sheari* and *A. cosmetoides*. Plotting the two principle components that account for most of the variation in the data clusters *A. sheari* in morphospace for all analyses; *A. cosmetoides* is discrete in three of four analyses, a male in the correlation matrix analysis brackets the *A. sheari* cluster. A. male, correlation matrix; B. female, correlation matrix; C. male, covariance matrix; D. female, covariance matrix.

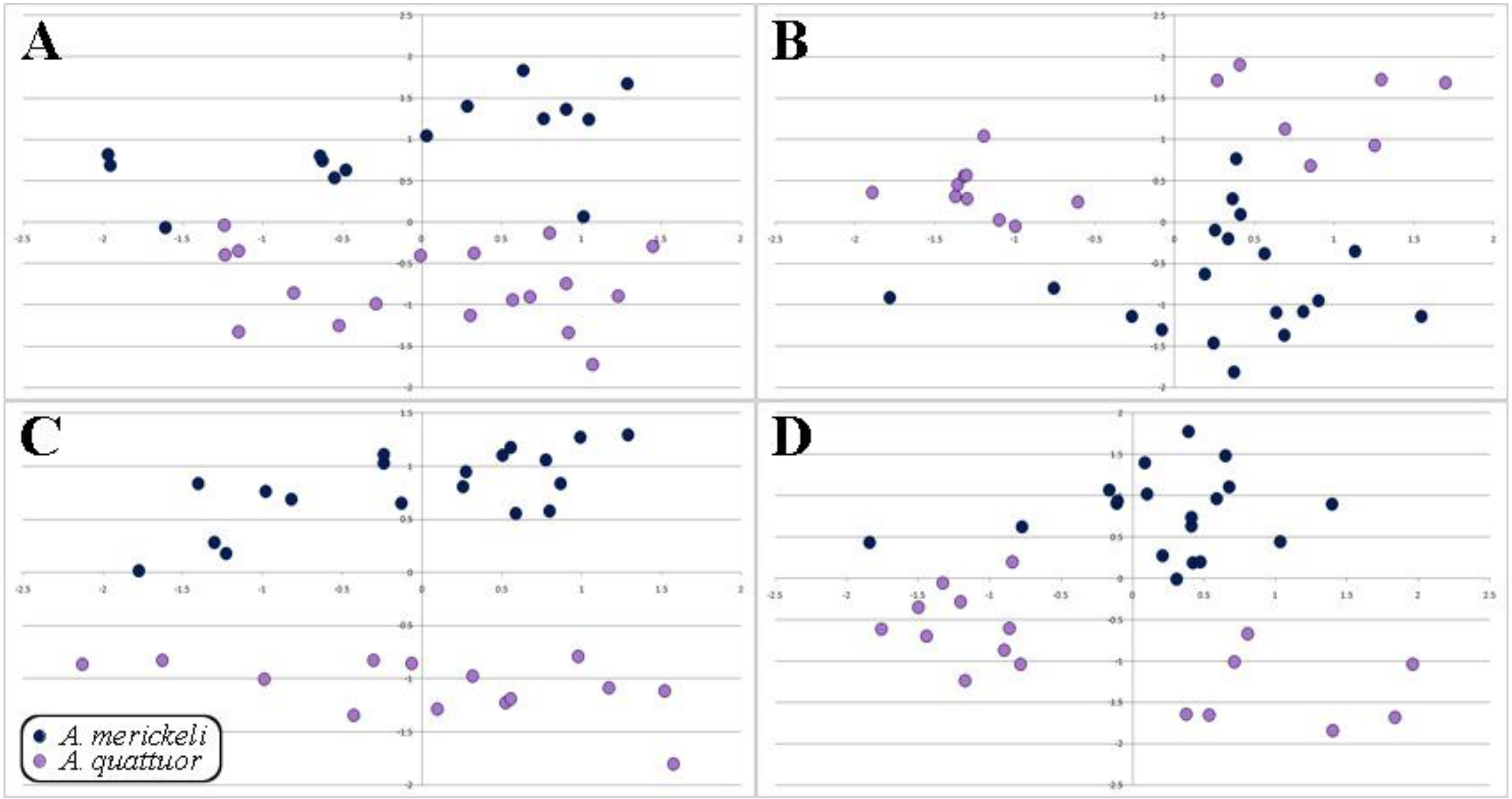

Figure Supplement D.16. Pairwise PCA – *A. quattuor* and *A. merickeli*. Plotting the two principle components that account for most of the variation in the data shows nearly-independent clusters for *A. merickeli* and *A. quattuor* in all analyses, though weakly in three of them. A. male, correlation matrix; B. female, correlation matrix; C. male, covariance matrix; D. female, covariance matrix.

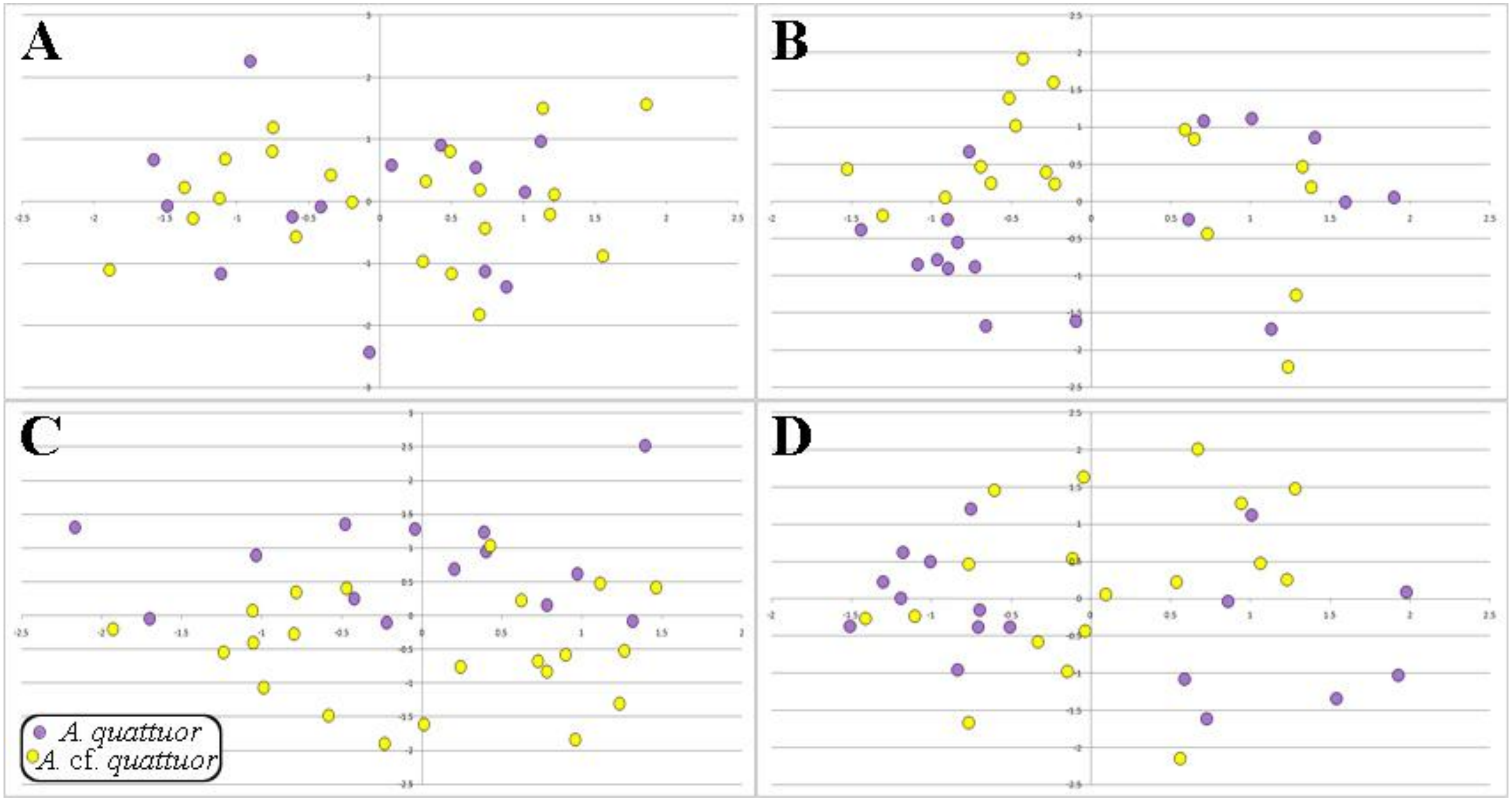

Figure Supplement D.17. Pairwise PCA – *A. quattuor* and *A.* cf. *quattuor*. Plotting the two principle components that account for most of the variation does not segregate *A.* cf. *quattuor* from *A. quattuor* for any of the analyses. A. male, correlation matrix; B. female, correlation matrix; C. male, covariance matrix; D. female, covariance matrix.

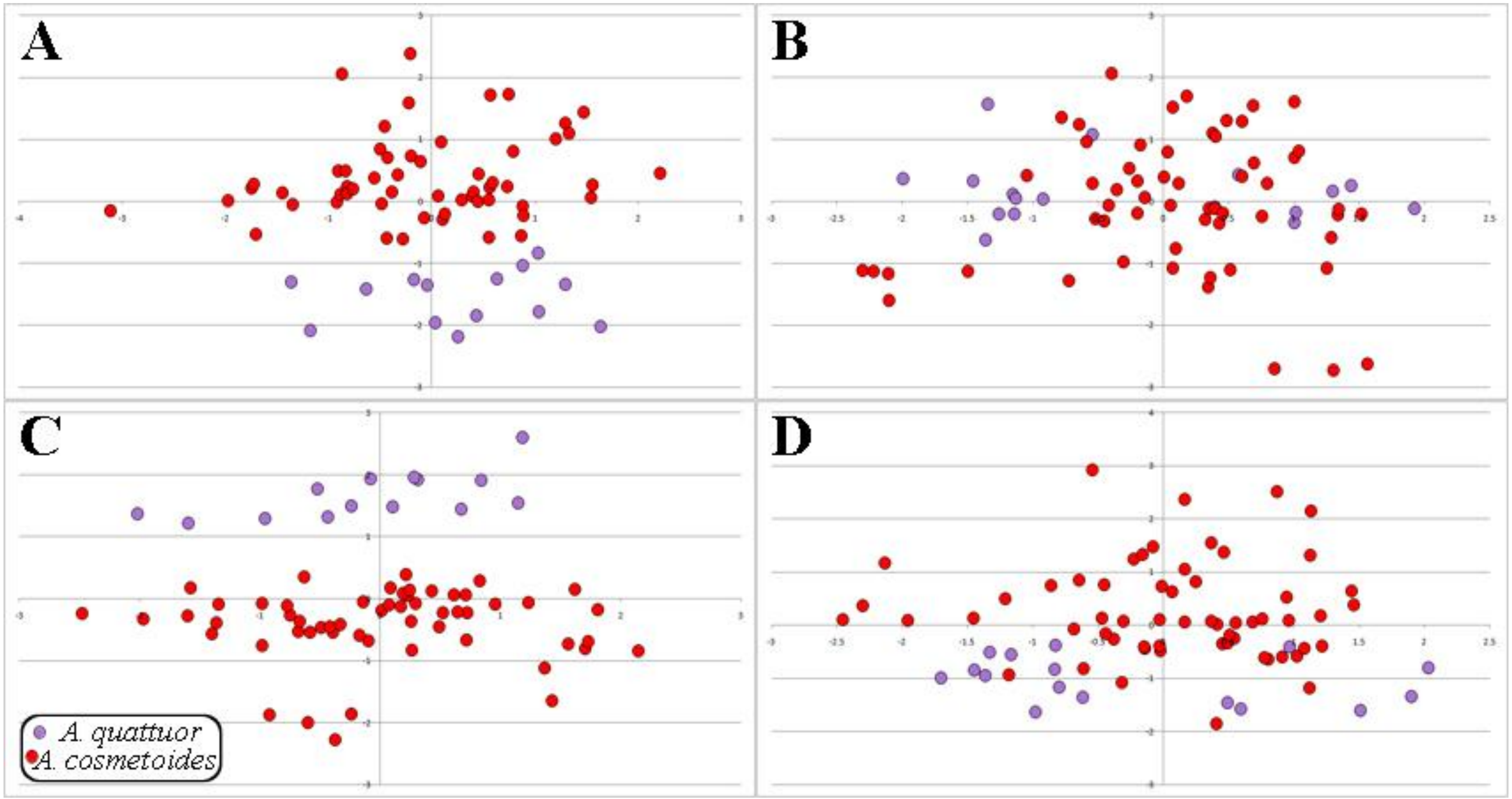

Figure Supplement D.18. Pairwise PCA – *A. quattuor* and *A. cosmetoides*. Plotting the two principle components that account for most of the variation in the data recovers *A. quattuor* and *A. cosmetoides* as discrete in male analyses only; female *A. quattuor* appear obscured by the variability of the female *A. cosmetoides* data set. A. male, correlation matrix; B. female, correlation matrix; C. male, covariance matrix; D. female, covariance matrix.

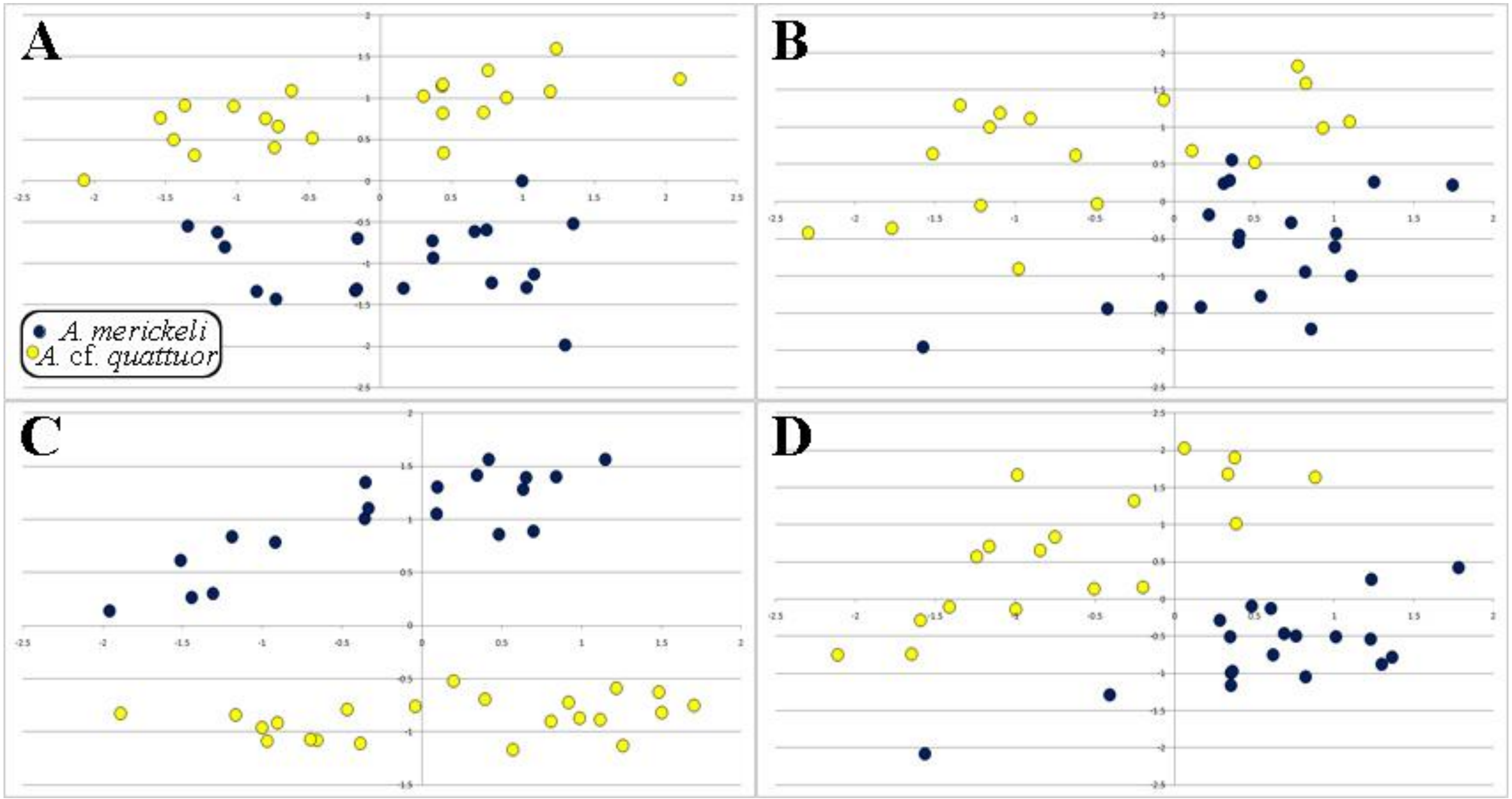

Figure Supplement D.19. Pairwise PCA – *A. merickeli* and *A.* cf. *quattuor*. Plotting the two principle components that account for most of the variation in the data shows discrete clusters for *A. merickeli* and *A. quattuor* in three of four analyses; the female covariance analyses nearly segregates the two species.. A. male, correlation matrix; B. female, correlation matrix; C. male, covariance matrix; D. female, covariance matrix.

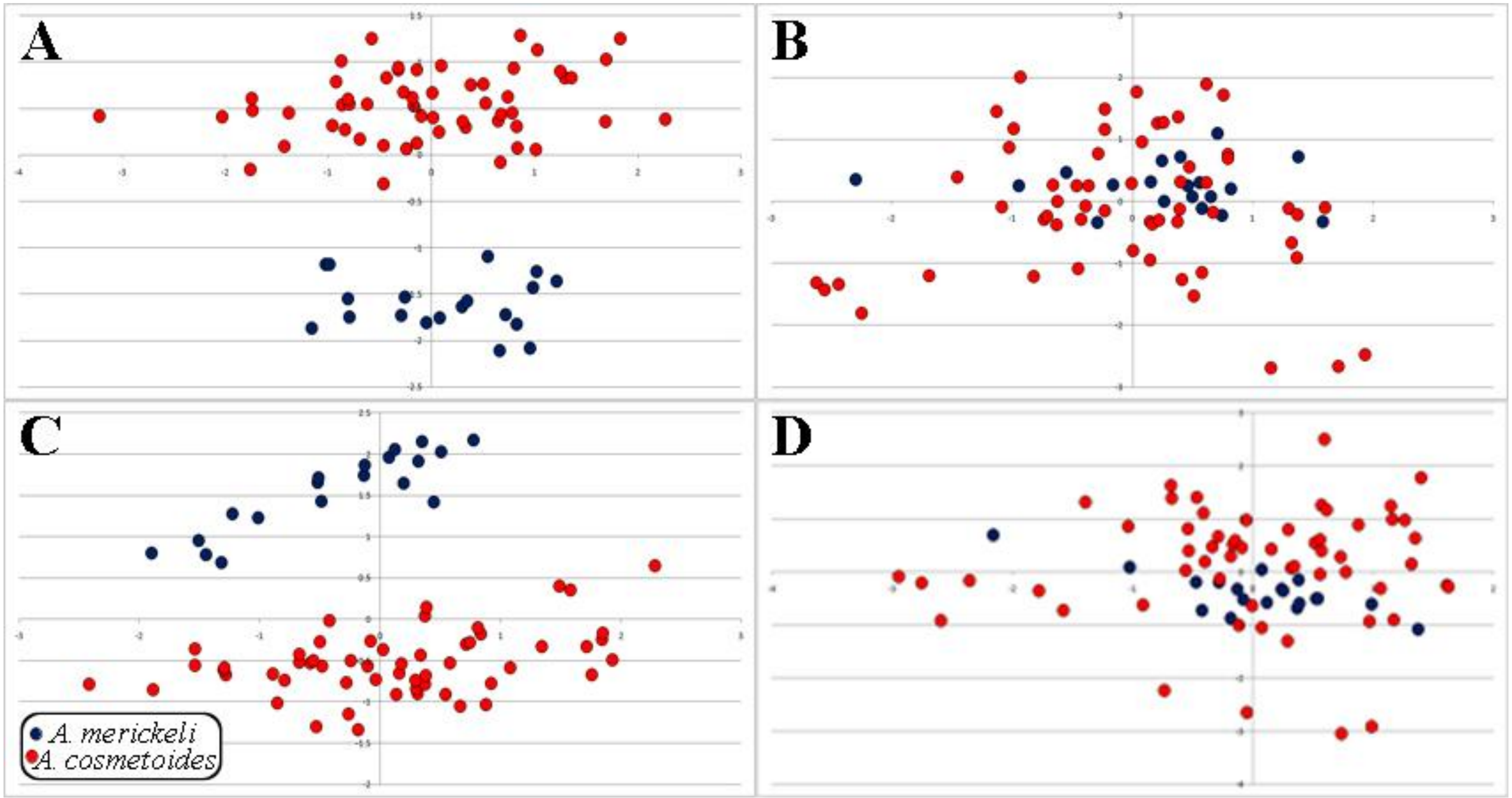

Figure Supplement D.20. Pairwise PCA – *A. merickeli* and *A. cosmetoides*. Plotting the two principle components that account for most of the variation in the data recovers *A. merickeli* and *A. cosmetoides* as discrete in male analyses only; female *A. merickeli* appear obscured by the variability of the female *A. cosmetoides* data set. A. male, correlation matrix; B. female, correlation matrix; C. male, covariance matrix; D. female, covariance matrix.

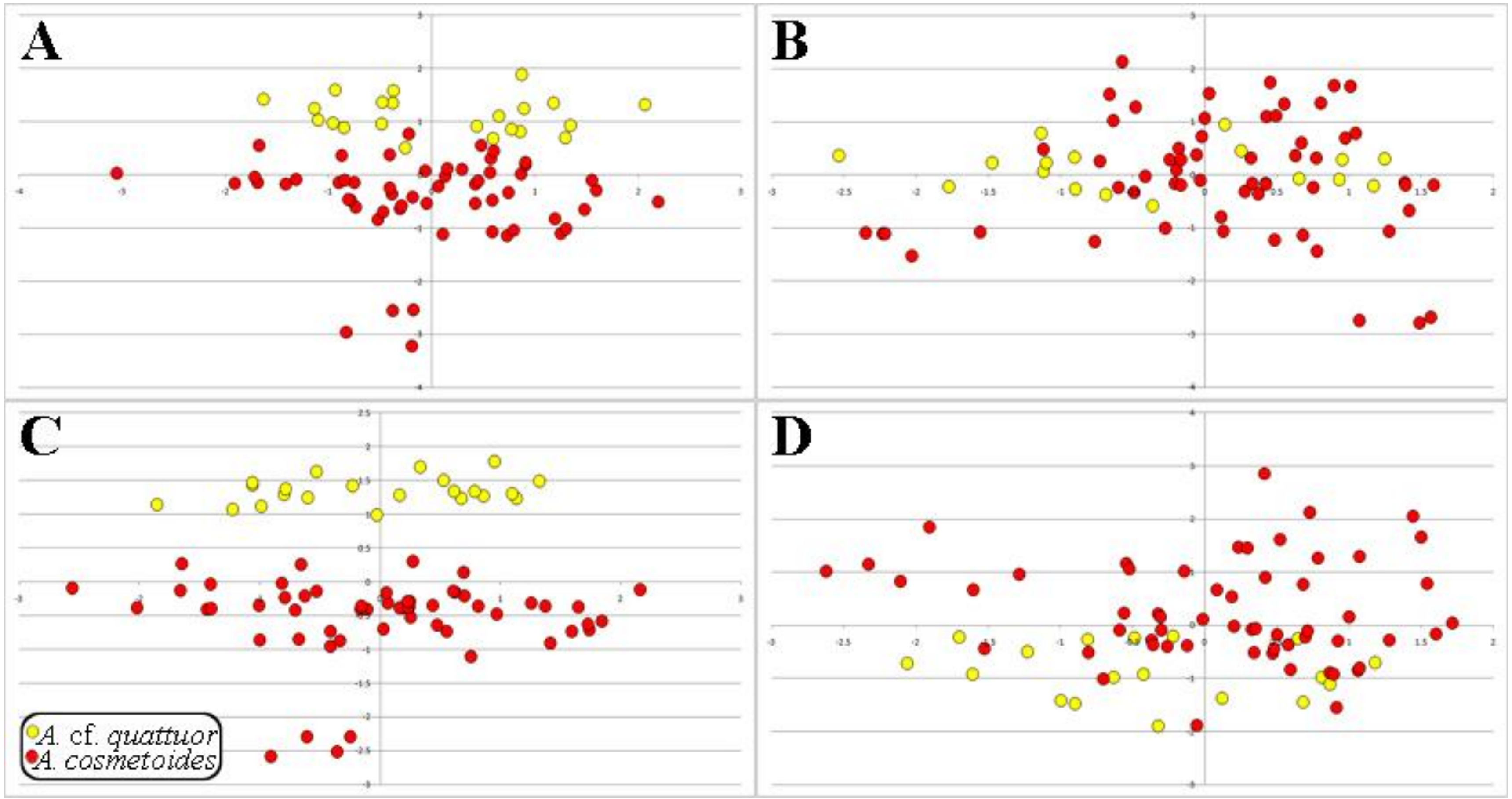

Figure Supplement D.21. Pairwise PCA – *A.* cf. *quattuor* and *A. cosmetoides*. Plotting the two principle components that account for most of the variation in the data recovers *A.* cf. *quattuor* and *A. cosmetoides* as discrete in the covariance male analysis only, though the correlation matrix is nearly so; female *A. merickeli* appear obscured by the variability of the female *A. cosmetoides* data set.
A. male, correlation matrix; B. female, correlation matrix; C. male, covariance matrix; D. female, covariance matrix.

Table Supplement D.7 Variables Influencing and Percent Variation Explained by Pairwise Principle Components.

Species I	Species II	Sex	Matix	Variation PC I	Variation PC II	% PC I	% PC II	% Total
A. makah	*A. leonardi*	M	Cor	pos: tib, fem, pat, II	pos: car, metat; neg I, eye2	49.6	12.2	61.8
A. makah	*A. leonardi*	F	Cor	pos: tib, fem, pat, II	pos: car, metat; neg I, eye2	37.4	14.8	52.2
A. makah	*A. leonardi*	M	Cov	pos: fem, metat, tar, II	pos: tar; neg: II	67.2	13.6	80.8
A. makah	*A. leonardi*	F	Cov	pos: fem, metat, tib	neg: metat, car, scu	56.6	17.8	74.4
A. leonardi	*A.* cf. *quattuor*	M	Cor	pos: metat, tib, tar, fem, II	neg: I, scu, eye2, pfem	36.9	28.1	65.0
A. leonardi	*A.* cf. *quattuor*	F	Cor	pos: metat, fem, III, tar, eye	pos: scu, eye2, I, pfem; neg eye	33.9	26.1	60.0
A. leonardi	*A.* cf. *quattuor*	M	Cov	pos: tar, fem, metat, tib, II	pos: I, eye2, scu	51.5	34.0	85.5
A. leonardi	*A.* cf. *quattuor*	F	Cov	pos: fem, metat, tar, II, I	pos: I, eye2, scu	43.3	27.9	71.2
A. leonardi	*A. cosmetoides*	M	Cor	pos: fem, tar, metat	pos: scu, III; neg: eye, fem, pat	37.3	17.0	54.3
A. leonardi	*A. cosmetoides*	F	Cor	pos: tib, fem, metat, tar	pos: III, II	27.1	18.9	46.0
A. leonardi	*A. cosmetoides*	M	Cov	pos: tar, metat, fem	pos: I, II, eye2, scu	49.1	19.7	68.8
A. leonardi	*A. cosmetoides*	F	Cov	pos: eye2, I, II, III, tar, fem	pos: metat, tar, fem; neg: eye2	29.1	26.3	55.4
A. sheari	*A. quattuor*	M	Cor	pos: II, eye, I, fem, metat	pos: III, IV; neg: metap	65.9	9.1	75.0
A. sheari	*A. quattuor*	F	Cor	pos: eye2, II, fem, metat, tar	neg: IV, III	62.9	11.1	74.0
A. sheari	*A. quattuor*	M	Cov	pos: tar, eye2, metat, fem, I	pos: scu, neg: metat	86.8	5.0	91.8
A. sheari	*A. quattuor*	F	Cov	pos: eye2, fem, tar, scu	pos: eye; neg: tar	82.4	5.2	87.6
A. sheari	*A. merickeli*	M	Cor	pos: tar, II, fem, eye, metat	neg: III, I, pfem	56.0	14.4	70.4
A. sheari	*A. merickeli*	F	Cor	pos: fem, tar, tib, metat, eye2	neg: III, I, IV	60.1	9.1	69.2
A. sheari	*A. merickeli*	M	Cov	pos: tar, metat, fem, II	pos: eye2; neg: metat, tar	86.3	5.2	91.5

(table continues)

Table Supplement D.7 Variables Influencing and Percent Variation Explained by Pairwise Principle Components (cont).

Species I	Species II	Sex	Matix	Variation PC I	Variation PC II	% PC I	% PC II	% Total
A. sheari	*A. merickeli*	F	Cov	pos: tar, eye2, fem, scu, II	pos: scu; neg: II	79.9	7.7	87.6
A. sheari	*A.* cf. *quattuor*	M	Cor	pos: fem, I, eye, metat, II	pos: eye2, scu	64.9	11.5	76.4
A. sheari	*A.* cf. *quattuor*	F	Cor	pos: fem, tib, metat, tar, eye2	neg: III, IV, metap; pos: eye	61.9	10.7	72.6
A. sheari	*A.* cf. *quattuor*	M	Cov	pos: tar, fem, metat, I	pos: eye2, scu	87.7	4.3	92.0
A. sheari	*A.* cf. *quattuor*	F	Cov	pos: eye2, tar, metat, fem	neg: scu, eye2	74.6	9.8	84.4
A. sheari	*A. cosmetoides*	M	Cor	pos: fem, tar, metat, tib, III	neg: IV, II, III	50.1	11.2	61.3
A. sheari	*A. cosmetoides*	F	Cor	pos: fem, tib, tar, metat	pos: III, II, IV; neg: metat	42.4	17.4	59.8
A. sheari	*A. cosmetoides*	M	Cov	pos: tar, fem, metat, I, III	pos: II, III, IV; neg: tar	66.3	11.3	77.6
A. sheari	*A. cosmetoides*	F	Cov	pos: tar, fem, metat, eye2	neg: metat, tar, fem; pos: II, III	45.3	21.9	67.2
A. quattuor	*A. merickeli*	M	Cor	pos: metat, fem, tar, tib, eye	pos: II, eye2; neg: IV, III	37.1	19.7	56.8
A. quattuor	*A. merickeli*	F	Cor	pos: fem, tib, tar, metat, II	pos: I, eye2, scu; neg: III, II, IV	35.4	15.9	51.3
A. quattuor	*A. merickeli*	M	Cov	pos: tar, metat, fem, eye2	neg: I, eye2	50.4	32.2	82.6
A. quattuor	*A. merickeli*	F	Cov	pos: metat, fem, eye2, tar, tib	neg: I, eye2, scu; pos: tar	47.3	24.5	71.8
A. quattuor	*A.* cf. *quattuor*	M	Cor	pos: fem, eye, metat, I, II, tib	pos: IV, III; neg: car	48.1	11.9	60.0
A. quattuor	*A.* cf. *quattuor*	F	Cor	pos: fem, II, metat, tib, eye2	neg: III, IV	44.0	11.7	55.7
A. quattuor	*A.* cf. *quattuor*	M	Cov	pos: metat, tar, fem, tib, eye	pos: eye; neg: tar, fem, metat	70.0	11.6	81.6
A. quattuor	*A.* cf. *quattuor*	F	Cov	pos: metat, fem, eye2, tar, tib	neg: eye2, scu; pos: tar	56.9	14.0	70.9
A. quattuor	*A. cosmetoides*	M	Cor	pos: tar, fem, metat, tib, eye	pos: III, IV; neg: eye2, car	38.5	17.6	56.1
A. quattuor	*A. cosmetoides*	F	Cor	pos: fem, tib, tar, metat	pos: II, III, eye; neg: met, tar	29.9	16.9	46.8
A. quattuor	*A. cosmetoides*	M	Cov	pos: tar, fem, metat, tib, eye2	pos: eye2, I; neg: III, IV	49.1	21.5	70.6

(table continues)

Table Supplement D.7 Variables Influencing and Percent Variation Explained by Pairwise Principle Components (cont).

Species I	Species II	Sex	Matix	Variation PC I	Variation PC II	% PC I	% PC II	% Total
A. quattuor	*A. cosmetoides*	F	Cov	pos: tar, metat, fem, eye2, I	neg: eye2, I; pos: metat, fem, tar	32.8	25.5	58.3
A. merickeli	*A.* cf. *quattuor*	M	Cor	pos: fem, tib, metat, tar, eye	pos: I; neg III, IV, I, eye	40.5	20.8	61.3
A. merickeli	*A.* cf. *quattuor*	F	Cor	pos: tib, fem, metat, II, eye	pos: eye2, I, scu; neg: III	36.7	14.6	51.2
A. merickeli	*A.* cf. *quattuor*	M	Cov	pos: tar, metat, fem, tib	neg: I, eye2, II, eye	53.8	28.3	82.1
A. merickeli	*A.* cf. *quattuor*	F	Cov	pos: tar, fem, II, metat, tib	pos: I, eye2	36.9	25.3	62.2
A. makah	*A. sheari*	M	Cor	pos: fem, metat, tib, tar, eye	neg: car, metap, tro	67.9	9.7	77.6
A. makah	*A. sheari*	F	Cor	pos: fem, tib, tar, eye, metap	neg: car, IV, scu	58.7	11.3	70.0
A. makah	*A. sheari*	M	Cov	pos: tar, fem, II, metat	neg: II, pos: tar	93.8	2.4	96.2
A. makah	*A. sheari*	F	Cov	pos: fem, tar, metat, II	neg: metat, scu, car	84.7	7.4	92.1
A. merickeli	*A. cosmetoides*	M	Cor	pos: tar, fem, metat, tib, tro	pos: III, I; neg: eye, metap, II	36.2	18.8	54.9
A. merickeli	*A. cosmetoides*	F	Cor	pos: tib, fem, metat, pfem	pos: II, III, eye; neg: metat, tar	26.5	16.1	42.6
A. merickeli	*A. cosmetoides*	M	Cov	pos: tar, metat, fem, I, III	neg: III, I; pos: II, tar	47.1	21.8	68.9
A. merickeli	*A. cosmetoides*	F	Cov	pos: tar, fem, eye2, II, I	pos: III, I; neg: metat, tar	27.0	22.8	49.8
A. cf. *quattuor*	*A. cosmetoides*	M	Cor	pos: fem, tar, tib, tro, II, I	neg: III, IV; pos: eye2	42.0	14.4	56.4
A. cf. *quattuor*	*A. cosmetoides*	F	Cor	pos: fem, tib, metat, tar	pos: II, III, IV; neg: metat, tar	29.6	16.9	46.5
A. cf. *quattuor*	*A. cosmetoides*	M	Cov	pos: tar, fem, metat, I, II	neg: III, IV; pos: eye2	50.3	20.9	71.2
A. cf. *quattuor*	*A. cosmetoides*	F	Cov	pos: tar, fem, metat, III, II	neg: eye2, I	29.2	26.8	55.9
A. makah	*A. quattuor*	M	Cor	neg: fem, tib, eye, II; pos: I	pos: eye2, pfem, car	46.6	20.6	67.2
A. makah	*A. quattuor*	F	Cor	neg: fem, II, tib, eye; pos: I	pos: pfem, eye2, pat, car	37.8	24.1	61.9
A. makah	*A. quattuor*	M	Cov	neg: fem, tar, metat, II	pos: eye2, tar, I	70.7	18.5	89.2

(table continues)

Table Supplement D.7 Variables Influencing and Percent Variation Explained by Pairwise Principle Components (cont).

Species I	Species II	Sex	Matix	Variation PC I	Variation PC II	% PC I	% PC II	% Total
A. makah	*A. quattuor*	F	Cov	pos: fem, II, metat, tib, tar	pos: scu, eye2, I, metat	61.5	21.2	82.7
A. makah	*A. merickeli*	M	Cor	pos: fem, tib, eye, metat	pos: car, pfem, scu, met, tar	43.6	19.0	62.6
A. makah	*A. merickeli*	F	Cor	neg: fem, eye, tib, II	pos: IV, pfem, car, metat	34.7	17.0	51.7
A. makah	*A. merickeli*	M	Cov	pos: fem, tar, metat	neg: fem; pos: scu	73.6	13.0	86.6
A. makah	*A. merickeli*	F	Cov	pos: metat, tib, II, eye	pos: scu, metat, tar	54.7	18.4	73.2
A. makah	*A.* cf. *quattuor*	M	Cor	pos: fem, eye, II, metat	pos: pfem, tib, metap	39.4	26.5	65.9
A. makah	*A.* cf. *quattuor*	F	Cor	pos: fem, tib, eye, II, metat	pos: pfem, IV, I, metap, scu	39.1	20.3	59.4
A. makah	*A.* cf. *quattuor*	M	Cov	pos: fem, II, tar, metat	pos: I, tar, metat, eye2, scu	69.8	18.5	88.3
A. makah	*A.* cf. *quattuor*	F	Cov	pos: fem, eye, tib, metat	pos: scu, metat, I, tar	64.8	14.0	78.8
A. makah	*A. cosmetoides*	M	Cor	pos: fem, tar, tib, eye	pos: I, car, II	35.9	23.6	59.5
A. makah	*A. cosmetoides*	F	Cor	pos: fem, tib, eye, tar, metat	pos: scu, I, pfem, pat	29.2	17.7	46.9
A. makah	*A. cosmetoides*	M	Cov	pos: tib, II, tar, eye	pos: I, III	51.9	21.0	72.9
A. makah	*A. cosmetoides*	F	Cov	pos: fem, tar, eye, tib, metat	pos: eye2, I, III, II	37.9	19.7	57.6
A. leonardi	*A. sheari*	M	Cor	pos: tar, fem, metat, I, eye	neg: scu; pos: car, pat, tro	51.7	16.8	68.5
A. leonardi	*A. sheari*	F	Cor	pos: tib, tar, eye2, metat, fem	neg: I, car, IV	64.6	19.9	84.5
A. leonardi	*A. sheari*	M	Cov	pos: tar, fem, metat, eye	pos: eye2, metat	93.8	2.7	96.5
A. leonardi	*A. sheari*	F	Cov	pos: fem, tar, metat, II	pos: scu, car; neg: II	87.7	6.8	94.5
A. leonardi	*A. quattuor*	M	Cor	neg: fem, eye, tib, II, tar;	pos: eye2, par, met, car, pfem	35.7	29.1	64.8
A. leonardi	*A. quattuor*	F	Cor	pos: pat, eye2, metat, pfem	pos: tro, I; neg: II, eye, tar, metat	35.9	25.2	61.1
A. leonardi	*A. quattuor*	M	Cov	neg: tar, fem, metat; pos: I, scu	pos: eye2, I, metat, tar	48.4	38.9	87.3
A. leonardi	*A. quattuor*	F	Cov	pos: fem, metat, eye2, tib, II	pos: scu, eye2, I; neg: fem, II	51.4	28.4	79.8
A. leonardi	*A. merickeli*	M	Cor	pos: metat, tar, eye, tib, II	pos: scu, pfem, met, III	31.2	25.8	57.0
A. leonardi	*A. merickeli*	F	Cor	pos: tib, metat, tar, fem, II, eye	pos: scu, tro; neg: eye, II	26.4	21.3	47.7
A. leonardi	*A. merickeli*	M	Cov	pos: tar, metat, fem, tib	pos: scu, eye2; neg: fem	61.6	18.8	80.4
A. leonardi	*A. merickeli*	F	Cov	pos: fem, metat, tar	pos: scu, eye2	40.2	27.8	68.0

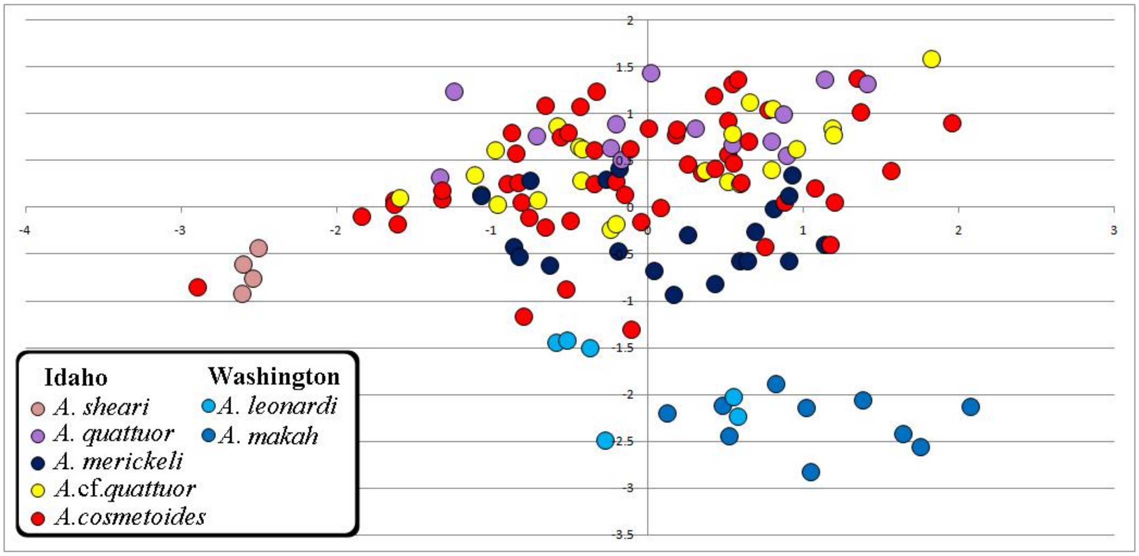

Figure Supplement D.22. Nested PCA – all males: correlation matrix. Plotting the two principle components accounting for the greatest percentage of variation shows WA groups to the exclusion of ID, and that *A. sheari* clusters. The first component (35.6% of the variation) is positively weighted for leg segment lengths, ocularium height, and scute area II spine height. The second component (17.5%) is positively weighted on eye to area II spine distance and scute area I spine height, and negatively weighted on ocularium and area II spine height.

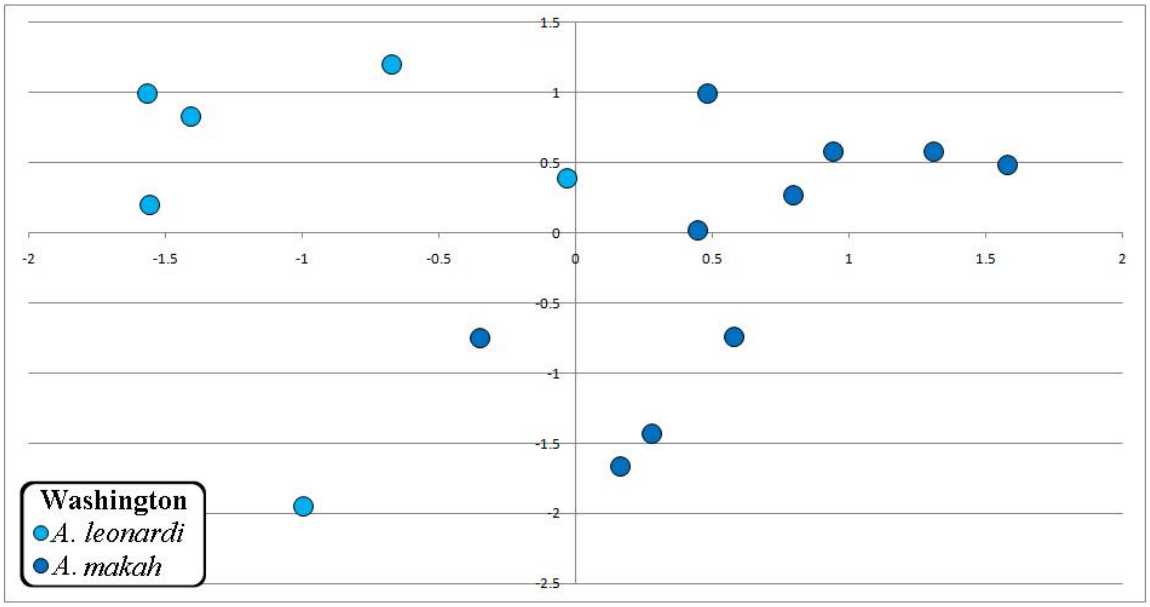

Figure Supplement D.23. Nested PCA – *A. makah*, *A. leonardi* males: correlation matrix. Plotting the two principle components accounting for most of variation shows that species from WA occupy a disparate morphospace. The first component (67.2%) is positively weighted for leg II femur, metatarsus, tarsus, and scute area II spine height. The second component (13.6%) is positively weighted on leg II tarsus and negatively weighted on scute area II spine height.

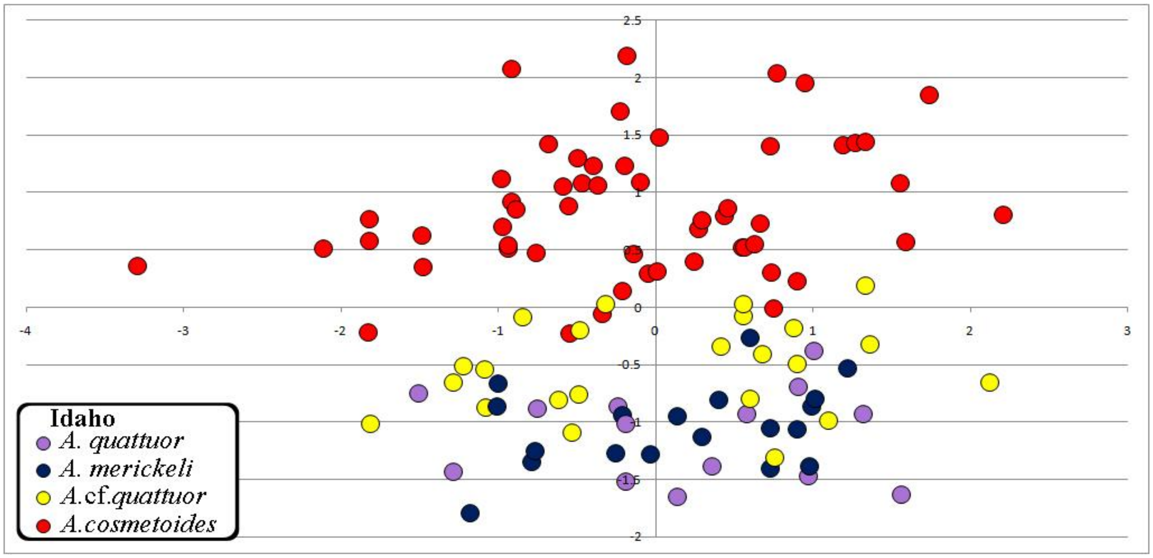

Figure Supplement D.24. Nested PCA – Idaho males no *A. sheari*: correlation matrix. Plotting the two principle components accounting for the greatest percentage of variation does not discretely cluster any hypothesized species, though nearly *A. cosmetoides*. The first component (36.7% of the variation) is positively weighted for leg segment lengths, ocularium height, and scute area II spine height. The second component (16.5%) is positively weighted on scute area III and IV spine height, and negatively weighted on carapace and the distance from the eye spine to scute area II spine.

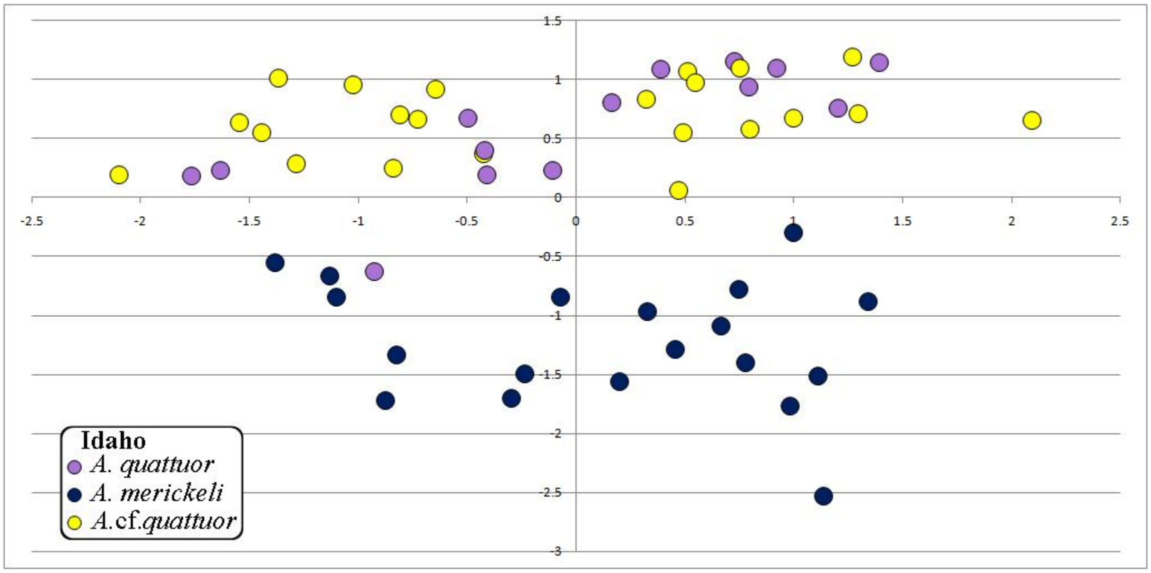

Figure Supplement D.25. Nested PCA – *A. quattuor*, *A. merickeli*, *A.* cf. *quattuor* males: correlation matrix. Plotting the two principle components accounting for most of variation shows that *A. merickeli* is distinct from *A. quattuor* and *A.* cf. *quattuor*. The first component (38.5%) is positively weighted for leg II femur, metatarsus, tibia, and ocularium height. The second component (13.6%) is positively weighted on scute area I spine, the distance from the eye spine to scute area II spine, and negative for scutal spines III and IV.

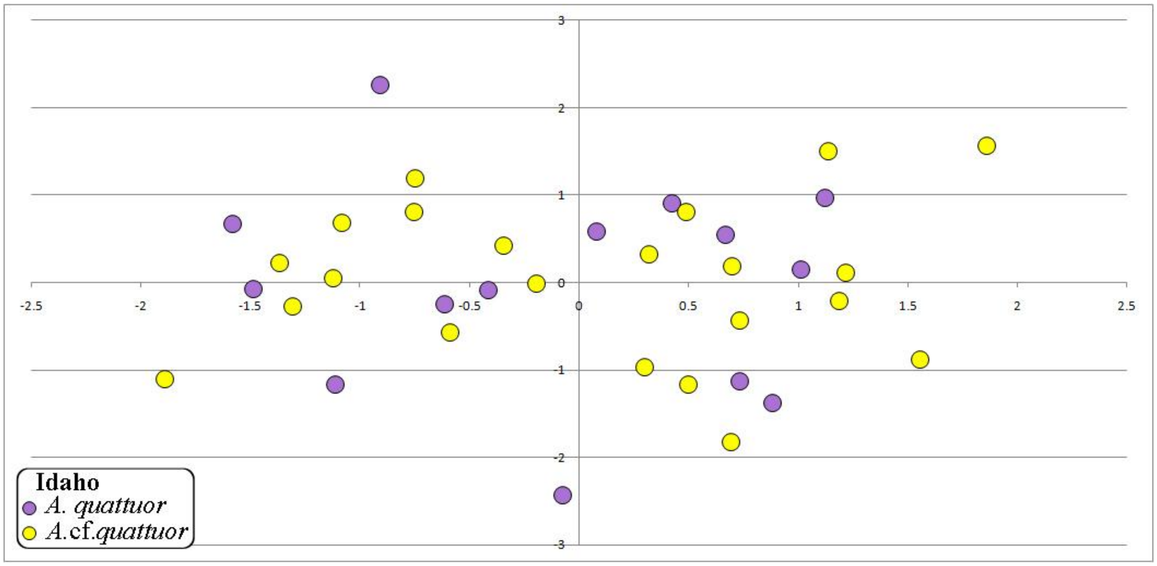

Figure Supplement D.26. Nested PCA – *A. quattuor*, *A.* cf. *quattuor* males: correlation matrix. Plotting the two principle components accounting for the greatest percentage of variation does not discretely cluster the two hypothesized species. The first component (48.1% of the variation) is positively weighted for leg segment lengths, ocularium height, and scute area I and II spine height. The second component (12.0%) is positively weighted on scute area IV and III spine height, and negatively weighted on carapace.

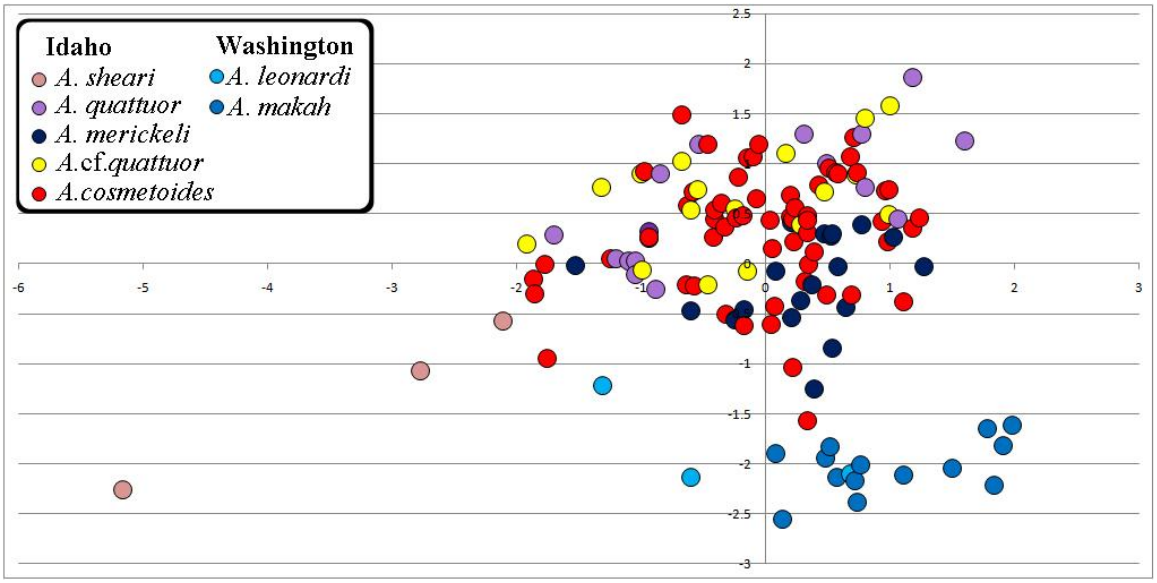

Figure Supplement D.27. Nested PCA – all females: correlation matrix. Plotting the two principle components accounting for the greatest percentage of variation shows WA groups to the exclusion of ID, and that *A. sheari* is discrete. The first component (32.1%) is positively weighted for all leg lengths, scute area II spine height, and ocularium height. The second component (16.6%) is positively weighted on scute length, scute area I spine height, and negatively weighted on ocularium height.

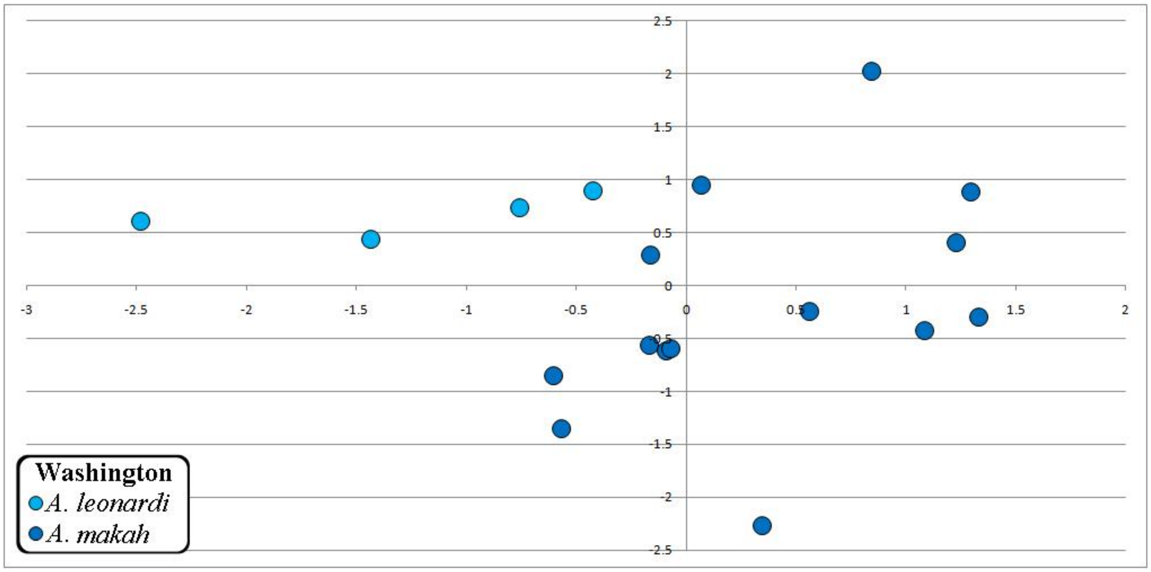

Figure Supplement D.28. Nested PCA – *A. makah, A. leonardi* females: correlation matrix. Plotting the two principle components accounting for the greatest percentage of variation does finds the two hypothesized Washington species as discrete cluster in morphospace. The first component (37.4% of the variation) is positively weighted for leg II tibia, femur, patella, and scute area II spine height. The second component (14.8%) is positively weighted on carapace and metatarsus, and negatively weighted on scute area I spine and the distance from the eye spine to the scute area II spine.

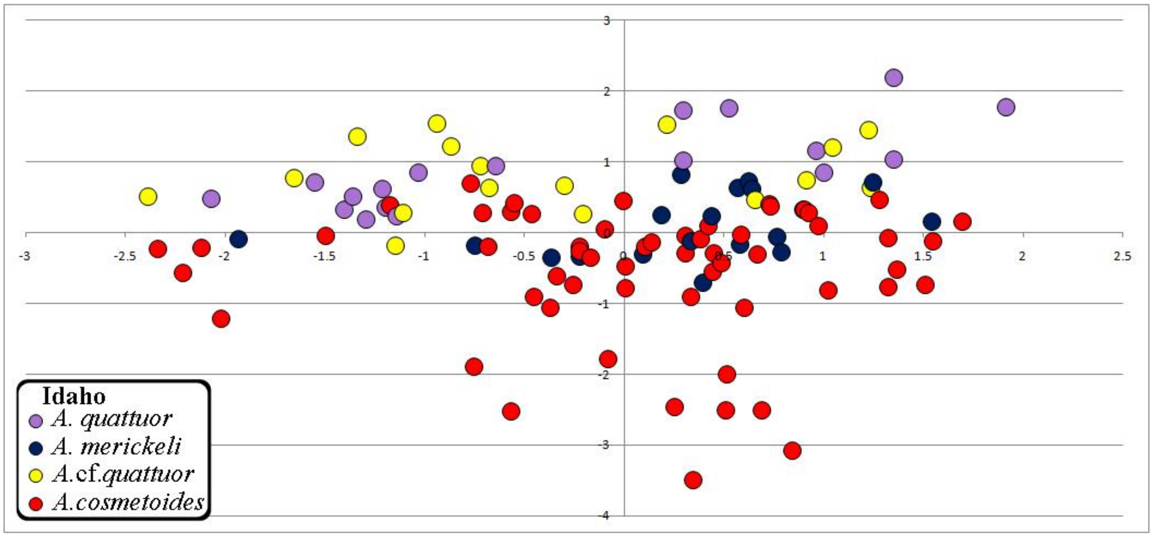

Figure Supplement D.29. Nested PCA – Idaho females no *A. sheari*: correlation matrix. Plotting the two principle components accounting for the greatest percentage of variation does not discretely cluster any hypothesized species. The first component (29.1%) is positively weighted for leg segment lengths and scute areas IV and I spine height. The second component (13.5%) is positively weighted on the eye spine to scute area II spine , and negatively on scute areas IV and III spine height.

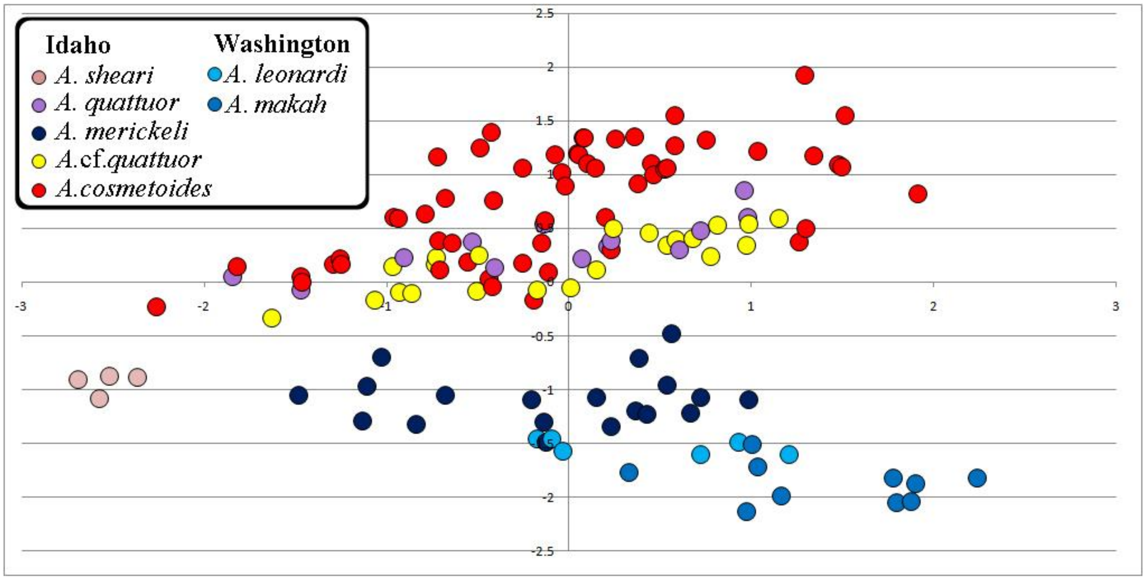

Figure Supplement D.30. Nested PCA – all males: covariance matrix. Plotting the two principle components accounting for the greatest percentage of variation shows three discrete groups of hypothesized species: WA groups with *A. merickeli*, *A. sheari*, and the remaining ID species. The first component (48.1% of the variation) is positively weighted for leg segment lengths and scute area II spine height. The second component (19.5%) is positively weighted on scute area I and III spines.

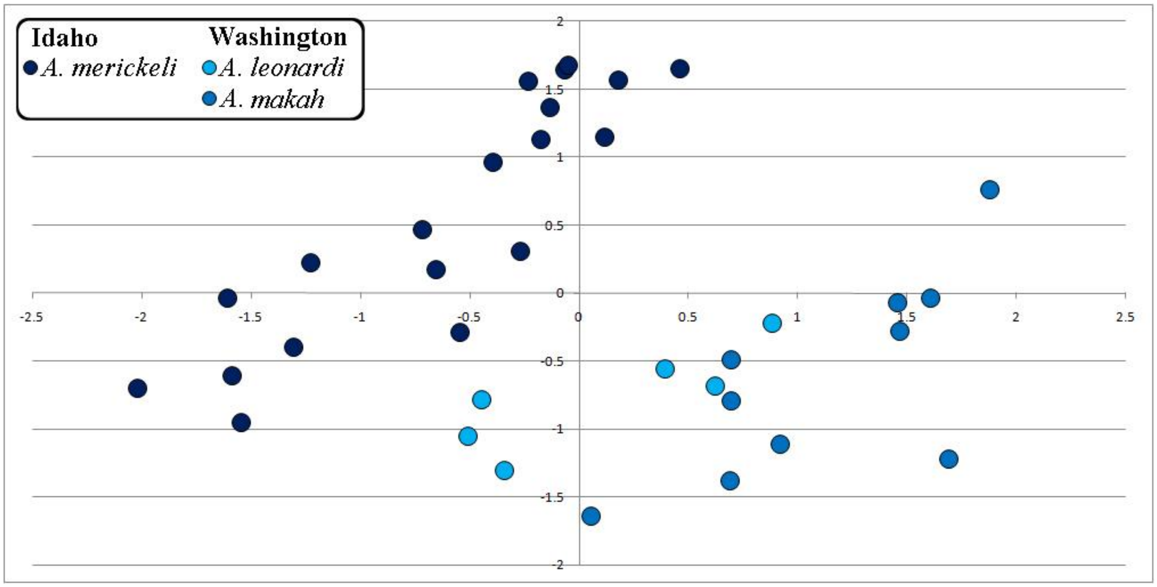

Figure Supplement D.31. Nested PCA – *A. merickeli* and WA males: covariance matrix. Plotting the two principle components accounting for the greatest percentage of variation shows that *A. merickeli* is discrete from WA populations. The first component (69.3%) is positively weighted for Leg II lengths, and eye spine and area II spine height. The second component (12.7%) is positively weighted on scute length, and negatively weighted for Leg II femur length.

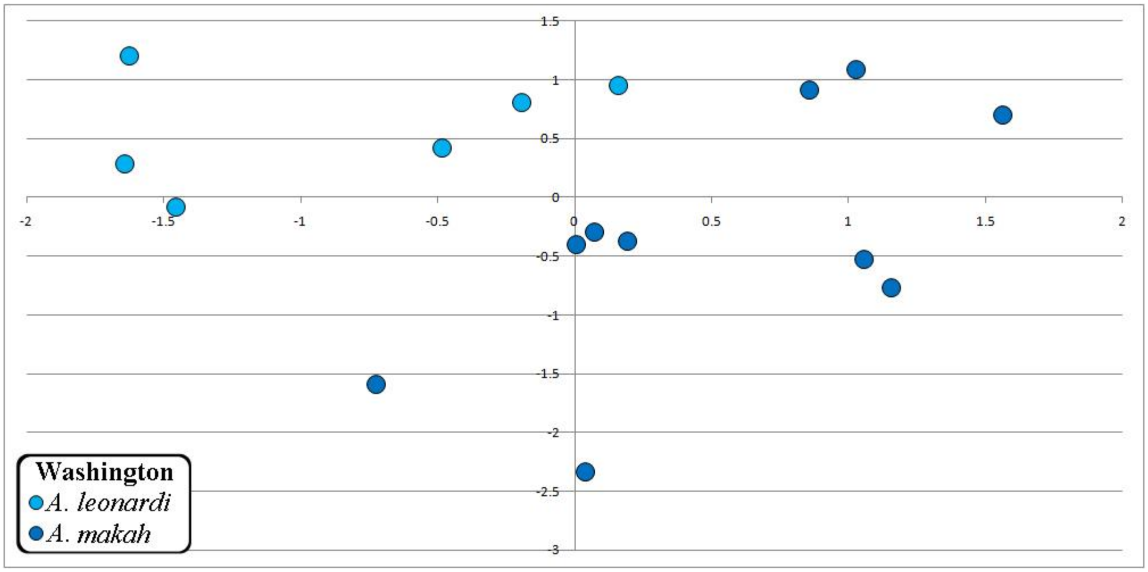

Figure Supplement D.32. Nested PCA – *A. makah*, *A. leonardi* males: covariance matrix. Plotting the two principle components accounting for the greatest percentage of variation shows that WA populations occupy disparate morphospace. The first component (67.2% of the variation), is positively weighted for leg II femur, metatarsus, and tarsus, and scute area II spine height. The second component (13.6%) is positively weighted on leg II tarsus, and negatively weighted on scute area II spine.

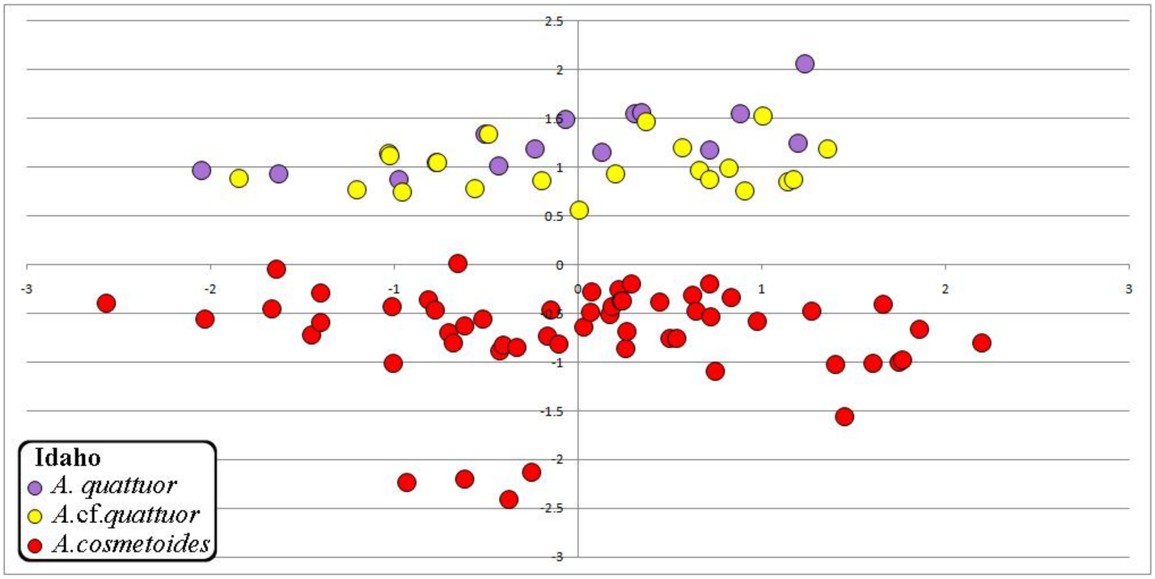

Figure Supplement D.33. Nested PCA – *A. quattuor*, *A.* cf. *quattuor*, *A. cosmetoides* males: covariance matrix. Plotting the two principle components accounting for the greatest percentage of variation shows that *A. cosmetoides* is discrete from *A. quattuor* and *A.* cf. *quattuor*. The first component, (48.0%) of the variation, is positively weighted for leg II lengths and the distance from the eye spine to area II spine. The second component (24.3%) is negatively weighted on scute areas III and IV spine height.

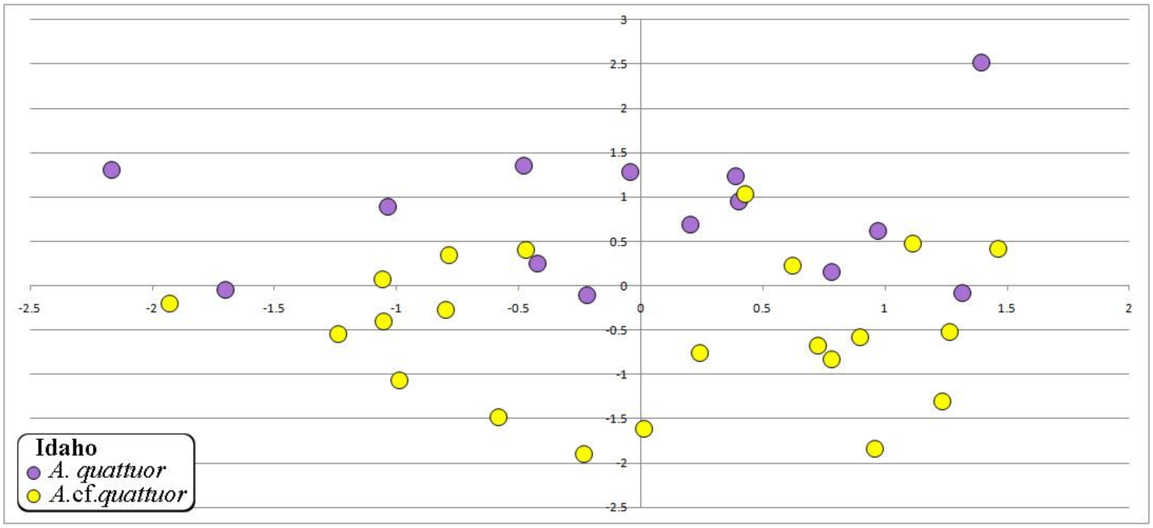

Figure Supplement D.34. Nested PCA – *A. quattuor, A.* cf. *quattuor* males: covariance matrix. Plotting the two principle components accounting for the greatest percentage of variation does not discretely cluster the two hypothesized species. The first component (70.0% of the variation) is positively weighted for leg segment lengths, ocularium height, and scute area II and III spine height. The second component (11.6%) is positively weighted on ocularium height, and negatively weighted on leg II lengths.

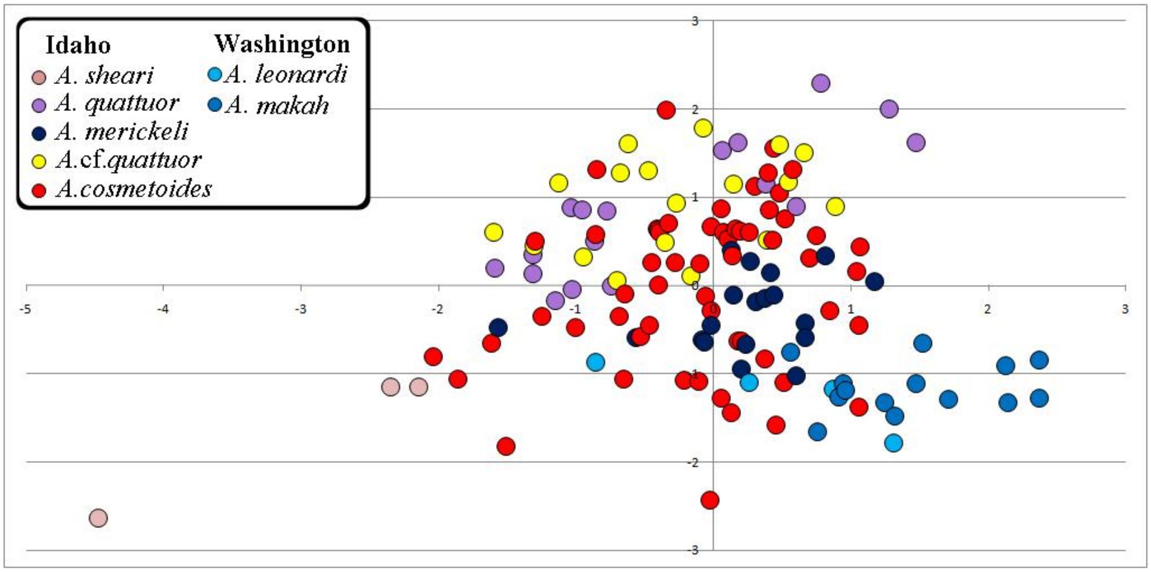

Figure Supplement D.35. Nested PCA – all females: covariance matrix. Plotting the two principle components accounting for the greatest percentage of variation recovers only *A. sheari* as discrete. The first component (39.2%) is positively weighted for all leg II tibia, femur, tarsus, metatarsus, and scute area II spine height. The second component (19.5%) is positively weighted on the length from the eye spine to scute area II spine, scute area I spine height, and scute length.

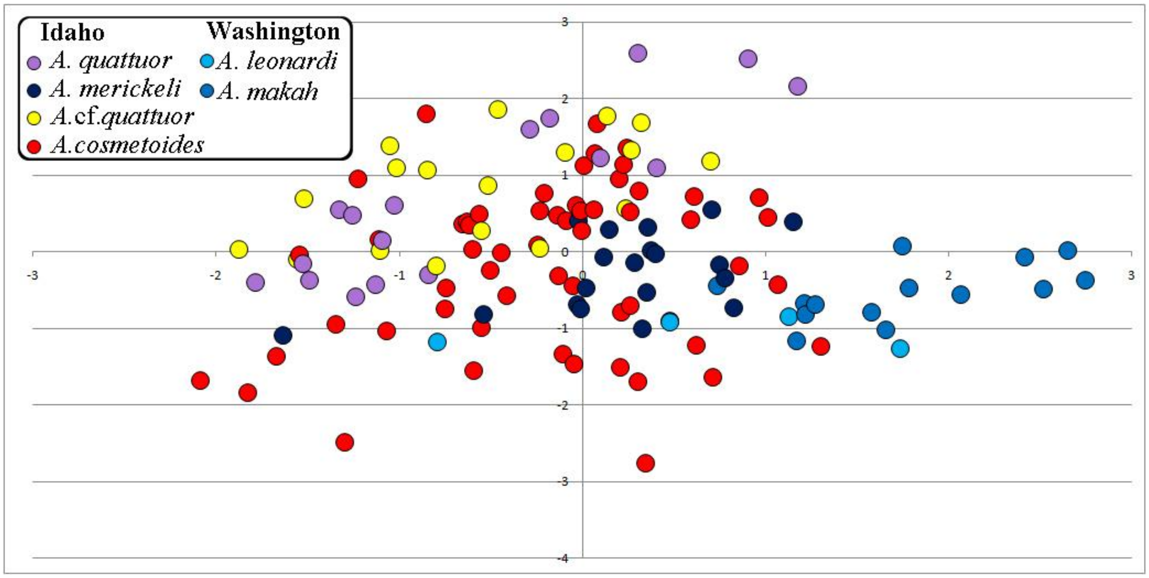

Figure Supplement D.36. Nested PCA – females, no *A. sheari*: covariance matrix. Plotting the two principle components accounting for the greatest percentage of variation does not further cluster hypothesized species discretely. The first component (35.1%) is positively weighted for eye spine and scute area II spine height, and leg segment lengths. The second component (19.4%) is positively weighted on scute length and scute area I spine height.

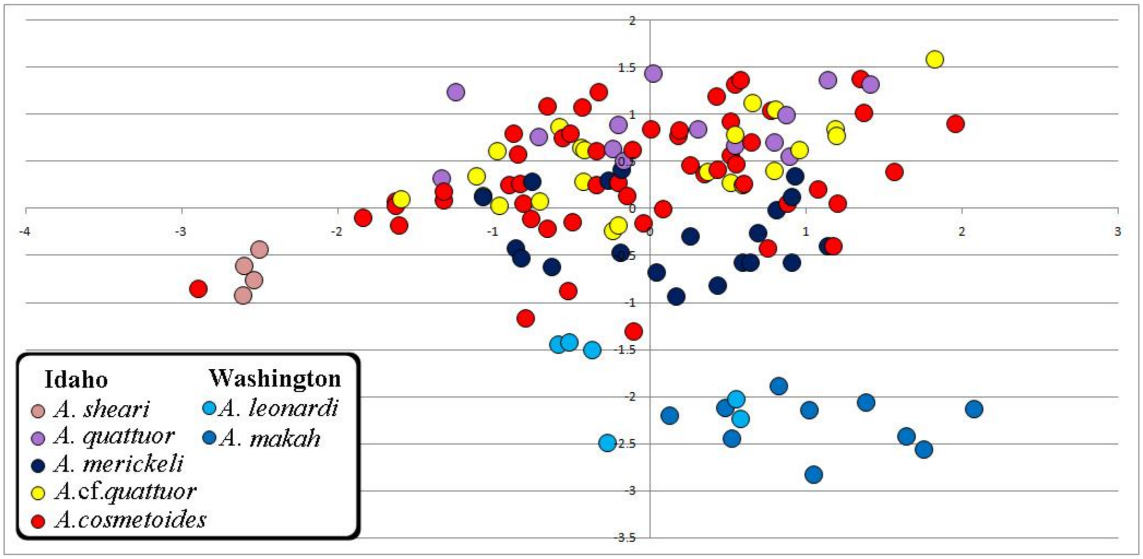

Figure Supplement D.37. Exhaustive components PCA: 1 and 2 – males: correlation matrix. Plotting the two principle components 1 and 2 shows WA groups to the exclusion of ID, and that *A. sheari* clusters. The first component (35.6% of the variation) is positively weighted for leg segment lengths, eye spine height, and scute area II spine height. The second component (17.5%) is positively weighted on eye to area II spine distance and scute area I spine height, and negatively weighted on eye spine and area II spine height.

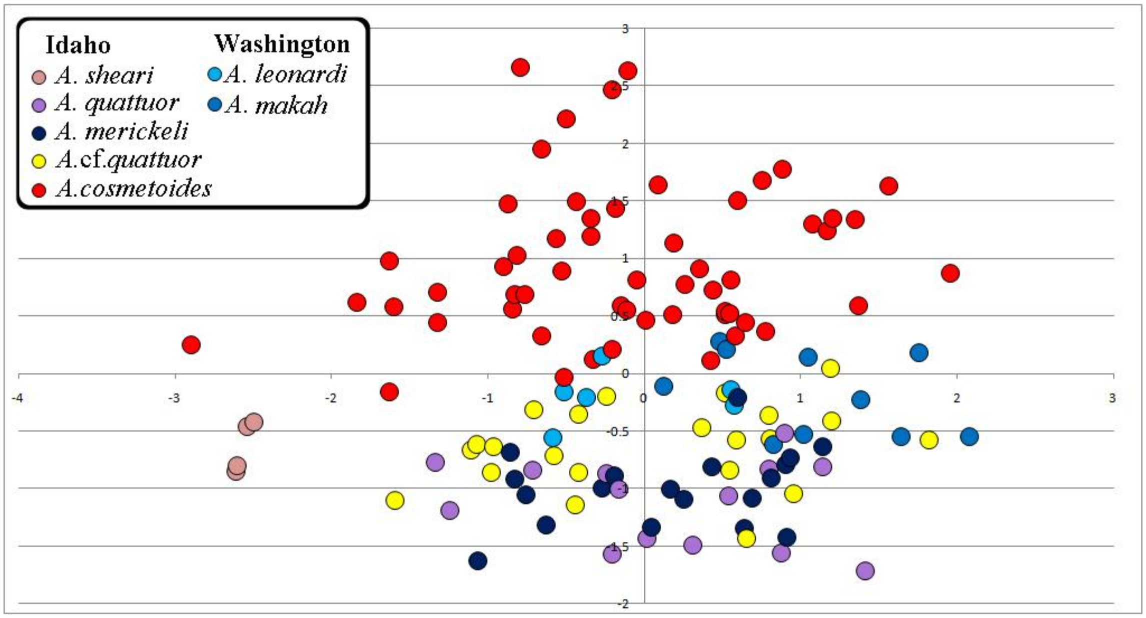

Figure Supplement D.38. Exhaustive components PCA: 1 and 3 – males: correlation matrix. Plotting the two principle components 1 and 3 shows that *A. sheari* clusters and *A. cosmetoides* nearly clusters. The first component (35.6% of the variation) is positively weighted for leg segment lengths, ocularium height, and scute area II spine height. The third component (13.6%) is positively weighted on scute area III and IV spine height, and negative for ocularium height.

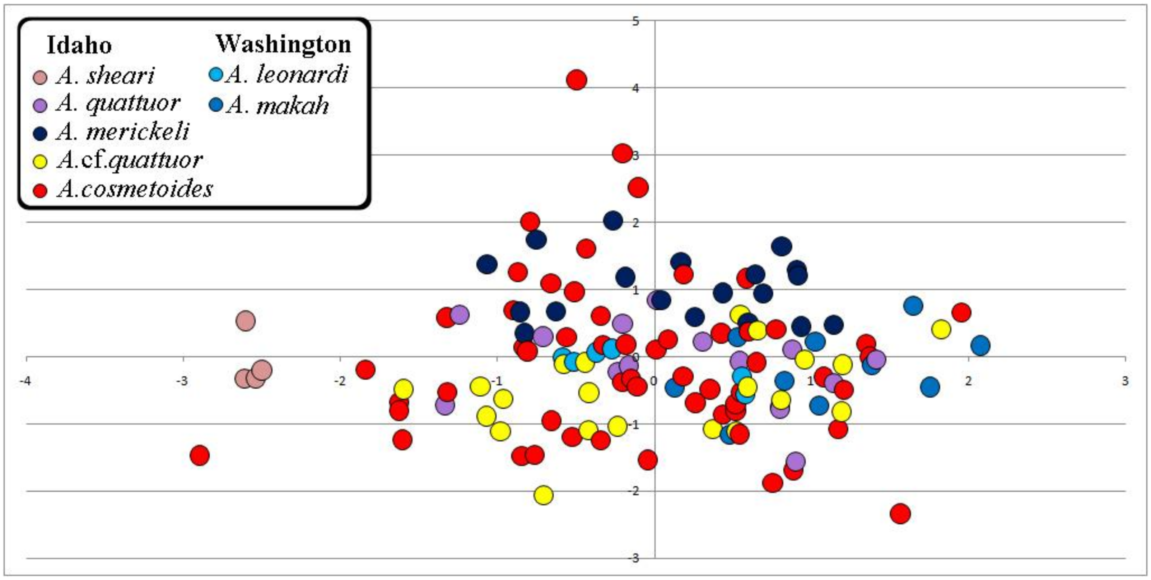

Figure Supplement D.39. Exhaustive components PCA: 1 and 4 – males: correlation matrix. Plotting the two principle components 1 (information above) and 4 recovers only *A. sheari* as discrete. The fourth component (7.0%) is positively weighted on scute area IV and II spine height.

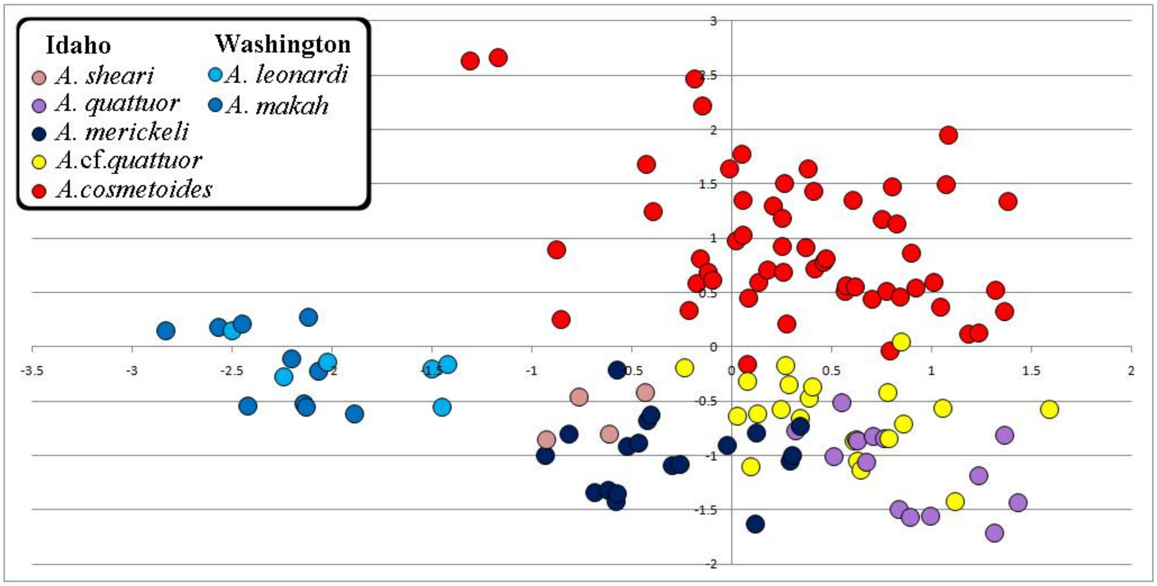

Figure Supplement D.40. Exhaustive components PCA: 2 and 3 – males: correlation matrix. Plotting the principle components 2 and 3 shows that WA and ID species occupy different morphospace and, within Idaho, *A. cosmetoides* nearly clusters. The second component (17.5%) is positively weighted on eye to area II spine distance and scute area I spine height, and negatively weighted on eye spine and area II spine height. The third component (13.6%) is positively weighted on scute area III and IV spine height, and negative for ocularium height.

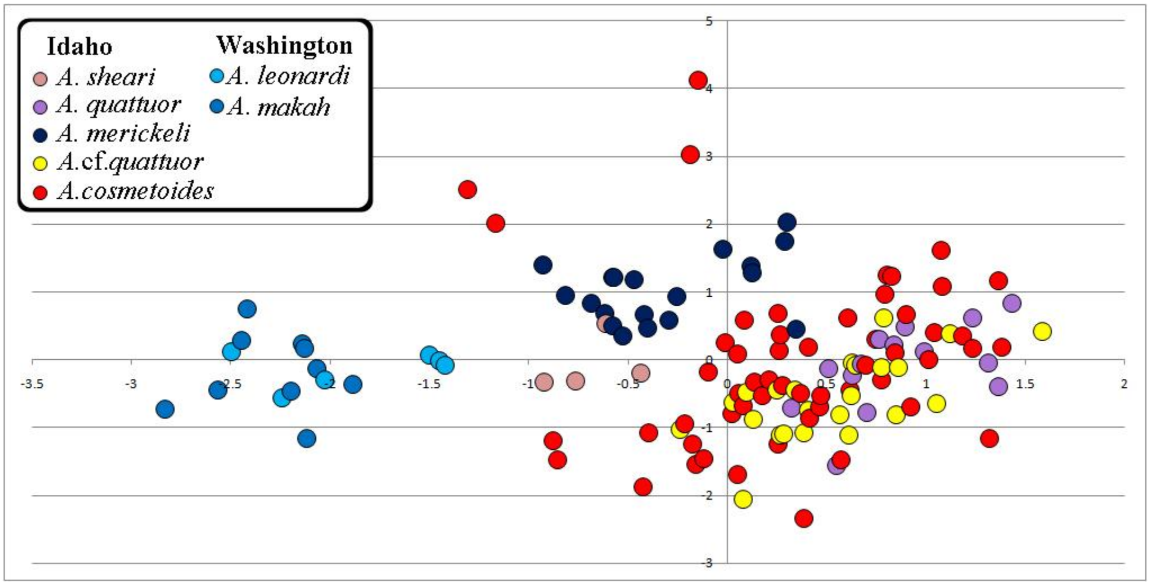

Figure Supplement D.41. Exhaustive components PCA: 2 and 4 – males: correlation matrix. Plotting the principle components 2 (information above) and 4 recovers WA and ID species as distinct. Within ID, *A. sheari* and *A. merickeli* are nearly discrete. The fourth component (7.0%) is positively weighted on scute area IV and II spine height.

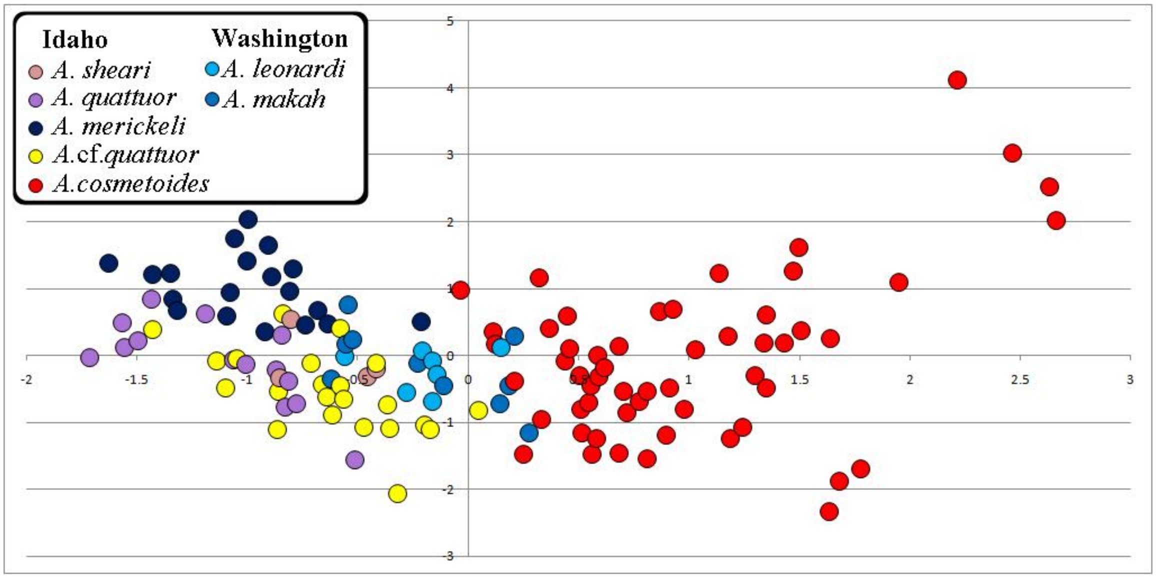

Figure Supplement D.42. Exhaustive components PCA: 3 and 4 – males: correlation matrix. Plotting principle components 3 and 4 does not discretely recover any hypothesized species, but *A. merickeli* and *A. cosmetoides* nearly cluster, as do the WA species. The third component (accounting for 13.6% of the variation) is positively weighted on scute area III and IV spine height, and negative for ocularium height. The fourth component (7.0%) is positively weighted on scute area IV and II spine height.

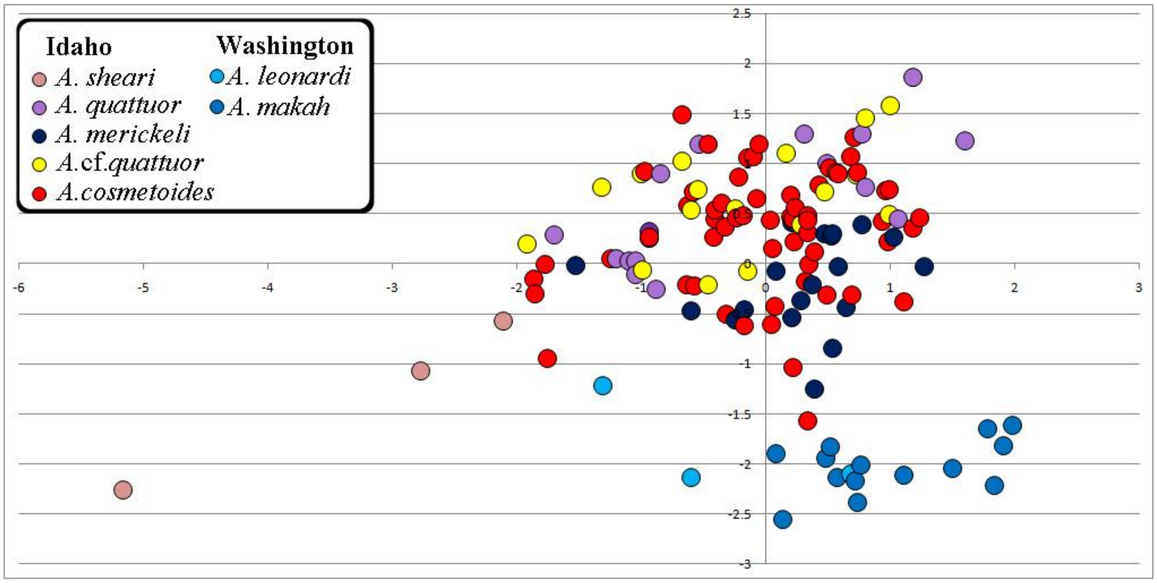

Figure Supplement D.43. Exhaustive components PCA: 1 and 2 – females: correlation matrix. Plotting principle components 1 and 2 shows WA groups to the exclusion of ID, and that *A. sheari* is discrete. The first component (32.1%) is positively weighted for all leg lengths, scute area II spine height, and ocularium height. The second component (16.6%) is positively weighted on scute length, scute area I spine height, and negatively weighted on ocularium height.

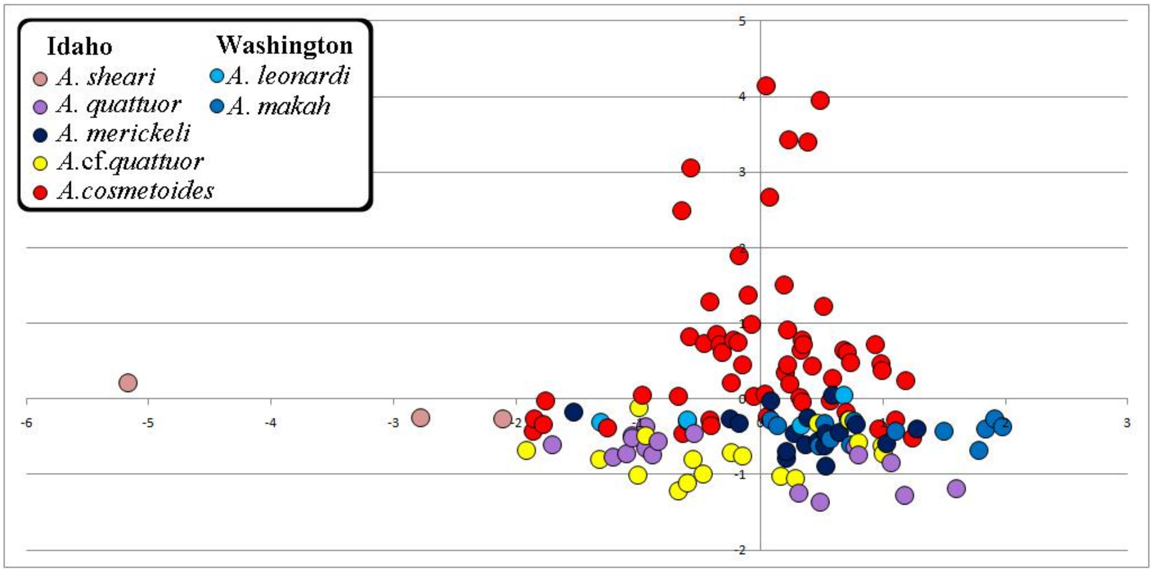

Figure Supplement D.44. Exhaustive components PCA: 1 and 3 – females: correlation matrix. Plotting principle components 1 recovers *A. sheari* as discrete, while *A. cosmetoides* dominates the variation in component 3. The first component (32.1% of the variation) is positively weighted for all leg lengths, scute area II spine height, and ocularium height. The third component (11.3%) is positively weighted on scute area IV and III spine height, and negative for the distance from the eye spine to scute area II spine.

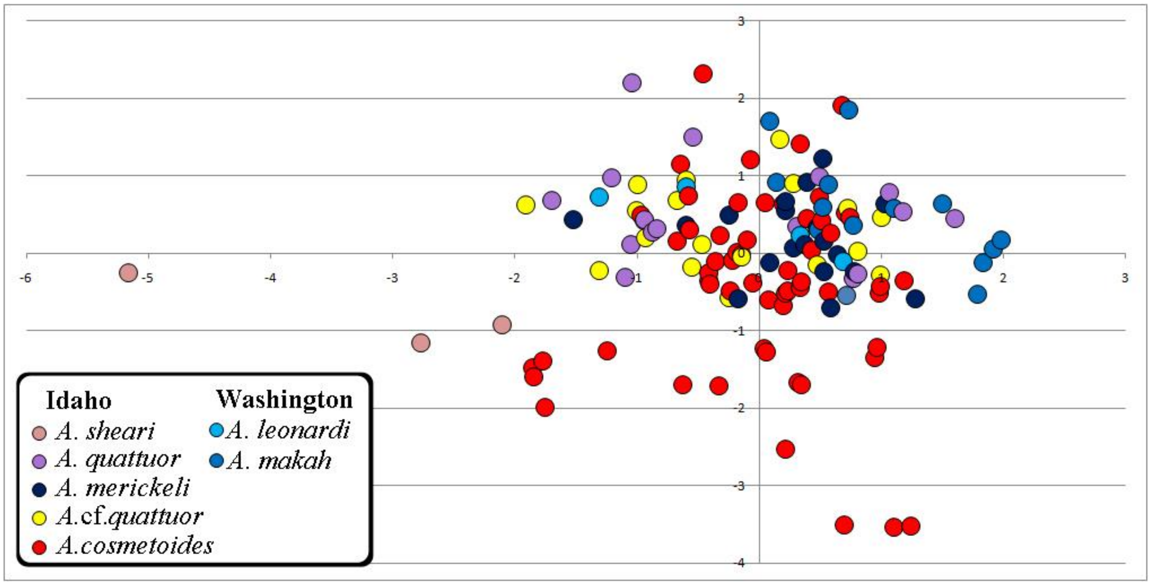

Figure Supplement D.45. Exhaustive components PCA: 1 and 4 – females: correlation matrix. Plotting principle components 1 (information above) and 4 shows that *A. sheari* is discrete. The fourth component (8.6%) is positively weighted on scute area II spine height, on ocularium height, and the distance from the eye spine to area II spine.

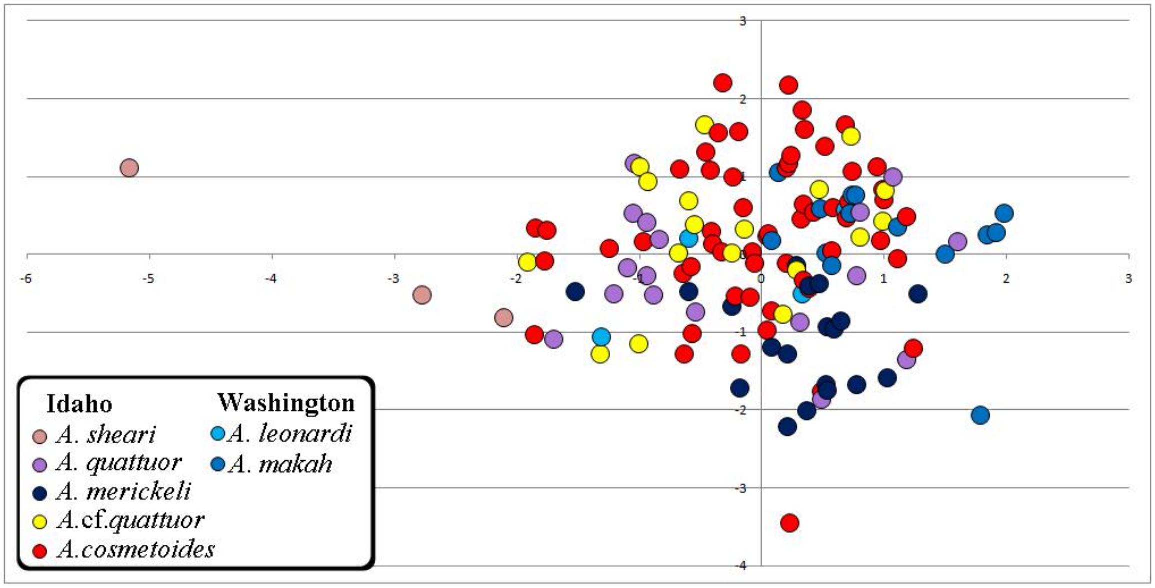

Figure Supplement D.46. Exhaustive components PCA: 1 and 5 – females: correlation matrix. Plotting principle components 1 and 5 recovers *A. sheari* as discrete, which is found by the first component. The first component (32.1% of the variation) is positively weighted for all leg lengths, scute area II spine height, and ocularium height. The fifth component (6.9%) is negatively weighted on carapace length, and positively weighted on scute area I spine height.

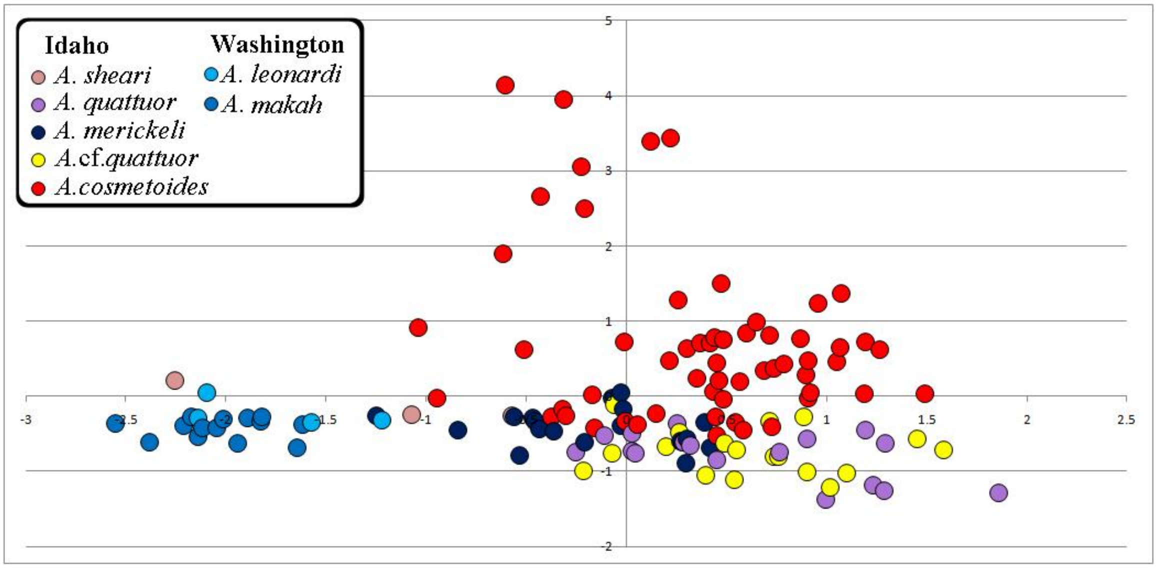

Figure Supplement D.47. Exhaustive components PCA: 2 and 3 – females: correlation matrix. Plotting principle components 2 and 3 does not recover any hypothesized species, though WA species nearly cluster. The second component (16.6%) is positively weighted on scute length, scute area I spine height, and negatively weighted on ocularium height. The third component (11.3%) is positively weighted on scute area IV and III spine height, and negative for the distance from the eye spine to scute area II spine.

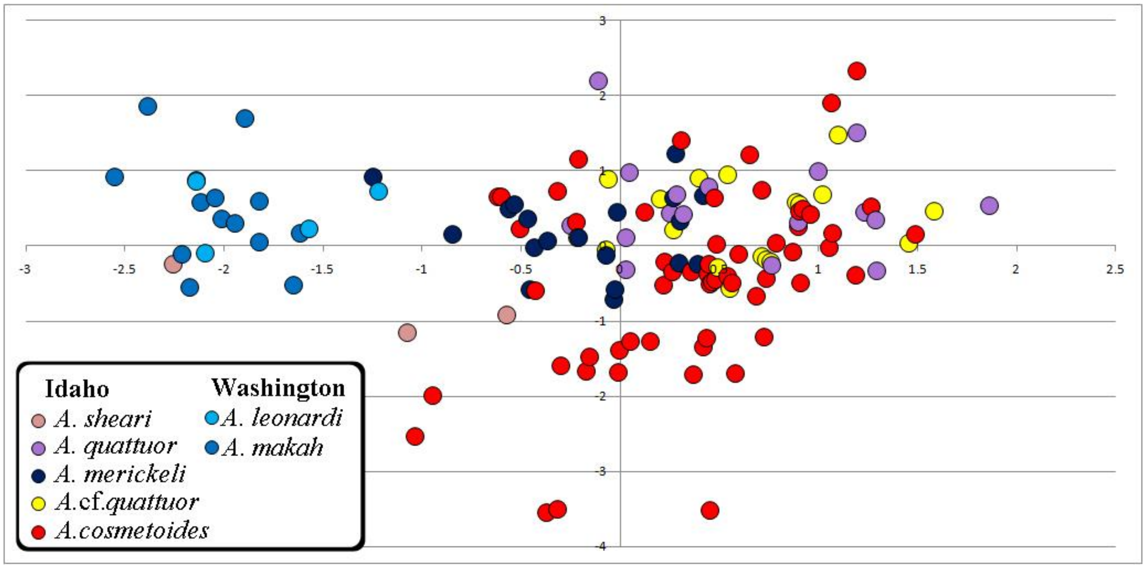

Figure Supplement D.48. Exhaustive components PCA: 2 and 4 – females: correlation matrix. Plotting principle components 2 and 3 does not recover any hypothesized species, though WA species nearly cluster. The second component (accounting for 16.6% of the variation) is positively weighted on scute length, scute area I spine height, and negatively weighted on ocularium height. The fourth component (8.6%) is positively weighted on scute area II spine height, ocularium height, and distance from eye spine to area II spine.

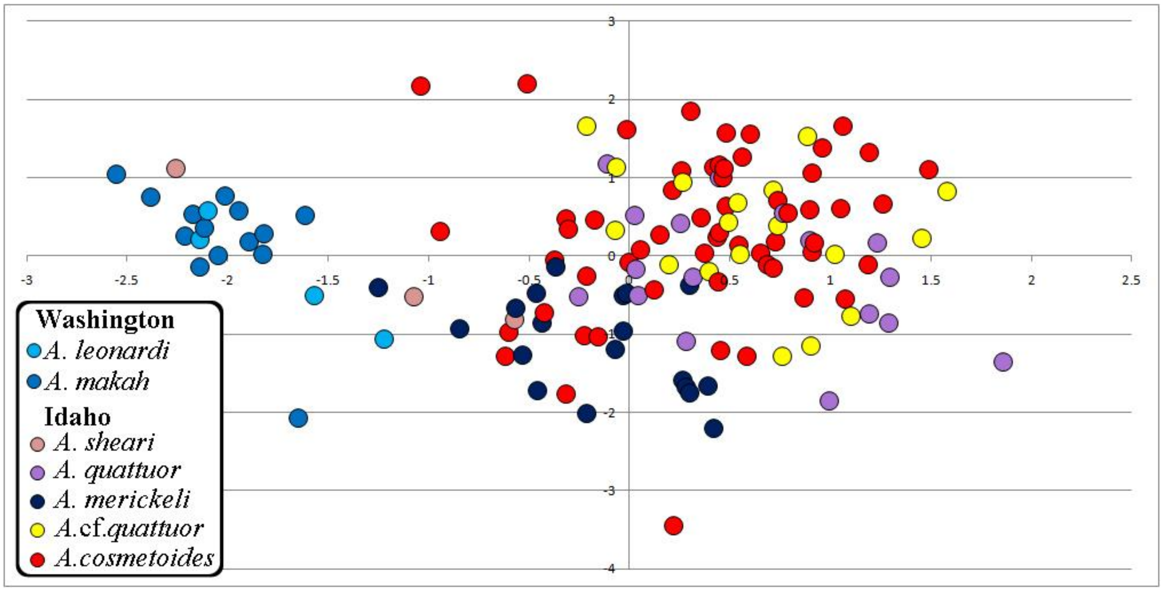

Figure Supplement D.49. Exhaustive components PCA: 2 and 5 – females: correlation matrix. Plotting principle components 2 (information above) and 5 does not recover any hypothesized species, though WA species cluster. The fifth component (6.9%) is negatively weighted on carapace length, and positively weighted on scute area I spine height.

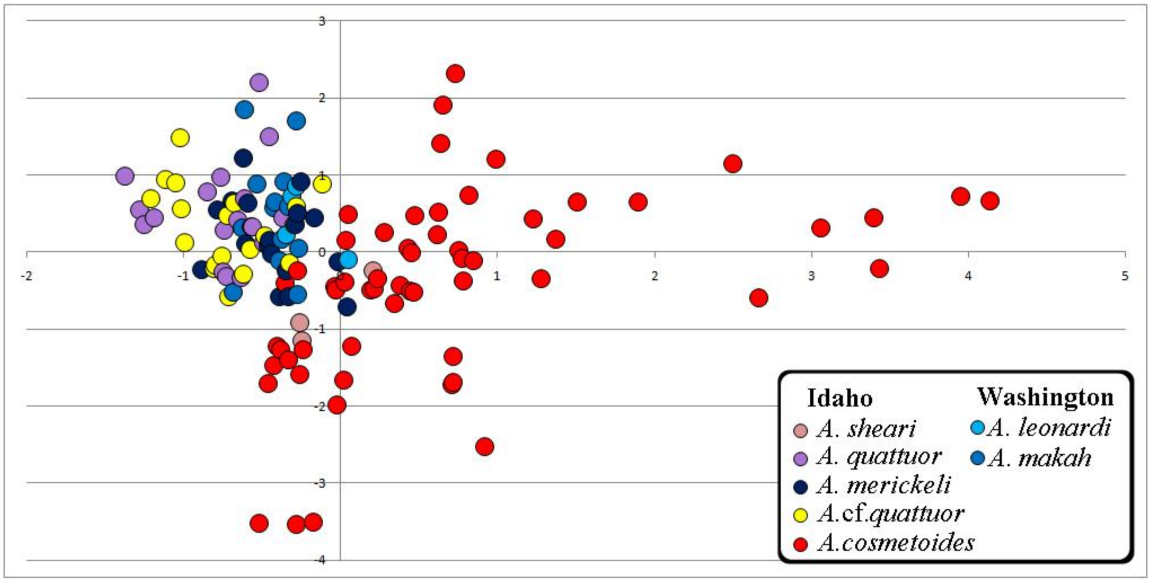

Figure Supplement D.50. Exhaustive components PCA: 3 and 4 – females: correlation matrix. Plotting principle components 3 and 4 does not recover any hypothesized species, though *A. cosmetoides* nearly clusters. The third component (accounting for 11.3% of the variation) is positively weighted on scute area IV and III spine height, and negative for distance from eye spine to scute area II spine. The fourth component (8.6%) is positively weighted on scute area II and ocularium height, and the eye spine to area II spine distance.

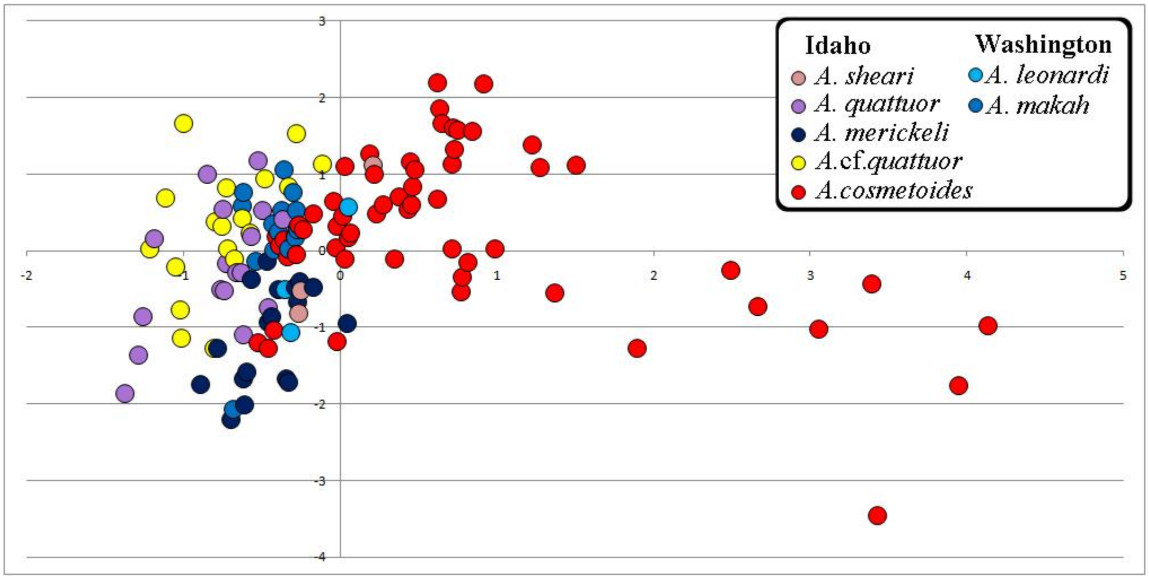

Figure Supplement D.51. Exhaustive components PCA: 3 and 5 – females: correlation matrix. Plotting principle components 3 (information above) and 5 does not recover any hypothesized species. The fifth component (6.9%) is negatively weighted on carapace length, and positively weighted on scute area I spine height.

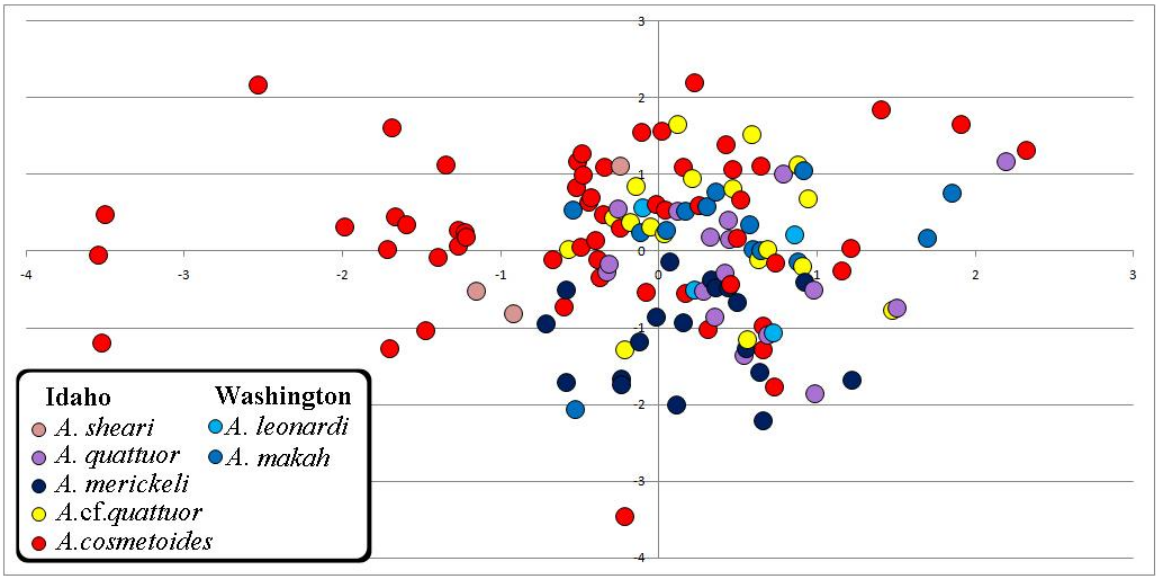

Figure Supplement D.52. Exhaustive components PCA: 4 and 5 – females: correlation matrix. Plotting principle components 4 and 5 does not recover any hypothesized species. The fourth component (accounting for 8.6% of the variation) is positively weighted on scute area II and ocularium height, and the eye spine to area II spine distance. The fifth component (6.9%) is negatively weighted on carapace length, and positively weighted on scute area I spine height.

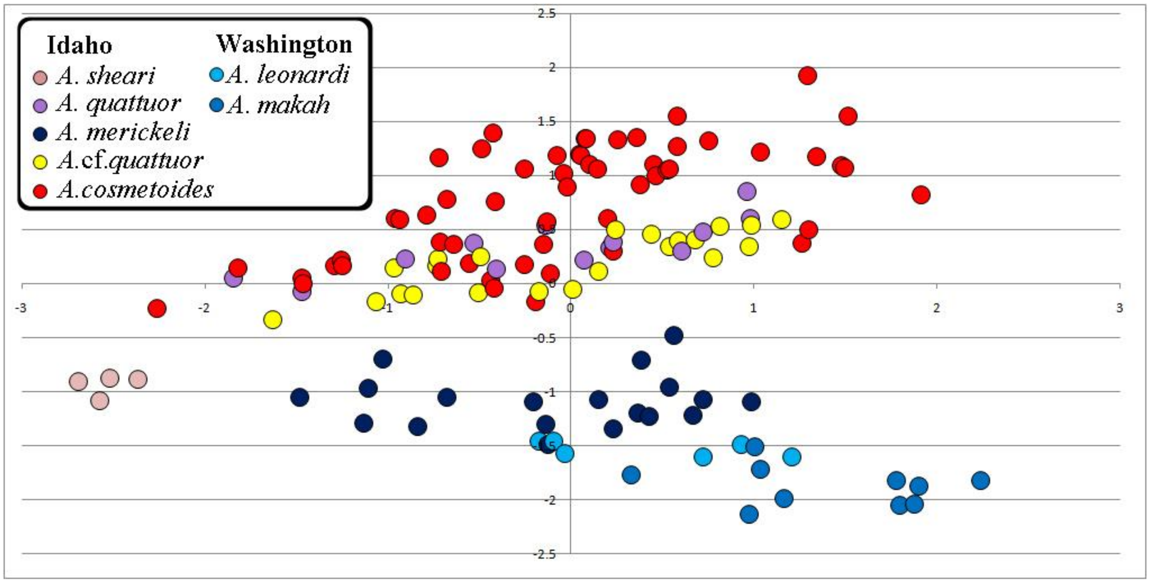

Figure Supplement D.53. Exhaustive components PCA: 1 and 2 – males: covariance matrix. Plotting principle components 1 and 2 shows three discrete groups of hypothesized species: WA groups with *A. merickeli*, *A. sheari*, and the remaining ID species. The first component (48.1% of the variation) is positively weighted for leg segment lengths and scute area II spine height. The second component (19.5%) is positively weighted on scute area I and III spines.

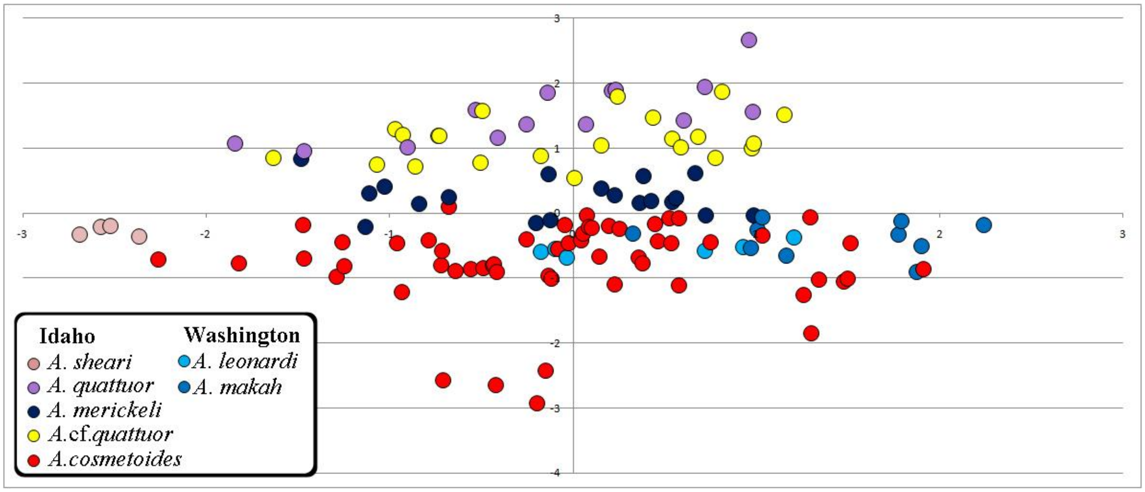

Figure Supplement D.54. Exhaustive components PCA: 1 and 3 – males: covariance matrix. Plotting principle components 1 and 3 recovers *A. sheari* as discrete, and *A. quattuor* and *A.* cf. *quattuor* group together. The first component (48.1% of the variation) is positively weighted for leg segment lengths and scute area II spine height. The third component (13.9%) is negatively weighted on scute area III spine height, and negatively weighted on the eye spine to scute area II spine distance.

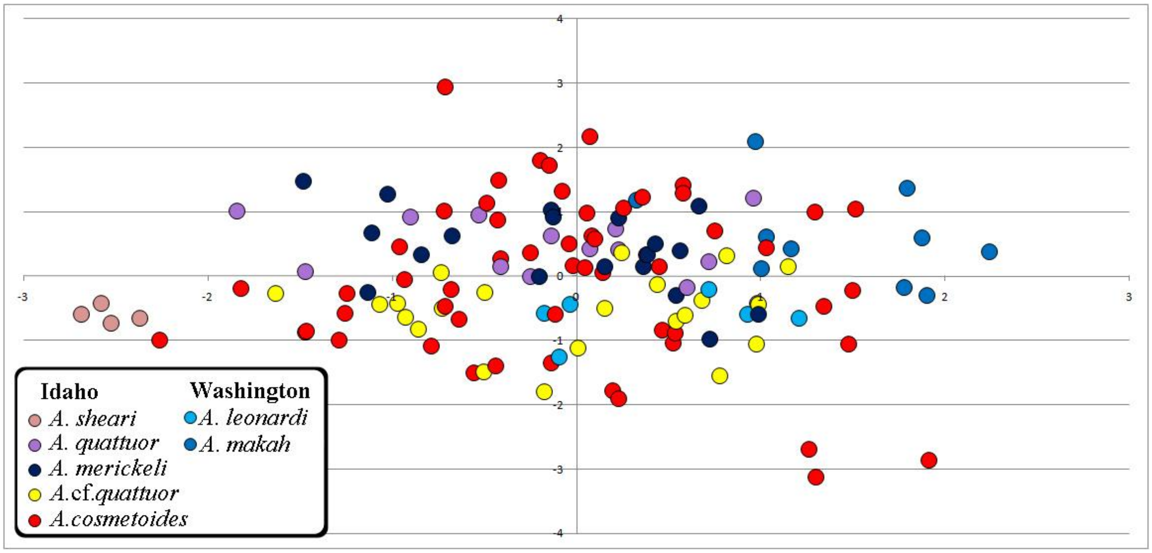

Figure Supplement D.55. Exhaustive components PCA: 1 and 4 – males: covariance matrix. Plotting principle components 1 (information above) and 4 recovers *A. sheari*, which is done by the first component. The fourth component (6.0%) is positively weighted on scute area II spine height, and negatively weighted on Leg II tarsus length.

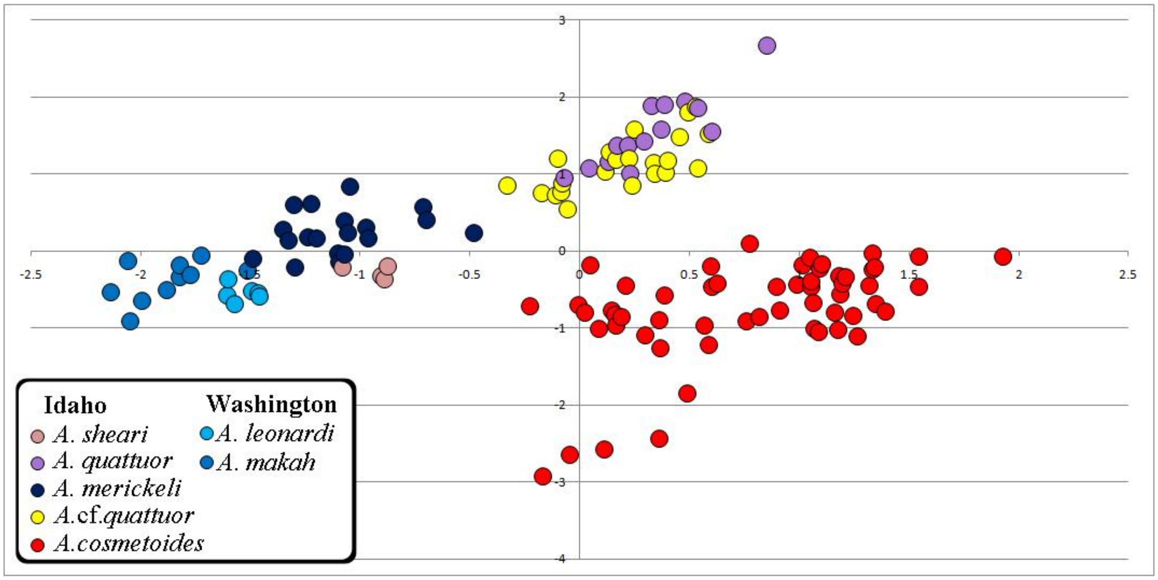

Figure Supplement D.56. Exhaustive components PCA: 2 and 3 – males: covariance matrix. Plotting components 2 and 3 recovers all hypothesized species except for *A. quattuor* and *A.* cf. *quattuor*. The second component (19.5% of the variation) is positively weighted on scute area I and III spines. The third component (13.9%) is negatively weighted on scute area III spine height, and negatively weighted on eye spine to scute area II spine distance.

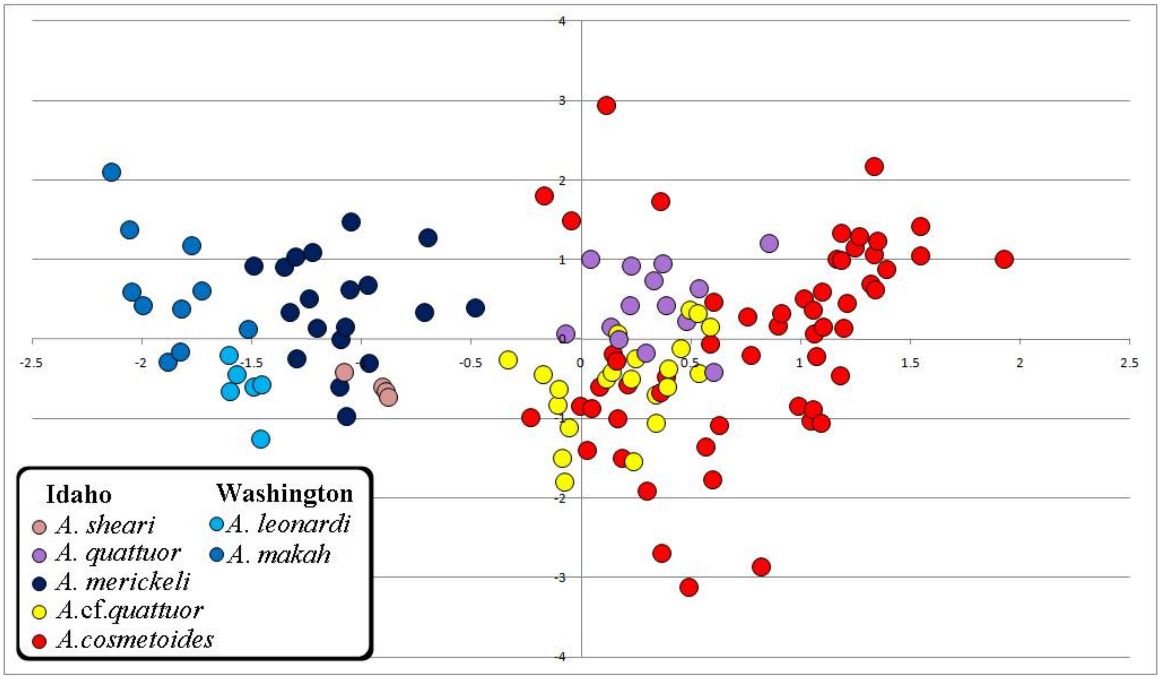

Figure Supplement D.57. Exhaustive components PCA: 2 and 4 – males: covariance matrix. Plotting principle components 2 (information above) and 4 recovers *A. sheari*, *A. merickeli*, *A. leonardi*, and *A. makah* as clusters. The fourth component (6.0%) is positively weighted on scute area II spine height, and negatively weighted on Leg II tarsus length.

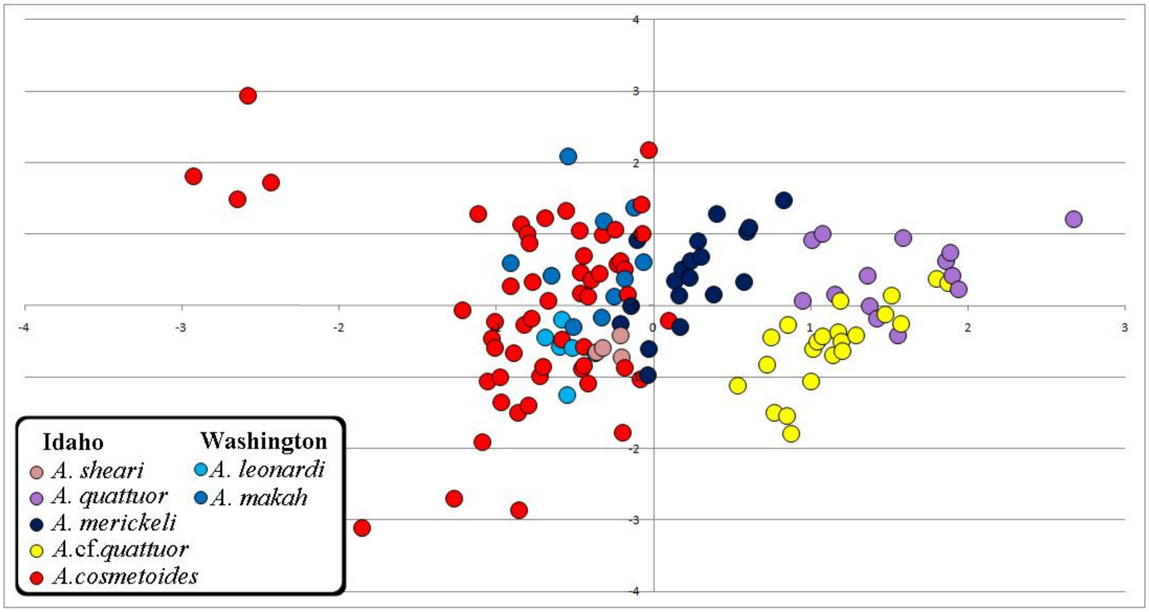

Figure Supplement D.58. Exhaustive components PCA: 3 and 4 – males: covariance matrix. Plotting components 3 and 4 does not recover any hypothesized species, though *A. quattuor* and *A.* cf. *quattuor* cluster together. The third component (13.9% of the variation) is negatively weighted on scute area III spine height, and negatively weighted on eye spine to scute area II spine distance. The fourth component (6.0%) is positively weighted on scute area II spine height, and negatively weighted on Leg II tarsus length.

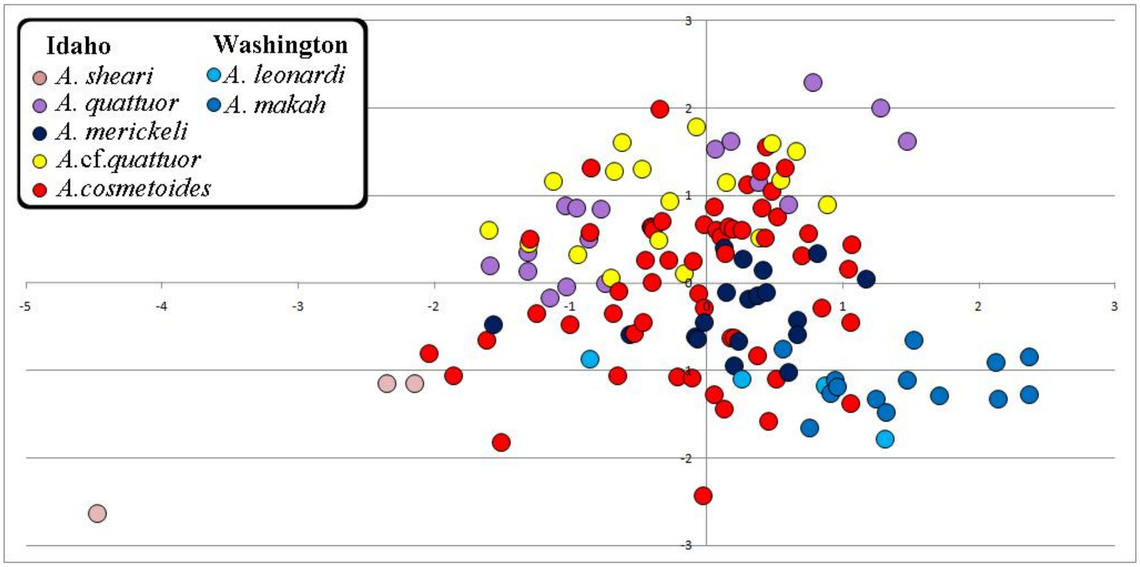

Figure Supplement D.59. Exhaustive components PCA: 1 and 2 – females: covariance matrix. Plotting principle components 1 and 2 recovers only *A. sheari* as discrete. The first component (39.2%) is positively weighted for leg II tibia, femur, tarsus, metatarsus, and scute area II spine height. The second component (19.5%) is positively weighted on eye spine to scute area II spine distance, scute area I spine height, and scute length.

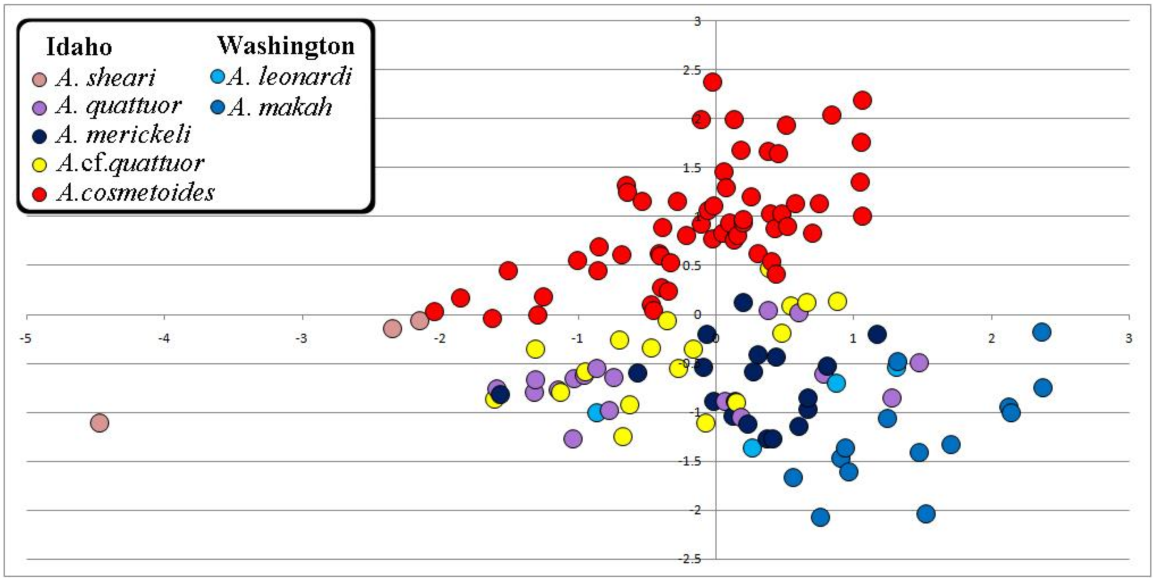

Figure Supplement D.60. Exhaustive components PCA: 1 and 3 – females: covariance matrix. Plotting components 1 and 3 recovers *A. sheari*, and nearly *A. cosmetoides* and *A. makah*. The first component (accounting for 39.2% of the variation in the data) is positively weighted for leg II tibia, femur, tarsus, and metatarsus, and on scute area II spine height. The third component (12.4%) is positively weighted on scute area III spine height, Leg II metatarsus, and negatively weighted on eye spine to area II spine distance.

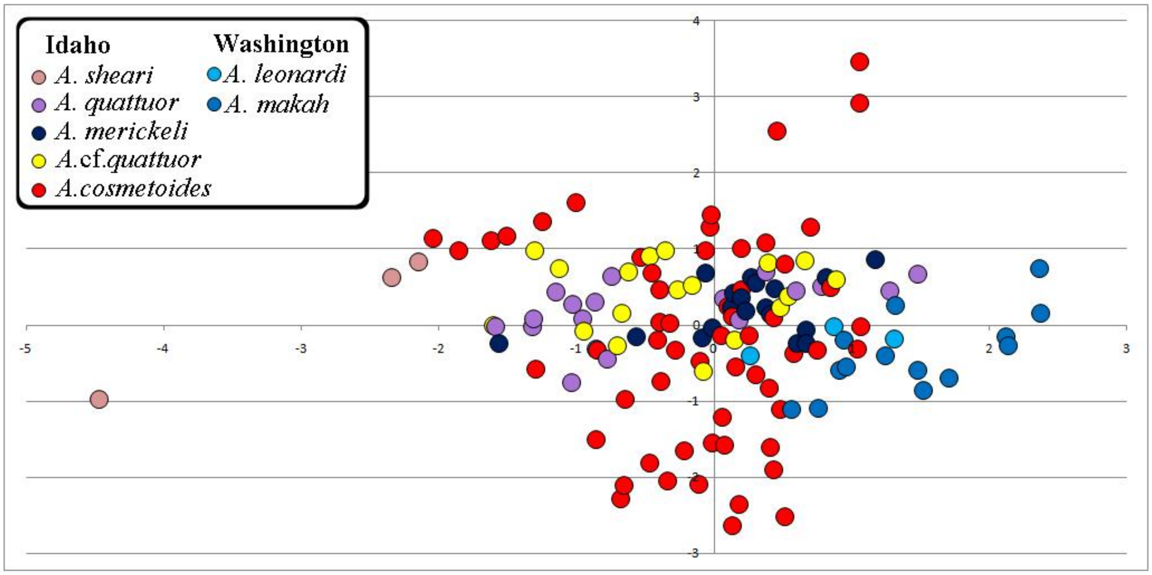

Figure Supplement D.61. Exhaustive components PCA: 1 and 4 – females: covariance matrix. Plotting principle components 1 (information above) and 4 recovers only *A. sheari* as discrete, though *A. makah* nearly clusters independently. The fourth component (9.9%) is negatively weighted on scute area II, III, and IV spine height, and positively weighted on Leg II metatarsus and tarsus.

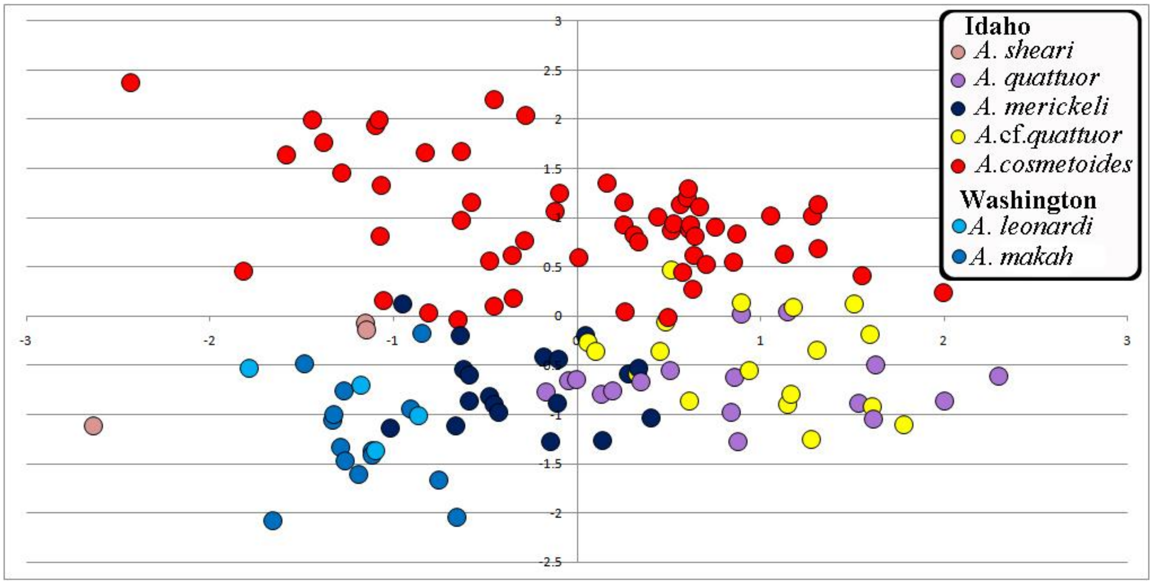

Figure Supplement D.62. Exhaustive components PCA: 2 and 3 – females: covariance matrix. Plotting components 2 and 3 nearly recovers *A. cosmetoides*, *A. sheari*, and WA species as discrete. The second component (accounting for 19.5% of the variation in the data) is positively weighted on eye spine to scute area II spine distance, scute area I spine height, and scute length. The third component (12.4%) is positively weighted on area III spine height, Leg II metatarsus, and negatively weighted on eye spine to area II spine distance.

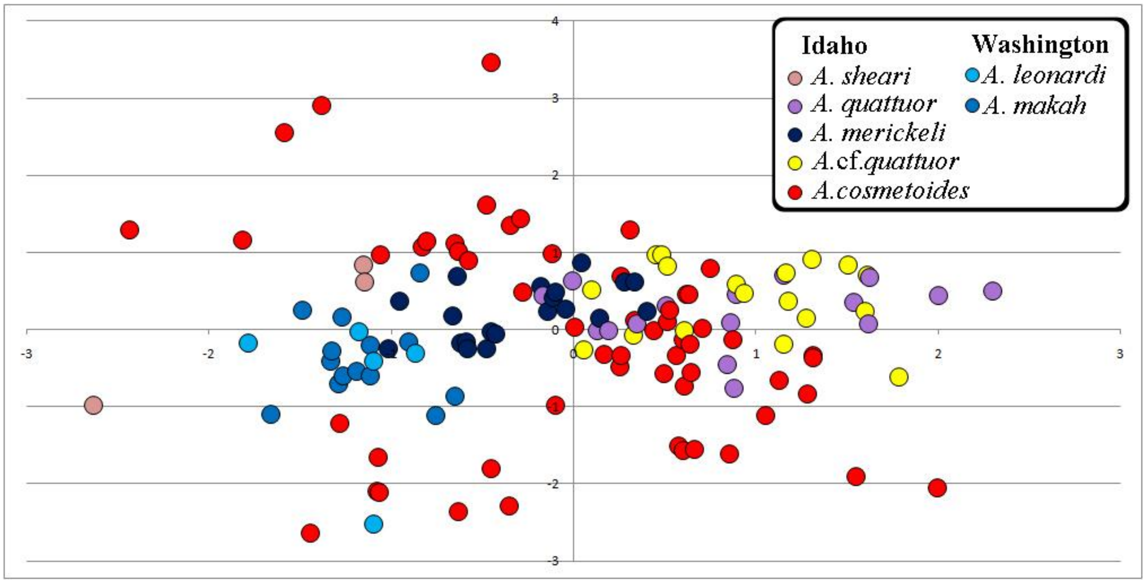

Figure Supplement D.63. Exhaustive components PCA: 2 and 4 – females: covariance matrix. Plotting principle components 2 (information above) and 4 does not recover hypothesized species of *Acuclavella*. The fourth component (9.9%) is negatively weighted on scute area II, III, and IV spine height, and positively weighted on Leg II metatarsus and tarsus.

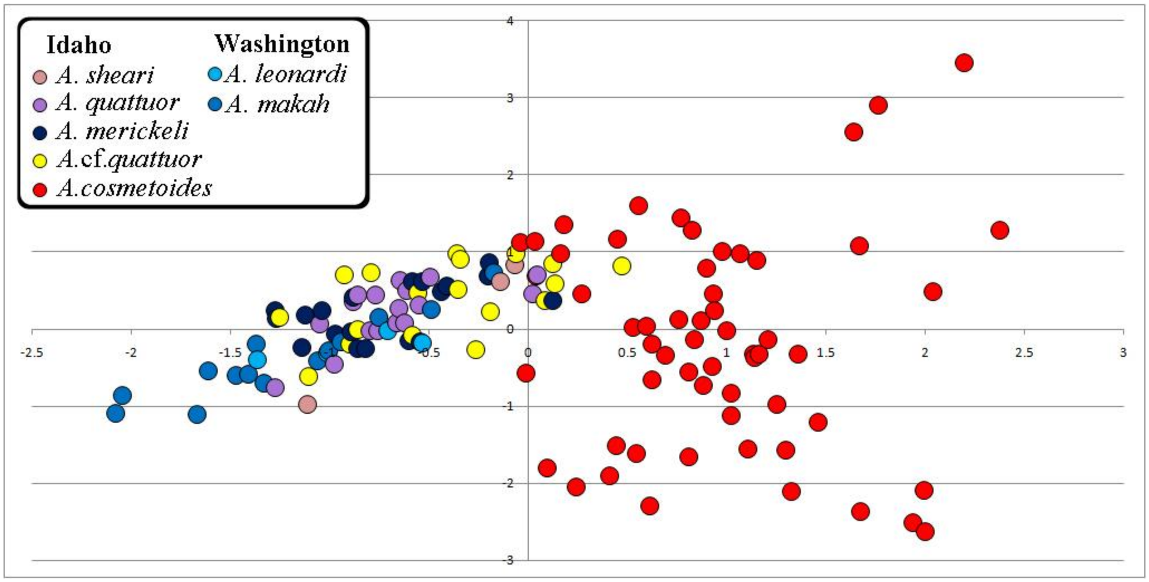

Figure Supplement D.64. Exhaustive components PCA: 3 and 4 – females: covariance matrix. Plotting components 3 are dominated by the variation seen within *A. cosmetoides*, which is nearly discretely recovered. The third component (accounting for 12.4% of the variation in the data) is positively weighted on area III spine height, Leg II metatarsus, and negatively weighted on eye spine to area II spine distance. The fourth component (9.9%) is negatively weighted on scute area II, III, and IV spine height, and positively weighted on Leg II metatarsus and tarsus length.

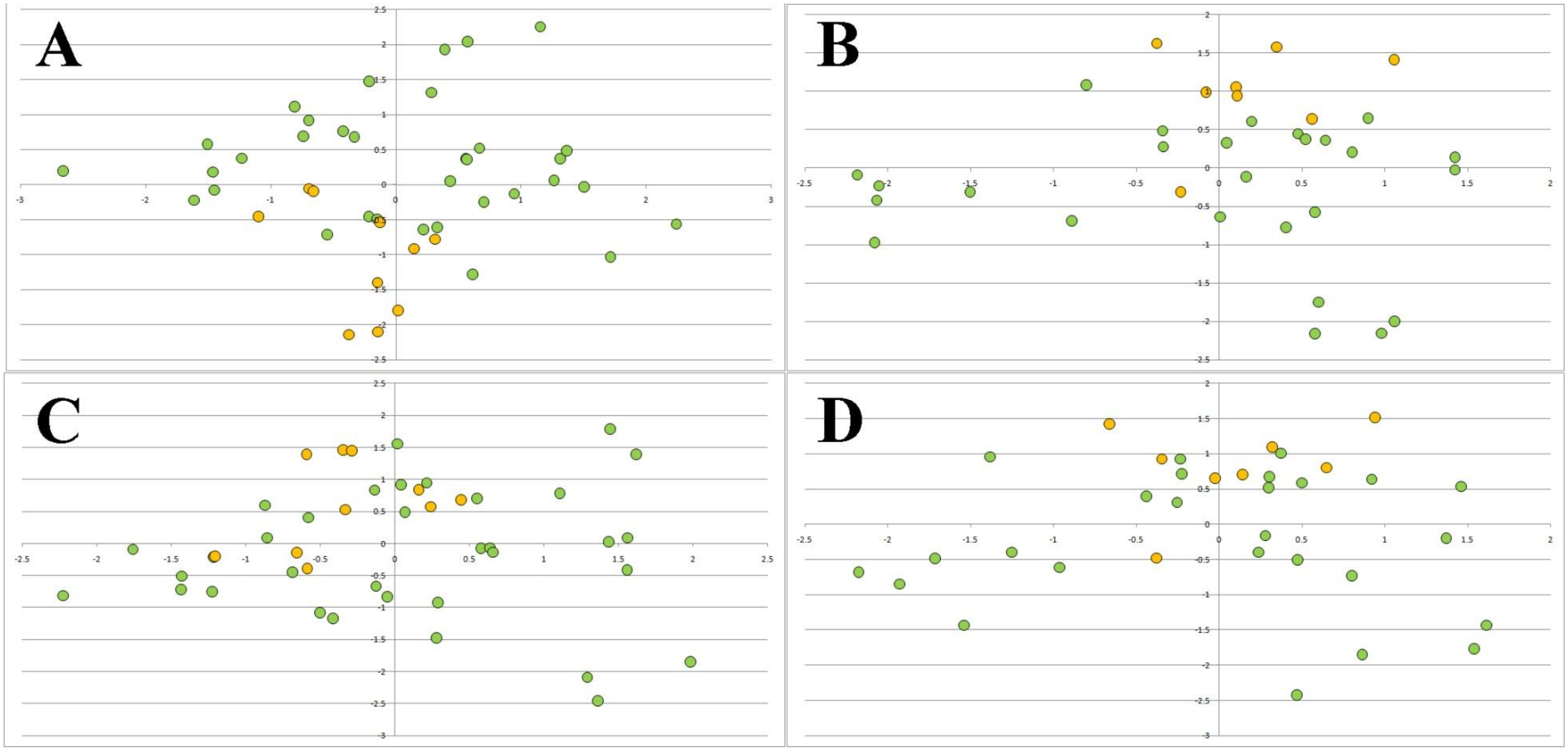

Figure Supplement D.65. Pairwise PCA, *A. cosmetoides* molecular groups green and orange from EF-1α analysis. A. Male data set on correlation matrix; B. Female data set on correlation matrix; C. Male data set on covariance matrix; D. Female data set on covariance matrix.

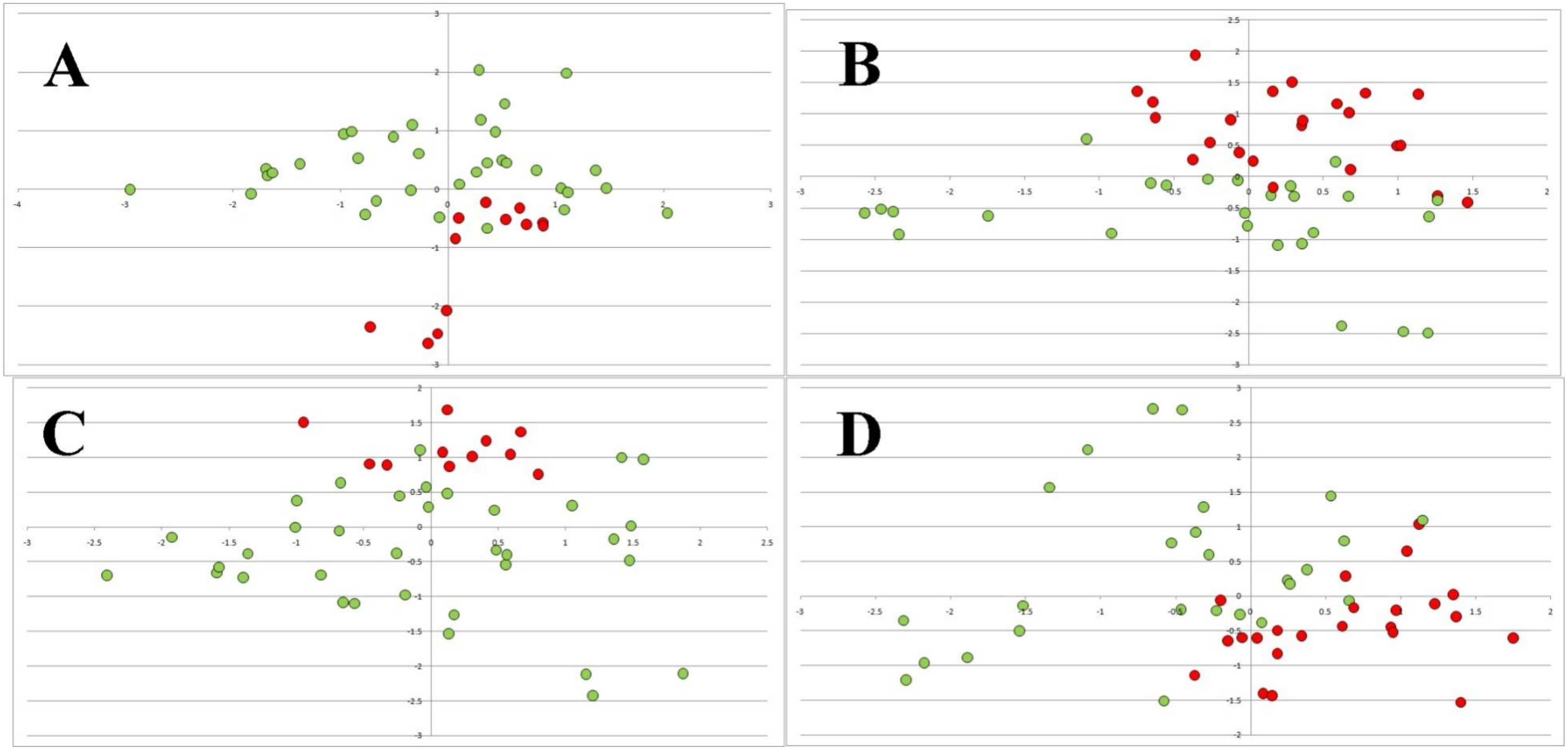

Figure Supplement D.66. Pairwise PCA, *A. cosmetoides* molecular groups green and red from EF-1α analysis. A. Male data set on correlation matrix; B. Female data set on correlation matrix; C. Male data set on covariance matrix; D. Female data set on covariance matrix.

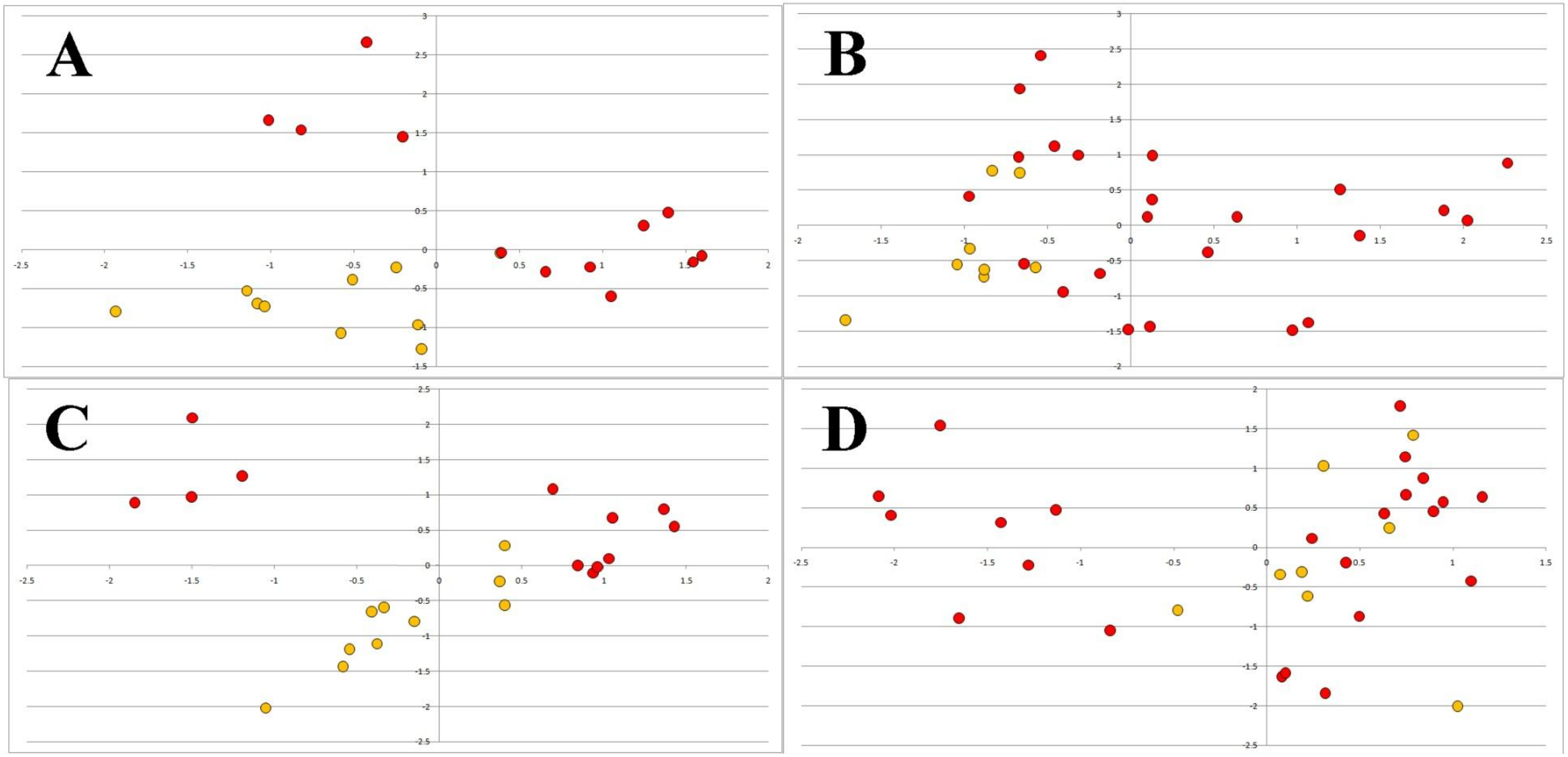

Figure Supplement D.67. Pairwise PCA, *A. cosmetoides* molecular groups orange and red from EF-1α analysis. A. Male data set on correlation matrix; B. Female data set on correlation matrix; C. Male data set on covariance matrix; D. Female data set on covariance matrix.

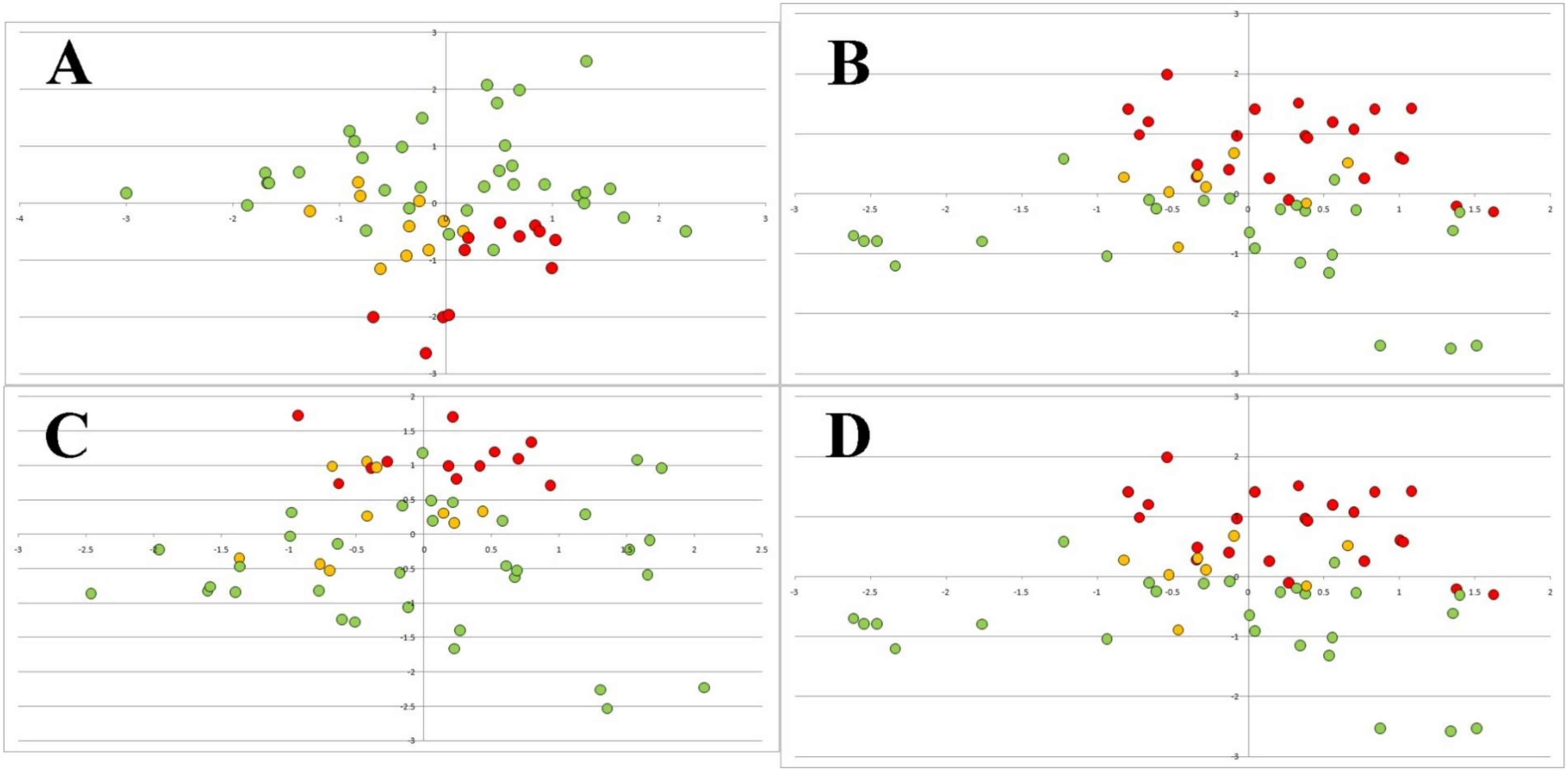

Figure Supplement D.68. Nested PCA, *A. cosmetoides* molecular groups from EF-1α analysis. A. Male data set on correlation matrix; B. Female data set on correlation matrix; C. Male data set on covariance matrix; D. Female data set on covariance matrix. No clades are recovered; further nesting unnecessary
