# Supplementary material for: Three new species in the harvestmen genus Acuclavella (Opiliones, Dyspnoi, Ischyropsalidoidea), including description of male Acuclavella quattuor Shear, 1986
Source: Zookeys. 2013 Jun 20;(311):19–68. doi: 10.3897/zookeys.311.2920 (PMC3698555; doi:10.3897/zookeys.311.2920)
Supplement: Supplementary file 11 — Morphologies of Acuclavella cf. cosmetoides and Geography of Morphotypes. Figure 1: male morphologies. Figure 2: female morphologies. Figure 3: geographic distribution of male morphologies. Figure 4:geographic distribution of female morphologies. (doi: 10.3897/zookeys.311.2920.app5) File format: Adobe PDF file (pdf). [file ZooKeys-311-019-s005.pdf]

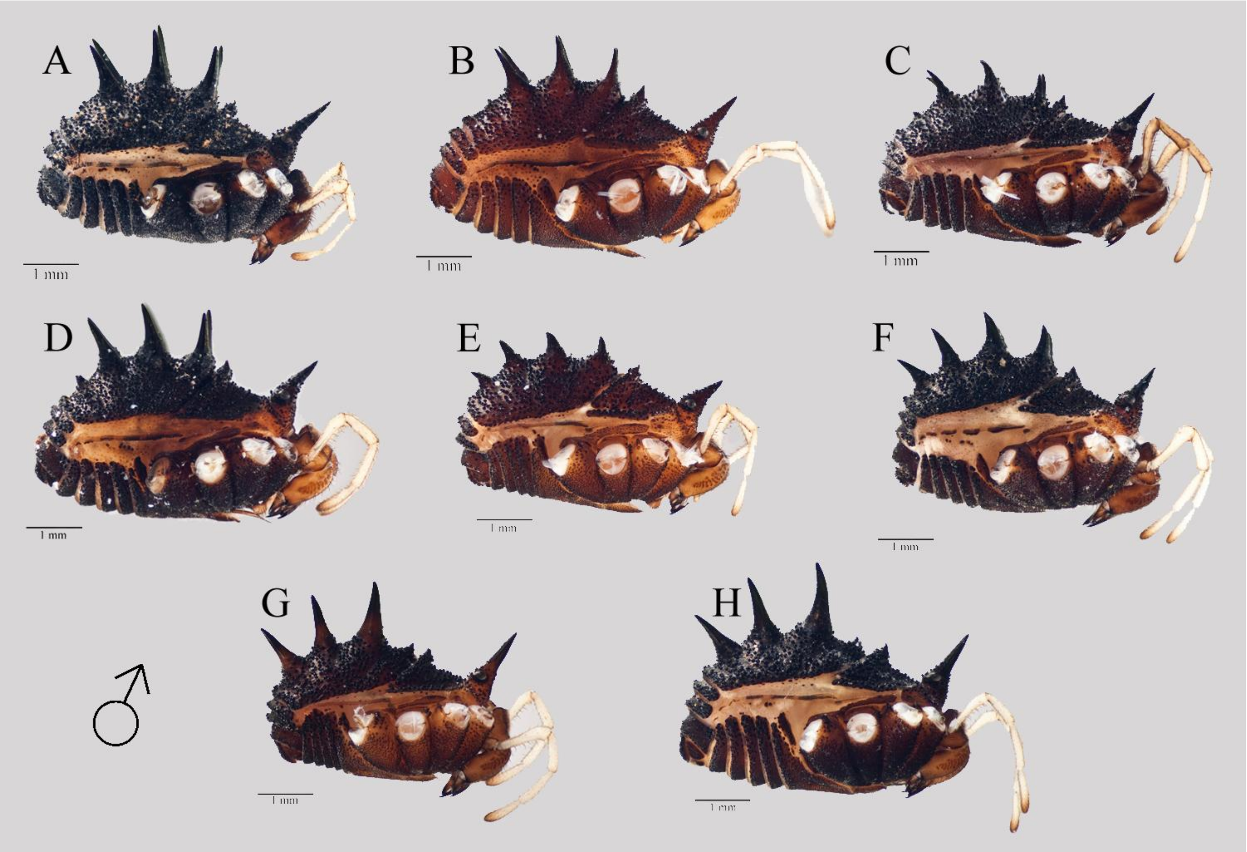

Male *Acuclavella* spine morphologies found north of the Clearwater River in northern Idaho: A. CHR2248.2, B. CHR2312.1, C. CHR2368.1, D. CHR2200.1, E. CHR2414.0, F. CHR2348.1, G. 2264.2, H. 2264.0. CHR2264.0 and CHR2264.2 are from the *A. cosmetoides* type locality and agree in the diagnostic figures outlined by Shear (1986). CHR2414.0 and CHR2348.1 have the diagnostic features defined for *A. shoshone*.

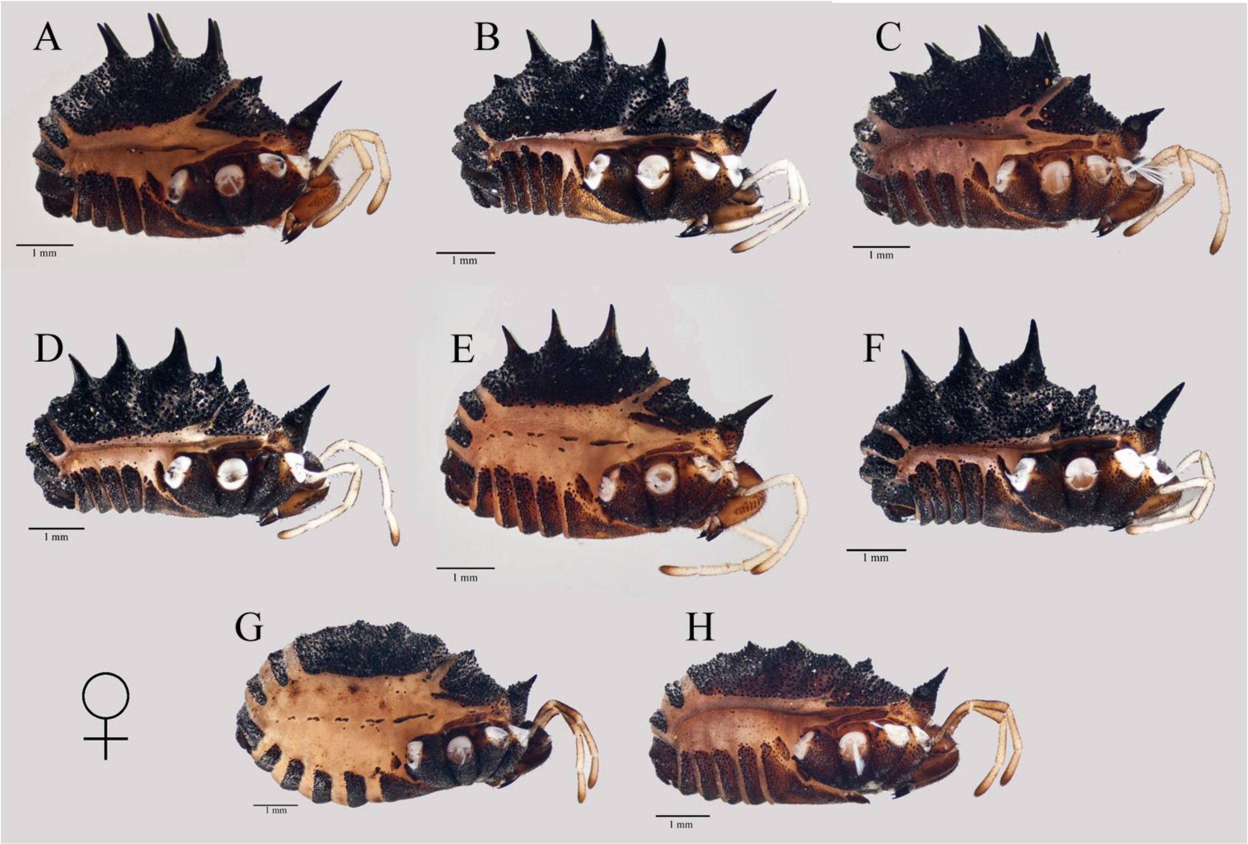

Female *Acuclavella* spine morphologies found north of the Clearwater River in northern Idaho: A. CHR2248.3, B. CHR2258, C. CHR2341.1, D. CHR2268, E. CHR2264.3, F. CHR2322, G. 2359.1, H. 2368.3. CHR2264.3 is from the type locality for *A. cosmetoides*, CHR2359.1 is from the type locality for *A. shoshone*; both agree in the diagnostic features outlined by Shear (1986).

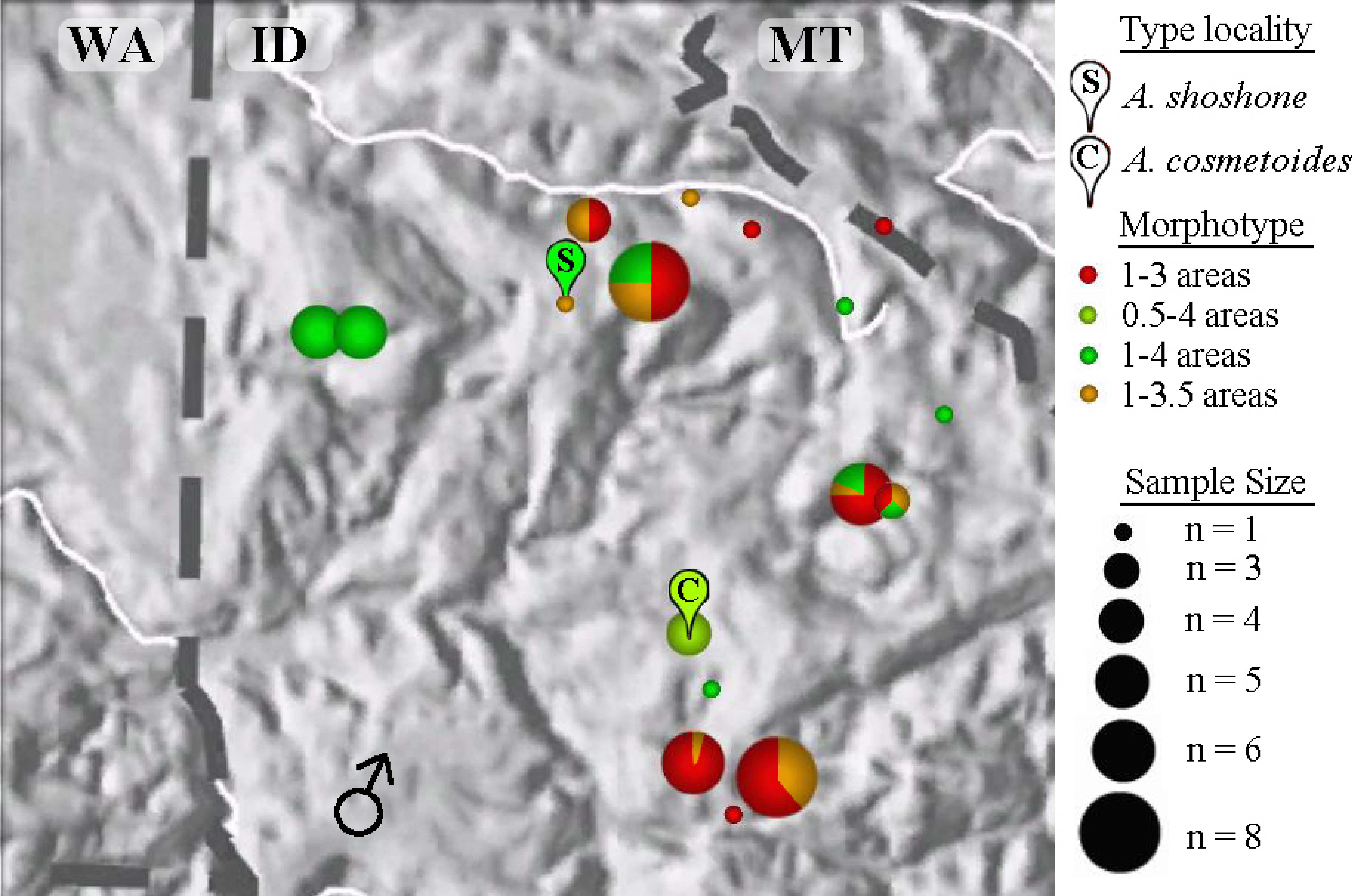

WA
ID
MT
S
C
♂
Type locality
S A. shoshone
C A. cosmetoides
Morphotype
1-3 areas
0.5-4 areas
1-4 areas
1-3.5 areas
Sample Size
n = 1
n = 3
n = 4
n = 5
n = 6
n = 8

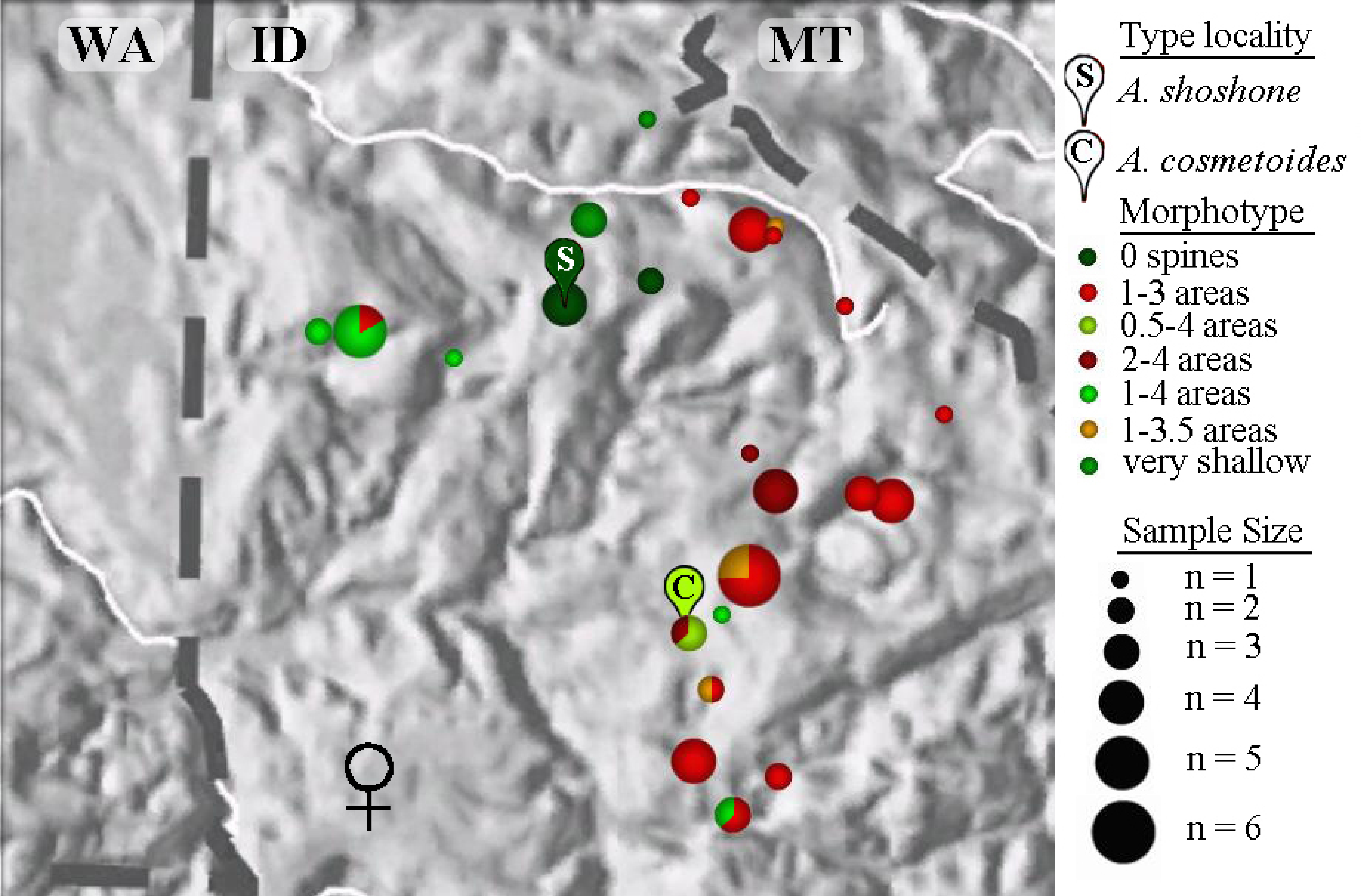

WA
ID
MT
S
C
Type locality
A. shoshone
A. cosmetoides
Morphotype
0 spines
1-3 areas
0.5-4 areas
2-4 areas
1-4 areas
1-3.5 areas
very shallow
Sample Size
n = 1
n = 2
n = 3
n = 4
n = 5
n = 6
