# Supplementary material for: Three new species in the harvestmen genus Acuclavella (Opiliones, Dyspnoi, Ischyropsalidoidea), including description of male Acuclavella quattuor Shear, 1986
Source: Zookeys. 2013 Jun 20;(311):19–68. doi: 10.3897/zookeys.311.2920 (PMC3698555; doi:10.3897/zookeys.311.2920)

Supplement H Phylogenetic Trees

Figure H.1 Outgroup taxa for Bayesian gene tree analyses showing posterior probabilities.

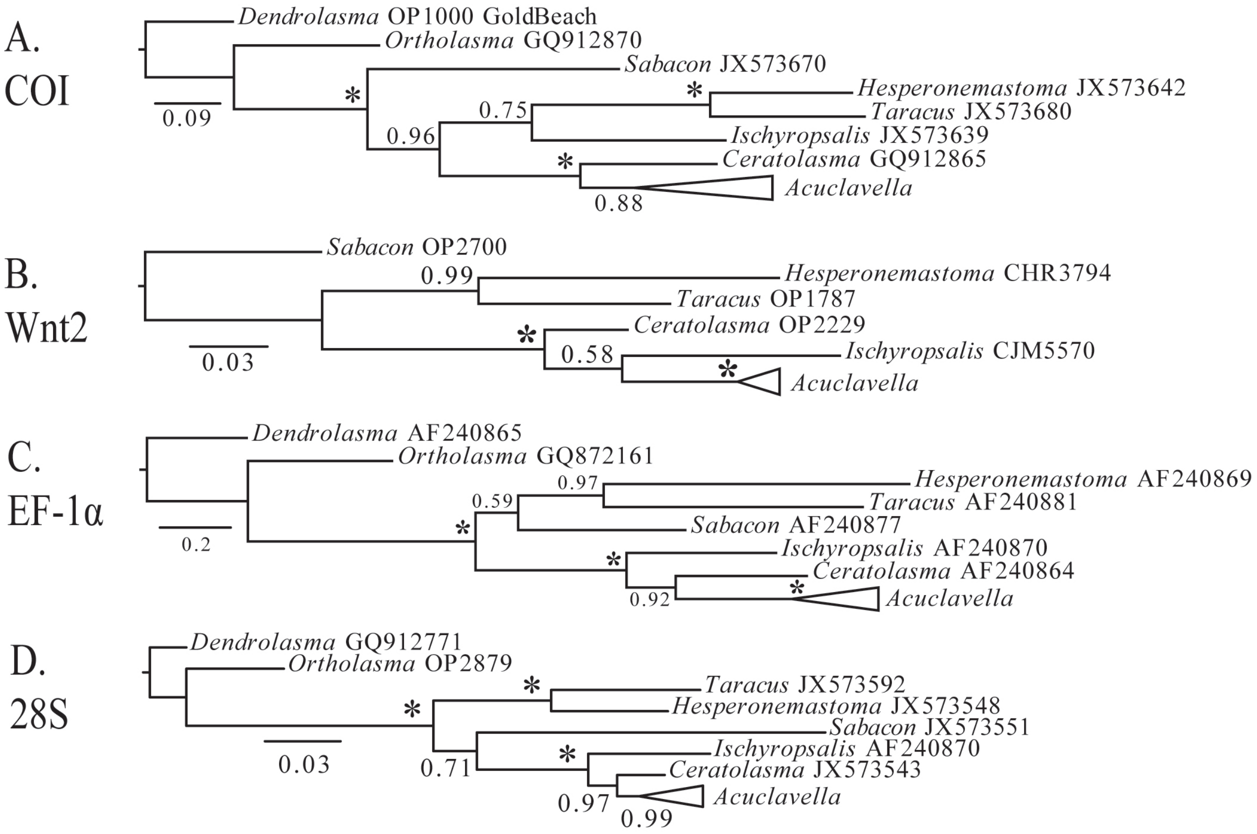

Figure H.2 Concatenated RAxML phylogeny showing bootstrap values.

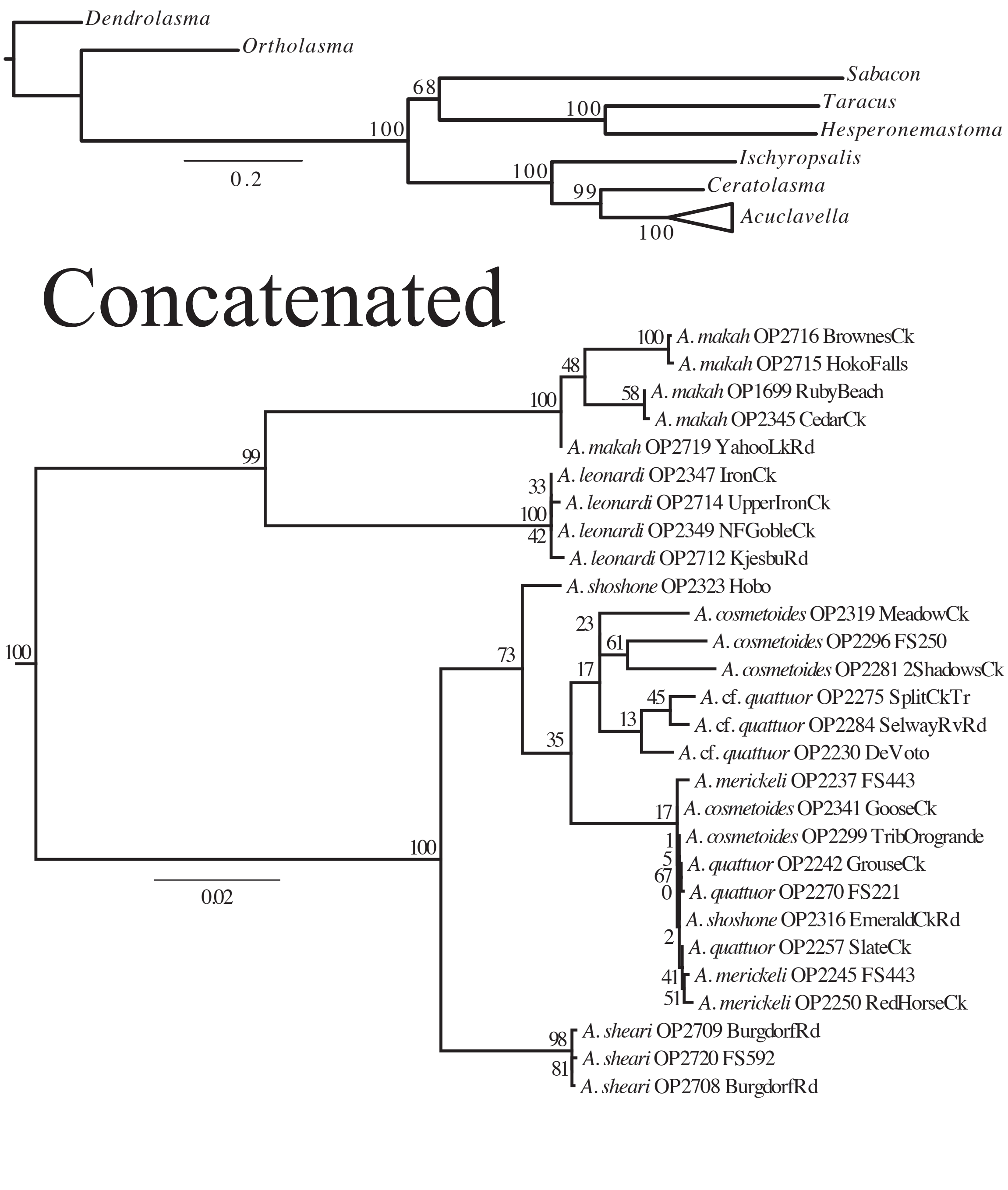

Figure H.3 RAxML COI gene tree showing bootstrap values.

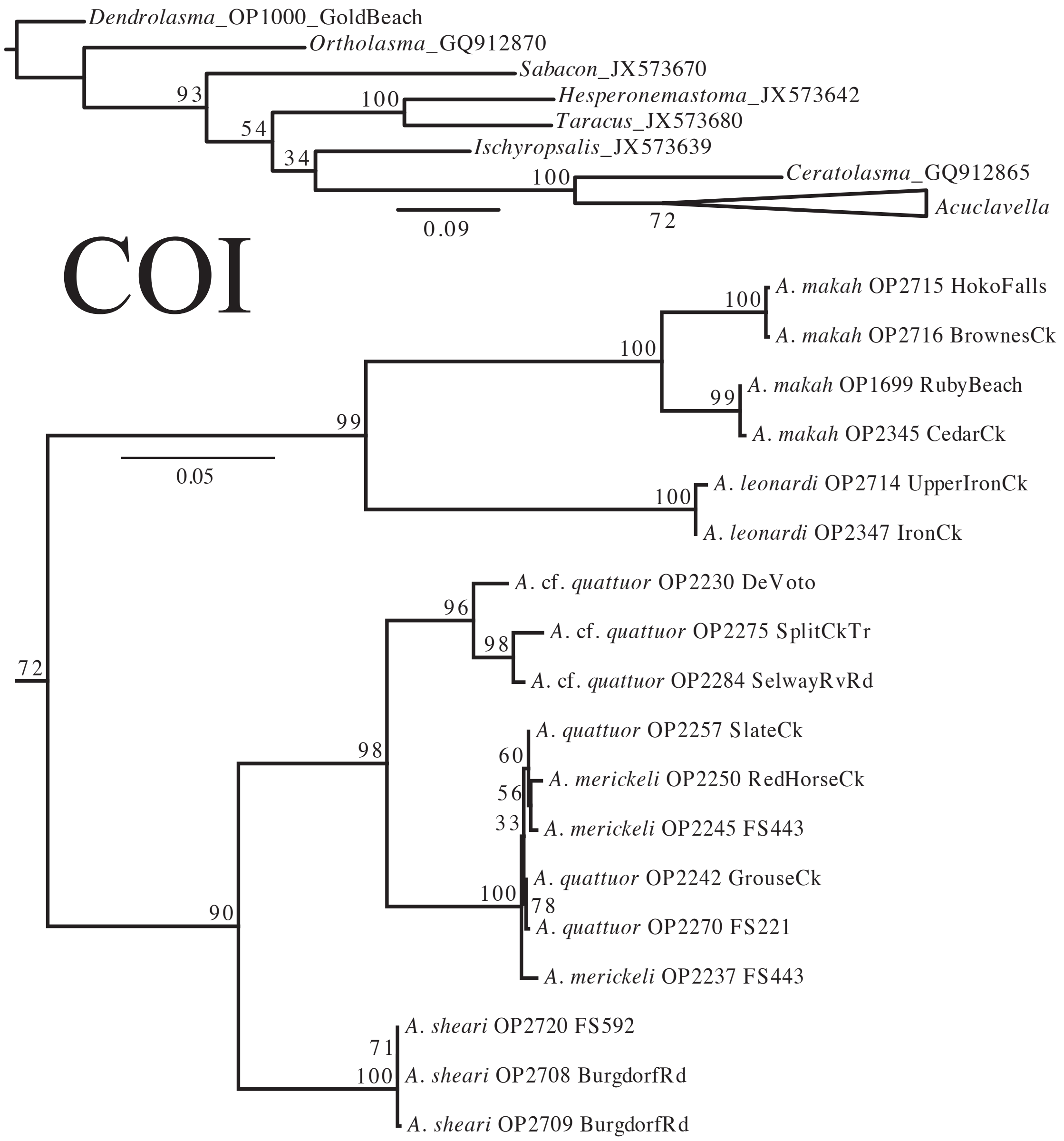

Figure H.4 RAxML EF-1α gene tree showing bootstrap values.

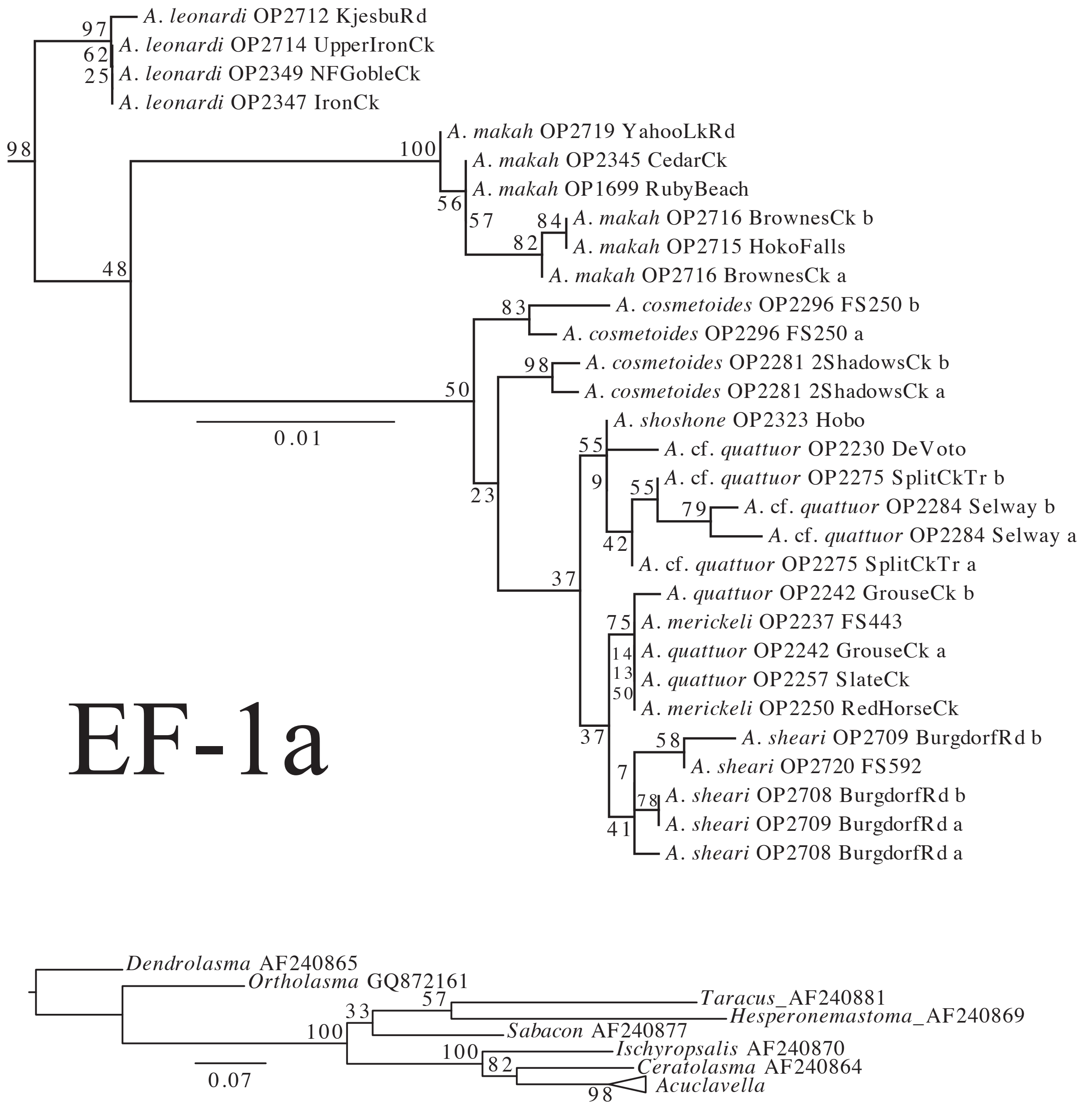

Figure H.5 RAxML 28S and Wnt2 gene trees showing bootstrap values.

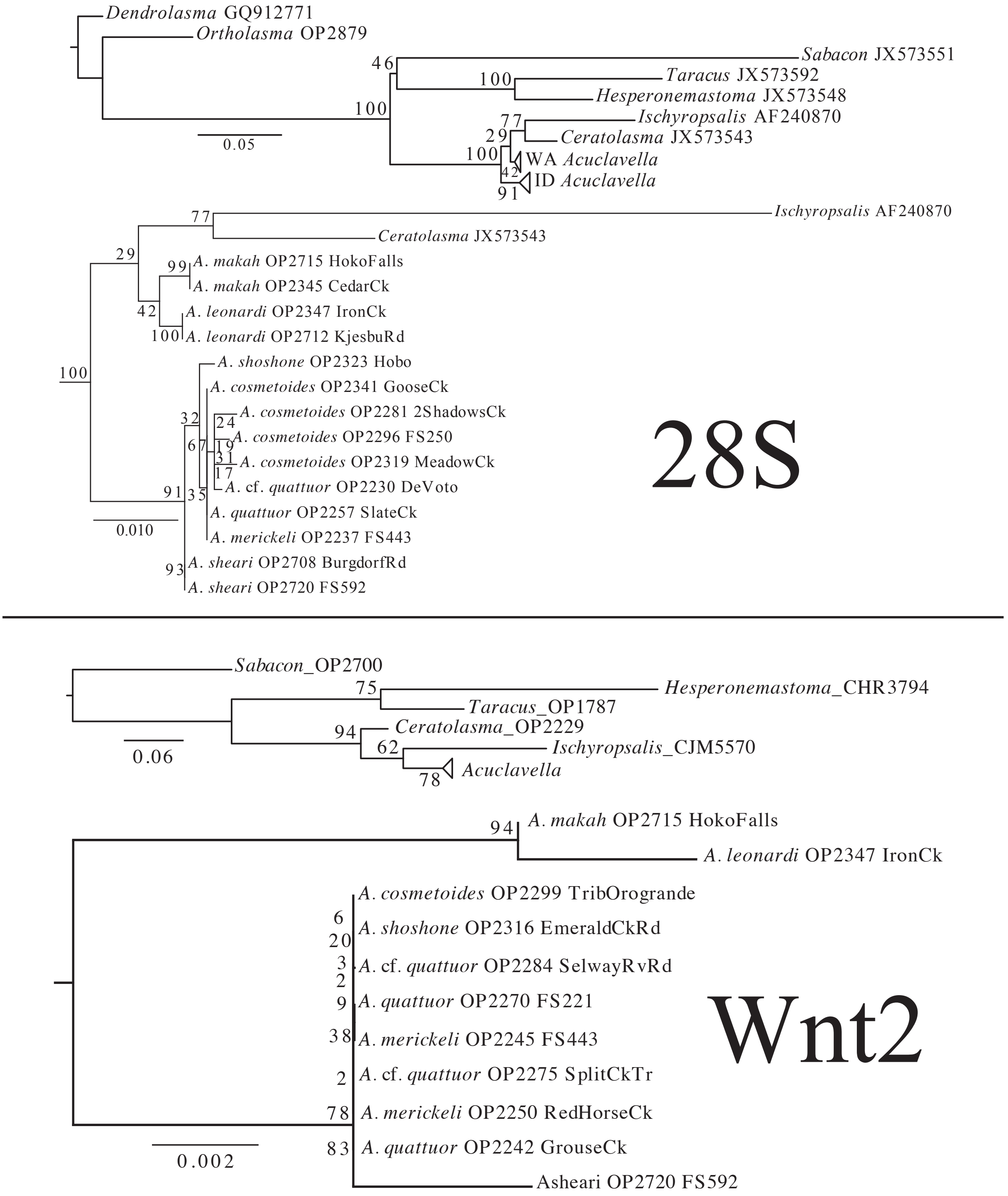

Supplement: Supplementary file 13 — Phylogenetic Trees. Figure 1: Outgroup relationships from Bayesian gene trees. Figure 2: RAxML concatenated phylogeny. Figure 3: RAxML COI gene tree. Figure 4: RAxML EF-1α gene tree. Figure 5: 28S and Wnt2 RAxML gene trees. (doi: 10.3897/zookeys.311.2920.app7) File format: Adobe PDF file (pdf). [file ZooKeys-311-019-s007.pdf]
