# Supplementary material for: Three new species in the harvestmen genus Acuclavella (Opiliones, Dyspnoi, Ischyropsalidoidea), including description of male Acuclavella quattuor Shear, 1986
Source: Zookeys. 2013 Jun 20;(311):19–68. doi: 10.3897/zookeys.311.2920 (PMC3698555; doi:10.3897/zookeys.311.2920)
Supplement: Supplementary file 14 — Acuclavella genitalia. Figure 1: penises. Figure 2: ovipositors. (doi: 10.3897/zookeys.311.2920.app8) File format: Adobe PDF file (pdf). [file ZooKeys-311-019-s008.pdf]

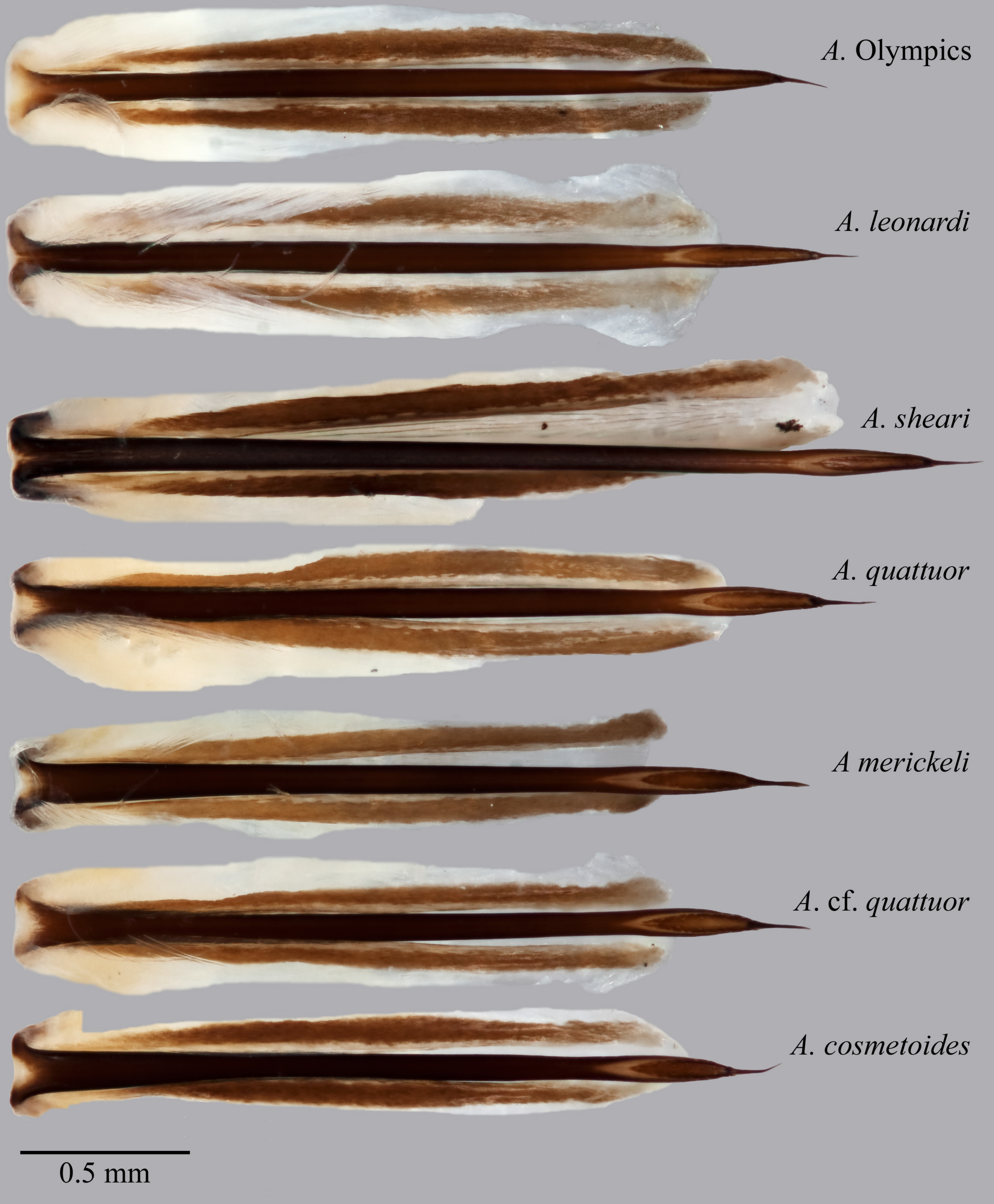
A. Olympics
A. leonardi
A. sheari
A. quattuor
A merickeli
A. cf. quattuor
A. cosmetoides
0.5 mm

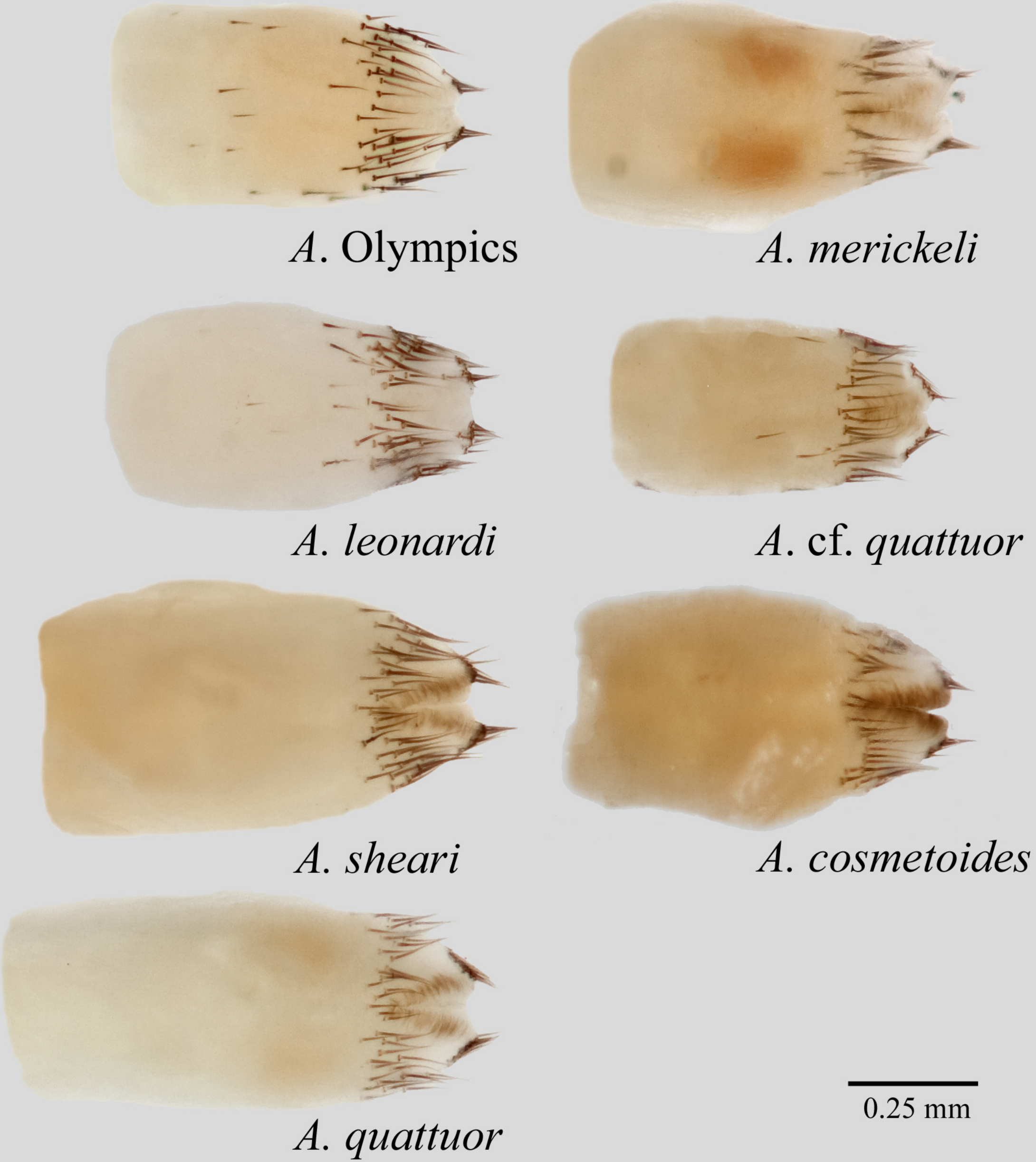
A. Olympics
A. merickeli
A. leonardi
A. cf. *quattuor*
A. sheari
A. cosmetoides
A. quattuor
0.25 mm
