# Supplementary figures and images for: Three new species in the harvestmen genus Acuclavella (Opiliones, Dyspnoi, Ischyropsalidoidea), including description of male Acuclavella quattuor Shear, 1986
Source: Zookeys. 2013 Jun 20;(311):19–68. doi: 10.3897/zookeys.311.2920 (PMC3698555; doi:10.3897/zookeys.311.2920)

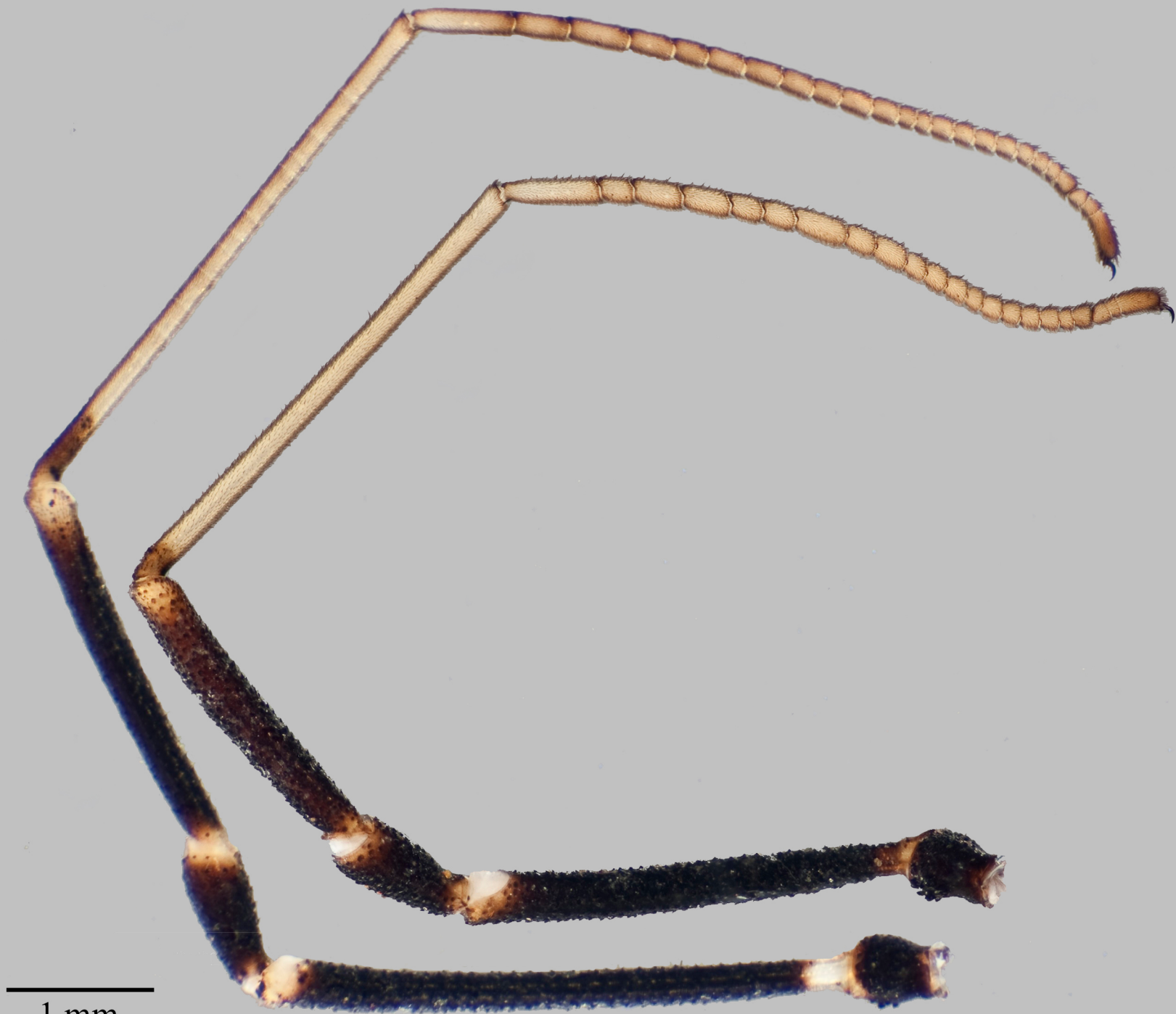

1 mm

Supplement: Supplementary file 15 — K. Leg II Morphologies. With and without femoral false leg articulations. (doi: 10.3897/zookeys.311.2920.app9) File format: Adobe PDF file (pdf). [file ZooKeys-311-019-s009.pdf]
